# Genome-wide exploration of the molecular evolution and regulatory network of mitogen-activated protein kinase cascades upon multiple stresses in *Brachypodium distachyon*

**DOI:** 10.1186/s12864-015-1452-1

**Published:** 2015-03-24

**Authors:** Min Jiang, Feng Wen, Jianmei Cao, Peng Li, Jessica She, Zhaoqing Chu

**Affiliations:** Shanghai Chenshan Plant Science Research Center, Shanghai Chenshan Botanical Garden, Shanghai Key Laboratory of Plant Functional Genomics and Resources, Shanghai Institutes for Biological Sciences, Chinese Academy of Sciences, 3888 Chenhua Road, 201602 Shanghai, Songjiang China; Current address: College of Pharmacy and Life Science, Jiujiang University, Jiujiang, 332000 China; Program of Biochemistry and Immunology, University of Toronto, 100 St. George Street, Toronto, M5G 3G3 Ontario Canada

**Keywords:** MAPK cascade kinases, *Brachypodium distachyon*, Evolution, Gene expression, Abiotic and biotic stresses

## Abstract

**Background:**

*Brachypodium distachyon* is emerging as a widely recognized model plant that has very close relations with several economically important Poaceae species. MAPK cascade is known to be an evolutionarily conserved signaling module involved in multiple stresses. Although the gene sequences of *MAPK* and *MAPKK* family have been fully identified in *B. distachyon*, the information related to the upstream *MAPKKK* gene family especially the regulatory network among MAPKs, MAPKKs and MAPKKKs upon multiple stresses remains to be understood.

**Results:**

In this study, we have identified MAPKKKs which belong to the biggest gene family of MAPK cascade kinases. We have systematically investigated the evolution of whole *MAPK* cascade kinase gene family in terms of gene structures, protein structural organization, chromosomal localization, orthologs construction and gene duplication analysis. Our results showed that most BdMAPK cascade kinases were located at the low-CpG-density region, and the clustered members in each group shared similar structures of the genes and proteins. Synteny analysis showed that 62 or 21 pairs of duplicated orthologs were present between *B. distachyon* and *Oryza sativa,* or between *B. distachyon* and *Arabidopsis thaliana* respectively. Gene expression data revealed that BdMAPK cascade kinases were rapidly regulated by stresses and phytohormones. Importantly, we have constructed a regulation network based on co-expression patterns of the expression profiles upon multiple stresses performed in this study.

**Conclusions:**

*BdMAPK* cascade kinases were involved in the signaling pathways of multiple stresses in *B. distachyon*. The network of co-expression regulation showed the most of duplicated *BdMAPK* cascade kinase gene orthologs demonstrated their convergent function, whereas few of them developed divergent function in the evolutionary process. The molecular evolution analysis of identified MAPK family genes and the constructed MAPK cascade regulation network under multiple stresses provide valuable information for further investigation of the functions of *BdMAPK* cascade kinase genes.

**Electronic supplementary material:**

The online version of this article (doi:10.1186/s12864-015-1452-1) contains supplementary material, which is available to authorized users.

## Background

*Brachypodium distachyon* is emerging as a widely recognized model plant of the grass subfamily Pooideae whose genome is completely sequenced [1]. The plant has a very close relationship with several economically important Poaceae species such as *Oryza sativa*, *Sorghum bicolor*, *Triticum aestivum* and turf grasses. The study of the *B. distachyon* genome will help scientists better understand the mechanism of gene-controlled physiological processes in Poaceae, and subsequently improve the abiotic or biotic stress tolerance of crops and turf grasses through gene engineering.

Mitogen-activated protein kinases (MAPKs) cascades are evolutionarily conserved signaling modules, which are involved in controlling many cellular functions, including cell division, development and multiple stresses in all eukaryotes [2-4]. Activated MAPK cascade kinases can regulate the phosphorylation level of transcription factors and other components related to the MAPK pathway. For example, reactive oxygen species (ROS) can active MEKK1-MPK4 cascade, and the activated MAPK cascade further regulates the ROS-responsive gene expression and other MAPKs [5]. Previous findings indicated that the MAPK cascade of MEKK1-MKK4/MKK5-MPK3/MPK6 was responsible for signal transmission when a plant recognized flagellin, and then triggered the disease resistant responses [6,7]. The MAPK cascade is not only involved in stresses responses, but also plays important roles in response to phytohormone. For instance, the MAPK cascade of MKK3–MPK6 has been proved as an important part of jasmonic acid (JA) signal transduction pathway in *Arabidopsis thaliana* [8,9]. Generally, a MAPK cascade contains three functionally conserved components: MAPKs, MAPK Kinases (MAPKKs/MKKs) and MAPKK Kinases (MAPKKKs/MEKKs). The external stimuli, once perceived by a membrane receptor, could translate them into cellular response signals, resulting in subsequent phosphorylation of MAPKKKs. The kinase-activation process of MAPKKKs initiates with the activation of MAPKKs by phosphorylating the serine and threonine residues in the S/TXXXXXS/T motif, then MAPKs at the last step of MAPK cascade through phosphorylation of both tyrosine and threonine residues in the TXY motif [4,10].

To date, numerous studies on plant MAPKs (also called MPKs) were conducted by many scientists [11]. Similar to animal ERK kinases, plants MAPKs also have two phosphorylation motifs of TDY and TEY. *A. thaliana* MPKs, which have been reported to classify into four groups, are involved in developmental processes and the activation in response to biotic and abiotic stresses [12-14]. In *A. thaliana*, the groups of A and B including AtMPK3, AtMPK4 and AtMPK6 were well-characterized to respond to a diversity of environmental stimuli. For example, AtMPK4 was activated in a few minutes in response to the flg22 peptide of flagellin, and the activated AtMPK4 controlled the disease resistance gene expression and defense responses, showing a negative regulation in the biotic stress treatment [6,15]. AtMPK3 and AtMPK6 were involved in various environmental stress and hormone responses. Studies also revealed that MPK3 and MPK6 were activated when *A. thaliana* seedlings were treated with flg22. MPK3 and MPK6 were proved to be involved in other signaling pathways which are independent of MPK4 [6,7]. So far, ten MAPKKs in *A. thaliana* and eight MAPKKs in *O. sativa* have been found in response to multiple stresses. The upstream activation of AtMPK4 by AtMKK1 and AtMKK2 were involved in not only the ROS homeostasis and salicylic acid (SA) accumulation, but also abiotic stresses such as cold, salt and wounding [16-18]. OsMEK1, a homolog of AtMKK1, has been demonstrated to play an important role in response to the low-temperature stress at wide ranges in *O. sativa* [19]. MAPKKKs (also named as MAP3Ks and MEKKs), known as the first step of MAPK cascade, constitute a diverse family of kinases which have been grouped into three large subfamilies based on the sequence of the kinase catalytic domain: the MEKK-like family, Raf-like family and ZIK-like family. Similar to mammalian MEKK1 and yeast STE11 and BCK1, plant MEKK-like subfamily members with a conserved catalytic domain are involved in stress responses by activating downstream MKKs [20]. In *A. thaliana*, AtMEKK1, as a downstream kinase of the flagellin receptor FLS2, can trigger a complete plant MAPK cascade (MEKK1, MKK4/MKK5 and MPK3/MPK6), then function as a conservation innate immunity in response to both bacterial and fungal pathogens [7]. The Raf-like family possesses more than half of MAPKKKs members, and the subfamily members share a specific polypeptide signature GTxx (W/Y) MAPE, which are similar to mammalian RAF1 [12,21,22]. The *A. thaliana* Raf-like members, Enhanced Disease Resistance 1 (EDR1) and Constitutive Triple Response 1 (CTR1), shared a homology with mammalian Raf-like MAPKKKs, were reported to participate in ethylene-mediated signaling and defense responses [23-26]. Sister clades such as ZIK-like kinases are presented in MAPKKKs phylogenetic analyses, however, these enzymes have not been shown to phosphorylate MAPKKs in plants [20,27].

So far, many members of MAPK cascades have been identified using functional genomic methods. Twenty MAPKs and 10 MAPKKs have been found in *A. thaliana*, 15 MAPKs and 8 MAPKKs are present in the *O. sativa* genome, while 16 MAPK genes and 12 MAPKK genes were identified from *B. distachyon* Bd21 genome [28]. MAPKKK gene family has been systematically investigated in *A. thaliana*, *O. sativa*, *Zea mays* and *Gossypium raimondii* [21,22,29-31]. In this study, we have systematically identified all MAPK cascade kinase genes including a total of 75 *MAPKKK* genes from *B. distachyon* Bd21 genome*.* We further investigated the evolutionary relationship of *B. distachyon* MAPK cascade kinase genes (MAPKs, MAPKKs and MAPKKKs) in terms of phytogenetic analysis, chromosomal localization and gene duplication with their counterparts from monocot *O. sativa* and dicot *A. thaliana*. Subsequently, we used qRT-PCR to examine their tissue-specific transcription profiles and the profiles in response to several biotic or abiotic stresses. In addition, we analyzed the expression changes of *BdMAPK* cascade kinase genes under those treatments, and established the MAPK signaling network based on the co-expression patterns upon different stresses treatment. The duplicated ortholog pairs of *BdMAPK* cascade kinase genes revealed their convergent or divergent function in the process of evolution. Our study provided the genome-wide evolutionary analyses and expression profiles of *MAPK* cascade kinase genes in *B. distachyon* under multiple-stress conditions, which paved a way for further investigation into MAPK cascade kinase genes functions across different plant species.

## Results and discussion

### Distribution of MAPK cascade kinase genes in the plants

**Figure 1:**
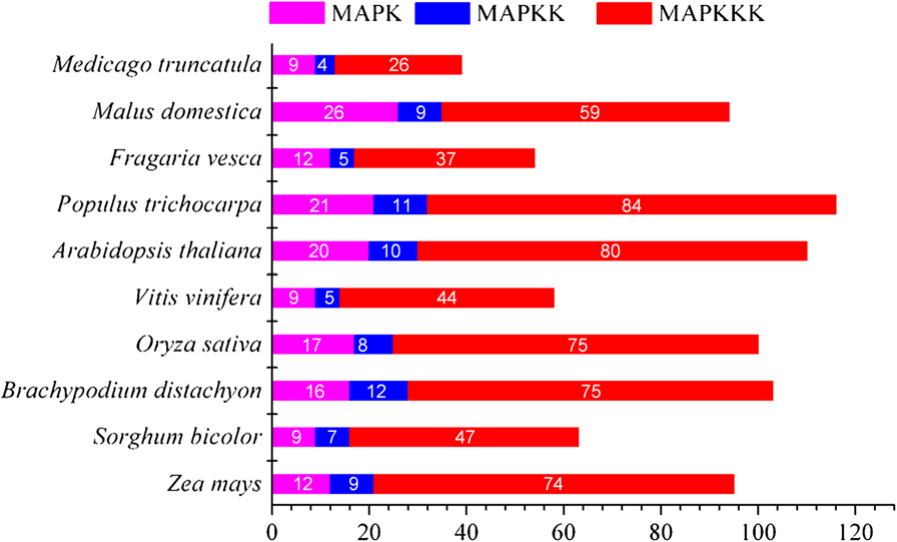
**Distribution of MAPK cascade kinase genes in Plant kingdom.** The total number of MAPK, MAPKK and MAPKKK homologous genes found in each genome is indicated in the bar.

MAPK cascades exist in all eukaryotes and orchestrate diverse cellular activities. Here, we searched for MAPK cascade kinase genes in ten common plant species from the reported data and the Plaza Database (http://bioinformatics.psb.ugent.be/plaza/). The result indicated that these genes play important roles in regulation of many cellular functions in eukaryotes, especially MAPK cascade kinase genes are widely existed in the plants (Figure 1). In general, the number of MAPK family members was more than that of MAPKK gene members. As the first step of the MAPK cascade, MAPKKK genes transmit all kinds of up-stream signals into the MAPK cascade. The MAPKKKs have the largest number among MAPK, MAPKK and MAPKKK gene families. Unfortunately, only a few reports about MAPKKK genes have been found in the literature due to its multiformity [29,30]. To date, 16 MAPK genes and 12 MAPKK genes, but no report about MAPKKK genes were identified from *B. distachyon* genome [28].

### Identification and annotation of the MAPK cascade kinase gene family from the *B. distachyon* genome

The availability of a completed genome of *B. distachyon* offers the feasibility to identify all the MAPK cascade kinase gene family members in the plant. The identification and phylogenetic analyses of MAPK and MAPKK genes have been completed in *B. distachyon* previously, and therefore we used the same annotation of MAPK and MAPKK genes in this study as reported previously (Additional file 1) [28]. In order to identify the MAPKKK genes, 155 queries of the MAPKKK sequences from *A. thaliana* and *O. sativa* were employed for BLASTP analyses against 32255 sequences of the protein database of *B. distachyon* available from MIPS (http://mips.helmholtz-muenchen.de/plant/brachypodium/), which retrieved 163 hits as target sequences [1]. A self BLAST of these sequences followed by manual editing to remove the redundancy has finally identified 75 MAPKKK genes from the *B. distachyon* genome (Additional file 1). Furthermore, we analyzed the sequence homology between putative *BdMAPKKK* genes and the *MAPKKK* gene family in *O. sativa* using the Best Blast Mutual Hit (BBMH) method. Since there was no nomenclature rule of *MAPKKKs* to follow in *A. thaliana* and *O. sativa*, all 75 *MAPKKK* gene family members in *B. distachyon* genome were designated as *BdMAPKKK1*-*BdMAPKKK75* based on the BBMH scores. The amino acid sequence analyses showed that all 75 BdMAPKKKs have a conserved protein kinase domain in the MAPK family. Protein subcellular localization was predicted by WoLF PSORT online analysis, and only the maximum probability was selected. The results revealed that most MAPK cascade kinases varied from the cytoplasm, chloroplast, and mitochondria to the nucleus except for 4 MAPKKKs and 4 MAPKs. For example, BdMAPKKK40 and BdMAPKKK75 were present in the cytoskeleton, whereas BdMAPKKK19 was localized in peroxisomes (Additional file 1).

### Gene and protein structural organization of BdMAPKs, BdMAPKKs and BdMAPKKKs

**Figure 2:**
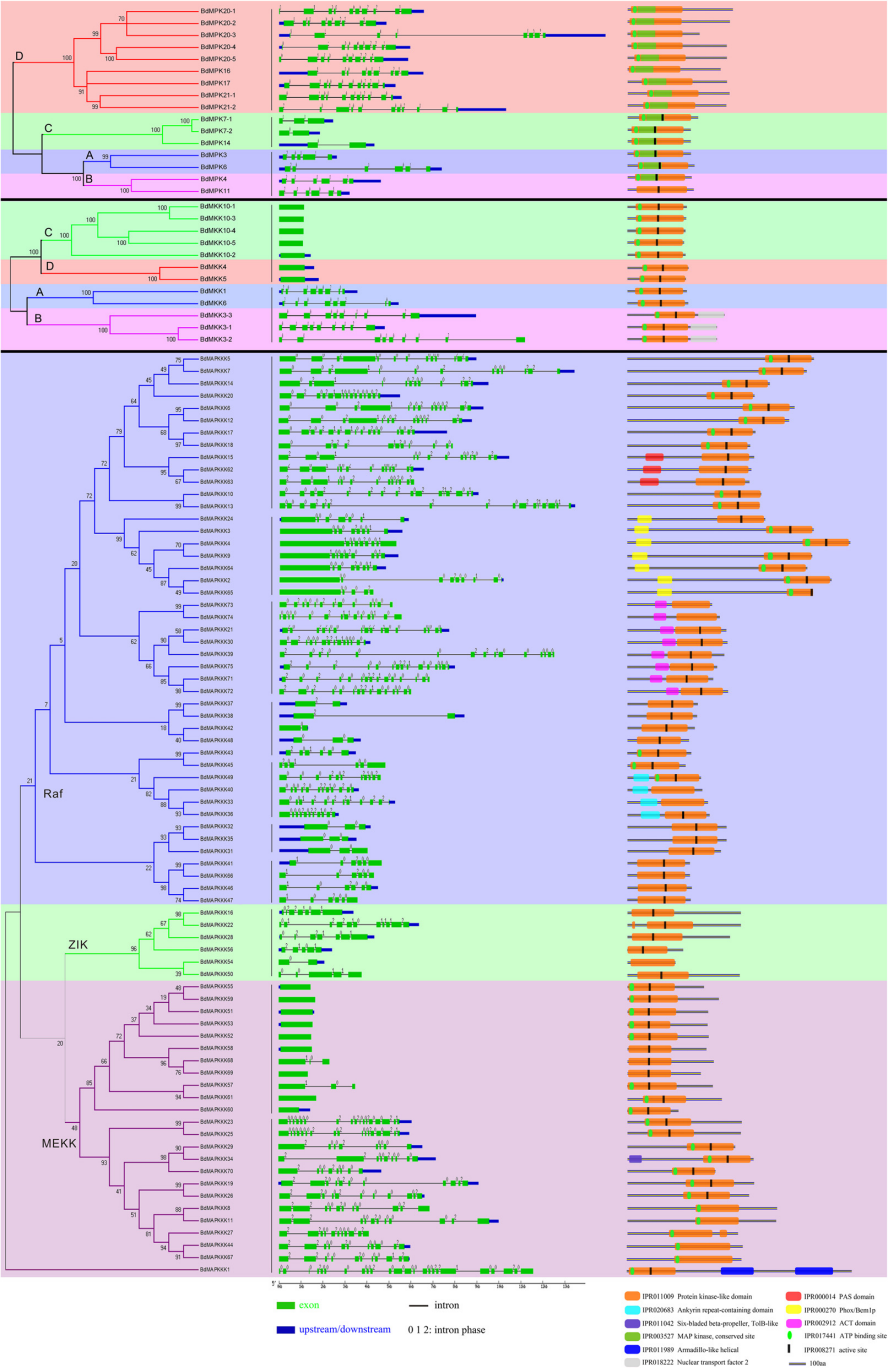
**Phylogenetic relationships (A), gene structures (B) and protein structures (C) analysis of MAPK cascade kinases in** ***B. distachyon*** **.**

In order to seek the evolutionary relationship among MAPK, MAPKK and MAPKKK family genes and further analyze the regulation network among these family genes, phylogenetic trees were constructed from alignments of the full-length MAPK cascade kinase nucleotide sequences using the Neighbor-Joining (NJ) method by MEGA5.0 (Figure 2A). According to the phylogenetic analyses of the nucleotide sequences, *BdMAPKs* were classified into four groups, consistent with the *MAPKs* in *A. thaliana*. Similarly, *BdMAPKKs* were also divided into four groups. The results were consistent with the previous study [28]. Correspondingly, *MAPKKKs* were allocated into three groups based on their sequence alignments: the MEKK-like family, Raf-like family and ZIK-like family. To date, there is no report regarding MAPKKK genes in *B. distachyon*, so searching for MAPKKK family genes in *B. distachyon* is necessary. The phylogenetic analysis indicated that *BdMAPKKKs* can also be divided into three major groups: MEKK, Raf and ZIK. The result showed that the Raf-like family contains large members, while the ZIK-like family only has six BdMAPKKK members.

Next, we analyzed the gene structure, exon position and phases of intron in BdMAPK cascade kinase genes, and 15 kinds of gene structures were identified (Figure 2B). Generally, the MAPK, MAPKK and MAPKKK genes could be divided into 3, 2 and 10 subgroups based on their exon/intron structures, respectively (Figure 2B). As shown in Figure 2B, all the members of the MAPK family had introns, whereas the members of groups C and D in the MAPKK family had no intron. In the Raf-like gene family, the number of introns varied from 1 to 15, whereas the number of exons varied from 2 to 11 in the ZIK-like gene family. Half of the MEKK group genes had only one exon and no intron. The results showed that the BdMAPK cascade kinase genes were consistent with their homologous genes in *A. thaliana*, *O. sativa* and *Zea may*s by gene structure analyses [22]. Additionally, most gene pairs, which were clustered together by phylogenetic analysis, shared a similar exon/intron structure and intron phases. The conserved exon/intron structure and intron phases in the BdMAPK cascade kinase genes revealed the close evolutionary relationship among all four species, and supported their classification.

Furthermore, we have predicted the protein domains of the BdMAPK cascade kinase family using InterProScan against protein databases. The structure of all members of the BdMAPK cascade kinases was shown as a scheme in Figure 2C. Generally, each cluster of the BdMAPK cascade kinases by phylogenetic analysis shared a similar protein structure. All members of the BdMAPKs, BdMAPKKs and BdMAPKKKs contained a protein kinase domain (IPR011009). Most protein kinases catalyzed the transfer of a phosphate group from nucleoside triphosphates (often ATP) to specific amino acid residues of a protein substrate, resulting in a conformational change affecting protein function. The ATP-binding site, which is located on the N-terminal extremity of the catalytic domain, belongs to the most conserved sequences in the protein kinase family. Thus, most BdMAPKs, BdMAPKKs and BdMAPKKKs contained an ATP-binding site (IPR017441), which suggested that these BdMAPK cascade kinases used ATP as a ligand in signal transduction pathways. In the central part of the catalytic domain, almost all BdMAPK cascade kinases, except the group D in the BdMAPKs, contain the active site conserved aspartic acid residue (IPR008271), which is important for the catalytic activity of the enzyme. The catalytic domain and their structures in the BdMAPK cascade kinases are similar among proteins within subfamilies, demonstrating that the protein architecture is remarkably conserved within a specific subfamily. Moreover, despite all of BdMAPK cascade kinases contained a protein kinase domain, many of them contained other domains, such as ACT domain, PAS domain, and Ankyrin repeat containing domain. Interestingly, these BdMAPK cascade kinases which contained the same domain were clustered together to a clade, and showed similar expression patterns in response to multiple stresses treatment. For instances, most Phox/Bemlp domain containing BdMAPKKK genes showed to be down-regulated after PEG or H_2_O_2_ treatment, while the ACT domain containing BdMAPKKK genes were up-regulated by Cd^2+^. These results suggested that the BdMAPK cascade kinases exhibited different biological functions in response to various physiologic reactions. Because the biological functions of many MAPK cascade kinase genes in *B. distachyon* remain to be elucidated, the above findings may facilitate the identification of functional units in BdMAPK cascade kinase genes and lead to the discovery of their roles in plant growth and development.

### Genomic distribution and gene duplication of *BdMAPK*s, *BdMAPKKs* and *BdMAPKKKs*

**Figure 3:**
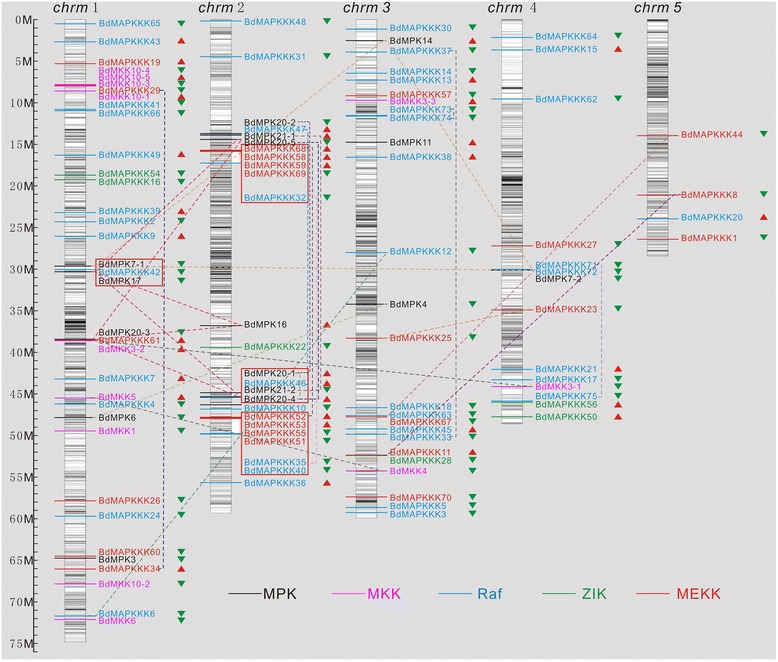
**Chromosomal locations and gene duplication for** ***B. distachyon MAPK*** **cascade kinase genes.** The chromosomal position of each *BdMAPK* cascade kinase gene was mapped according to the *B. distachyon* genome. The chromosome number is indicated at the top of each chromosome. The CpG island distribution maps shown in each chromosome depended on the CpG density in *B. distachyon* genome. The colored lines and characters represent which subgroup the proteins in each clade belong. The dotted line showed the gene duplication events among these *BdMAPK* cascade kinase genes. The scale bar was show on the left.

Although the genome of *B. distachyon* has already been sequenced, the information regarding genomic distribution and gene duplication of the *BdMAPK* cascade gene family in *B. distachyon* remains unclear. In order to investigate the relationship between genetic divergence and gene duplication within the *BdMAPK* cascade gene family in *B. distachyon*, we investigated the chromosomal locations of the *BdMAPK* cascade kinases genes based on the information from the *B. distachyon* genomic database (http://www.brachypodium.org/). The physical locations of the MAPK cascade kinase genes on *B. distachyon* chromosomes and the CpG island distribution map were depicted in Figure 3. We found that 103 BdMAPK cascade kinases were mapped on all 5 chromosomes of *B. distachyon*, in which 35 genes were present on chromosomes 1; 25 genes were located on chromosomes 2; and 25 genes were located on chromosomes 3. In addition, chromosome 4 had 14 MAPK cascade kinases, whereas chromosome 5 only encoded 4 MAPKKKs members. Interestingly, all members of the BdMAPK cascade kinase genes were located at the low density region of CpG islands on all 5 chromosomes. As shown in Figure 3, 26 pairs of duplication genes were identified, including 11 duplication events within the same chromosome and 15 segmental duplication events between chromosomes. This result suggested the duplication events could play vital roles in the expansion of the MAPK cascade kinase genes in the *B. distachyon* genome. Interestingly, among these paralogs, 8 gene pairs located at chromosome 2, 3 and 5 formed two pairs of duplicated chromosome regions, which were consistent with the result of duplicated regions of *BdWRKY* genes as reported previously [32]. These results revealed that members of the large gene family might be the consequence of genomic rearrangements and expansions during the process of evolution. Many DNA fragments from different origins could be recombined into another chromosome to form a new member of the large gene family. The function of the duplicated gene might be diverged by base substitutions, deletions and insertions.

### Comparative analysis of the phylogenetic ortholog genes of MAPKs, MAPKKs and MAPKKKs in *B. distachyon*, *A. thaliana* and *O. sativa*

To examine the evolutionary relationships between different MAPK cascade kinases in *B. distachyon*, *A. thaliana* and *O. sativa*, phylogenetic trees were constructed from alignments of the full MAPK cascade kinase amino acid sequences using the Neighbor-Joining (NJ) method by MEGA5.0 (Additional file 2). The gene model and amino acid sequences of MAPK cascade kinases in *B. distachyon*, *A. thaliana* and *O. sativa* were shown in Additional files 3 and 4. The phylogenetic analysis indicated that each of the BdMAPKs and BdMAPKKs can be divided into four subgroups in *A. thaliana* and *O. sativa*, which were consistent with the previous report [28], whereas MAPKKKs can be subdivided into three major subtypes, Raf-like family, MEKK-like family and ZIK-like family (Additional file 2). Moreover, Raf-like family contains more than half of MAPKKKs members in all three candidate plants. In general, all MAPK cascade kinases and their subgroups were present in monocots and dicots (Additional file 2), indicating that the occurrence of most components of the MAPK cascades in plants predates the monocot-dicot divergence and MAPK cascade kinase genes were conserved during evolution. Furthermore, MAPKKK phylogenetic tree showed similar clustering patterns in *O. sativa* and *B. distachyon*. In total, about 15 pairs of MAPKs, 8 pairs of MAPKKs and 60 pairs of MAPKKKs from *O. sativa* and *B. distachyon* were clustered as pairs, indicating that they might be the orthologous genes (Additional file 5). For instance, the amino acid sequence of BdMAPKKK29 and OsMAPKKK22 showed more than eighty percent of identities, indicating many consensuses in the MAPKKK protein sequences that may have existed before the species divergence between *B. distachyon* and *O. sativa*. The phylogenetic similarity found in *O. sativa* and *B. distachyon* suggested that they might have evolved conservatively. In contrast, *B. distachyon* has less orthologous genes than those in *O. sativa* compared to *A. thaliana* and a large number of MAPK cascade kinase genes were also clustered as pairs between *B. distachyon* and *A. thaliana*, suggesting that MAPK cascade kinase genes were large conserved gene families whose origin were very old (Additional file 5). The alignment of the conserved protein kinase domains showed that all Raf-like family in *B. distachyon* as well as *O. sativa* and *A. thaliana* shared Raf-like specific polypeptide signature GTxx (W/Y) MAPE, whereas ZIK-like subfamily contained a conserved polypeptide GTPEFMAPE (L/V/M)(Y/F) and MEKK-like members shared conserved polypeptide G (T/S) Px (W/F) MAPEV (Additional file 6).

**Figure 4:**
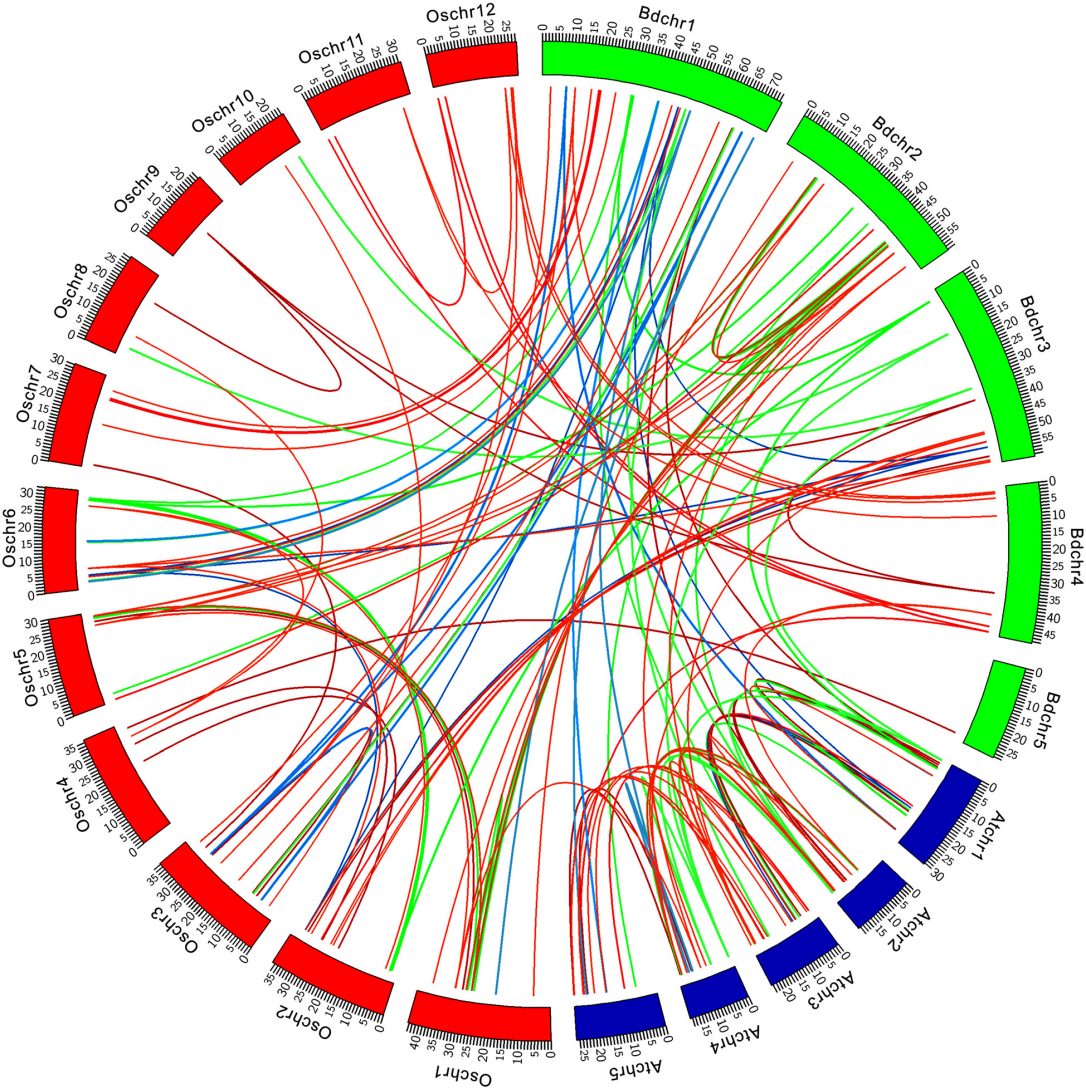
**Genomic map of** ***MAPK*** **cascade kinase genes synteny among** ***B. distachyon*** **,** ***O. sativa*** **and** ***A. thaliana*** **.** Schematic representation of 154 couples of duplicated genes was displayed on 12 *O. sativa* chromosomes, 5 *A. thaliana* chromosomes and 5 *B. distachyon* chromosomes by connecting lines using the CIRCOS software (Additional file 7). The size of chromosomes was consistent with the actual pseudo-chromosome size. Positions are in Mb.

Gene duplications play an important role in biological evolution [33-35]. With the technical development of DNA sequencing, the genomic sequence data can provide substantial evidence for the abundance of duplicated genes. In order to investigate the *MAPK* cascade kinase gene synteny among *B. distachyon*, *O. sativa* and *A. thaliana*, we searched the gene duplication within the *MAPK*, *MAPKK* and *MAPKKK* gene family in these genomes of three model plants based on the information from the Plant Genome Duplication Database (http://chibba.agtec.uga.edu/duplication/index/locus). These 154 couples of duplicated genes were displayed in Additional file 7. As shown in the chromatin topology of 22 chromosomes of all genomes of three model plants duplicated segments were distributed across all 22 chromosomes, except for the most central chromosome regions, which might be the centromeric region of the chromosome (Figure 4). Generally, the distribution of orthologs along the respective chromosomes of these model plant genomes was biased. Despite the duplication of orthologs occurred in the same species, most of the gene duplications were present between *B. distachyon* and *O. sativa* (Figure 4). For example, we have found 62 pairs of gene duplication between *B. distachyon* and *O. sativa*, however, only 21 pairs of gene duplication can be found between *B. distachyon* and *A. thaliana* (Figure 4). These results were consistent with the current understanding of the speciation process of the three species. Furthermore, we also found that the tightly linked genes at the bottom of *B. distachyon* Chromosome 2 were still tightly linked in the *O. sativa* Chromosome 1 with the identical orientation such as the *BdMAPKKK46/10/40* and *OsMAPKKK31/40/72*. A similar phenomenon could also be observed between the bottom of Chromosome 3 in *B. distachyon* and the bottom of Chromosome 2 in *O. sativa*. Interestingly, these highly similar regions were located at the low density region of CpG islands in both *B. distachyon* and *O. sativa* genome. Such instance could be explained as tightly linked sequences of genomic regions, which exhibited high expression, might be as donor regions in the evolution process of *B. distachyon* and *O. sativa*. The conservation of genome synteny between *B. distachyon* and *O. sativa* also confirmed that *B. distachyon* can be utilized as a temperate grass model species alternative to *O. sativa*. Moreover the evolutionary relationship between *O. sativa* and *A. thaliana* was remotest, which implied that *B. distachyon* might fall in between *O. sativa* and *A. thaliana* during evolutionary history.

### Expression pattern of the *BdMAPK*, *BdMAPKK* and *BdMAPKKK* genes in different tissues

**Figure 5:**
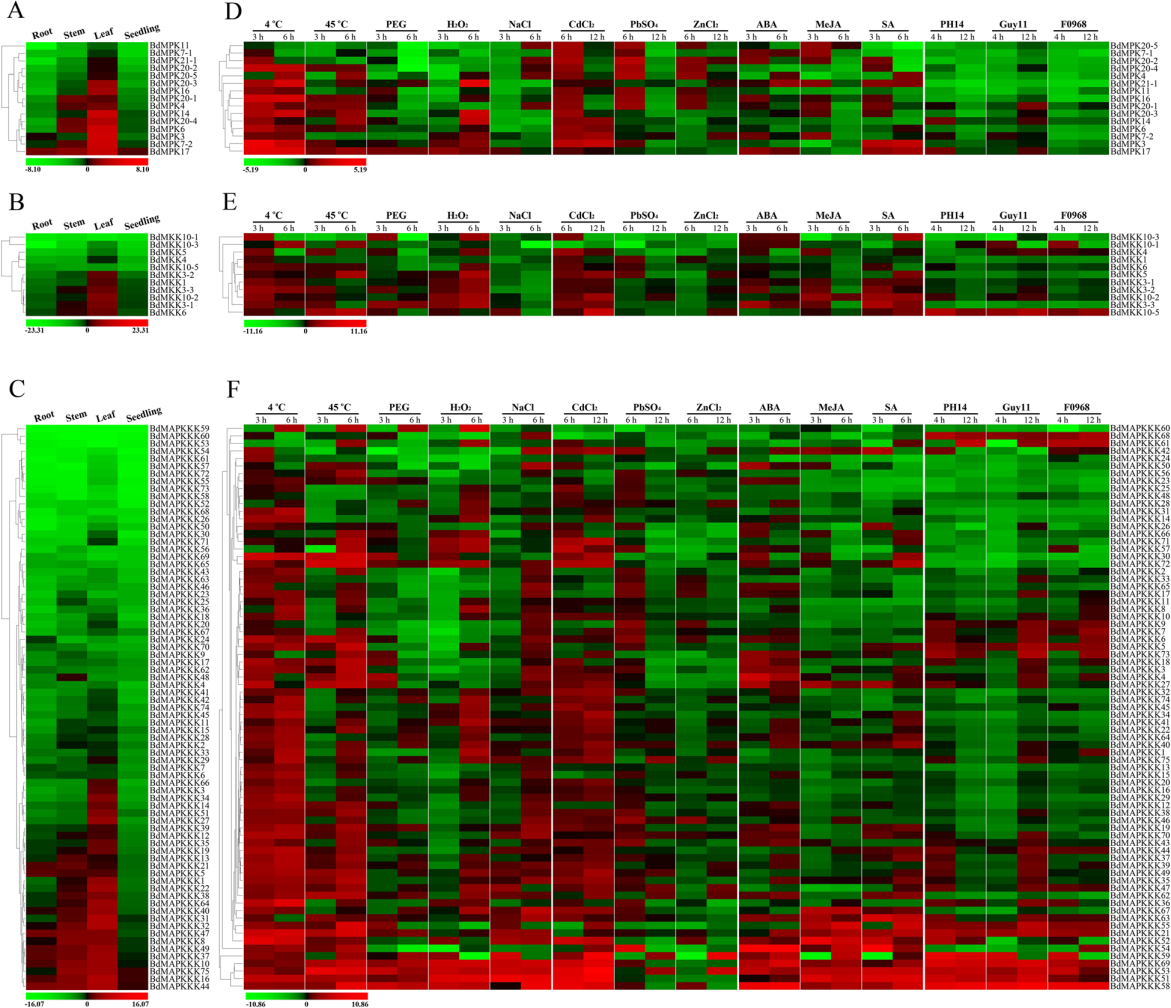
**Expression profiles of MAPK cascade kinase genes. (A-C)** Expression patterns of *MAPK*, *MAPKK* and *MAPKKK* genes in *B. distachyon* in different tissues. **(D-F)** Expression patterns of *MAPK*, *MAPKK* and *MAPKKK* genes upon multiple phytohormone treatments and abiotic or biotic stresses. The expression values of the *MAPK* cascade kinase genes were assessed upon the qPCR result analysis. The expression profile was shown by a green-red gradient using the PermutMatrix program.

It has been noted previously that different members of large gene families exhibit great disparities in abundance among different tissues to accommodate different physiological processes. Thus, gene expression patterns in different tissues can provide important clues for gene functions and gene co-regulations. To extract information about the relative abundance of transcripts of *B. distachyon* MAPK cascade kinases, we tested three different tissues that were a part of Bd21 seedling, leaves, stems and roots, to analyze the tissue specific expression patterns of all gene family members of MAPK cascade. The expression of all of the *BdMAPK*, *BdMAPKK* and *BdMAPKKK* gene family members was detected in all three tissues (Figure 5A-C). The results revealed that most of MAPK and MAPKK genes were highly expressed in Bd21 leaves, while the MAPKKK genes were much more abundantly expressed in the stems and leaves than that in the roots. As shown in Figure 5C, several *BdMAPKKK* genes, including *BdMAPKKK10*, −*16*, −*37*, −*44*, −*47* and −*49*, showed higher expression levels than other members of *MAPKKK* family in all of the three tissues tested. The expression of *BdMAPKKK3*, −*14*, −*27*, −*34* and −*51*, was highly expressed in the leaf while its expression level was relatively low in the root and stem. The expression pattern of these genes suggested that *BdMAPKKKs* were involved in the growth and development of organs or tissues under specific conditions. Interestingly, the *BdMKK6* exhibited a highly similar expression pattern with BdMPK6 in the three different tissues, which was consistent with the previous result that BdMKK6 exhibited a significant interaction with BdMPK6 [28]. The interacting partners, between BdMKK3-1 and BdMPK14, were also clustered together with similar expression patterns, while the expression levels of BdMPK7-1 in leaves and stems were lower than its interacting partner BdMKK3-1 [28]. These results suggested that the tissue specific expression patterns of all gene family members can be a possible precondition to presume possible co-regulatory genes or interacting partners.

### Differential expression profile of *BdMAPK*, *BdMAPKK* and *BdMAPKKK* gene upon multiple phytohormone treatments and abiotic or biotic stresses

In the following experiments, we demonstrated that MAPK cascades were not only involved in plant growth and development, but also played key roles in the control of plant response to multiple environmental stimuli including abiotic, biotic stresses and phytohormones. Firstly, the expression profiles of all gene members of the MAPK cascade kinases under different stress conditions were examined using qRT-PCR. A total of five abiotic stress types, i.e. heat, cold, NaCl, PEG and H2O2, which all can be activators of MAPK cascade pathway were tested in this study. Detailed expression profiles of the gene members of MAPK cascade kinases under different stress conditions were summarized in Additional file 8. Heat map representing the expression profiles of these gene members of MAPK cascade kinases under different stress conditions was shown in Figure 5D-F. Generally, our expression profile results were consistent with the Chen *et al.*, who found that most of the MAPKs and MAPKKs were induced or constitutively expressed under stresses treatment [28]. Similarly, our data revealed that 90% and 60% of MAPK cascade kinase genes were up-regulated under cold and heat stress conditions, respectively. Less MAPK cascade kinase genes were up-regulated under three other kinds of abiotic stress in this study, including NaCl, PEG and H2O2, which was also consist with Chen *et al.*, who found that only 43.75% of MAPK family genes were up-regulated in the PEG treatment [28].

These results indicated that the temperature alteration is the most sensitive stress perceived by plants. Furthermore, a large number of MAPK cascade kinase genes were unchanged or slightly down-regulated at 3 h after treatment with heat, H_2_O_2_ and NaCl, wheaeas the genes were up-regulated at 6 hrs after treatments. Over a half of MAPK cascade kinase genes were up-regulated in both heat and cold treatments. A few MAPK cascade kinase genes were up-regulated under the conditions of all five kinds of abiotic stresses, such as *BdMAPKKK51*, −*53*, −*58*, −*69* and so on. Most of the clustered gene pairs such as *BdMAPKK3-1/3-2, MAPKKK2/65, MAPKKK6/12*, and so on, showed the similar expression pattern after stress treatments, suggesting that these gene pairs might have similar physiological functions. On the other hand, several BdMAPKK gene pairs, which exhibited different expression patterns, may be involved in different signaling pathways. Furthermore, we also examined the expression profiles of all the gene members of MAPK cascade kinases to investigate whether the genes were involved in the response to heavy metal toxicity, including CdCl_2_, PbSO_4_ and ZnCl_2_ (Figure 5D-F). MAPK cascade kinase genes showed a very rapid increase in response to CdCl_2_ and PbSO_4_, while few MAPK cascade kinase genes were up-regulated by ZnCl_2_ treatment. In comparison with the abiotic stresses which have been discussed above, the expression level of *BdMAPK* and *BdMAPKK* genes *(BdMKK1, BdMPK7-1, BdMPK16, BdMPK20-1, etc.*) firstly increased within 6 hrs after heavy metal treatment and down-regulated at 12 hrs (Figure 5C and D). The same result was obtained in the expression pattern of most of *BdMAPKKK* genes which was in response to PbSO_4_ and ZnCl_2_ (Figure 5F). These results suggested that MAPK cascade kinases played a crucial role in response to heavy metal stress and the response was triggered within 6 hrs after stress treatment.

Recent studies of the MAPK cascades have also shown that the genes were responsive to JA, SA and ABA treatments. By treating plants with ABA, several components of MAPK signaling cascade genes showed a distinct inducible expression in many plant species, suggesting an important function of MAPK pathways in ABA signaling [36-39]. *OmMKK1* was increased progressively in response to increasing lengths of exposure of MeJA, SA, ethephon and MV [40]. To investigate the hormonal control mechanisms underlying MAPK cascade kinases, we treated Bd21 seedlings with three phytohormones, MeJA, SA and ABA, respectively and analyzed the changes of the transcription abundance of these MAPK cascade kinase genes using qRT-PCR. Our results demonstrated that 40% of MAPK cascade kinase genes were up-regulated by these three phytohormones after 3 hrs of treatment, respectively (Figure 5D-F). Only three of MAPK and MAPKK genes were up-regulated by all three phytohormones, whereas more than ten *MAPKKKs* were induced by MeJA, SA and ABA. It has reported that ABA as a phytohormone played an important role in integrating various abiotic stress signals and controlling downstream stress responses [41]. Our data indicated that most *BdMAPKKKs* were showed a similar expression pattern under drough (PEG) and H_2_O_2_ stress conditions compared with ABA treatment, except for *BdMAPKKK3*, −*4*, −*18*, −*27* and −*73*. These correlations of *BdMAPKKKs* expression levels between abiotic stress and phytohormone treatment suggested that the stress induced MAPK cascade signal transduction might be linked to the stress induced phytohormone alteration.

It has been reported that pathogen-associated molecular patterns (PAMPs)-triggered immunity requires a signal transduction from receptors to downstream components via the MAPK cascade, suggesting that plant MAPK cascades play a key role in the induction of defense mechanisms [11,15,42]. For example, the *A. thaliana fls2* mutant was more susceptible than the wild type (WT) to infection by the virulent pathogen DC3000, and the WT showed an enhanced resistance to DC3000 after flg22 treatment [43]. To investigate the mechanisms of MAPK cascades in disease defense, we determined the expression profiles of MAPK cascade kinase genes in *B. distachyon* after phytopathogen treatments. A total of three phytopathogens, including *Fusarium graminearum* (F0968) and two strains of *Magnaporthe grisea* (Guy11, avirulent ACE1 genotype; PH14, virulent ACE1 genotype) were used to inoculate Bd21 seedling in this study. The expression profiles of the MAPK cascade kinase genes at 4 hpi (hour post-inoculation) and 12 hpi were shown in Figure 5D-F. Only a few members of BdMAPK and BdMAPKK genes were up-regulated by phytopathogen treatment, whereas approximately 40% of BdMAPKKK genes were phytopathogen-induced. Interestingly, a large number of BdMAPKKK genes were induced faster by PH14 than by Guy11. For example, *BdMAPKKK39*, −*47*, −*49* and −*63* were up-regulated at 4 hpi after infection by PH14, but at 12 hpi, they were down regulated by PH14 and up-regulated by Guy11. These results were consistent with the previous report about the expression pattern of *BdWRKY* genes, suggesting that BdMAPKKK genes might play an important role with *BdWRKY*s in plant defense [32]. The relationship between MAPK cascade kinases and WRKY transcript factors in phytopathogen-induced plant disease defense in *B. distachyon* should be further investigated.

### Regulatory network of MAPK cascade kinase genes

**Figure 6:**
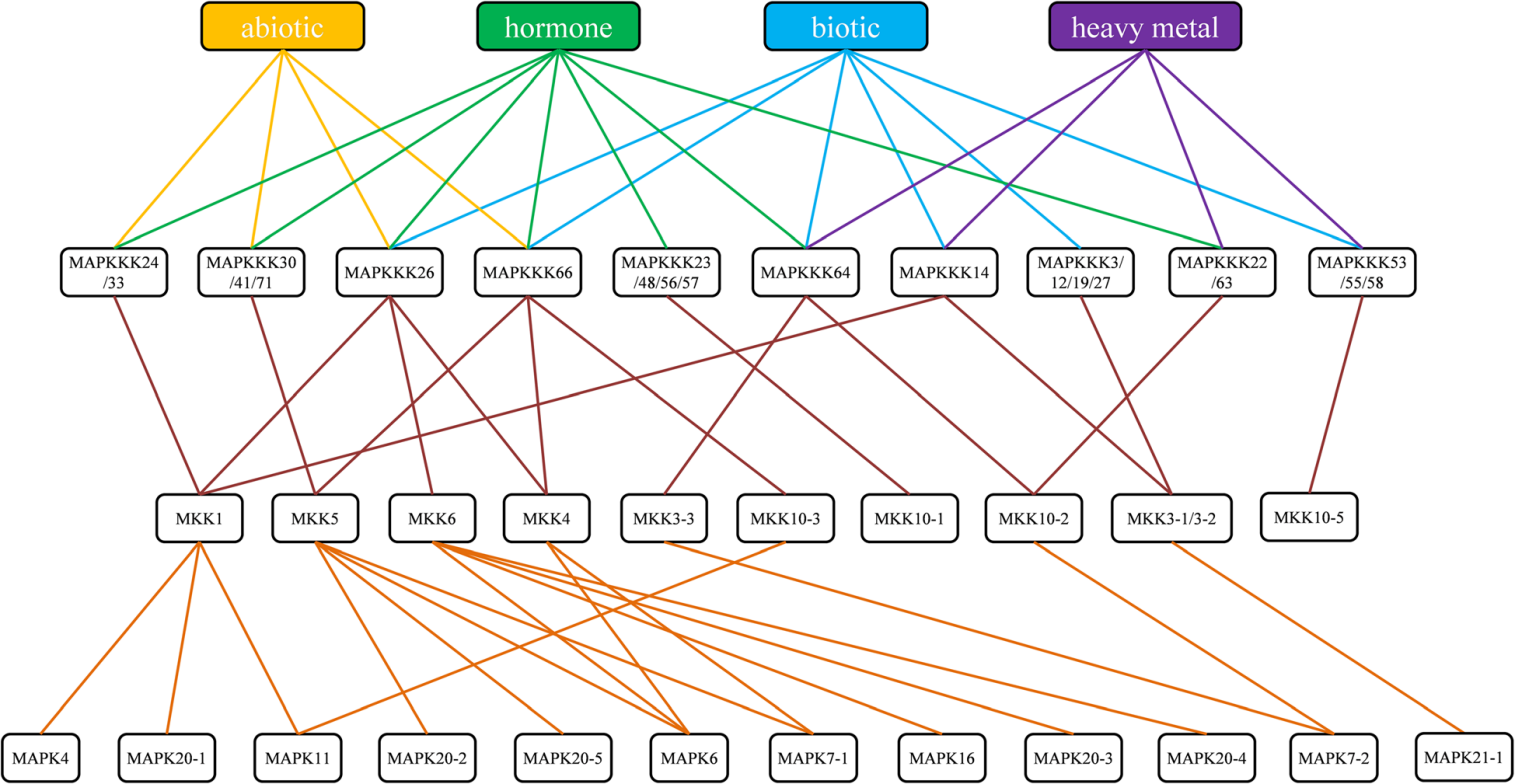
**Regulatory networks of** ***MAPK*** **cascade kinase genes.** Co-expression regulatory network of *MAPK* cascade kinase genes in *B. distachyon*. Connections based on the co-expression regulatory *MAPK* cascade kinase genes with the correlation of expression patterns between *MAPK* cascade kinase genes > 0.5, under different kind of treatment conditions, including biotic, abiotic, and heavy metal stress and hormone treatment, respectively.

In general, MAPK cascade kinase genes formed conserved signaling modules, which contained three functionally linked protein kinases: MAPKs, MAPKKs and MAPKKKs [2,3]. In this study, we constructed the regulatory network of MAPK cascade kinases upon different stress treatments in *B. distachyon*. To investigate clusters of co-expressed MAPK cascade kinase genes in *B. distachyon*, the expression data of *BdMAPK*s, *BdMAPKK*s and *BdMAPKKK*s were clustered together to form integrated expression profiles. In order to investigate whether the expression profiles of MAPK cascade kinase genes in a set of MAPK cascades were correlated, we compared their tissue-specific expression patterns and changes of the expression levels under different treatment conditions, including biotic stress, abiotic heavy metal stress and hormone treatment, respectively. The results showed several groups of co-expression regulatory MAPK cascade kinases whose correlation coefficients were greater than 0.5, according to the clustered results of the integrated expression profiles (Additional file 9). The integrated expression profiles indicated that specific clusters of co-expressed MAPK cascade kinase genes were involved in response to the stresses. A scheme representing the co-expression regulatory network was constructed according to the data of co-expression regulatory MAPK cascade kinase genes (Figure 6). In *A. thaliana*, modules including the MAPKs MPK3/MPK6 and MAPKKs MKK1/MKK4/MKK5/MKK9 were clearly proved to be involved in multiple stress-response strategies [11]. Consistently, those orthologous genes in *B. distachyon*, such as MAPKs BdMPK6/BdMPK7 and MAPKKs BdMKK1/BdMKK4/BdMKK5/BdMKK6, were also found to be involved in several different types of stress treatments (Figure 6). From the scheme in Figure 6, the comparison results indicated that most of duplicated paralog pairs at the terminal nodes of the phylogenetic tree may have maintained their ancestral function. For example, two duplicated genes of BdMKK3-1/BdMKK3-2 in *B. distachyon* that were clustered together in the phylogenetic tree were constitutively expressed in all the tissues. These two genes were also clustered together in tissue specific expression profile. Therefore, these two duplicated genes could be classified together in the co-expression regulatory network. As shown in Figure 6, expression level changes of BdMKK6 and BdMPK6 were clustered together, in which two proteins have been proved to exhibit significant interaction with each other [28]. Moreover, some duplicated paralogous genes showed divergent function in the evolutionary process, even though the reasons for expression divergence of the duplicated genes in many gene families remain unclear. It has been shown that BdMPKK3-1 exhibited a significant interaction with BdMPK7-1. However, the changes of the expression level between BdMPKK3-1 and BdMPK7-1 couldn’t be clustered together, while BdMPKK3-1 showed a similar expression pattern with BdMPK7-2, a homolog of BdMPK7-1. These results indicated that the scheme of the regulatory network could provide clues for a possible signaling pathway of BdMAPK cascade kinases, which show the same function or interaction in response to corresponding stress treatment. In addition, by comparing the regulatory network of MAPK cascade kinase orthologous genes in *A. thaliana*, our network showed a reliable result in BdMAPK signaling pathway prediction.

## Conclusions

The identification and characterization of all MAPK cascade kinase genes in a grass model plant *B distachyon* would facilitate a better understanding of the evolutionary processes and functions of these gene families. First, this study has completed identification of MAPKKK genes, the biggest upstream kinase gene family of the MAPK cascade in *B. distachyon*. Subsequently, this study has done systematic molecular evolutionary analysis of all MAPK, MAPKK and MAPKKK genes as a whole in *B. distachyon* comparing to *O. sativa* and *A. thaliana*. Furthermore, most importantly, this study has established a MAPK signaling co-regulation network to investigate the multiple-stress-driven interactions between BdMAPKs, BdMAPKKs and BdMAPKKKs upon different stresses treatment. Base on the regulation network, we found that most of duplicated BdMAPK cascade kinase gene orthologs showed their convergent function, whereas few of them developed divergent function in the evolutionary process. The molecular evolutionary analysis of identified MAPK family genes and the constructed MAPK cascade regulation network under multiple stresses provided useful information for further investigation of the functions of BdMAPK cascade kinase genes across different plant species.

## Methods

### Sequence retrieval

To identify *B. distachyon* genes encoding MAPKKK proteins with a kinase domain, we performed a BLASTP search among 32255 sequences of the protein database of *B. distachyon* from MIPS (http://mips.helmholtz-muenchen.de/plant/brachypodium/) using 155 query *MAPKKK* sequences from *A. thaliana* and *O. sativa* [1]. A self BLAST of these sequences followed by manual editing to remove the redundancy finally resulted in the identification of 75 *MAPKKK* genes. To verify the reliability of our results, all putative non-redundant sequences were assessed with UniProt (http://www.uniprot.org/) and SMART (http://smart.embl-heidelberg.de/) analysis, respectively. A total of 75 *BdMAPKKK* genes were found in *B. distachyon* (Additional file 1). All of these 75 putative *MAPKKK* gene family members in *B. distachyon* genome were designed as *BdMAPKKK1*-*BdMAPKKK75* base on the BBMH scores between putative *BdMAPKKK* genes with *MAPKKK* gene family in *O. sativa*. The annotation of 16 *MAPK* genes and 12 *MAPKK* genes from *B. distachyon* Bd21 genome in Additional file 1 were made according to the previous research [28].

### Sequence and phylogenetic analysis

To analyze the sequence of the 75 typical identified BdMAPKKKs, 12 MAPKKs and 16 MAPKs, we performed multiple alignment analyses of the kinase domains, sequence by ClustalW (www.ebi.ac.uk/clustalw/). A neighbor-joining (NJ) tree was constructed using the MEGA version 5 software, based on the alignment of MAPK cascade nucleotide or amino acid sequences in *O. sativa*, *A. thaliana* and *B. distachyon*. To determine the statistical reliability, we conducted bootstrap analysis with the following parameters: p-distance and pairwise deletion. Bootstrap analysis was performed with 1000 replicates. The data of the phylogenetic tree were deposited in Treebase Web (Accession URL: http://www.psort.org/).

### Gene structure analysis

The information of *BdMAPK* cascade kinase genes, including accession number, chromosomal location, ORF length and exon-intron structure, were retrieved from the *B. distachyon* genome Database (http://www.brachypodium.org/). As well as the gene struc-tures of the BdMAPK cascade kinases were generated with the GSDS version 2.0 (Gene Structure Display Server 2: http://gsds.cbi.pku.edu.cn/).

### Protein analysis of BdMAPK cascade kinases

The conserved domains and motifs in the MAPK cascade kinases was predicted using InterProScan against protein databases (http://www.ebi.ac.uk/interpro/). The schematic representing the structure of all members of BdMAPK cascade kinases was based on the InterProScan analysis results. Subcellular localization predictions of each of the BdMAPK cascade kinases were carried out using WoLF PSORT server (http://purl.org/phylo/treebase/phylows/study/TB2:S17106?x-accesscode=37158a103e4acaea162c89867a5c7061&format=html). The theoretical pI (isoelectric point) and Mw (molecular weight) of BdMAPK cascade kinases were carried out using Compute pI/Mw tool online (http://web.expasy.org/compute_pi/). Subcellular localization predictions of each of the BdMAPK cascade kinases were carried out using WoLF PSORT server (http://wolfpsort.seq.cbrc.jp/).

### *MAPKKK* gene synteny analysis

The gene duplications within the *MAPK* cascade kinase gene family in *B. distachyon*, *O. sativa* and *A. thaliana* genomes were based on the information from the Plant Genome Duplication Database (http://chibba.agtec.uga.edu/duplication/index/locus). In order to visualize duplicated regions in the *B. distachyon*, *O. sativa* and *A. thaliana* genome, lines were drawn between matching genes using Circos-0.64 program (http://circos.ca/) [44].

### Cluster analysis of expression data

The 2-week old seedlings (Bd21) were used for harvesting leaf, stem and root samples. The protocol of abiotic stresses treatment for Bd21 was adopted according with the previous work with some modifies [28]. For phytohormone analysis, 2-week-old seedlings were treated in MS liquid medium containing 100 μM MeJA, 100 μM ABA, 1 mM SA and 20 μM 6-BA for 3 h or 6 h, respectively. For abiotic stress treatment, 2-week-old seedlings were treated in MS liquid medium containing 20% PEG, 200 mM NaCl and 10 mM H_2_O_2_ for 3 h or 6 h, respectively. For heavy metal stress treatment, 2-week-old seedlings were treated in MS liquid medium containing 100 μM ZnCl_2_, 100 μM PbSO_4_ and 100 μM CdCl_2_ for 6 h or 12 h, respectively. Cold and heat treatments were achieved by placing 2-week-old seedlings in MS liquid medium at 4°C or 45°C for 3 h or 6 h, respectively. For phytopathogen treatment, 2-week-old seedlings were sprayed with *Fusarium graminearum* (F0968) and two strains of *Magnaporthe grisea* (Guy11, avirulent ACE1 genotype; PH14, virulent ACE1 genotype) for 4 h or 12 h. The samples of each treatment were collected three biological replications. The *BdMAPK* cascade kinase genes array constituted of 103 primer-sets representing all members of the *B. distachyon MAPK* cascade kinase gene family. The primer-sets were listed in Additional file 10. The expression of the 103 *BdMAPK* cascade kinase genes was assessed upon the qPCR result analysis. Each qPCR experiment was repeated three separate times. The expression profile was calculated from the –ΔΔ*C*T value [−ΔΔ*C*T = (*C*Tcontrol.gene – *C*Tcontrol.actin) – (*C*Ttreat.gene – *C*Ttreat.actin)], obtained by PermutMatrixEN version 1.9.3 software, and shown by a green-red gradient. The data were statistically analyzed using OriginPro 7.5 software. The up-regulated genes were defined as a fold-change greater than 2 with *p-value* < 0.05 and a fold change of 0.5 or less was used to define down-regulated genes when the *p-value* < 0.05. All qPCR data were submitted to NCBI GEO dataset. The accession number is GSE66497.

### Regulatory network construction

The expression data of *BdMAPK*, *BdMAPKK* and *BdMAPKKK* were clustered together to form an integrated expression profile by Cluster 3.0 software and visualized by using TreeView software. The *MAPK* cascade kinase genes, whose correlation coefficients of expression profiles were greater than 0.5, were clustered together as a set of co-expression regulatory *MAPK* cascade kinase genes under different kinds of treatment conditions, including biotic, abiotic, heavy metal stress and hormone treatment, respectively (Additional file 9). The line drawing which represented the co-expression regulatory network was constructed according to the data of co-expression regulatory *MAPK* cascade kinase genes.

## Additional files

Additional file 1:
**List of MAPK cascade kinase genes in** ***B. distachyon.***
Additional file 2:
**Neighbor-joining analyses of MAPK (A), MAPKK (B) and MAPKKK (C) amino acid sequences from** ***O. sativa,*** ***A. thaliana*** **and** ***B. distachyon.***
Additional file 3:
**List of MAPK cascade kinase genes in** ***O. sativa,*** ***A. thaliana*** **and** ***B. distachyon*** **chromosomes.**
Additional file 4:
**The amino acid sequences of MAPKKK members in** ***B. distachyon***, ***A. thaliana*** **and** ***O. sativa.***
Additional file 5:
**Orthologs of MAPK cascade kinase genes among** ***B. distachyon, A. thaliana and O. sativa.***
Additional file 6:
**Alignment of MAPKKKs from** ***O. sativa,*** ***A. thaliana*** **and** ***B. distachyon.***
Additional file 7:
**The duplicated genes in** ***O. sativa,*** ***A. thaliana*** **and** ***B. distachyon*** **chromosomes.**
Additional file 8:
**Expression data of the** ***BdMAPK*** **cascade kinase genes after stresses and phytohormone treatment.**
Additional file 9:
**The co-expression regulatory MAPK cascade kinase genes.**
Additional file 10:
**The list of qRT-PCR primers of MAPK cascade kinase genes.**
