# Supplementary material for: Genome-wide exploration of the molecular evolution and regulatory network of mitogen-activated protein kinase cascades upon multiple stresses in Brachypodium distachyon
Source: BMC Genomics. 2015 Mar 24;16(1):228. doi: 10.1186/s12864-015-1452-1 (PMC4404688; doi:10.1186/s12864-015-1452-1)
Supplement: Additional file 1: — List of MAPK cascade kinase genes in B. distachyon. [file 12864_2015_1452_MOESM1_ESM.pdf]

Additional file 1. List of MAPK cascade kinase genes in *B. distachyon*

Gene	Score	Position	Gene model	Group	Size (aa)	Subcellular localization *	pI	MW(kD)
BdMPK3	672	Bd1:64739946-64742370	Bradi1g65810	A	369	cyto	5.46	42.87
BdMPK4	719	Bd3:34209148-34213422	Bradi3g32000	B	375	cysk	5.75	42.76
BdMPK6	724	Bd1:47836748-47843578	Bradi1g49100	A	391	cysk	5.5	44.20
BdMPK7-1	688	Bd1:29632402-29634667	Bradi1g34030	C	413	chlo	8.46	47.11
BdMPK7-2	N/A	Bd4:30118480-30120192	Bradi4g24912	C	369	cyto	6.63	42.37
BdMPK11	714	Bd3:14742468-14745425	Bradi3g16560	B	387	chlo	6.24	43.96
BdMPK14	N/A	Bd3:2521821-2525826	Bradi3g03780	C	369	cyto	6.5	42.32
BdMPK16	972	Bd2:36786589-36792652	Bradi2g36470	D	544	cyto	8.77	61.60
BdMPK17	951	Bd1:30307750-30312635	Bradi1g34700	D	581	cyto	6.81	65.49
BdMPK20-1	1070	Bd2:44852084-44858179	Bradi2g44350	D	615	nucl	9.1	69.95
BdMPK20-2	N/A	Bd2:13689255-13693771	Bradi2g15317	D	597	cysk	9.14	66.75
BdMPK20-3	727	Bd1:38419711-38433437	Bradi1g41780	D	421	cyto	8.72	48.62
BdMPK20-4	998	Bd2:46302880-46308387	Bradi2g45870	D	580	cyto	9.38	66.39
BdMPK20-5	N/A	Bd2:14412074-14417497	Bradi2g16337	D	581	cysk	9.33	66.41
BdMPK21-1	904	Bd2:13875717-13880863	Bradi2g15620	D	595	chlo	6.62	67.14
BdMPK21-2	832	Bd2:45394037-45403577	Bradi2g45010	D	577	cyto	9.04	66.41
BdMKK1	617	Bd1:49443017-49446304	Bradi1g51000	A	348	nucl	5.46	38.65
BdMKK3-1	N/A	Bd4:44107411-44111847	Bradi4g39490	B	523	cyto	5.7	58.46
BdMKK3-2	979	Bd1:38580168-38590500	Bradi1g41860	B	523	cyto	5.66	58.60
BdMKK3-3	N/A	Bd3:9689290-9697559	Bradi3g11260	B	567	cyto	5.74	63.33
BdMKK4	558	Bd3:54258852-54260320	Bradi3g53650	D	357	chlo	9.47	38.43
BdMKK5	536	Bd1:45433080-45434736	Bradi1g46880	D	343	mito	9.21	36.99
BdMKK6	660	Bd1:72118481-72123498	Bradi1g75150	A	356	cyto	5.52	39.96
BdMKK10-1	408	Bd1:8573765-8574808	Bradi1g11525	C	347	chlo	7.65	36.88
BdMKK10-2	391	Bd1:67832493-67833815	Bradi1g69400	C	340	mito	6.56	36.09
BdMKK10-3	390	Bd1:7865287-7866321	Bradi1g10800	C	344	chlo	8.39	36.52
BdMKK10-4	N/A	Bd1:7809375-7810400	Bradi1g10770	C	341	cyto	9.07	35.92
BdMKK10-5	N/A	Bd1:7835644-7836642	Bradi1g10790	C	332	chlo	9.29	34.73
BdMAPKKK1	2402	Bd5:26381665-26393219	Bradi5g24870	MEKK	1346	nucl	5.93	148.92
BdMAPKKK2	1875	Bd1:24311845-24322003	Bradi1g28950	Raf	1221	nucl	5.48	134.33
BdMAPKKK3	1801	Bd3:59221921-59227497	Bradi3g60210	Raf	1116	nucl	5.48	122.77
BdMAPKKK4	1801	Bd1:46211026-46216334	Bradi1g47570	Raf	1334	nucl	5.75	142.24
BdMAPKKK5	1631	Bd3:58646176-58654454	Bradi3g59510	Raf	1115	nucl	5.2	122.69
BdMAPKKK6	1588	Bd1:71704725-71713297	Bradi1g74480	Raf	1002	chlo	6.22	110.03
BdMAPKKK7	1559	Bd1:43232910-43245317	Bradi1g45040	Raf	1074	chlo	5.51	117.63
BdMAPKKK8	1538	Bd5:21229500-21236337	Bradi5g18180	MEKK	897	nucl	9.7	97.55
BdMAPKKK9	1526	Bd1:26089105-26094508	Bradi1g30720	Raf	1107	nucl	5.53	118.69
BdMAPKKK10	1461	Bd2:46786767-46795821	Bradi2g46340	Raf	803	nucl	5.9	89.17
BdMAPKKK11	1434	Bd3:52432077-52442048	Bradi3g51380	MEKK	892	nucl	9.62	96.71
BdMAPKKK12	1407	Bd3:27996347-28004441	Bradi3g27120	Raf	969	nucl	5.84	107.09
BdMAPKKK13	1394	Bd3:7283630-7297067	Bradi3g09170	Raf	793	nucl	6.24	88.33

BdMAPKKK14	1223	Bd3:6429088-6437874	Bradi3g08260	Raf	854	chlo	5.45	93.21
BdMAPKKK15	1207	Bd4:3687248-3696919	Bradi4g04470	Raf	759	nucl	7.26	83.82
BdMAPKKK16	1179	Bd1:19257221-19260591	Bradi1g23970	ZIK	681	nucl	5.51	76.87
BdMAPKKK17	1166	Bd4:43347286-43354339	Bradi4g38400	Raf	768	chlo	6.46	83.65
BdMAPKKK18	1122	Bd3:46685117-46693548	Bradi3g44710	Raf	736	chlo	7.61	80.21
BdMAPKKK19	1110	Bd1:5382770-5391852	Bradi1g07650	MEKK	760	pero	9.32	82.7
BdMAPKKK20	1095	Bd5:23982964-23988026	Bradi5g21330	Raf	763	chlo	6.92	83.41
BdMAPKKK21	1082	Bd4:42043874-42051570	Bradi4g36880	Raf	593	cyto	5.7	65.65
BdMAPKKK22	1062	Bd2:39411876-39418219	Bradi2g39350	ZIK	679	nucl	5.09	76.16
BdMAPKKK23	1040	Bd4:34951319-34957350	Bradi4g29500	MEKK	685	nucl	6.06	74.62
BdMAPKKK24	1026	Bd1:59671548-59677424	Bradi1g60340	Raf	827	nucl	8.66	91.38
BdMAPKKK25	986	Bd3:38320000-38325924	Bradi3g36080	MEKK	681	nucl	6.01	74.81
BdMAPKKK26	961	Bd1:57883897-57890503	Bradi1g58810	MEKK	730	chlo	9.3	79.38
BdMAPKKK27	921	Bd4:27230487-27234555	Bradi4g22760	MEKK	664	nucl	9.53	71.88
BdMAPKKK28	920	Bd3:52493768-52498074	Bradi3g51460	ZIK	616	chlo	5.02	68.33
BdMAPKKK29	919	Bd1:7973279-7979810	Bradi1g10970	MEKK	647	chlo	5.19	69.75
BdMAPKKK30	902	Bd3:1128750-1132858	Bradi3g01850	Raf	601	cyto	6.86	66.83
BdMAPKKK31	890	Bd2:4698348-4702360	Bradi2g06260	Raf	562	nucl	9.36	63.39
BdMAPKKK32	858	Bd2:17241445-17245595	Bradi2g19590	Raf	596	nucl	8.49	66.37
BdMAPKKK33	821	Bd3:49832148-49837407	Bradi3g48360	Raf	484	chlo	8.3	53.93
BdMAPKKK34	820	Bd1:66064380-66071523	Bradi1g67400	MEKK	757	nucl	7.17	82.5
BdMAPKKK35	820	Bd2:49740523-49744032	Bradi2g49700	Raf	595	nucl	8.94	66.32
BdMAPKKK36	805	Bd2:55622119-55624811	Bradi2g57470	Raf	494	chlo	9.01	55.23
BdMAPKKK37	789	Bd3:3901063-3904136	Bradi3g05520	Raf	424	cyto	7.21	46.99
BdMAPKKK38	788	Bd3:16597394-16605813	Bradi3g18150	Raf	418	cyto	8.39	45.99
BdMAPKKK39	779	Bd1:23247985-23260462	Bradi1g28110	Raf	582	chlo	5.93	64.65
BdMAPKKK40	747	Bd2:49806289-49809892	Bradi2g49790	Raf	451	cysk	6.05	50.68
BdMAPKKK41	745	Bd1:10874042-10878698	Bradi1g14000	Raf	377	cyto	8.51	41.42
BdMAPKKK42	716	Bd1:30837694-30839002	Bradi1g35350	Raf	405	chlo	9.26	45.24
BdMAPKKK43	697	Bd1:2776045-2779519	Bradi1g04080	Raf	384	cyto	8.07	42.45
BdMAPKKK44	696	Bd5:13989179-13995141	Bradi5g10670	MEKK	697	nucl	9.19	75.55
BdMAPKKK45	685	Bd3:49203215-49208033	Bradi3g47600	Raf	351	cyto/ER	6.9	39.41
BdMAPKKK46	681	Bd2:45294740-45299227	Bradi2g44910	Raf	388	chlo	7.59	42.91
BdMAPKKK47	679	Bd2:13839453-13843007	Bradi2g15560	Raf	380	nucl	7.96	42.11
BdMAPKKK48	622	Bd2:394744-398444	Bradi2g00670	Raf	371	nucl	9.19	41.06
BdMAPKKK49	621	Bd1:16330864-16335462	Bradi1g20390	Raf	443	cyto	7.08	49.1
BdMAPKKK50	612	Bd4:47836156-47839912	Bradi4g44430	ZIK	674	nucl	5.94	73.23
BdMAPKKK51	577	Bd2:47911559-47913163	Bradi2g47510	MEKK	485	mito	5.15	52.34
BdMAPKKK52	558	Bd2:47895034-47896500	Bradi2g47480	MEKK	489	cyto	4.77	52.09
BdMAPKKK53	541	Bd2:47901623-47903160	Bradi2g47490	MEKK	482	chlo	4.58	51.38
BdMAPKKK54	538	Bd1:18706049-18708119	Bradi1g23320	ZIK	291	cyto	5.95	32.71
BdMAPKKK55	533	Bd2:47907123-47908569	Bradi2g47500	MEKK	461	chlo	4.91	48.52
BdMAPKKK56	520	Bd4:45996792-45999204	Bradi4g41940	ZIK	367	cyto	6.41	41.13
BdMAPKKK57	471	Bd3:9151086-9154557	Bradi3g10890	MEKK	514	chlo	5.41	53.75

BdMAPKKK58	442	Bd2:15856972-15858475	Bradi2g17820	MEKK	476	chlo	4.52	50.3
BdMAPKKK59	437	Bd2:15863628-15865277	Bradi2g17830	MEKK	550	cyto	4.44	57.92
BdMAPKKK60	375	Bd1:64476929-64478355	Bradi1g65500	MEKK	308	chlo	10.4	32.29
BdMAPKKK61	N/A	Bd1:38555071-38556771	Bradi1g41850	MEKK	567	chlo	6.28	60.54
BdMAPKKK62	N/A	Bd4:9550465-9557023	Bradi4g09990	Raf	742	nucl	7.59	82.36
BdMAPKKK63	N/A	Bd3:47663323-47669443	Bradi3g45660	Raf	731	nucl	6.85	81.78
BdMAPKKK64	N/A	Bd4:2137135-2141974	Bradi4g02900	Raf	1077	nucl	5.21	118.49
BdMAPKKK65	N/A	Bd1:463748-468001	Bradi1g00580	Raf	1110	nucl	5.1	121.6
BdMAPKKK66	N/A	Bd1:10919176-10923468	Bradi1g14010	Raf	377	cyto	8.15	41.44
BdMAPKKK67	N/A	Bd3:47768461-47774400	Bradi3g45790	MEKK	689	mito	9.08	75.14
BdMAPKKK68	N/A	Bd2:15848962-15851260	Bradi2g17800	MEKK	519	chlo	4.82	54.34
BdMAPKKK69	N/A	Bd2:15868375-15869700	Bradi2g17840	MEKK	442	chlo	4.74	46.62
BdMAPKKK70	N/A	Bd3:57407383-57412051	Bradi3g57740	MEKK	529	chlo	6.89	57.81
BdMAPKKK71	N/A	Bd4:30029484-30036316	Bradi4g24830	Raf	516	cyto	5.34	57.59
BdMAPKKK72	N/A	Bd4:30037627-30043618	Bradi4g24840	Raf	604	nucl	6.06	67.11
BdMAPKKK73	N/A	Bd3:11660021-11665140	Bradi3g13050	Raf	509	nucl	5.12	57.58
BdMAPKKK74	N/A	Bd3:11666314-11671851	Bradi3g13060	Raf	555	nucl	6.41	62.56
BdMAPKKK75	N/A	Bd4:45819310-45827277	Bradi4g41870	Raf	539	cysk	4.91	58.74

* chlo: Chloroplast; cyto: Cytoplasm; cysk: Cytoskeleton; mito: Mitochondria; nucl: Nucleus; pero: Peroxisome;
