# Supplementary figures and images for: Genome-wide exploration of the molecular evolution and regulatory network of mitogen-activated protein kinase cascades upon multiple stresses in Brachypodium distachyon
Source: BMC Genomics. 2015 Mar 24;16(1):228. doi: 10.1186/s12864-015-1452-1 (PMC4404688; doi:10.1186/s12864-015-1452-1)

A

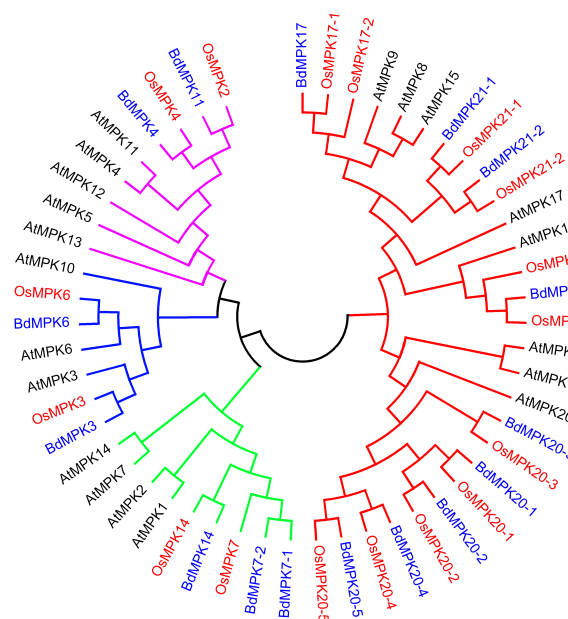

B

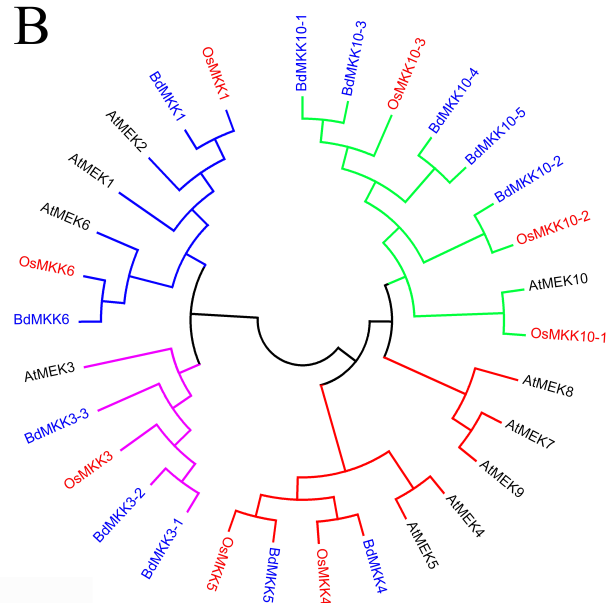

C

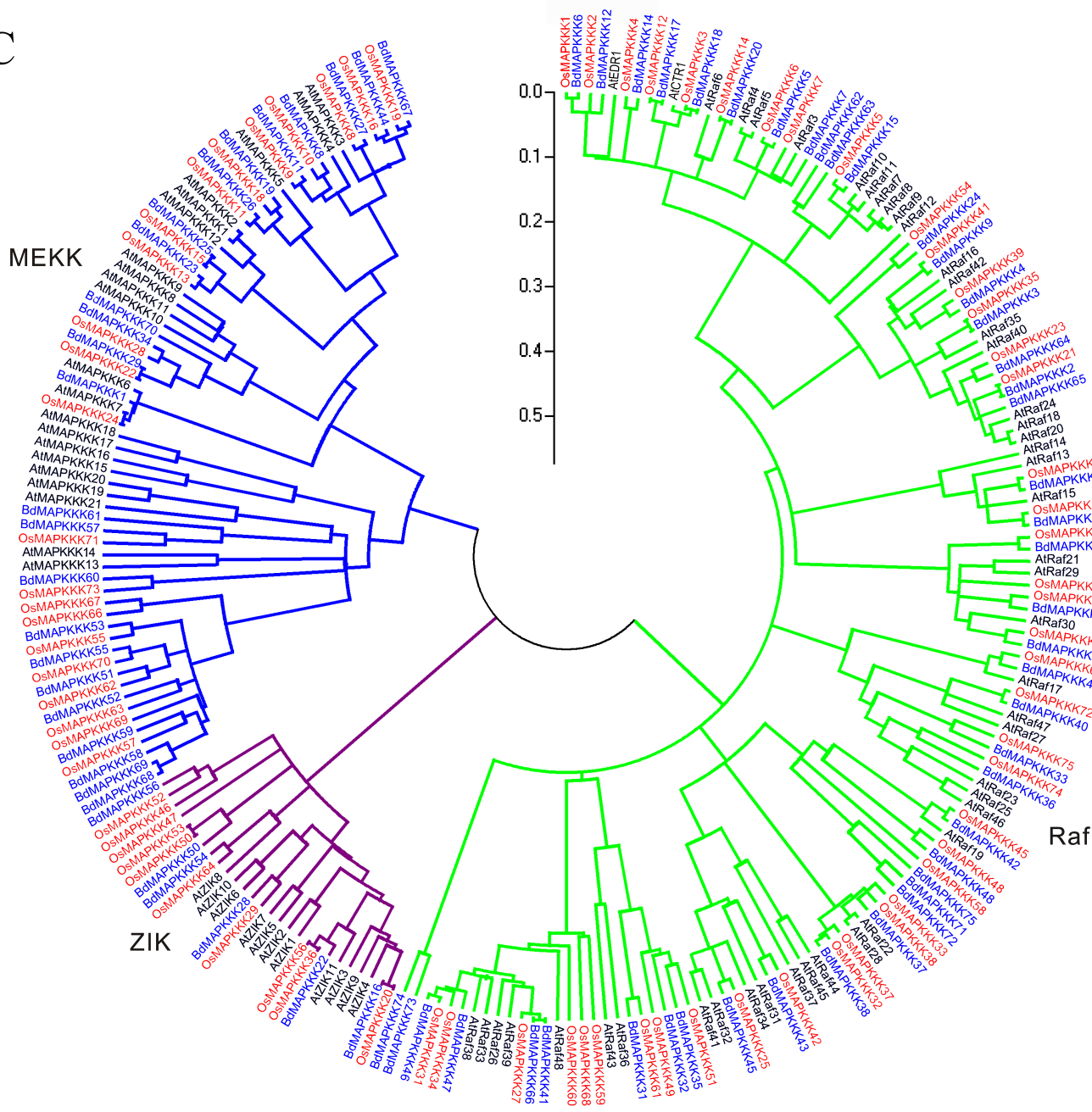

Supplement: Additional file 2: — Neighbor-joining analyses of MAPK (A), MAPKK (B) and MAPKKK (C) amino acid sequences from O. sativa, A. thaliana and B. distachyon. [file 12864_2015_1452_MOESM2_ESM.pdf]
