# Supplementary material for: Genome-wide exploration of the molecular evolution and regulatory network of mitogen-activated protein kinase cascades upon multiple stresses in Brachypodium distachyon
Source: BMC Genomics. 2015 Mar 24;16(1):228. doi: 10.1186/s12864-015-1452-1 (PMC4404688; doi:10.1186/s12864-015-1452-1)
Supplement: Additional file 3: — List of MAPK cascade kinase genes in O. sativa, A. thaliana and B. distachyon chromosomes. [file 12864_2015_1452_MOESM3_ESM.pdf]

Additional file 3A List of MAPK genes in *O. sativa*, *A. thaliana* and *B. distachyon* chromosomes.

A. thaliana		*O. sativa*		*B. distachyon*	
gene name	gene model	gene name	gene model	gene name	gene model
AtMPK1	At1g10210	OsMPK2	LOC_Os08g06060	BdMPK3	Bradi1g65810
AtMPK2	At1g59580	OsMPK3	LOC_Os03g17700	BdMPK4	Bradi3g32000
AtMPK3	At3g45640	OsMPK4	LOC_Os10g38950	BdMPK6	Bradi1g49100
AtMPK4	At4g01370	OsMPK6	LOC_Os06g06090	BdMPK7-1	Bradi1g34030
AtMPK5	At4g11330	OsMPK7	LOC_Os06g48590	BdMPK7-2	Bradi4g24912
AtMPK6	At2g43790	OsMPK14	LOC_Os02g05480	BdMPK11	Bradi3g16560
AtMPK7	At2g18170	OsMPK15	LOC_Os05g05160	BdMPK14	Bradi3g03780
AtMPK8	At1g18150	OsMPK16	LOC_Os11g17080	BdMPK16	Bradi2g36470
AtMPK9	At3g18040	OsMPK17-1	LOC_Os06g49430	BdMPK17	Bradi1g34700
AtMPK10	At3g59790	OsMPK17-2	LOC_Os02g04230	BdMPK20-1	Bradi2g44350
AtMPK11	At1g01560	OsMPK20-1	LOC_Os01g43910	BdMPK20-2	Bradi2g15317
AtMPK12	At2g46070	OsMPK20-2	LOC_Os05g50560	BdMPK20-3	Bradi1g41780
AtMPK13	At1g07880	OsMPK20-3	LOC_Os06g26340	BdMPK20-4	Bradi2g45870
AtMPK14	At4g36450	OsMPK20-4	LOC_Os01g47530	BdMPK20-5	Bradi2g16337
AtMPK15	At1g73670	OsMPK20-5	LOC_Os05g49140	BdMPK21-1	Bradi2g15620
AtMPK16	At5g19010	OsMPK21-1	LOC_Os05g50120	BdMPK21-2	Bradi2g45010
AtMPK17	At2g01450	OsMPK21-2	LOC_Os01g45620		
AtMPK18	At1g53510				
AtMPK19	At3g14720				
AtMPK20	At2g42880				

Additional file 3B List of MAPKK genes in *O. sativa, A. thaliana* and *B. distachyon* chromosomes.

A. thaliana		*O. sativa*		*B. distachyon*	
gene name	gene model	gene name	gene model	gene name	gene model
AtMKK1	AT4G26070	OsMKK1	LOC_Os06g05520	BdMKK1	Bradi1g51000
AtMKK2	At4g29810	OsMKK3	LOC_Os06g27890	BdMKK3-1	Bradi4g39490
AtMKK3	At5g40440	OsMKK4	LOC_Os02g54600	BdMKK3-2	Bradi1g41860
AtMKK4	At1g51660	OsMKK5	LOC_Os06g09180	BdMKK3-3	Bradi3g11260
AtMKK5	At3g21220	OsMKK6	LOC_Os01g32660	BdMKK4	Bradi3g53650
AtMKK6	At5g56580	OsMKK10-1	LOC_Os02g46760	BdMKK5	Bradi1g46880
AtMKK7	At1g18350	OsMKK10-2	LOC_Os03g12390	BdMKK6	Bradi1g75150
AtMKK8	At3g06230	OsMKK10-3	LOC_Os03g50550	BdMKK10-1	Bradi1g11525
AtMKK9	At1g73500			BdMKK10-2	Bradi1g69400
AtMKK10	At1g32320			BdMKK10-3	Bradi1g10800
				BdMKK10-4	Bradi1g10770
				BdMKK10-5	Bradi1g10790

Additional file 3C List of MAPKKK genes in *O. sativa*, *A. thaliana* and *B. distachyon* chromosomes.

A. thaliana		*O. sativa*		*B. distachyon*	
gene name	gene model	gene name	gene model	gene name	gene model
AtCTR1	At5g03730	OsMAPKKK1	LOC_Os03g06410	BdMAPKKK1	Bradi5g24870
AtEDR1	At1g08720	OsMAPKKK2	LOC_Os10g29540	BdMAPKKK2	Bradi1g28950
AtMAPKKK1	At1g09000	OsMAPKKK3	LOC_Os02g32610	BdMAPKKK4	Bradi1g47570
AtMAPKKK2	At1g54960	OsMAPKKK4	LOC_Os02g12810	BdMAPKKK3	Bradi3g60210
AtMAPKKK3	At1g53570	OsMAPKKK5	LOC_Os12g37570	BdMAPKKK5	Bradi3g59510
AtMAPKKK4	At1g63700	OsMAPKKK6	LOC_Os02g50970	BdMAPKKK6	Bradi1g74480
AtMAPKKK5	At5g66850	OsMAPKKK7	LOC_Os06g12590	BdMAPKKK7	Bradi1g45040
AtMAPKKK6	At3g07980	OsMAPKKK8	LOC_Os11g10100	BdMAPKKK8	Bradi5g18180
AtMAPKKK7	At3g13530	OsMAPKKK9	LOC_Os02g44642	BdMAPKKK9	Bradi1g30720
AtMAPKKK8	At4g08500	OsMAPKKK10	LOC_Os04g47240	BdMAPKKK10	Bradi2g46340
AtMAPKKK9	At4g08480	OsMAPKKK11	LOC_Os07g02780	BdMAPKKK11	Bradi3g51380
AtMAPKKK10	At4g08470	OsMAPKKK12	LOC_Os09g39320	BdMAPKKK12	Bradi3g27120
AtMAPKKK11	At4g12020	OsMAPKKK13	LOC_Os09g21510	BdMAPKKK13	Bradi3g09170
AtMAPKKK12	At3g06030	OsMAPKKK14	LOC_Os04g52140	BdMAPKKK14	Bradi3g08260
AtMAPKKK13	AT1G07150	OsMAPKKK15	LOC_Os08g32600	BdMAPKKK15	Bradi4g04470
AtMAPKKK14	AT2G30040	OsMAPKKK16	LOC_Os04g35700	BdMAPKKK16	Bradi1g23970
AtMAPKKK15	AT5G55090	OsMAPKKK17	LOC_Os09g37230	BdMAPKKK17	Bradi4g38400
AtMAPKKK16	AT4G26890	OsMAPKKK18	LOC_Os03g55560	BdMAPKKK18	Bradi3g44710
AtMAPKKK17	AT2G32510	OsMAPKKK19	LOC_Os02g35010	BdMAPKKK19	Bradi1g07650
AtMAPKKK18	AT1G05100	OsMAPKKK20	LOC_Os07g38530	BdMAPKKK20	Bradi5g21330
AtMAPKKK19	AT5G67080	OsMAPKKK21	LOC_Os07g25680	BdMAPKKK21	Bradi4g36880
AtMAPKKK20	AT3G50310	OsMAPKKK22	LOC_Os03g49640	BdMAPKKK22	Bradi2g39350
AtMAPKKK21	AT4G36950	OsMAPKKK23	LOC_Os12g40279	BdMAPKKK23	Bradi4g29500
AtRaf3	At5g11850	OsMAPKKK24	LOC_Os04g56530	BdMAPKKK24	Bradi1g60340
AtRaf4	At1g18160	OsMAPKKK25	LOC_Os02g38080	BdMAPKKK25	Bradi3g36080
AtRaf5	At1g73660	OsMAPKKK26	LOC_Os07g29330	BdMAPKKK26	Bradi1g58810
AtRaf6	At4g24480	OsMAPKKK27	LOC_Os03g43760	BdMAPKKK27	Bradi4g22760
AtRaf7	At3g06620	OsMAPKKK28	LOC_Os03g15570	BdMAPKKK28	Bradi3g51460
AtRaf8	At3g06630	OsMAPKKK29	LOC_Os02g45130	BdMAPKKK29	Bradi1g10970
AtRaf9	At3g06640	OsMAPKKK30	LOC_Os02g02780	BdMAPKKK30	Bradi3g01850
AtRaf10	At5g49470	OsMAPKKK31	LOC_Os01g45380	BdMAPKKK31	Bradi2g06260
AtRaf11	At1g67890	OsMAPKKK32	LOC_Os08g12750	BdMAPKKK32	Bradi2g19590
AtRaf12	At4g23050	OsMAPKKK33	LOC_Os02g07790	BdMAPKKK33	Bradi3g48360
AtRaf13	At2g31010	OsMAPKKK34	LOC_Os05g50190	BdMAPKKK34	Bradi1g67400
AtRaf14	At2g42630	OsMAPKKK35	LOC_Os02g54510	BdMAPKKK35	Bradi2g49700
AtRaf15	At3g58640	OsMAPKKK36	LOC_Os05g01780	BdMAPKKK36	Bradi2g57470
AtRaf16	At1g04700	OsMAPKKK37	LOC_Os04g51950	BdMAPKKK37	Bradi3g05520
AtRaf17	At1g14000	OsMAPKKK38	LOC_Os06g45300	BdMAPKKK38	Bradi3g18150
AtRaf18	At1g16270	OsMAPKKK39	LOC_Os06g08280	BdMAPKKK39	Bradi1g28110
AtRaf19	At1g62400	OsMAPKKK40	LOC_Os01g48330	BdMAPKKK40	Bradi2g49790
AtRaf20	At1g79570	OsMAPKKK41	LOC_Os06g43840	BdMAPKKK41	Bradi1g14000
AtRaf21	At2g17700	OsMAPKKK42	LOC_Os03g60150	BdMAPKKK42	Bradi1g35350
AtRaf22	At2g24360	OsMAPKKK43	LOC_Os06g50920	BdMAPKKK43	Bradi1g04080

AtRaf23	At2g31800	OsMAPKKK44	LOC_Os02g14530	BdMAPKKK44	Bradi5g10670
AtRaf24	At2g35050	OsMAPKKK45	LOC_Os06g43030	BdMAPKKK45	Bradi3g47600
AtRaf25	At2g43850	OsMAPKKK46	LOC_Os11g06140	BdMAPKKK46	Bradi2g44910
AtRaf26	At4g14780	OsMAPKKK47	LOC_Os07g08750	BdMAPKKK47	Bradi2g15560
AtRaf27	At4g18950	OsMAPKKK48	LOC_Os01g01740	BdMAPKKK48	Bradi2g00670
AtRaf28	At4g31170	OsMAPKKK49	LOC_Os05g44290	BdMAPKKK49	Bradi1g20390
AtRaf29	At4g35780	OsMAPKKK50	LOC_Os12g02250	BdMAPKKK50	Bradi4g44430
AtRaf30	At4g38470	OsMAPKKK51	LOC_Os01g54350	BdMAPKKK51	Bradi2g47510
AtRaf31	At5g01850	OsMAPKKK52	LOC_Os12g06490	BdMAPKKK52	Bradi2g47480
AtRaf32	At5g40540	OsMAPKKK53	LOC_Os11g02305	BdMAPKKK53	Bradi2g47490
AtRaf33	At5g50000	OsMAPKKK54	LOC_Os03g28300	BdMAPKKK54	Bradi1g23320
AtRaf34	At5g50180	OsMAPKKK55	LOC_Os01g50400	BdMAPKKK55	Bradi2g47500
AtRaf35	At5g57610	OsMAPKKK56	LOC_Os05g01780	BdMAPKKK56	Bradi4g41940
AtRaf36	At5g58950	OsMAPKKK57	LOC_Os05g46750	BdMAPKKK57	Bradi3g10890
AtRaf37	At5g66710	OsMAPKKK58	LOC_Os03g39150	BdMAPKKK58	Bradi2g17820
AtRaf38	At3g01490	OsMAPKKK59	LOC_Os12g41260	BdMAPKKK59	Bradi2g17830
AtRaf39	At3g22750	OsMAPKKK60	LOC_Os03g53410	BdMAPKKK60	Bradi1g65500
AtRaf40	At3g24720	OsMAPKKK61	LOC_Os01g10450	BdMAPKKK61	Bradi1g41850
AtRaf41	At3g27560	OsMAPKKK62	LOC_Os01g50420	BdMAPKKK62	Bradi4g09990
AtRaf42	At3g46920	OsMAPKKK63	LOC_Os01g50370	BdMAPKKK63	Bradi3g45660
AtRaf43	At3g46930	OsMAPKKK64	LOC_Os07g39520	BdMAPKKK64	Bradi4g02900
AtRaf44	At3g50720	OsMAPKKK65	LOC_Os07g43900	BdMAPKKK65	Bradi1g00580
AtRaf45	At3g50730	OsMAPKKK66	LOC_Os10g04010	BdMAPKKK66	Bradi1g14010
AtRaf46	At3g59830	OsMAPKKK67	LOC_Os10g04000	BdMAPKKK67	Bradi3g45790
AtRaf47	At3g58760	OsMAPKKK68	LOC_Os12g30570	BdMAPKKK68	Bradi2g17800
AtRaf48	At3g63260	OsMAPKKK69	LOC_Os05g46760	BdMAPKKK69	Bradi2g17840
AtZIK1	AT3G51630	OsMAPKKK70	LOC_Os01g50410	BdMAPKKK70	Bradi3g57740
AtZIK2	AT5G58350	OsMAPKKK71	LOC_Os02g21700	BdMAPKKK71	Bradi4g24830
AtZIK3	At3g22420	OsMAPKKK72	LOC_Os01g54480	BdMAPKKK72	Bradi4g24840
AtZIK4	AT3G04910	OsMAPKKK73	LOC_Os03g18170	BdMAPKKK73	Bradi3g13050
AtZIK5	AT3G18750	OsMAPKKK74	LOC_Os01g66860	BdMAPKKK74	Bradi3g13060
AtZIK6	AT5G41990	OsMAPKKK75	LOC_Os02g39560	BdMAPKKK75	Bradi4g41870
AtZIK7	AT1G49160				
AtZIK8	AT5G55560				
AtZIK9	AT5G28080				
AtZIK10	AT1G64630				
AtZIK11	AT3G48260				
