# Supplementary material for: Genome-wide exploration of the molecular evolution and regulatory network of mitogen-activated protein kinase cascades upon multiple stresses in Brachypodium distachyon
Source: BMC Genomics. 2015 Mar 24;16(1):228. doi: 10.1186/s12864-015-1452-1 (PMC4404688; doi:10.1186/s12864-015-1452-1)
Supplement: Additional file 4: — The amino acid sequences of MAPKKK members in B. distachyon, A. thaliana and O. sativa. [file 12864_2015_1452_MOESM4_ESM.pdf]

Additional file 4 The amino acid sequences of MAPK cascade members in *B. distachyon*, *Arabidopsis* and *O. sativa*.

Gene name	Amino acid sequences
AtMPK1	MATLVDPPNGIRNEGKHYFSMWQTLFEIDTKYMPIKPIGRGAYGVVCSSVNSDTNEKVAIKKIHNVYENRIDALRTLRELKLLRHLRHENVIALKDVMMPIHKMSFKDVYLVYELMDTDLHQIIKSSQRLSNDHCQYFLFQLLRGLKYIHSANILHRDLKPGNLLVNANCDLKICDFGLARASNTKGQFMTEYVVTRWYRAPELLLCCDNYGTSIDVWSVGCIFAELLGRKPIFQGTECLNQLKLIVNIIGSQREEDLEFIVNPKAKRYIRSLPYSPGMSLSRLYPCAHVLAIDLLQKMLVFDPSKRISASEALQHPYMAPLYDPNANPPAQVPIDLDVDEDLREEMIREMIWNEMLHYHPQASTLNTEL
AtMPK2	MATPVDPPNGIRNQGKHYFSMWQTLFEIDTKYMPIKPIGRGAYGVVCSSVNRESNERVAIKKIHNVFENRIDALRTLRELKLLRHLRHENVVALKDVMMANHKRSFKDVYLVYELMDTDLHQIIKSSQVLSNDHCQYFLFQLLRGLKYIHSANILHRDLKPGNLLVNANCDLKICDFGLARTSNTKGQFMTEYVVTRWYRAPELLLCCDNYGTSIDVWSVGCIFAELLGRKPVFPGTECLNQIKLIINILGSQREEDLEFIDNPKAKRYIESLPYSPGISFSRLYPGANVLAIDLLQKILVLDPSKRISVTEALQHPYMAPLYDPSANPPAQVPIDLDVDEDEDLGAEMIRELMWKEMIHYHPEAATINNNEVSEF
AtMPK3	MNTGGGQYTDFPAVDTHGGQFISYDIFGSLFEITSKYRPPIIPIGRGAYGIVCSVLDTETNELVAMKKIANAFDNHMDAKRTLREIKLLRHLDHENIIAIRDVVPPPLRRQFSDVYISTELMDTDLHQIIRSNQSLSEEHCQYFLYQLLRGLKYIHSANIIHRDLKPSNLLLNANCDLKICDFGLARPTSENDFMTEYVVTRWYRAPELLLNSSDYTAAIDVWSVGCIFMELMNRKPLFPGKDHVHQMRLLTELLGTPTESDLGFTHNEDAKRYIRQLPNFPRQPLAKLFSHVNPMAIDLVDRMLTFDPNRRITVEQALNHQYLAKLHDPNDEPICQKPFSFEFEQQPLDEEQIKEMIYQEAIALNPTYG
AtMPK4	MSAESCFGSSGDQSSSKGVATHGGSYVQYNVYGNLFEVSRKYVPPLRPIGRGAYGIVCAATNSETGEEVAIKKIGNAFDNIIDAKRTLREIKLLKHMDHENVIAVKDIIKPPQRENFNDVYIVYELMDTDLHQIIRSNQPLTDDHCRFFLYQLLRGLKYVHSANVLHRDLKPSNLLLNANCDLKLGDFGLARTKSETDFMTEYVVTRWYRAPELLLNCSEYTAAIDIWSVGCILGETMTREPLFPGKDYVHQLRLITELIGSPDDSSLGFLRSDNARRYVRQLPQYPRQNFAARFPNMSAGAVDLLEKMLVFEPSRRITVDEALCHPYLAPLHDINEEPVCVRPFNFDFEQPTLTEENIKELIYRETVKFNPQDSV
AtMPK5	MAKEIESATDLGDTNIKGVLVHGGRYFQYNVYGNLFEVSNKYVPPIRPIGRGAYGFVCPAVDSETHEEIAIKKIGKAFDNKVDAKRTLREIKLLRHLEHENVVVIKDIIRPPKKEDFVDVYIVFELMDTDLHQIIRSNQSLNDDHCQYFLYQILRGLKYIHSANVLHRDLKPSNLLLNSNCDLKITDFGLARTTSETEYMTEYVVTRWYRAPELLLNSSEYTSAIDVWSVGCIFAEIMTREPLFPGKDYVHQLKLITELIGSPDGASLEFLRSANGGKYVKELPKFPRQNFSARFPSMNSTAIDLLEKMLVFDPVKRITVEEALCYPYLSALHDLNDEPVCSNHFSFHFEDPSSTEEEIKELVWLESVKFNPLPSI
AtMPK6	MDGGSGQPAADTEMTEAPGGFPAAAPSPQMPGIENIPATLSHGGRFIQYNIFGNIFEVTAKYKPPIMPIGKGAYGIVCSAMNSETNESVAIKKIANAFDNKIDAKRTLREIKLLRHMDHENIVAIRDIIPPPLRNAFNDVYIAYELMDTDLHQIIRSNQALSEEHCQYFLYQILRGLKYIHSANVLHRDLKPSNLLLNANCDLKICDFGLARVTSESDFMTEYVVTRWYRAPELLLNSSDYTAAIDVWSVGCIFMELMDRKPLFPGRDHVHQLRLLMELIGTPSEEELEFLNENAKRYIRQLPPYPRQSITDKFPTVHPLAIDLIEKMLTFDPRRRITVLDALAHPYLNSLHDISDEPECTIPFNFDFENHALSEEQMKELIYREALAFNPEYQQ
AtMPK7	MAMLVEPPNGIKQQGKHYYSMWQTLFEIDTKYVPIKPIGRGAYGVVCSSINRETNERVAIKKIHNVFENRVDALRTLRELKLLRHVRHENVIALKDVMLPANRTSFKDVYLVYELMDTDLHQIIKSSQSLSDDHCKYFLFQLLRGLKYLHSANILHRDLKPGNLLVNANCDLKICDFGLARTSQGNEQFMTEYVVTRWYRAPELLLCCDNYGTSIDVWSVGCIFAEILGRKPIFPGTECLNQLKLIINVVGSQQESDIRFIDNPKARRFIKSLPYSRGTHLSNLYPQANPLAIDLLQRMLVFEPTKRISVTDALLHPYMAGLFEPGTNPPAHVPISLDIDENMEEPVIREMMWNEMLYYHPEAEILNA
AtMPK8	MGGGGNLVDGVRRWLFQRPSSSSSSSSSNNNNNNHEQPIFNSSSFSSSSNPNHSANSGELIIEEDLDFSGLTLINVPKRNHLPMDPHKKGETEFFTEYGEANRYQIQEVVGKGSYGVVASAVDSHTGERVAIKKINDVFEHVSDATRILREIKLLRLLRHPDVVEIKHIMLPPSRREFPHIYVVFELMESDLHQVIKANDDLTPEHYQFFLYQLLRGLKYVHAANVFHRDLKPKNILANADCKLKICDFGLARVSFNDAPTAIFWTDYVATRWYRAPELCGSFFSKYTPAIDIWSVGCIFAEMLLGKPLFPGKNVVHQLDLMTDFLGTPPPESISRIRNEKARRYLSSMRKKQPVPFSHKFPKAEPLALRLLERLLAFDPKDRASAEDALADPYFSGLSNSEREPTTQPISKLEFDFERKKLVKDDVRELLYREILEYHPQMLEEYLRGGDQLSFMYPSGVDRFKRQFAHLEENQGKPVAAGGGRSTALHRHHASLPRERVPAPNGETAEESSDVERRAAAAVASTLESEEQTMEEVTVLVTS
AtMPK9	MDPHKKVALETEFFTEYGEASRYQIQEVIGKGSYGVVASAIDTHSCEKVAIKKSNDVFEHVSDATRILREIKLLRLLRHPDIVEIKHVMLPPSRRDFRDIYVVFELMESDLHQVIKANDDLTPEHYQFFLYQLLRGLKFIHTANVFHRDLKPKNILANSDCKLKICDFGLARVSFNDAPSAIFWTDYVANRWYRAPELCGSFFSKYTPAIDIWSIGCIFAEMLTGKPLFPGKNVVHQLDIMTDLLGTPPPEAIARIRNEKARRYLGNMRRKPPVPFTHKFPHVDPLALRLLHRLLAFDPKDRPSAEEALADPYFYGLANVDREPSTQPIPKLEFEFERRKLTKEDVRELIYREILEYHPQMLQEYLRGGEQTSFMYPSGVDRFKRQFAHLEENYGKGEKGSPLQRQHASLPRERVPAPKKENGSHNHDIENRSIASLVTTLESPPTSQHEGSDYRNGTSQQGYSARSLLKSASISASKCIGMKPRNKSEYGESNNDTVDALSQKVAALHT
AtMPK10	MEPTNDAETLETQGEVTTAIWPSSQILKTTIDIPGTLSHDGRYIQYNLFGHIFELPAKYKPPIRPIGRGACGIVCSAVDSETNEKVAIKKITQVFDNTIEAKRTLREIKLLRHFDHENIVAIRDVILPPQRDSFEDVYIVNELMEFDLYRTLKSDQELTKDHGMYFMYQILRGLKYIHSANVLHRDLKPSNLLLSTQCDLKICDFGLARATPESNLMTEYVVTRWYRAPELLLGSSDYTAAIDVWSVGCIFMEIMNREPLFPGKDQVNQLRLLLELIGTPSEEELGSLSEYAKRYIRQLPTLPRQSFTEKFPNVPPLAIDLVEKMLTFDPKQRISVKEALAHPYLSSFHDITDEPECSEPFNFDLDEHPFSEEQFRELIYCEALAFNPETSND
AtMPK11	MSIEKPFFGDDSNRGVSINGGRYVQYNVYGNLFEVSKKYVPPLRPIGRGASGIVCAAWNSETGEEVAIKKIGNAFGNIIDAKRTLREIKL

	LKHMDHDNVIAIIDIIRPPQPDNFNDVHIVYELMDTDLHHIIRSNQPLTDDHSRFFLYQLLRGLKYVHSANVLHRDLKPSNLLLNANCDLKIGDFGLARTKSETDFMTEYVVTRWYRAPELLLNCSEYTAAIDIWSVGCILGEIMTREPLFPGRDYVQQLRLITELIGSPDDSSLGFLRSDNARRYVRQLPQYPRQNFAARFPNMSVNAVDLLQKMLVFDPNRRITVDEALCHPYLAPLHEYNEEPVCVRPFHFDFEQPSLTEENIKELIYRESVKFNP
AtMPK12	MDLVSSRDTLGDPSTSTKALLLGFSRSRSDHCIIMSGESSSGSTEHCIKVVPTHGGRYVQYNVYGQLFEVSRKYVPPIRPIGRGACGIVCAAVNSVTGEKVAIKKIGNAFDNIIDAKRTLREIKLLRHMDHENVITIKDIVRPPQRDIFNDVYIVYELMDTDLQRILRSNQTLTSDQCRFLVYQLLRGLKYVHSANILHRDLRPSNVLLNSKNELKIGDFGLARTTSDTDFMTEYVVTRWYRAPELLLNCSEYTAAIDIWSVGCILGEIMTGQPLFPGKDYVHQLRLITELVGSPDNSSLGFLRSDNARRYVRQLPRYPKQQFAARFPKMPTTAIDLLERMLVFDPNRRISVDEALGHAYLSPHHDVAKEPVCSTPFSFDFEHPSCTEEHIKELIYKESVKFNPDH
AtMPK13	MEKREDGGILTYDGRYVMYNVLGNIFELSSKYIPPIEPIGRGAYGIVCCATNSETNEEVAIKKIANAFDNRVDAKRTLREIKLLSHMDHDNVIKIKDIIELPEKERFEDVYIVYELMDTDLHQIIRSTQTLTDDHCQYFLYQILRGLKYIHSANVLHRDLKPSNLVLNTNCDLKICDFGLARTSNETEIMTEYVVTRWYRAPELLLNSSEYTGAIDIWSVGCIFMEILRRETLFPGKDYVQQLKLITELLGSPDDSDLDFLRSDNARKYVKQLPHVQKQSFREKFPNISPMALDLAEKMLVFDPSKRITVDEALKQPYLASLHEINEEPTCPTPFSFDFEETALDEQDIKELVWRESLHFKNM
AtMPK14	MAMLVDPPNGIRQEGKHYYTMWQTLFEIDTKYVPIKPIGRGAYGVVCSSINSETNERVAIKKIHNVFENRIDALRTLRELKLLRHVRHENVISLKDVMLPTHRYSFRDVYLVYELMDSDLNQIIKSSQSLSDDHCKYFLFQLLRGLKYLHSANILHRDLKPGNLLVNANCDLKICDFGLARTYEQFMTEYVVTRWYRAPELLLCCDNYGTSIDVWSVGCIFAEILGRKPIFPGTECLNQLKLIINVVGSQQDWDLQFIDNQKARRFIKSLPFSKGTHFSHIYPHANPLAIDLLQRMLVFDPTKRISVSDALLHPYMEGLLEPECNPSENVPVSSLEIDENMEGDMIREMMWEEMLHYLPRA
AtMPK15	MGGGGNLVDGVRRWLFFQRRPSSSSSSNNHDQIQNPPTVSNPNDDEDLKKLTDPSKLRQIKVQQRNHLPMEKKGIPNAEFFTEYGEANRYQIQEVVGKGSYGVVGSAIDTHTGERVAIKKINDVFDHISDATRILREIKLLRLLLHPDVVEIKHIMLPPSRREFRDVYVVFELMESDLHQVIKANDDLTPEHHQFFLYQLLRGLKYVHAANVFHRDLKPKNILANADCKLKICDFGLARVSFNDAPTAIFWTDYVATRWYRAPELCGSFFSKYTPAIDIWSVGCIFAEMLLGKPLFPGKNVVHQLDIMTDFLGTPPPEAISKIRNDKARRYLGNMRKKQPVPFSKKFPKADPSALRLLERLIAFDPKDRPSAEEALADPYFNGLSSKVREPSTQPISKLEFEFERKKLTKDDIRELIYREILEYHPQMLEEYLRGGNQLSFMYPSGVDRFRRQFAHLEENQGPGGRSNALQRQHASLPRERVPASKNETVEERSNDIERRTTAAVASTLDSPKASQQAEGTENGGGGGYSARNLMKSSSISGSKCIGVQSKTNIEDSIVEEQDETVAVKVASLHNS
AtMPK16	MQPDHRKKSSVEVDFFTEYGEGSRYRIEEVIGKGSYGVVCSAYDTHTGEKVAIKKINDIFEHVSDATRILREIKLLRLLRHPDIVEIKHILLPPSRREFRDIYVVFELMESDLHQVIKANDDLTPEHYQFFLYQLLRGLKYIHTANVFHRDLKPKNILANADCKLKICDFGLARVAFNDTPTAIFWTDYVATRWYRAPELCGSFFSKYTPAIDIWSIGCIFAELLTGKPLFPGKNVVHQLDLMTDMLGTPSAEAIGRVRNEKARRYLSSMRKKKPIPFSHKFPHTDPLALRLLEKMLSFEPKDRPTAEEALADVYFKGLAKVEREPSAQPVTKLEFEFERRRITKEDVRELIYRESLEYHPKMLKEYLDGSEPTNFMYPSAVEHFKKQFAYLEEHYKNGTSHNPPERQQHASLPRACVLYSDNNHPVAQQSSAEVTDGLSKCSIRDERPRGADRNAQMPMSRIPINVPQTIQGAAVARPGKVVGSVLRYNNCGAATGVEALEQQQRRMVRNPAAASQYPKRTQPCKSNRGDEDCATAAEGPSRLKPNTQYIPQKVSAAQDTAMSRWY
AtMPK17	MLEKEFFTEYGEASQYQIQEVVGKGSYGVVASAECPHTGGKVAIKKMTNVFEHVSDAIRILREIKLLRLLRHPDIVEIKHIMLPPCRKEFKDIYVVFELMESDLHHVLKVNDDLTPQHHQFFLYQLLRGLKFMHSAHVFHRDLKPKNILANADCKIKICDLGLARVSFTDSPSAVFWTDYVATRWYRAPELCGSFYSNYTPAIDMWSVGCIFAEMLTGKPLFPGKNVVHQLELVTDLLGTPSPITLSRIRNEKARKYLGNMRRKDPVPFTHKFPNIDPVALKLLQRLIAFDPKDRPSAEEALADPYFQGLANVDYEPSRQPISKLEFEFERRKLTRDDVRELMYREESLKTFFFVLFLDMKQILEYHPQMLQEYLQGEENINSHFLYPRESVSYFGESILISSGVDQFKQEFARLEEHNDDEEEHNSPPHQRKYTSLPRERVCSSEDEGSDSVHAQSSSASVVFTPPQTPNTATGLSSQKASQVDKAATPVKRSACLMRSDSICASRCVGVSSAVS
AtMPK18	MEFFTEYGDANRYRILEVIGKGSYGVVCAAIDTHTGEKVAIKKINDVFEHISDALRILREVKLLRLLRHPDIVEIKSIMLPPSKREFKDIYVVFELMESDLHQVIKANDDLTREHHQFFLYQMLRALKFMHTANVYHRDLKPKNILANANCKLKVCDFGLARVAFNDTPTTVFWTDYVATRWYRAPELCGSFFSKYTPAIDVWSIGCIFAEVLTGKPLFPGKSVVHQLELITDLLGTPKSETISGVRNDKARKYLTEMRKKNPVTFSQKFSKADPLALRLLQRLLAFDPKDRPTPAEALADPYFKGLSKIEREPSSQQISKMEFEFERRRLTKDDIRELIYREILEYHPQLLKDYMSGSEGSNFVYPSAIGHLRQQFTYLEENSSRNGPVIPLERKHASLPRSTVHSTVVHSTSQPNLGATDSRRVSFEPSKNGASSAGHPSTSAYPTKSIGPPPRVPPSGRPGRVVESSVSYENGRNLKEAYFRSAVSSPHCYFRPNTMTNPENRNIEASSFPPKPQNPVHQFSPTEPPAATTNQADVETMNHPNPYFQPQLPKTDQLNNNTHMAIDAKLLQAQSQFGPAGAAAVAVAAHRNIGTISYSAAS
AtMPK19	MQKTQEKKNMKEMEFFTEYGDANRYRILEVIGKGSYGVVCAAIDTQTGEKVAIKKINDVFEHVSDALRILREVKLLRLLRHPDIVEIKSIMLPPSKREFKDIYVVFELMESDLHQVIKANDDLTREHHQFFLYQMLRALKYMHTANVYHRDLKPKNILANANCKLKVCDFGLARVSFNDTPTTVFWTDYVATRWYRAPELCGSFCSKYTPAIDIWSIGCIFAEVLTGKPLFPGKSVVHQLDLITDLLGTPKSETIAGVRNEKARKYLNEMRKKNLVPFSQKFPNADPLALRLLQRLLAFDPKDRPTAAEALADPYFKCLAKVEREPSCQPISKMEFEFERRRLTKDDIRELIYREILEYHPQLLKDYMNSEGSSFLYPSAIGHLRKQFAYLEENSGKSGPVIPPDRKHASLPRSAVHSSAVNSNAQPSLNASDSRRVSIEPSRNGVVPSTSAYSTKPLGPPPRVPSGKPGRVVESSVTYENDRNLKESSYDARTSYYRSTVLPPQTVSPNCYFLPNTMNQEKRSGTEAASQPKPQFVPTQCNSAKPAELNPNPYVQSQHKVGIDAKLLHAQSQYGPAGAAAVAVAAHRNIGAVGYGMS
AtMPK20	MQQDNRKKNNLEMEFFSDYGDANRFKVQEVIGKGSYGVVCSAIDTLTGEKVAIKKIHDIFEHISDAARILREIKLLRLLRHPDIVEIKHIMLPPSRREFKDIYVVFELMESDLHQVIKANDDLTREHYQFFLYQLLRALKYIHTANVYHRDLKPKNILANANCKLKICDFGLARVAFNDTPTTIFWTDYVATRWYRAPELCGSFYSKYTPAIDIWSIGCIFAEVLMGKPLFPGKNVVHQLDLMTDLLGTPSLDTISRVRNEKARRYLTSMRKKPPIPFAQKFPNADPLSLKLLERLLAFDPKDRPTAEEALADPYFKGLAKVEREPSCQPITKMEFEFERRKVTKEDIRELISREILEYHPQLLKDHMNGADKASFLYPSAVDQFRRQFAHLEENSGKTGPVAPLERKHASLPRSTVIHSTAVARGGQPKLMNNTNTLNPETTQNIPFNHATIQAQQRNLSAAKPSTFMGPVAPFDNGRISRDAYDPRSFIRSTNLPFSQQSAATVAMGKQQERRTTMEPEKQARQISQYNRYAPDVAINIDNNPFIMARTGMNKAENISDRIIDTNLLQATAGIGVAAAAAAAAPGGSAHRKVGAVRYGMSKMY

BdMPK3	MDGAPVAEFRPTMTHGGRFLLYNIFGNQFEITAKYQPPIMPIGRGAYGIVCSVMNFETREMVAIKKIANAFDNNMDAKRTLREIKLLRHLDHENIVGLRDVIPPAIPQSFNDVYIATELMDTDLHHIIRSNQELSEEHCQYFLYQLLRGLKYIHSANVIHRDLKPSNLLLNANCDLKICDFGLARPSSESDMMTEYVVTRWYRAPELLLNSTDYSAAIDVWSVGCIFMELINRAPLFPGRDHMHQMRLITEVIGTPTDDDLGFIRNEDARRYMRHLPQFPRRPFPAQFPRVQPAALDLIERMLAFNPLQRITVEEALEHPYLERLHDIADEPICTDPFSFDFEQHPLTEDQMKQLIFNEALELNPNFRY
BdMPK4	MDTSGGGGAGGAAQIQGMATHGGRYVLYNVYGNLFEVASKYAPPIRPIGRGAYGIVCAAVSSDTGEEVAIKKIGNAFDNHIDAKRTLREIKLLRHMDHENIIAIKDIIRPPRRDDFKDVYIVTELMDTDLHQIIRSNQPLTDDHCQYFLYQLLRGLKYVHSANVLHRDLKPSNLFLNANCDLKIADFGLARTTSETDLMTEYVVTRWYRAPELLLNCSQYTAAIDVWSVGCILGEIITRQPLFPGRDYIQQLKLITELIGSPDDSSLGFLRSDNARRYMKQLPQYPRQDFRLRFRNMSDGAVDLLERMLVFDPSRRITVDEALHHPYLASLHDINEEPTCPAPFSFDFEQPSFTEEHMKELIWRETLAFNPDPPY
BdMPK6	MDGGAQPPDAEMADAGAAVPGAGAGAAGTMENIQATLSHGGRFIQYNIFGNVFEVTVKYKPPILPIGKGAYGIVCSALNSETGEQVAIKKIANAFDNKIDAKRTLREIKLLRHMDHENIVAIRDIIPPAQRNSFNDVYIAYELMDTDLHQIIRSNQALSEEHCQYFLYQILRGLKYIHSANVLHRDLKPSNLLLNANCDLKICDFGLARTTSETDFMTEYVVTRWYRAPELLLNSSEYTAAIDVWSVGCIFMELMDRKPLFPGRDHVHQLRLLMELIGTPNEADLDFVNENARRYIRQLPRHARQSFSEKFPHVHPSAIDLVEKMLTFDPRQRITVEGALAHPYLASLHDISDEPVCTMPFSFDFEQHALSEEQMKDLIHQEALAFNPDYQ
BdMPK7-1	MPSRFPFLSPGCFIRLLSGSASSDLLLLLPRRGQSAPRARVLLKMAMMVDPPNGTGNHGKHYYTMWQTMFEIDTKYVPIKPIGRGAYGIVCSSTNQENNEKVAIKKINNVFDNRVDALRTLRELKLLRHLRHENVIALKDIMMPVHRRSFKDVYLVSELMDTDLHQIIKSSQPLSNDHCQYFLFQLLRGLKYLHSAGILHRDLKPGNLLVNANCDLKICDFGLARTNNTKGQFMTEYVVTRWYRAPELLLCCDNYGTSIDVWSVGCIFAELLGRKPIFPGTECLNQLKLIVNVLGTMSENDLEFIDNPKARKYIKSLPYTPGTPLTSMYPQAHPLAIDLLQKMLVFDPSKRISVTEALEHPYMSPLYDPSANPPAQVPIDLDIDENLGVEMIREMLWQEMLQYHPEAARMVNM
BdMPK7-2	MAMMVDPPNGTGNHGKHYYTMWQTMFEIDTKYMPIKPIGRGAYGIVCSSTNQENNEKVAIKKINNVFDNRVDALRTLRELKLLRHLRHENVIALKDIMMPVHRRSFKDVYLVFELMDTDLHQIIKSSQPLSNDHCQYFLFQLLRGLKYLHSAGILHRDLKPGNLLVNANCDLKICDFGLARTNNTKGQFMTEYVVTCLYRAPELLLCCDNYGTSIDVWSVGCIFAELLGRKPIFPRTQCLDQLKLIVNVLGTMSENDLEFIDNPKARKYIKSLPYTPGTPLTSMYPQAHPLAIDLLQKMLVFDPSKRISVTEALEHPYMSPLYDPSANPPAQVPIDLDIDENLGVEMIREMLWQEMLQYHPEAARMVNM
BdMPK11	MRMEGGGAGPAAAAAAGGAHGLGEAQIKGTLTHGGRYVQYNVYGNLFEVSAKYVPPIRPVGRGACGIICAAINVQTREEVAIKKIGNAFDNQIDAKRTLREVKLLRHMNHENVISIKDIIRPPRRENFNDVYIVYELMDTDLHHLLRSNQPLTDDHCQYFLYQVLRGLKYVHSANVLHRDLRPSNLLLNAKCDLKIGDFGLARTTTETDFMMEYVVTRWYRAPELLLNCSEYTGAIDMWSVGCILGEIATREPLFPGKDYVHQLRLITELLGSPDDTSLGFLRSDNARRYVRSLPQYPKQQFRSRFPNMSSGAMDLLERMLVFDPNKRITVDEALCHPYLASLHEINDEPVCPAPFSFDFEQPSFTEEDIKELIWRESVKFNPEPIH
BdMPK14	MAMLVDPPNGMGNQGKHYYSMWQTLFEIDTKYVPIKPIGRGAYGIVCSSINRETNEKVAIKKIHNVFDNRVDALRTLRELKLLRHLRHENVISLKDIMMPVQRRSFKDVYLVYELMDTDLHQIIKSPQGLSNDHCQYFLFQLLRGLKYLHSAEILHRDLKPGNLLVNANCDLKICDFGLARTNSSKGQFMTEYVVTRWYRAPELLLCCDNYGTSIDVWSVGCIFAELLGRKPIFPGTECLNQLKLIVNVLGTMSESDLEFIDNPKARRYIKTLPYTPGVPLASMYPHAHPLAIDLLQKMLIFDPTKRISVTEALEHPYMSPLYDPSANPPAQVPIDLDLDENISADMIREMMWQEMLHYHPEAATAISM
BdMPK16	MDFFTEYGEGNRYKIEEVIGKGSYGVVCSALDTHTGDKVAIKKINDIFEHVSDATRILREIKLLRLLRHPDIVEIKHILLPPSRREFKDIYVVFELMESDLHQVIKANDDLTPEHYQFFLYQLLRGLKYIHTANVFHRDLKPKNILANADCKLKICDFGLARVAISDTPTAIFWTDYIATRWYRAPELCGSFFSKYTPAIDIWSIGCIFAELLTGKPLFPGKNVVHQLDIITDLLGTPSAETIARIRNEKARRYLSSMRRKKTVPFTQKFPNADPLALNLLERMLAFDPKDRPSAEEALADPYFKNIASVDREPSAQPITKLEFEFERRRITKDDIRELIYREILEYHPNMLREFLEGAEPTGFMYPSAVDHFKKQFTFLEEHYAKGSTAAPPERQHNSLPRPSVIYSDNRPQNAANITDDLSRCAIRDNTQKAQKDSASVGANRIPHGAAAAAARPGKVVGSVLRYGNCSTSGTEQYEQRRVVRSPGIAPNGVTSGSSYPRRNNTCKSETGEAERIDTNQGGPPKPYVANKLPATVDGRNGHW
BdMPK17	MVGGGGIVDGFRRLFHRRTPSGSGPSSNQSSAGEESSDIDAVVEDLDLVGLRAIRVPKRKMPLPVESHKKNILEKEFFTEYGEASQYQIQEVIGKGSYGVVAAAVDTRTGERVAIKKINDVFEHVSDATRILREVKLLRLLRHPDVVEIKHIMLPPSRREFQDIYVVFELMESDLHQVIRANDDLTPEHYQFFLYQLLRALKYIHAANVFHRDLKPKNILANSDCKLKICDFGLARVSFNDAPTAIFWTDYVATRWYRAPELCGSFFSKYTPAIDIWSIGCIFAELLTGRPLFPGKNVVHQLDIITDLLGTPSSETLSRIRNEKARRYLSCMRKKHPVPLTQKFPNVDPLALRLLGRLLAFDPKDRPSAEEALADPYFAVLANVEREPSRNPISKLEFEFERRKVTKDDVRELIYREILEYHPQMLQEYMQGGEQLSFLYPSGVDRFKRQFAHLEENYSKGERGSPLQRKHASLPRQRVGASNDNNDQHASDQERGADSIAPATGNPPGSQDAGQQHVSGGQNGVGSTNISPRSYQKSASISASKCVVVNANKNPEYDDDISEEMEGAVDGLSEKVSRMHP
BdMPK20-1	MQQDQRKKSSAEAEFFTEYGDASRYKIQEIVGKGSYGVVCSAIDVHTGEKVAIKKIHDIFEHISDAARILREIKLLRLLRHPDIVEIKHIMLPPSRRDFKDIYVVFELMESDLHQVIKANDDLTKEHYQFFLYQLLRALKYIHTANVYHRDLKPKNILANSNCKLKICDFGLARVAFNDTPTTIFWTDYVATRWYRAPELCGSFFSKYTPAIDVWSIGCIFAEVLTGKPLFPGKNVVHQLDLMTDLLGTPSMDTISRVRNEKARRYLSSMRKKEPIPFSQKFPNADPLALDLLQRLLAFDPKDRPTAGEALSHPYFKGLAKVEREPSCQPITKMEFEFERRRATKEDIRELIFREILEYHPQLLKDYINGTERTTFLYPSAVDQFRKQFAHLEENSESGPVVPMDRKHTSLPRSTIVHSAPIHAKEQPRIGPSRDRPLTDESYKNPRDSEKYSGNVPRSSHHSQAPQRVPTARPGRVVGPVLPYENVGTSHPYDPRRVAMNSGYPPQQQIPQTYGYYQTTGKSACSEPSQAERYTLHQQAYACANSSTAPDVALDMRAPPFHQSAGPKSGSSDRLTAETNLYTRSLNGIVATTSGVAASAHRKVSVVPYGMSQMY
BdMPK20-2	MSTDVDFFSEYGDANRYRIQEVIGKGSYGVVCSAMDMQTRQKVAIKKIHNIFDHVSDAARILREIKLLRLLRHPDVVEIKHIMLPPSRKDFKDIYVVFELMESDLHQVIKANDDLTKEHYQFFLYQLLRALKYIHTASVYHRDLKPKNILANSNCKLKICDFGLARVAFNDTPTTVFWTDYVATRWYRAPELCGSFFTKYTPAIDIWSIGCIFAEVLTGKPLFPGKNVVHQLDLMTDLLGTPSMDTISRVRNEKARRYLSSMRKKDPVPFSKKFPNADPLGLKLLEKLLAFDPKDRPTAEEALTDPYFKSLSKPDREPSCQPIRKVEFDFEHRRMSKDDIRELIFQEILEYHPQLLK

	NYIDGTEKTTFLYPSAVDQFKKQFSHLEESDGSGPVVPTERKHASLPRSTTVHSTPIPAKEQPLVASSRGRPIANEPCKPWAPGNVPGASQTAHVAQAGRAVGVGSVPPYESGSGKYPYDATSRPAVSSGYPPQQKIPQTYGYHHHHQTPGAGQSSQAMGGYACGYTKGTTPPPAAAQDMRASPYHHRSAGTKNDPLNRLAAESDIYTRSLNGIVAAAASAGTGAHRKVGAVPFGMSGMY
BdMPK20-3	MQHGDLHKKSAAEMDFFTAYDDANRYKILEVIGKGSYGLVCSANDLQTGEKVAIKKIHNIFEHISDAARILREIKLLRLLRHPDVVEIKHILLPPSKKDFKDIYVVFELMESDLHQVIKANDDLTREHYQFFLYQMLRALKYMHTANVYHRDLKPKNVLANANCKLKICDFGLARVAFSDAPTTVFWTDYVATRWYRAPELCGSFYSKYTPAIDIWSIGCIFAEVLIGKPLFPGKNVVHQLDLITDILGTPSLDAISRVRNDKARKYLTCMRKKQPASFSQKFPKADPLALQLLRRLLAFDPKDRPSAEEALADPYFNGLAKVEREPSCQPIPKIEFEFEGRRVTKEDIKELIFEEILEYHPQLLKEHIIGKERPNFVHLSAVDQFKKHFTQLEENDNETGAAVSLQRKHSSLPRQAFNHR
BdMPK20-4	MQTTEQQRKKGSSEMDFFSEYGDANRYKIQEVIGKGSYGVVCSAIDQHTGDKVAIKKIHNIFEHLSDAARILREIKLLRLLRHPDIVEIRHIMLPPSRRDFKDIYVVFELMDTDLHQVIKANDDLTKEHHQFFLYQMLRALKYIHTANVYHRDLKPKNILANANCKLKICDFGLARVAFNDTPTTVFWTDYVATRWYRAPELCGSFFTKYSRAIDIWSIGCIFAEILTGKPLFPGKNVVHQLDLMTDLLGTPSLDTVSRIRNEKARRYLSSMRKKQSVSFSERFPKADPAALKLLQRLLAFDPKDRPTAEEALADPYFKGLGKVEREPSCQPITKMEFEFERKNVTKADVKELIFREILEYHPQLLKDYMNGTEKTNFLYPSAVDNFRRQFANLEENGGKGGAVVPPDRKHVSLPRNTTVHSTPIPPKDQKYSQVPQRIPTGRPGRVVGPVIPFENSSTMDPYSQRRVARNPVLPAAATNLSAYAYRNSDNSERELQQELEKDRMQYHPMQRFMDAKMVSPDLRSTSYYMPKGVPKADVAERSALQSNMMQGIAPFNGIATVGGAFNKVSAVQYGVSRMY
BdMPK20-5	MPEANAAARGGGGGGDARAAEQRSNNKNSEEMGFFSEYVDASRYKILEIIGKGSYGVVCSAIDQETGDKVAIKKIQNIFEHLSDAARILREIKLLRLLRHPDIVQIKHIMLPPSRRDFRDIYVVFELMDTDLHQVIKANDDLTKEHYQFFLYQMLRALKYIHTANVYHRDLKPKNILANANCKLKICDFGLARVAFNDTPTTVFWTDYVATRWYRAPELCGSFFTKYSPAIDTWSIGCIFAEILTGKPLFPGKNVVHQLDLMTDLLGTPSTETISRIRNDKARKYLSSMRRKQPIPFSEKFPNADPSALKLLERLLAFDPKDRPTAEEALAHPYFKRLARVEREPSCQQPISKTEFEFERRKFTKEDVKELIFREILEYHPKLLKDYMNGSEKTSFLYPSAVDNFRRQFANLEIDGGRSGAADRKHFSLPRTTTVHSAPILPTNGPTSQVPQRIPTARPGRVVSSAMQTDNPSVSDRHNGRRVARDPAVPPAAAYHLKSDYSDRQHQQEFEKDRVRPDRQRQEELEKDRMQYRPGHHSMDAKVAPEISPYMRSSPYYIPPFNGIAAVASGYSKVAAVTRMY
BdMPK21-1	MGGLIRWLRHHRSRRVSSSSSSHLPSNTTSSSSTSDLRAHSLPQHQGDHHGEVVVEWEDAAEGPDSDPEEYIVVVLGDDEQGVVAARAPVRTKPPRVMDPGKKTSESEFFTEYGEANRYKVSEVIGKGSYGVVAAAVDTQTGERCAIKKINDVFDHVSDATRILREIKLLRLLRHPDIVEIKHIMLPPSRREFRDIYVVFELMESDLHQVIKANDDLTPEHHQFFLYQLLRGMKYIHTANVFHRDLKPKNILANADCKLKICDFGLARVSFNDGAPSAIFWTDYVATRWYRAPELCGSFFSKYTPAIDIWSVGCIFAEMLTGKPLFPGKNVVHQLDLMTDLLGTPSAESLSKIRNEKARRYLSNMRKKPKVPLTKKFPGIDPMALHLLERLLAFDPKDRPSADEALTDPYFTGLANSEREPITQPISKLEFEFEKRKLAKDDVRELIYREILEYHPHMLQEYLRGGGDQMSFMFPSGVDRFKRQFAHLEEGGAKGEKSSPQLRQNASLPRERVIGNKHGDGDYNMKLNVGEKPELASVSDGISKPLMSARSLLKSETMSASKCIGEIKNKDEDSLSECVEGTDDDVSQKIAQLKT
BdMPK21-2	MESGRWRKFMSSRREVEKSVIFASTKRGSGRTWRTAYYRKSRDCEGKEKGCEVLMRKEKQENTSRYTRTRGRRGKSGLQGSDEAEFFTEYGEANRYEVGEVVGKGSYGVVAAAVDTHTGERVAIKKINDVFEHVSDATRILREIKLLRLLRHPDIVEIKHIILPPSRREFRDIYIIFELMESDLHQVIKANDDLTPEHHQFFFYQLLRGMKYIHAANVFHRDLKPRNILANADCKLKICDFGLARVSFNDTPSAIFWTDYVATRWYRAPELCGSFFSKYTPAIDIWSIGCIFAEMLSGRPLFPGKNVVHQLDLMTDLLGTPSAESLSRIRNEKARRYLGNMRKKHPVPFSQKFPGVDPMALDLLERLLAFDPKDRPTAAEALADPYFTGLANSDREPTTQPISKLEFEFERRKLARDDVRELIYREILEYHPQMLHEYHHGGDQANFVYPSGVDRFKRQFVHLEEGVTKGEKTSPQLRQHASLPRERIIGIGDELGRPNADYCIKLHVGEEPGHTSVTDGLSKPLLNARNFLKSESISASQCVVIKEKREKDTLLLKTKSFYFVHSDHARADSSVQD
OsMPK2	MRMEGGGGGGHGHHGGGGGGHGHHGGIGGGEAQIKGTLTHGGRYVQYNVYGNLFEVSSKYVPPIRPVGRGACGIICAVVNAQTRQEVAIKKIGNAFDNQIDAKRTLREIKLLRHMDHDNVISIKDIIRPPRRENFNDVYIVYELMDTDLHHLLRSNQPLTDDHCQYFLYQVLRGLKYVHSANVLHRDLRPSNLLLNAKCDLKIGDFGLARTTNETDFMMEYVVTRWYRAPELLLNCSEYTAAIDIWSVGCILGEIVTREPLFPGKDYVHQLRLITELIGSPDDSSLGFLRSDNARRYVRSLPQYPKQQFRARFPTMSSGAMDLLERMLVFDPSKRITVDEALCHPYLASLHEIYDEPVCPAPFSFDFEQPSLTEEDIKEIIWREALKFNPEPIH
OsMPK3	MDGAPVAEFRPTMTHGGRYLLYDIFGNKFEVTNKYQPPIMPIGRGAYGIVCSVMNFETREMVAIKKIANAFNNDMDAKRTLREIKLLRHLDHENIIGIRDVIPPPIPQAFNDVYIATELMDTDLHHIIRSNQELSEEHCQYFLYQILRGLKYIHSANVIHRDLKPSNLLLNANCDLKICDFGLARPSSESDMMTEYVVTRWYRAPELLLNSTDYSAAIDVWSVGCIFMELINRQPLFPGRDHMHQMRLITEVIGTPTDDELGFIRNEDARKYMRHLPQYPRRTFASMFPRVQPAALDLIERMLTFNPLQRITVEEALDHPYLERLHDIADEPICLEPFSFDFEQKALNEDQMKQLIFNEAIEMNPNIRY
OsMPK4	MDSSSGGAGGGGGAQIKGMGTHGGRYVLYNVYGNFFEVSSKYAPPIRPIGRGAYGIVCAAVNSENGEEVAIKKIGNAFDNHIDAKRTLREIKLLRHMDHENIIAIKDIIRPPRRDNFNDVYIVSELMDTDLHQIIRSNQPLTDDHCQYFLYQLLRGLKYVHSANVLHRDLKPSNLFLNANCDLKIADFGLARTTTETDLMTEYVVTRWYRAPELLLNCSQYTAAIDVWSVGCILGEIVTRQPLFPGRDYIQQLKLITELIGSPDDSSLGFLRSDNARRYMKQLPQYPRQDFRLRFRNMSAGAVDLLEKMLVFDPSRRITVDEALHHPYLASLHDINEEPTCPAPFSFDFEQPSFTEEHIKELIWRESLAFNPDPPY
OsMPK6	MDAGAQPPDTEMAEAGGGQQPPAAAAAAGAGAGAGMMENIQATLSHGGRFIQYNIFGNVFEVTAKYKPPILPIGKGAYGIVCSALNSETGEQVAIKKIANAFDNKIDAKRTLREIKLLRHMDHENIVAIRDIIPPPQRNSFNDVYIAYELMDTDLHQIIRSNQALSEEHCQYFLYQILRGLKYIHSANVLHRDLKPSNLLLNANCDLKICDFGLARTTSETDFMTEYVVTRWYRAPELLLNSSEYTAAIDVWSVGCIFMELMDRKPLFPGRDHVHQLRLLMELIGTPNEADLDFVNENARRYIRQLPRHARQSFPEKFPHVHPLAIDLVEKMLTFDPRQRITVEGALAHPYLASLHDISDEPVCSSPFSFDFEQHALSEEQMKDLIYQEGLAFNPDYQ
OsMPK7	MAMMVDPPNGMGNQGKHYYTMWQTLFEIDTKYVPIKPIGRGAYGIVCSSINRATNEKVAIKKINNVFDNRVDALRTLRELKLLRHLRHENVIALKDIMMPVHRRSFKDVYLVYELMDTDLHQIIKSSQPLSNDHCQYFLFQLLRGLKYLHSAGILHRDLKPGNLLVNANCDLKICDFGLARTNNTKGQFMTEYVVTRWYRAPELLLCCDNYGTSIDVWSVGCIFAELLGRKPIFPGTECLNQLKLIVNVLGTMSEADIEFIDNPKARKYIKTLPYTPGIPLTSMYPQAHPLAIDLLQKMLVFDPSKRISVTEALEHPYMSPLYDPSANPPAQVPIDLDIDENLGVDMIREMMW

	QEMLHYHPEVVAGVNM
OsMPK14	MAIMVDPPNGMGNQGKYYYSMWQTLFEIDTKYVPIKPIGRGAYGIVCSSINRETNEKVAIKKIHNVFDNRVDALRTLRELKLLRHLRHENVIALKDIMMPVHRRSFKDVYLVYELMDTDLHQIIKSPQGLSNDHCQYFLFQLLRGLKYLHSAEILHRDLKPGNLLVNANCDLKICDFGLARTNSSKGQFMTEYVVTRWYRAPELLLCCDNYGTSIDVWSVGCIFAELLGRKPIFPGTECLNQLKLIVNVLGTMSESDLEFIDNPKARRYIKSLPYTPGVPLASMYPHAHPLAIDLLQKMLIFDPTKRISVTEALEHPYMSPLYDPSANPPAQVPIDLDIDENISADMIREMMWHEMLHYHPEVVAAMSAR
OsMPK15	MDFFTEYGEGNRYKIEEVIGKGSYGVVCSALDTHTGDKVAIKKINDIFEHVSDATRILREIKLLRLLRHPDIVEIKHILLPPSRREFKDIYVVFELMESDLHQVIKANDDLTPEHYQFFLYQLLRGLKYIHTANVFHRDLKPKNILANADCKLKICDFGLARVAFSDTPTAIFWTDYIATRWYRAPELCGSFFSKYTPAIDIWSIGCIFAELLTGKPLFPGKNVVHQLDIITDLLGTPSPETISRIRNEKARRYLNSMRRKKPIPFTQKFPNADPLAMRLLERMLAFDPKDRPSAEEALADPYFKNIANVDREPSAQPITKLEFEFERRRITKEDIRELIYREILEYHPKMLREFLEGTESTGFMYPSAVDHFKKQFAYLEEHYAKGSTAAPPERQHNSLPRPCVVYSDNRPQSTASVTEDLSRCLIRDNNLKSQDSASVGASRIPQGAAARPGKAVGSVLRYGNCSTSAAEQQYEQRRVVRNPAIAPNSSVPLGSSYPRRNQTCKSETGDVERIDSSQTGPPKPYVANKLPATVDGRSGHW
OsMPK16	MDFFTEYGEGNRYKIEEVIGKGSYGVVCSALDTHTGEKVAIKKINDIFEHVSDATRILREIKLLRLLRHPDIVEIKHILLPPSRREFKDIYVVFELMESDLHQVIKANDDLTPEHYQFFLYQLLRGLKYIHTANVFHRDLKPKNILANADCKLKICDFGLARVAFSDTPTAIFWTDYVATRWYRAPELCGSFFSKYTPAIDIWSIGCIFAELLTGKPLFPGKNVVHQLDIITDLLGTPSTEAISRIRNEKARRYLSSMRRKKPIPFTQKFPNADPLALRLLERMLSFEPKDRPNAEEALADPYFRNIANVDREPSAQPVTKLEFEFERRRITKEDIRELIYRDILEYHPNMLREYLEGTESAGFMYPSAVDHFKKQFAYLEEHYAKGSTAAPPERQHNSLPRPSVLYSDDRPQNTANIAEDLSKCVLGDNTQKMHQGSASVCANRVPQGGAARPGKVVGSALRYGNCSTSTAEQYEHRRTDRNPALATNTVSPRGSYP
OsMPK17-1	MGGGGTLVDGFRRLFHRRTASGSNQSSNAGEEAASSDLEVADDPDLVALRSIRIRVPKRKMPLPVESHKKNTVEMEFFTEYGEASQYQIQEVIGKGSYGVVAAAVDTRTGERVAIKKINDVFEHVSDATRILREIKLLRLLRHPDIVEIKHIMLPPSRREFQDIYVVFELMESDLHQVIRANDDLTPEHYQFFLYQLLRALKYIHAANVFHRDLKPKNILANSDCKLKICDFGLARASFNDAPSAIFWTDYVATRWYRAPELCGSFFSKYTPAIDIWSIGCIFAELLTGRPLFPGKNVVHQLDIITDLLGTPSSETLSRIRNEKARRYLSTMRKKHAVPFSQKFRNTDPLALRLLERLLAFDPKDRSSAEEALADPYFASLANVEREPSRHPISKLEFEFERRKLTKDDVRELIYREILEYHPQMLQEYMKGGEQISFLYPSGVDRFKRQFAHLEENYSKGERGSPLQRKHASLPRERVGVSKDGYNQQNTNDQERSADSVARTTVSPPMSQDAQQHGSAGQNGVTSTDLSSRSYLKSASISASKCVAVKDNKEPEDDYISEEMEGSVDGLSEQVSRMHS
OsMPK17-2	MEFFTEYGEASQYQIQEVVGKGSYGVVAAAVDTHTGERVAIKKINDVFEHVSDAIRILREIKVLRLLRHPDIVVIKHIMLPPTRREFRDIYVVFELMESDLHQVIEANHDLSPEHHRFFLYQLLCALKYIHSANVFHRDLKPKNILANSDCKLKICDFGLARVAFNDSPSTIFWTDYVATRWYRAPELCGSFFSKYTPAIDIWSIGCIFAEILTGRPLFPGRNVVHQLDLITDLLGTPSSETLSRIRNENARGYLTGMQRKHPIPFSHKFHNADPLALRLLERLLAFDPKDRPTAEEALADPYFRGISKLSREPSRLPVSKFEFEFERRKLTKDDVREMIYREILEYHPQMLQEYIRGGEQISFLYPSGVDRFKRQFAHLEENYSRGERSTPLRRQHASLPRERVCSSVDSNNQDSDNEERRAISSIARTMISPPRSQEKGKNRASAYPNGIINLNSNPKIYLKSASISASTCIIRGNKGPKENGISEDMEEVVYELSDNVTRMLS
OsMPK20-1	MQQDQRKKSSTEADFFTEYGDASRYKIQEVIGKGSYGVVCSAIDVHTGEKVAIKKIHDIFEHISDAARILREIKLLRLLRHPDIVEIKHIMLPPSRRDFKDIYVVFELMESDLHQVIKANDDLTKEHYQFFLYQLLRALKYIHTANVYHRDLKPKNILANSNCKLKICDFGLARVAFNDTPTTIFWTDYVATRWYRAPELCGSFFSKYTPAIDIWSIGCIFAEVLTGKPLFPGKNVVHQLDLMTDLLGTPSMDTISRVRNDKARRYLSSMRKKEPILFSQKFPSADPLALDLLQKLLAFDPKDRPTAEEALAHPYFKGLAKVEREPSCQPITKMEFEFERRRVTKEDIRELIFREILEYHPQLLKDYINGTERTTFLYPSAVDQFRKQFAHLEENGGNGPVIPMDRKHTSLPRSTIVHSTPIPAKEQPRIGPSRDKPSDEPYSNPREFDRFSGNAPRTSQAPQRVPTARPGRVVGPVLPYENGATKDSYDARRLAMNSGYPPQQQIPQAYGYYQIPGKSACSELSQAERYTLHQQAYTCANSATVTDVALDMRAPPFHLSGGPKSDSSERLAAETNLYTRSLNGLAATAAGVAASAHRKVGVVPYGMSRMY
OsMPK20-2	MAVKMRIGRRRAIQQGIAEGGFEWRRVWGCREADSRGALWELVWGERSVRERNAAGAAEEVIALFIMDEMCDPASNLEYVVEKAKCDVHRTSSAEEFFTEYGDANRYRIQEVIGKGSYGVVCSAIDLHTRQKVAIKKVHNIFEHVSDAARILREIKLLRLLRHPDIVEIKHIMLPPSRRDFKDIYVVFELMESDLHQVIKANDDLTKEHYQFFLYQLLRALKYIHTASVYHRDLKPKNILANSNCKLKICDFGLARVAFNDTPTTVFWTDYVATRWYRAPELCGSFFSKYTPAIDIWSIGCIFAEVLTGKPLFPGKNVVHQLDLMTDLLGTPSMDTISRVRNEKARRYLSSMRKKDPVPFSQKFPNADPLALKLLQRLLAFDPKDRPTAEEALTDPYFKGLSKIDREPSCQPIRKLEFEFEQKKLSKEDIRELIFQEILEYHPQLQKNYRNGRERATFLYPSAVDQFKKQFSNLEESNGSGSAIPMERKHASLPRSTTVHSTPIPPKEQPLAASLKSSRPVSDEPCKNPWVMGGFSGNIPTSSQVSQVAKPVAPGRPVGSVFPYETGSTNDPYGPRGPVMSSGYPPQQQISQAYGYHQVPARMNCVEQSQAMDAYKMHSQSQTQAYAYPNSKVTADVALDMRGSTFHHSAGSKNGSLDRMVTQTDIYTRSLNGIVAAATSAGVGTNRKVGAVPISTSRMY
OsMPK20-3	MQTSNFRKKNAAEVDFFMGYGDVNRYEVLEVIGKGSYGLVCSANDIHTGEKVAIKKIHNIFEHISDAARILREIKLLRLLRHPDIVEIKHIMLPPSKMDFRDIYVVFELMESDLHQVIKANDDLTREHYQFFLYQMLRALKYIHTANVYHRDLKPKNILANANCKLKICDFGLARVAFTDAPTTVFWTDYVATRWYRAPELCGSFYSKYTPAIDIWSIGCIFAEVLIGKPLFPGKNVVHQLDLITDLLGTPSLDAISQVRNDKARKYLTCMRKKQPASFSHKFLKADPLALQLLRKLLAFDPKDRPSAQEALADPYFNGLAKVEREPSCQPIPKMEFEFERRRATKEDIKELIFQEILEYHPQLLKEHISGTERPNFHHLSVVDQFRKQFTQVEENLNGSGAAVSLQRKHSSLPRSTIVHSAAIPAKDYKHVASSSTKLAVDGSWNAQIQGVHANIAGEPSTIVRPAVSSERSLAPTLQWQPNMTHFLNHALCYQNTVFSGSLLDATGPAQAIPRTTPYVDYRSGNLDLYQHHVSREDVQSDTATAQAHAASHGPVPAVSYSLPGTYRIT
OsMPK20-4	MAMQTMQTEQQQQQRRKGSPEMDFFSEYGDANRYKIQEVIGKGSYGVVCSAIDQHTGDKVAIKKIHNIFEHLSDAARILREIKLLRLLRHPDIVEIKHIMLPPSRRDFKDIYVVFELMDTDLHQVIKANDDLTKEHHQFFLYQMLRALKYIHTANVYHRDLKPKNILANANCKLKICDFGLARVAFNDTPTTVFWTDYVATRWYRAPELCGSFFTKYSPAIDIWSIGCIFAEILTGKPLFPGKNVVHQLDLMTDLLGTPSMDTVTRIRNEKARRYLSSMRKKQPVPFSERFPKADPAALKLLQRLLAFDPKDRPTAEEALADPYFKGLAKAEREPSCQPITKMEFEFERRKVTKEDVKELIFREILEYHPQLLKDYMNGTEKTNFLYPSALDNFRRQFANLEENGGKNGDAVPSDRKHVSLPRTTTVHSAPIPPKDHQNITSQVPQRIPGRTGRGACPVIPFENLSAMGPYNQRRVVRNPVLPPATTNLSAYAYHRKSDSSERELQQELEKDRMRYQPSEHFMDAKVVSHMS

	HDLRASSYYVSKAKSDVADRAALQSNMMQGIGPFNGIAAVGGNYNKVSTVQYGVSRMY
OsMPK20-5	MDFFSEYGDSSRYKIQEIVGKGSYGVVCSAIDQHTGDKVAIKKIHNIFEHLSDAARILREIKLLRLLRHPDIVEIKHIMLPPSRRDFKDIYV<br>VFELMDTDLHQVIKANDDLTKEHHQFFLYQMLRALKYIHTANVYHRDLKPKNILANANCKLKICDFGLARVAFNDTPTTVFWTDYVA<br>TRWYRAPELCGSFFSKYSPAIDTWSIGCIFAEILTGKPLFPGKNVVHQLDLMTDLLGTPSMDAISRIRNDKARRYLSSMRRKQPVPFSEK<br>FPNVDPLALKLLQRLLAFDPKDRPTAEEALADPYFKGLAKVEREPSCQPISKMEFEFERRKVTKDDIKELIFREILEYHPQLLKDYMNGS<br>ENTSFLYPSAVDNFRRQFAILEENGGKSGALDRKHVSLPRATTVHSTSIPPNEGLDATSQVTQRIPTARPGRTVGPVLPFENPGAADPHSA<br>RRVVRNPMVPPAAANKSGYSYNLKSDYSDRQHQEELEKDRVQYRPAQHLMDAKVAPDTAPDIRSSQYYFTRSAPRTDLTDRAALQGS<br>MLYGIAPFNGIAAVAGGYSKVGAVQYGVSRMY
OsMPK21-1	MGGRARSILRWLRHHRSRRVSSSSFHLTTTGDDTVKDLHDPRREDAEGDGWEEVHEGPESDPEEYIALVSEDAGTHLPVRTEPRRMDP<br>SKKEPDFFTEYGEANRYKVSEVIGKGSYGVVAAAVDTQTGERVAIKKINDVFDHVSDATRILREIKLLRLLRHPDIVEIKHIMLPPSRREF<br>RDIYVIFELMESDLHQVIKANDDLTPEHHQFFLYQLLRGMKYIHAASVFHRDLKPKNILANADCKLKVCDFGLARVSFNDTPSAIFWTD<br>YVATRWYRAPELCGSFFSKYTPAIDIWSVGCIFAELLTGKPLFPGKNVVHQLDLMTDLLGTPSAESLAKIRNEKARRYLSNMRKKPRVP<br>FTKKFPGVDPMALHLLERLLAFDPKDRPSAEEALTDPYFNGLANSEREPIAQPISKLEFEFEKRKLAKDDVRELIYREILEYHPHMLQEY<br>LRGGDQMSFMYPSGVDRFKRQFAHLEEGVSKGEKSSPQLRQNASLPRERAIGNKHGDDEYHAKLNVGEKPCHASVTDGISKPLMSAR<br>SLLKSESISASKCIGEKPKQDRDQEDSLTESMDETADEVSEKVAQLKT
OsMPK21-2	MDAKKGSGEPEFFSEYGDASRYEVTEVVGKGSYGVVAAAVDTHTGGRVAIKKINDVFEHISDATRILREIKLLRLLRHPDIVEIKHIMLP<br>PSRREFRDIYIIFELMESDLHQVIKANDDLTPEHHQFFLYQLLRGMKYIHAASVFHRDLKPKNILANADCKVKICDFGLARVSFDDTPSA<br>IFWTDYVATRWYRAPELCGSFFSKYTPAIDIWSVGCIFAEMLMGKPLFPGKNVVHQLDLMTDLLGSPSGETISRIRNEKARRYLGNMRK<br>KPRVPFSQKFPGADPMALHLLERLLAFDPKDRPTAAEALTDPYFTGLANSEREPIAQPISKLEFEFERRKLAKDDVRELIYREILEYHPQ<br>MMQKYLRGGDQSNFLYPSGVDRFKRQFAHLEEGVAQGDKTSPQLRQHVSLPRERVVRNGDEPDPTADYCIKLHVGEQPGHSSVTDGL<br>NKPLLSARNFLKSESIGASQCVVIKEKREKDEESMSEYMNEAADGVPHKIAQLKT
AtMEK1	MNRGSLCPNPICLPPLEQSISKFLTQSGTFKDGDLRVNKDGIQTVSLSEPGAPPPIEPLDNQLSLADLEVIKVIGKGSSGNVQLVKHKLTQ<br>QFFALKVIQLNTEESTCRAISQELRINLSSQCPYLVSCYQSFYHNGLVSIILEFMDGGSLADLLKKVGKVPENMLSAICKRVLRGLCYIHH<br>ERRIIHRDLKPSNLLINHRGEVKITDFGVSKILTSTSSLANSFVGTYPYMSPERISGSLYSNKSDIWSLGLVLLECATGKFPYTPPEHKKGW<br>SSVYELVDAIVENPPPCAPSNLFSPEFCSFISQCVQKDPRDRKSAKELLEHKFVKMFEDSDTNLSAYFTDAGSLIPPLAN
AtMEK2	MKKGGFSNNLKLAIPVAGEQSITKFLTQSGTFKDGDLRVNKDGVRIISQLEPEVLSPIKPADDQLSLSDLDMVKVIGKGSSGVVQLVQH<br>KWTGQFFALKVIQLNIDEAIRKAIAQELKINQSSQCPNLVTSYQSFYDNGAISLILEYMDGGSLADFLKSVKAIPDSYLSAIFRQVLQGLI<br>YLHHDRHIIHRDLKPSNLLINHRGEVKITDFGVSTVMTNTAGLANTFVGTYNYMSPERIVGNKYGNKSDIWSLGLVVLECATGKFPYA<br>PPNQEETWTSVFELMEAIVDQPPPALPSGNFSPELSSFISTCLQKDPNSRSSAKELMEHPFLNKYDYSGINLASYFTDAGSPLATLGNLSG<br>TFSV
AtMEK3	MAALEELKKKLSPLFDAEKGFSSSSSLDPNDSYLLSDGGTVNLLSRSYGVYNFNELGLQKCTSSHVDESESSETTYQCASHEMRVFGAI<br>GSGASSVVQRAIHIPNHRILALKKINIFEREKRQQLLTEIRTLCEAPCHEGLVDFHGAFYSPDSGQISIALEYMNGGSLADILKVTKKIPEP<br>VLSSLFHKLLQGLSYLHGVRHLVHRDIKPANLLINLKGEPKITDFGISAGLENSMAMCATFVGTVTYMSPERIRNDSYSYPADIWSLGL<br>ALFECGTGEFPYIANEGPVNLMLQILDDPSPTPPKQEFSPEFCSFIDACLQKDPDARPTADQLLSHPFITKHEKERVDLATFVQSIFDPTQR<br>LKDLADMLTIHYYSLFDGFDDLWHHAKSLYTETSVFSFSGKHNTGSTEIFSALSDIRNTLTGDLPSEKLVHVVEKLHCKPCGSGGVIIRA<br>VGSFIVGNQFLICGDGVQAEGLPSFKDLGFDVASRRVGRFQEQFVVESGDLIGKYFLAKQELYITNLD
AtMEK4	MRPIQSPPGVSVPVKSRPRRRPDLTLPLPQRDVSLAVPLPLPPTSGGSGGSSGSAPSSGGSASSTNTNSSIEAKNYSDLVRGNRIGSGAGG<br>TVYKVIHRPSSRLYALKVIYGNHEETVRRQICREIEILRDVNHPNVVKCHEMFDQNGEIQVLLEFMDKGSLEGAHVWKEQQLADLSRQ<br>ILSGLAYLHSRHIVHRDIKPSNLLINSAKNVKIADFGVSRILAQTMDPCNSSVGTIAYMSPERINTDLNQGKYDGYAGDIWSLGVSILEFY<br>LGRFPFPVSRQGDWASLMCAICMSQPPEAPATASPEFRHFISCCLQREPGKRRSAMQLLQHPFILRASPSQNRSPQNLHQLLPPPRPLSSS<br>SSPTT
AtMEK5	MKPIQSPSGVASPMKNRLRKRPDLSLPLPHRDVALAVPLPLPPPSSSSSAPASSSAISTNISAAKSLSELERVNRIGSGAGGTVYKVIHTPT<br>SRPFALKVIYGNHEDTVRRQICREIEILRSVDHPNVVKCHDMFDHNGEIQVLLEFMDQGSLEGAHIWQEQELADLSRQILSGLAYLHRR<br>HIVHRDIKPSNLLINSAKNVKIADFGVSRILAQTMDPCNSSVGTIAYMSPERINTDLNHGRYDGYAGDVWSLGVSILEFYLGRFPFAVSR<br>QGDWASLMCAICMSQPPEAPATASQEFRHFVSCCLQSDPPKRWSAQQLLQHPFILKATGGPNLRQMLPPPRPLPSAS
AtMEK6	MVKIKSNLKQLKLSVPAQESPISSFLTASGTFHDGDFLLNQKGLRLTSDEKQSRQSDSKELDFEITAEDLETVKVIGKGSGGVVQLVRHK<br>WVGKFFAMKVIQMNIQEEIRKQIVQELKINQASSQCPHVVVCYHSFYHNGAFSLVLEYMDRGSLADVIRQVKTILEPYLAVVCKQVLL<br>GLVYLHNERHVIHRDIKPSNLLVNHKGEVKISDFGVSASLASSMGQRDTFVGTYNYMSPERISGSTYDYSSDIWSLGMSVLECAIGRFP<br>YLESEDQQNPPSFYELLAAIVENPPPTAPSDQFSPEFCSFVSACIQKDPPARASSLDLLSHPFIKKFEDKDIDLGILVGTLEPPVNYLR
AtMEK7	MALVRKRRQINLRLPVPPLSVHLPWFSFASSTAPVINNGISASDVEKLHVLGRGSSGIVYKVHHKTTGEIYALKSVNGDMSPAFTRQLA<br>REMEILRRTDSPYVVRCQGIFEKPIVGEVSILMEYMDGGNLESLRGAVTEKQLAGFSRQILKGLSYLHSLKIVHRDIKPANLLLNSRNEV<br>KIADFGVSKIITRSLDYCNSYVGTCAYMSPERFDSAAGENSDVYAGDIWSFGVMILELFVGHFPLLPQGQRPDWATLMCVVCFGEPPRA<br>PEGCSDEFRSFVDCCLRKESSERWTASQLLGHPFLRESL
AtMEK8	MVMVRDNQFLNLKLSPIQAPTTIPPCRFPIIPATKVSATVSSCASNTFSVANLDRISVLGSGNGGTVFKVKDKTTSEIYALKKVKENWDS<br>TSLREIEILRMVNSPYVAKCHDIFQNPSGEVSILMDYMDLGSLESLRGVTEKQLALMSRQVLEGKNYLHEHKIVHRDIKPANLLRSSKE<br>EVKIADFGVSKIVVRSLNKCNSFVGTFAYMSPERLDSEADGVTEEDKSNVYAGDIWSFGLTMLEILVGYYPMLPDQAAIVCAVCFGEPP<br>KAPEECSDDLKSFMDCCLRKKASER
AtMEK9	MALVRERRQLNLRLPLPPISDRRFSTSSSSATTTTVAGCNGISACDLEKLNVLGCGNGGIVYKVRHKTTSEIYALKTVNGDMDPIFTRQL<br>MREMEILRRTDSPYVVKCHGIFEKPVVGEVSILMEYMDGGTLESLRGGVTEQKLAGFAKQILKGLSYLHALKIVHRDIKPANLLLNSK

	NEVKIADFGVSKILVRSLDSCNSYVGTCAYMSPERFDSESSGGSSDIYAGDIWSFGLMMLELLVGHFPLLPPGQRPDWATLMCAVCFGEPPRAPEGCSEEFRSFVECCLRKDSSKRWTAPQLLAHPFLREDL
AtMEK10	MTLVRERRHQEPLTLSIPPLIYHGTAFSVASSSSSSPETSPIQTLNDLEKLSVLGQGSGGTVYKTRHRRTKTLYALKVLRPNLNTTVTVEADILKRIESSFIIKCYAVFVSLYDLCFVMELMEKGSLHDALLAQQVFSEPMVSSLANRILQGLRYLQKMGIVHGDIKPSNLLINKKGEVKIADFGASRIVAGGDYGSNGTCAYMSPERVDLEKWGFGGEVGFAGDVWSLGVVVLECYIGRYPLTKVGDKPDWATLFCAICCNEKVDIPVSCSLEFRDFVGRCLEKDWRKRDTVEELLRHSFVKNR
BdMKK1	MRKPGKLALPSHESTIGKFLTQSGTFKDGDLLVNKDGLRIVHNSEEGEAPPIVPLDDHQLSLDDLDAIKVIGKGSSGIVQLVRHKWTDQFFALKVIQLNIQESIRKQIAQELKISLSTQCQYVVTCYQCFYVNGVISIVLEYMDGGSLADFLKTVRTIPEAYLAAICKQVLQGLMYLHHEKRVIHRDLKPSNILINHRGEVKISDFGVSAIIASSSAQRDTFTGTFNYMAPERISGQKHGYMSDIWSLGLVMLECATGNFPYPSPDSFYELLEAVVDQPPPSAPTDQFSPEFCSFISACIQKEATDRSSAQVLSDHPFLSMYDDLNIDLADYFTTAGSPLATFKQIVL
BdMKK3-1	MAGLEELKKKLQPLLFDDPDKDGISTRVPFLEDNCDSYVVSDGGTINLLSRSFGEYNINEHGFHKRSTGADESDFGEKAYRCASHDMHIFGPIGNGASSVVQRAIFIPVHRILALKKINIFEKEKRQQILNEMRTLCEASCYPGLVEFQGAFYMPDSGQISIALEYMDGGSLADVIKVKKSIPEQVLAHMLQKVLLGLRYLHEVRHLVHRDIKPANMLVNLKGEAKITDFGVSAGLDNTMAMCATFVGTVTYMSPERIRNENYSYAADIWSLGLTILECATGKFPYNVNEGPANLMLQILDDPSPTPPADAYSPEFCSFVNDCLQKDPDARPTCEQLFGHPFIKRYENAGVDLIAYVKGVVDPTERLKEIAEMLAVHYYLLFNGSDGLWHHMKTFYMEESTFSFSGNVYVGRNDIFDTLSSIRKKLKGDRPREKIVHVVEKLHCRANGETGIAIRVSGSLIVGNQFLVCGEGLQAEGMPSVEELSIDIPSKRVGQFREQFMMLPGISMGSFHISRQDLYIIQA
BdMKK3-2	MAGLEELKKKLQPLMFNDPDKDGFSTRVPFPEDTCDSYVVSDGGTINLLSRSFGEYNINEHGFHKRSAGADESDFGEKAYRCASQDMHIFGPIGNGASSVVQRAIFIPVHRILALKKINIFEKEKRQQILNEMRTLCEACCYPGLVEFQGAFYMPDSGQISIALEYMDGGSLADVIKVKKSIPEPVLAHMLQKVLLGLRYLHEVRHLVHRDIKPANMLVNLKGEAKITDFGVSAGLDNTMAMCATFVGTVTYMSPERIRNENYSYAADIWSLGLTILECATGKFPYNVNEGPANLMLQILDDPSPAPPENAFSSEFCSFVNDCLQKDADARPTCEQLLSHPFIKRYENAGVDLAAYVKGVVNPEERLKQIAEMLAVHYYLLFNGSDGLWHHMKTFYMEDSTFSFSGNLYVGQSDIFDTLSNIRTKLKGDRPREKIVHVVEKLHCRANEETGIAIRVSGSFIVSNQFLICGEGLQAEGMPSLEELSIDIPSKRVGQFREQFIMHPGRSMGCYYISRQDLYIIQA
BdMKK3-3	MDGGWDGMGQRKRRHRAGIPIPTYPGLAASLEFCFVTAGACHTAPGPGSKIAASLFAALLFQRPPASTSRAIFFCFVIMAAGLEDLRRRVQPIFFDADGNVMPAPDDDSEVLDGGTINLLSRSSDEYNINERGFHKRTIRSDDEYSSEKAFRCSCHDMHIFDSVGNGASSVVHRAIYVPVHRVLALKKINIFEKERRQQILNEIITLSEACCYPGLVEFHGVFYTPDSGEIYFALEYMDGGSLADIIRVKKFISEPVLSHMLQKVLLALRYLHEVRHLVHRDIKPANLLLNLKGDTKITDFGVTSGLHDSIDMCATFLGSVTYMSPERIRNESYSYSADIWSLGLTALECATGRYPYDVNGGEADLMLQKDADARPTCDQLLSHSFIKRYEGPGVDLSEYNKSVHDPSERLSQIAHMLAVHYYLIFDGGDDQWCHMKTFYQQDSIFSFSGETHVGKSEIFETLSRIRKMLKGNSPCEKIAHVMEKVYCRSHGEEGMRVRVSGSFIVGNEFVVCADGVRAEGMLSIDELSPDILSKQAGHFQEDFFMEPGTALGCYVISKQELHIADT
BdMKK4	MRPGGPPNARPQQPGTPGRARRRPDLTLPLPQRDLTSLAVPLPLPPPPSSAPSSASSSGSSLSSMGAPTPPNSAGSAPPPPPPLAELERVRRIGSGAGGTVWMVRHRPTGRPYALKVLYGNHDDAVRRQITREIAILRTAEHPAIVRCHGMYEQAGELQILLEFMDGGSLEGRRIASEAFLADVARQVLSGIAYLHRRHIVHRDIKPSNLLIDSGRRVKIADFGVGRILNQTMDPCNSSVGTIAYMSPERINTDLNDGAYDGYAGDIWSFGLSILEFYLGRFPLGENLGKQGDWAALMCAICYSDSPAPPPIASPEFKSFISCCLQKNPARRPSAAQLLQHRFIAGPQPQVLAAPPS
BdMKK5	MRPAGSLPSPQPGTPGRPRRRPDLTLPMPQRPDVSSSLAVPLPLPPPSSLGLAQPPAAAAAAAAPPPPPLGELERVRRVGSGAGGTVWMVRHRPTGRCYALKQLYGNHDDAVRRQIAREIAILRTAEHPAVVRCHGMYERGGELQILLEYMDGGSLDGRRIAAEGFLADVARQVLSGIAYLHRRHIVHRDIKPSNLLIDSARRVKIADFGVGRILNQTMDPCNSSVGTIAYMSPERINTDLNDGAYDGYAGDIWSFGLSILEFYLGRFPFGENLGKQGDWAALMVAICYNDPPEPSAAASPEFRGFISCCLQKNPAKRLSAAQLLQHPFVAGPQPLPLAAPPS
BdMKK6	MRGKKPLKELKLSVPAQETSVDKFLTASGTFKDGELRLNQRGLRLISEEENGDEHQSTNMKVEDVQLSMDDLEMIQVIGKGSGGVVQLVQHKWVGTFYALKGIQMNIQEAVRKQIVQELKINQATQSPHIVSCHQSFYHNGVIYLVLEYMDRGSLADIIKQVKTILEPYLAVLCKQVLEGLLYLHHERHVIHRDIKPSNLLVNHKGEVKITDFGVSAVLASSIGQRDTFVGTYNYMAPERISGSSYDYKSDVWSLGLVILECAIGRFPYTPSEGEGWLSFYELLEAIVDQPPPGAPADQFSPEFCSFISACIQKDPAERMSASELLNHAFIKKFEGKDLDLRILVESLEPPMNVPE
BdMKK10-1	MALLREKRLQLSLHVPTRAADAQEAGLHRRPNPAAALPLAATTPAARSSQFRVADFEKLAVLGRGNGGTVYKVRHRETCELYALKVQHCNGDATAEAEVLSRTASPFVVRCHSVLPAAASGDVAMLLELVDGGSLDSIVKSRSRGQAEAFSQFPEEALAEVAAQALSGLAYLHARRIVHLDVKPGNLLVSTGGEVKIADFGIARVLPRAGGDDVRCTAYAGTAAYMSPERFDPEAHGGHYDPYAADVWGLGVTVLELLMGRYPLLPAGQRPSWAALMCAICFGETPALSDGEASAELRGFVAACLHKDYRRRASVAELLAHPFVAGRDVAASKCALRKLVTEASMSP
BdMKK10-2	MALVRQRRQLPHLTLPLDHFALRPPPAPAPTVAASTSSEAAGLRLSDFERISLLGQGNGGTVYKARHRRAAAQPPVALKLFVAGDPSAAREAEILRLAADAPHVVRLHAVVPSSSPAAGAEQPPPAALALELLPGGSLAGLLRRLGRSMGERPIAAVARQALLGLDALHALRVVHRDLKPSNLLLGSHGEVKIADFGAGKVLRRRLDPCASYVGTAAYMSPERFDPEAYSGDYDPYAADVWSLGLAILELYLGHFPLLPAGQRPDWAALMCAICFGDAPEAPAAASEEFRDFVARCLEKKAGQRASVAELLEHPFIAERDAEEAKRALAALVAEAELGDL
BdMKK10-3	MALLREKRLQLSLHVPTRAAEALDAVHRRPNPVAATLAASTPAAARSSQFRLADFDKLTVLGRGNGGTVYKVRHRETCELYALKVQHCNGDPTAAAEAEVLSRTASPFIVRCHSVLPGAASGDVAMLLELVDGGSLDSIVKSRRAHAFPFPEEALAEVAAQALSGLAYLHARRIVHLDIKPGNLLVSTGGEVKVADFGIAKVLPRAGADDARCKSYAGTAAYMSPERFDPEAHGGHYDAYAADVWGLGVTVLELLMGRYPLLPAGQRPSWPALMCAICFGETPVLSDGEASAELRGFVAACLRKDHTKRASVAELLAHPFVAGRDVATSKCALRKLVTEASTSP
BdMKK10-4	MASAKERRLPQLHLKLDVPTCAFRCAAPAPAPATAATPATSASRPPHGEFRLNDFDRLSVLGRGNGGSVYKVSHRRTSALYALKIIHGAHARPGAADEEADIVRRVVDSPNVVRCHSVLPTASGDAAALLLELVDGGSLDSLVGGGGFLPEAAVADVAAQALSGLAHLRARRVAHRDIKPANLLLSAAGEVKIADFGIAKVVVSGAGGRARALAYEGTVAYMSPERFDSERHADADPYAADVWGLGVTLLELLMGRYPLLPAGQKPTWAALMCAICFGELPALPEGAASLEFRGFVAACLRKDHRKRASVVELLAHPFVAGRDVAASRRALREAIERRCSC
BdMKK10-5	MALTVRQRRLPQLHISLDLPSCSFRCPNPPVAATASTSGEFRASDFERLAVLGRGNGGTVYKVAHRRTSAQYALKVLHGGGDPGAAAAEADVLRRAADSPYVVRCHSVFPAASGSGETALLLELVDGGSLDSVRRGVGVSVFFPEAALAEVAAQALAGLAHLHARRVVHRDIKPA

	NLLVSGAGGVKVADFGIAMVLPSRAGGERCAAAYEGTVAYMSPERFDSEGRADADPRGADVWGLGVTVLELLMGRYPLLPAGQKPT<br>WAALMCAICFGELPALPEGAASTELRGFIAACLRKDHTKRASVAELIKHPFVAGRNMAASRLALRRLVAGA
OsMKK1	MGKPGKLALPSHESTIGKFLTQSGTFKDGDLLVNKDGLRIVSQSEEGEAPPIEPLDHNQLSLDDLDAIKVIGKGSSGIVQLVRHKWTGQF<br>FALKVIQLNIQENIRRQIAQELKISLSTQCQYVVACCQCFYVNGVISIVLEYMDSGSLSDFLKTVKTIPEPYLAAICKQVLKGLMYLHHE<br>KHIIHRDLKPSNILINHMGEVKISDFGVSAIIASSSAQRDTFTGTYNYMAPERISGQKHGYMSDIWSLGLVMLELATGEFPYPPRESFYEL<br>LEAVVDHPPPSAPSDQFSEEFCSFVSACIQKNASDRSSAQILLNHPFLSMYDDLNIDLASYFTTDGSPLATFNTSNRYDDR
OsMKK3	MAGLEELKKKLQPLLFDDPDKGGVSSRVPLPEDTCDSYVVSDGGTVNLLSRSLGEYNINEHGFHKRSTGPEESDSGEKAYRCASHDM<br>HIFGPIGNGASSVVQRAVFIPVHRILALKKINIFEKEKRQQILNEMRTLCEACCYIGLVEFQGAFYMPDSGQISIALEYMDGGSLANVIKI<br>KKSIPEPVLAHMLQKVLLGLRYLHEVRHLVHRDIKPANLLVNLKGEAKITDFGVSAGLDNTMAMCATFVGTVTYMSPERIRNENYSYA<br>ADIWSLGLAILECATGKFPYNVNEGPANLMLQILDDPSPTPPKDSYSSEFCSFINDCLQKDADARPSCEQLLSHPFIKRYENTTVDLVAYV<br>KSIVDPTERLKQIAEMLAVHYYLLFNGTDGIWHYMKTFYMEESTFSFSGNVYVGQSDIFDTLSNIRKKLKGDCPREKIVHVVEKLHCR<br>AHGETGIAIRVSGSFIVGNQFLICGEGLQAEGMPSLEELSIDIPSKRVGQFREQFIMEPGSSMGCYYILRQDLYIIQA
OsMKK4	MRPGGPPSLRAGLQQQQQQPGTPGRSRRRPDLTLPLPQRDLTSLAVPLPLPLPPSSAPSSTSSSGSSSLGGVPTPPNSVGSAPPAPPPLSE<br>LERVRRIGSGAGGTVWMVRHRPTGRPYALKVLYGNHDDAVRRQITREIAILRTAEHPAVVRCHGMYEQAGELQILLEYMDGGSLEGR<br>RIASEAFLADVARQVLSGIAYLHRRHIVHRDIKPSNLLIDSGRRVKIADFGVGRILNQTMDPCNSSVGTIAYMSPERINTDLNDGAYDGY<br>AGDIWSFGLSILEFYMGRFPLGENLGKQGDWAALMCAICYSDSPAPPPNASPEFKSFISCCLQKNPARRPSAAQLLQHRFVAGPQQQQQ<br>PQPQPLAPPPS
OsMKK5	MRAGDMPGRGARRRPDLTLPMPQRDAPTSLAVPLPLPPAATTTTSAPPAGGAMHPLASAGAAPPPPLEELERVRRVGSGAGGTVWMV<br>RHRGTGKEYALKVLYGNHDDAVRRQIAREIAILRTAEHPAVVRCHDMYERGGELQILLEYMDGGSLDGRRIADERFLADVARQVLSGI<br>AYLHRRHIVHRDIKPSNLLIDSARRVKIADFGVGRILNQTMDPCNSSVGTIAYMSPERINTDLNDGAYDGYAGDIWSFGLSILEFYMGKF<br>PFGENLGKQGDWAALMCAICYSDPPEPPAAVSPEFRSFVGYCLQKNPAKRPSAAQLMQHPFVAGPQPQPLAAPPPSS
OsMKK6	MRGKKPHKELKLSVPAQETPVDKFLTASGTFKDGELRLNQRGLQLISEETADEPQSTNLKVEDVQLSMDDLEMIQVIGKGSGGIVQLV<br>RHKWVGTLYALKGIQMNIQEAVRKQIVQELKINQATQNAHIVLCHQSFYHNGVIYLVLEYMDRGSLADIIKQVKTILEPYLAVLCKQVL<br>EGLLYLHHERHVIHRDIKPSNLLVNRKGEVKITDFGVSAVLASSMGQRDTFVGTYNYMAPERISGSSYDYKSDIWSLGLVILECAIGRFP<br>YIPSEGEGWLSFYELLEAIVDQPPPSAPADQFSPEFCAFISSCIQKDPAERMSASELLNHPFIKKFEDKDLDLRILVESLEPPMNISE
OsMKK10-1	MAKLRERRQLRLSVPASPPPFPHLDHPFAALPSTPPGSPVLAELEMLSVVGRGAGGTVYRARHRRTGAALAVKEMRDDGAALREAGA<br>HLRVAAAAPDHPSVVRLHGVCVGHPVAGNRFVYLVLEYLPEGSLSDVLVRGALPEPAIAGVTRCVLRGLSHLHRLGVAHGDVKPSNLL<br>VGHRGEIKIADFGASRVVTGRDEAHHQSPGTWAYMSPEKLHPEGFGGGGGADFSGDVWSLGVVLLECHAGRFPLVAAGERPDWPALV<br>LAVCFAAAPEVPVAASPEFGGFVRRCLEKDWRRRATVEELLGHPFVAGKPSRCERQNEWRTTFQDKTGQVNTSIYGEE
OsMKK10-2	MALVRQRRHLPHLTLPLDHFALRPPPAPQQQQQPAVAPSTSSDVRLSDFERISVLGHGNGGTVYKARHRRGCPAQQPLALKLFAAGDLS<br>AAREAEILRLAADAPHVVRLHAVVPSAAGGVEEPAALALELMPGGSLAGLLRRLGRPMGERPIAAVARQALLGLEALHALRIVHRDLK<br>PSNLLLGADGEVKIADFGAGKVLRRRLDPCASYVGTAAYMSPERFDPEAYSGDYDPYAADVWSLGVAILELYLGHFPLLPVGQRPDW<br>AALMCAICFGEAPEMPAAASEEFRDFVSRCLEKKAGRRASVGELLEHPFIAERDAADAQRSLAALVAEAEQSGDL
OsMKK10-3	MALIREKRFSQMNLSLHVPSRVPFQDAAAAARRQCPPPVAAASTSSTPASRASQFRLADFERVAVLGRGNGGTVYKVRHRETCALYAL<br>KVQHSAGGGELAGVEADILSRTASPFVVRCHAVLPASASASGDVALLLELVDGGSLASVAARAGAFPEAAVAEVAAQALSGLACLHAR<br>RVVHRDIKPGNLLVSVDGEVKIADFGIAKVVPPRRGGEHRAAYEYEGTAAYMSPERFDSELHGDGADPFAADVWGLGVTVLELLMAR<br>YPLLPAGQKPSWAALMCAICFGELPPLPDGAASPELRAFLAACLHKDHTKRPSAAHLLTHQFVAGRNVAASKLALRRLVAGA
AtMAPKKK1	WRKGQLIGRGAFGTVYMGMNLDSGELLAVKQVLIAANFASKEKTQAHIQELEEEVKLLKNLSHPNIVRYLGTVREDDTLNILLEFVP<br>GGSISSLLEKFGPFPESVVRTYTRQLLLGLEYLHNHAIMHRDIKGANILVDNKGCIKLADFGASKQVAELATMTGAKSMKGTPYWMA<br>PEVILQTGHSFSADIWSVGCTVIEMVTGKAPWSQQYKEVAAIFFIGTTKSHPPIPDTLSSDAKDFLLKCLQEVPNLRPTASELLKHPFV
AtMAPKKK2	WRKGQLIGRGAFGTVYMGMNLDSGELLAVKQVLITSNCASKEKTQAHIQELEEEVKLLKNLSHPNIVRYLGTVREDETLNILLEFVPG<br>GSISSLLEKFGAFPESVVRTYTNQLLLGLEYLHNHAIMHRDIKGANILVDNQGCIKLADFGASKQVAELATISGAKSMKGTPYWMAPE<br>VILQTGHSFSADIWSVGCTVIEMVTGKAPWSQQYKEIAAIFHIGTTKSHPPIPDNISSDANDFLLKCLQQEPNLRPTASELLKHPFV
AtMAPKKK3	WKKGKFLGSGTFGQVYLGFNSEKGKMCAIKEVKVISDDQTSKECLKQLNQEINLLNQLCHPNIVQYYGSELSEETLSVYLEYVSGGSI<br>HKLLKDYGSFTEPVIQNYTRQILAGLAYLHGRNTVHRDIKGANILVDPNGEIKLADFGMAKHVTAFSTMLSFKGSPYWMAPEVVMSQ<br>NGYTHAVDIWSLGCTILEMATSKPPWSQFEGVAAIFKIGNSKDTPEIPDHLSNDAKNFIRLCLQRNPTVRPTASQLLEHPFL
AtMAPKKK4	WKKGRLLGMGSFGHVYLGFNSESGEMCAMKEVTLCSDDPKSRESAQQLGQEISVLSRLRHQNIVQYYGSETVDDKLYIYLEYVSGGS<br>IYKLLQEYGQFGENAIRNYTQQILSGLAYLHAKNTVHRDIKGANILVDPHGRVKVADFGMAKHITAQSGPLSFKGSPYWMAPEVIKNS<br>NGSNLAVDIWSLGCTVLEMATTKPPWSQYEGVPAMFKIGNSKELPDIPDHLSEEGKDFVRKCLQRNPANRPTAAQLLDHAFV
AtMAPKKK5	WKKGKLIGRGTFGSVYVASNSETGALCAMKEVELFPDDPKSAECIKQLEQEIKLLSNLQHPNIVQYFGSETVEDRFFIYLEYVHPGSIN<br>KYIRDHCGTMTESVVRNFTRHILSGLAYLHNKKTVHRDIKGANLLVDASGVVKLADFGMAKHLTGQRADLSLKGSPYWMAPELMQ<br>AVMQKDSNPDLAFAVDIWSLGCTIIEMFTGKPPWSEFEGAAAMFKVMRDSPPIPESMSPEGKDFLRLCFQRNPAERPTASMLLEHRFL
AtMAPKKK6	YMLGDEIGKGAYGRVYIGLDLENGDFVAIKQVSLENIGQEDLNTIMQEIDLLKNLNHKNIVKYLGSLKTKTHLHIILEYVENGSLANIIK<br>PNKFGPFPESLVTVYIAQVLEGLVYLHEQGVIHRDIKGANILTTKEGLVKLADFGVATKLNEADFNTHSVVGTPYWMAPEVIELSGVC<br>AASDIWSVGCTIIELLTCVPPYYDLQPMPALYRIVQDDTPPIPDSLSPDITDFLRLCFKKDSRQRPDAKTLLSHPWI
AtMAPKKK7	YMLGDEIGKGAYGRVYKGLDLENGDFVAIKQVSLENIVQEDLNTIMQEIDLLKNLNHKNIVKYLGSSKTKTHLHIILEYVENGSLANII<br>KPNKFGPFPESLVAVYIAQVLEGLVYLHEQGVIHRDIKGANILTTKEGLVKLADFGVATKLNEADVNTHSVVGTPYWMAPEVIEMSG<br>VCAASDIWSVGCTVIELLTCVPPYYDLQPMPALFRIVQDDNPPIPDSLSPDITDFLRQCFKKDSRQRPDAKTLLSHPWI

AtMAPKKK8	WQKGQLLGRGSFGSVYEGISGDGDFFAVKEVSLLDQGSQAQECIQQLEGEIKLLSQLQHQNIVRYRGTAKDGSNLYIFLELVTQGSLL<br>KLYQRYQLRDSVVSLYTRQILDGLKYLHDKGFIHRDIKCANILVDANGAVKLADFGLAKVSKFNDIKSCKGTPFWMAPEVINRKDSD<br>GYGSPADIWSLGCTVLEMCTGQIPYSDLEPVQALFRIGRGTLPEVPDTLSLDARLFILKCLKVNPEERPTAAELLNHPFV
AtMAPKKK9	WQKGQLLRQGSFGSVYEAISEDGDFFAVKEVSLLDQGSQAQECIQQLEGEIALLSQLEHQNILRYRGTDKDGSNLYIFLELVTQGSLLE<br>LYRRYQIRDSLISLYTKQILDGLKYLHHKGFIHRDIKCATILVDANGTVKLADFGLAKVSKLNDIKSRKETLFWMAPEVINRKDNDGY<br>RSPADIWSLGCTVLEMCTGQIPYSDLEPVEALFRIRRGTLPEVPDTLSLDARHFILKCLKLNPEERPTATELLNHPFV
AtMAPKKK10	WLKGQLLGRGSYASVYEAISEDGDFFAVKEVSLLDKGIQAQECIQQLEGEIALLSQLQHQNIVRYRGTAKDVSKLYIFLELVTQGSVQ<br>KLYERYQLSYTVVSLYTRQILAGLNYLHDKGFVHRDIKCANMLVDANGTVKLADFGLAEASKFNDIMSCKGTLFWMAPEVINRKDS<br>DGNGSPADIWSLGCTVLEMCTGQIPYSDLKPIQAAFKIGRGTLPDVPDTLSLDARHFILTCLKVNPEERPTAAELLHHPFV
AtMAPKKK11	WQKGQLLGRGSLGSVYEGISADGDFFAFKEVSLLDQGSQAHEWIQQVEGGIALLSQLQHQNIVRYRGTTKDESNLYIFLELVTQGSLR<br>KLYQRNQLGDSVVSLYTRQILDGLKYLHDKGFIHRNIKCANVLVDANGTVKLADFGLAKVMSLWRTPYWNWMAPEVIVLKSFPLF
AtMAPKKK12	WRKGELIGCGAFGRVYMGMNLDSGELLAIKQVLIAPSSASKEKTQGHIRELEEEVQLLKNLSHPNIVRYLGTVRESDSLNILMEFVPGG<br>SISSLLEKFGSFPEPVIIMYTKQLLLGLEYLHNNGIMHRDIKGANILVDNKGCIRLADFGASKKVVELATVNGAKSMKGTPYWMAPEVI<br>LQTGHSFSADIWSVGCTVIEMATGKPPWSEQYQQFAAVLHIGRTKAHPPIPEDLSPEAKDFLMKCLHKEPSLRLSATELLQHPFV
AtMAPKKK13	WVRGACIGRGCFGAVSTAISKTNGEVFAVKSVDLATSLPTQSESLENEISVFRSLKPHPYIVKFLGDGVSKEGTTTFRNLYLEYLPNGD<br>VASHRAGGKIEDETLLQRYTACLVSALRHVHSQGFVHCDVKARNILVSQSSMVKLADFGSAFRIHTPRALITPRGSPLWMAPEVIRRE<br>YQGPESDVWSLGCTIIEMFTGKPAWEDHGIDSLSRISFSDELPVFPSKLSEIGRDFLEKCLKRDPNQRWSCDQLLQHPFL
AtMAPKKK14	WIRGSCVGRGCFGTVSKALSKIDGGLFAVKSIDLATCLPSQAESLENEIVILRSMKSHPNIVRFLGDDVSKEGTASFRNLHLEYSPEGDV<br>ANGGIVNETLLRRYVWCLVSALSHVHSNGIVHCDVKSKNVLVFNGGSSVKLADFGSAVEFEKSTIHVSPRGSPLWMAPEVVRREYQG<br>PESDVWSLGCTVIEMLTGKPAWEDHGFDSLSRIGFSNDLPFIPVGLSELGRDFLEKCLKRDRSQRWSCDQLLQHPFL
AtMAPKKK15	WIRGPIIGRGSTATVSLGITNSGDFFAVKSAEFSSSAFLQREQSILSKLSSPYIVKYIGSNVTKENDKLMYNLLMEYVSGGSLHDLIKNSG<br>GKLPEPLIRSYTRQILKGLMYLHDQGIVHCDVKSQNVMIGGEIAKIVDLGCAKTVEENENLEFSGTPAFMSPEVARGEEQSFPADVWA<br>LGCTVIEMATGSSPWPELNDVVAAIYKIGFTGESPVIPVWLSEKGQDFLRKCLRKDPKQRWTVEELLQHPFL
AtMAPKKK16	WTRGPIIGRGSTATVSIAISSSGELFAVKSADLSSSSLLQKEQSILSTLSSPHMVKYIGTGLTRESNGLVYNILMEYVSGGNLHDLIKNSG<br>GKLPEPEIRSYTRQILNGLVYLHERGIVHCDLKSHNVLVEENGVLKIADMGCAKSVDKSEFSGTPAFMAPEVARGEEQRFPADVWAL<br>GCTMIEMMTGSSPWPELNDVVAAMYKIGFSGESPAIPAWISDKAKDFLKNCLKEDQKQRWTVEELLKHPFL
AtMAPKKK17	WTRGRILGRGSTATVYAAAGHNSDEILAVKSSEVHRSEFLQREAKILSSLSSPYVIGYRGSETKRESNGVVMYNLLMEYAPYGTLTDA<br>AAKDGGRVDETRVVKYTRDILKGLEYIHSKGIVHCDVKGSNVVISEKGEAKIADFGCAKRVDPVFESPVMGTPAFMAPEVARGEKQG<br>KESDIWAVGCTMIEMVTGSPPWTKADSREDPVSVLYRVGYSSETPELPCLLAEEAKDFLEKCLKREANERWTATQLLNHPFL
AtMAPKKK18	WTRGKTLGRGSTATVSAATCHESGETLAVKSAEFHRSEFLQREAKILSSLNSPYVIGYRGCEITREPFHNNGEATTYSLLMEYAPYGTL<br>TDVATKNGGFIDEARVVKYTRQILLGLEYIHNSKGIAHCDIKGSNVLVGENGEAKIADFGCAKWVEPEITEPVRGTPAFMAPEAARGE<br>RQGKESDIWAVGCTVIEMVTGSQPWIGADFTDPVSVLYRVGYLGELPELPCSLTEQAKDFLGKCLKKEATERWTASQLLNHPFL
AtMAPKKK19	WIRGETIGYGTFSTVSLATRSNNDSGEFPPLMAVKSADSYGAASLANEKSVLDNLGDDCNEIVRCFGEDRTVENGEEMHNLFLEYASR<br>GSLESYLKKLAGEGVPESTVRRHTGSVLRGLRHIHANGFAHCDLKLGNILLFGDGAVKIADFGLAKRIGDLTALNYGVQIRGTPLYMA<br>PESVNDNEYGSEGDVWALGCVVVEMFSGKTAWSLKEGSNFMSLLLRIGVGDEVPMIPEELSEQGRDFLSKCFVKDPKKRWTAEMLL<br>NHPFV
AtMAPKKK20	WVRGETIGFGTFSTVSTATKSRNSGDFPALIAVKSTDAYGAASLSNEKSVLDSLGDCPEIIRCYGEDSTVENGEEMHNLLLEYASRGSL<br>ASYMKKLGGEGLPESTVRRHTGSVLRGLRHIHAKGFAHCDIKLANILLFNDGSVKIADFGLAMRVDGDLTALRKSVEIRGTPLYMAPE<br>CVNDNEYGSAADVWALGCAVVEMFSGKTAWSVKEGSHFMSLLIRIGVGDELPKIPEMLSEEGKDFLSKCFVKDPAKRWTAEMLLNH<br>SFV
AtMAPKKK21	WIRRETIGHGSFSTVSLATTSGSSSKAFPSLMAVKSSGVVCSAALRNERDVLDDLGDCSEIVRCFGEGRTVENGEEIYNLFLEYASGGS<br>LADRIKSSGEALPEFEVRRFTRSIVKGLCHIHGNGFTHCDIKLENVLVFGDGDVKISDFGLAKRRSGEVCVEIRGTPLYMAPESVNHGEF<br>ESPADIWALGCSVVEMSSGKTAWCLEDGVMNNVMSLLVRIGSGDEVPRIPVELSEEGKDFVSKCFVKNAAERWTAEMLLDHPFL
AtCTR1	LNIKEKIGAGSFGTVHRAEWHGSDVAVKILMEQDFHAERVNEFLREVAIMKRLRHPNIVLFMGAVTQPPNLSIVTEYLSRGSLYRLLH<br>KSGAREQLDERRRLSMAYDVAKGMNYLHNRNPPIVHRDLKSPNLLVDKKYTVKVCDFGLSRLKASTFLSSKSAAGTPEWMAPEVLR<br>DEPSNEKSDVYSFGVILWELATLQQPWGNLNPAQVVAAVGFKCKRLEIPRNLNPQVAAIIEGCWTNEPWKRPSFATIMDLLRPLI
AtEDR1	LVIAERIGLGSYGEVYHADWHGTEVAVKKFLDQDFSGAALAEFRSEVRIMRRLRHPNVVFFLGAVTRPPNLSIVTEFLPRGSLYRILHR<br>PKSHIDERRRIKMALDVAMGMNCLHTSTPTIVHRDLKTPNLLVDNNWNVKVGDFGLSRLKHNTFLSSKSTAGTPEWMAPEVLRNEPS<br>NEKCDVYSFGVILWELATLRLPWRGMNPMQVVGAVGFQNRRLEIPKELDPVVGRIILECWQTDPNLRPSFAQLTEVL
AtRaf3	LQIGERIGIGSYGEVYRAEWNGTEVAVKKFLDQDFSGDALTQFKSEIEIMLRLRHPNVVLFMGAVTRPPNFSILTEFLPRGSLYRLLHRP<br>NHQLDEKRRMRMALDVAKGMNYLHTSHPTVVHRDLKSPNLLVDKNWVVKVCDFGLSRMKHHTYLSSKSTAGTPEWMAPEVLRNE<br>PANEKCDVYSFGVILWELATSRVPWKGLNPMQVVGAVGFQNRRLEIPDDIDLTVAQIIRECWQTEPHLRPSFTQLMQSLKRL
AtRaf4	ITVAERIGLGSYGEVYRGDWHGTAVAVKKFIDQDITGEALEEFRSEVRMMRRLRHPNIVLFMGAVTRPPNLSIVTEFLPRGSLYRLIHR<br>PNNQLDERKRLRMALDAARGMNYLHSCNPVIVHRDLKSPNLLVDKNWVVKVCDFGLSRMKVSTYLSSKSTAGTAEWMAPEVLRNE<br>PADEKCDVYSYGVILWELFTLQQPWGKMNPMQVVGAVGFQHRRLDIPEFVDPGIADIIRKCWQTDPRLRPSFGEIMDSL
AtRaf5	ITVGERIGLGSYGEVYRGDWHGTEVAVKKFLDQDLTGEALEEFRSEVRIMKKLRHPNIVLFMGAVTRPPNLSIVTEFLPRGSLYRLIHR<br>PNNQLDERRRLRMALDAARGMNYLHSCNPMIVHRDLKSPNLLVDKNWVVKVCDFGLSRMKHSTYLSSKSTAGTAEWMAPEVLRN<br>EPADEKCDVYSYGVILWELFTLQQPWGKMNPMQVVGAVGFQHRRLDIPDFVDPAIADLISKCWQTDSKLRPSFAEIMASL

AtRaf6	LHIKERVGAGSFGTVHRAEWHGSDVAVKILSIQDFHDDQFREFLREVCKQAVAIMKRVRHPNVVLFMGAVTERPRLSIITEYLPRGSLFRLIHRPASGELLDQRRRLRMALDVVCAIPHYAKGLNYLHCLNPPVVHWDLKSPNLLVDKNWTVKVCDFGLSRFKANTFIPSKSVAGTPEWMAPEFLRGEPTNEKSDVYSFGVVLWELITLQQPWNGLSPAQVVGAVAFQNRRLIIPPNTSPVLVSLMEACWADEPSQRPAFGSIVDTL
AtRaf7	LTIGEQVGQGSCGTVYHGLWFGSDVAVKVFSKQEYSAEVIESFKQEVLLMKRLRHPNVLLFMGAVTSPQRLCIVSEFLPRGSLFRLLQKSTSKLDWRRRIHMALDIARGMNYLHHCSPPIIHRDLKSSNLLVDKNWTVKVADFGLSRIKHETYLTSKSGKGTPQWMAPEVLRNESADEKSDIYSFGVVLWELATEKIPWETLNSMQVIGAVGFMDQRLEIPKDIDPRWISLMESCWHSDTKLRPTFQELMDKL
AtRaf8	LTIGEQIGRGSCGTVYHGIWFGSDVAVKVFSKQEYSESVIKSFEKEVSLMKRLRHPNVLLFMGAVTSPQRLCIVSEFVPRGSLFRLLQRSMSKLDWRRRINMALDIARGMNYLHCCSPPIIHRDLKSSNLLVDRNWTVKVADFGLSRIKHQTYLTSKSGKGTPQWMAPEVLRNESADEKSDIYSFGVVLWELATEKIPWENLNSMQVIGAVGFMNQRLEIPKDTDPDWISLIESCWHR
AtRaf9	LTIGEQIGQGSCGTVYHGLWFGSDVAVKLISKQEYSEEVIQSFRQEVSLMQRLRHPNVLLFMGAVTLPQGLCIVSEFLPRGSLFRLLQRNMSKLDWRRRINMALDIARGMNYLHRCSPPIIHRDLKSSNLLVDKNLTVKVADFGLSRIKHHTYLTSKSGKGMPQWMAPEVLRNESADEKSDIYSFGVVLWELATEKIPWENLNSMQVIGAVGFMNQRLEIPKDIDPDWISLIESCWHRDAKLRPTFQELMERL
AtRaf10	LTIGEQIGQGSCGTVYHGLWFGSDVAVKVFSKQEYSEEIITSFRQEVSLMKRLRHPNVLLFMGAVTSPQRLCIVTEFLPRGSLFRLLQRNTSKLDWRRRIHMASDIARGMNYLHHCTPPIIHRDLKSSNLLVDKNWTVKVADFGLSRIKHETYLTTKTGRGTPQWMAPEVLRNEAADEKSDVYSFGVILWELVTEKIPWESLNAMQVIGAVGFMNQRLEVPKNVDPQWISLMESCWHSEPQDRPSFQEIMEKL
AtRaf11	LTIGEQIGQGSCGTVYHGLWFGSDVAVKVFSKQEYSEEIITSFKQEVSLMKRLRHPNVLLFMGAVASPQRLCIVTEFLPRGSLFRLLQRNKSKLDLRRRIHMASDIARGMNYLHHCSPPIIHRDLKSSNLLVDRNWTVKVADFGLSRIKHETYLTTNGRGTPQWMAPEVLRNEAADEKSDVYSFGVVLWELVTEKIPWENLNAMQVIGAVGFMNQRLEVPKDVDPQWIALMESCWHSEPQCRPSFQELMDKL
AtRaf12	LQLGEEVGRGSFAAVHRGVWNGSDVAIKVYFDGDYNAMTLTECKKEINIMKKLRHPNVLLFMGAVCTEEKSAIIMEYMPRGSLFKILHNTNQPLDKKRRLRMALDVARGMNYLHRRNPPIVHRDLKSSNLLVDKNWNVKVGDFGLSKWKNATFLSTKSGKGTPQWMAPEVLRSEPSNEKCDVFSFGVILWELMTTLVPWDRLNSIQVVGVVGFMDRRLDLPEGLNPRIASIIQDCWQTDPAKRPSFEELISQM
AtRaf13	LTVGTRVGIGFFGEVFRGVWNGTDVAIKLFLEQDLTAENMEDFCNEISILSRVRHPNVVLFLGACTKPPRLSMITEYMELGSLYYLIHMSGQKKKLSWHRRLRMLRDICRGLMCIHRMKIVHRDLKSANCLVDKHWTVKICDFGLSRIMTDENMKDTSSAGTPEWMAPELIRNRPFTEKCDIFSLGVIMWELSTLRKPWEGVPPEKVVFAVAHEGSRLEIPDGPLSKLIADCWAEPEERPNCEEILRGLL
AtRaf14	LKVGASVGSGTSGVVCRGVWNKTEVAIKIFLGQQLTAENMKVFCNEISILSRLQHPNVILLLGACTKPPQLSLVTEYMSTGSLYDVIRTRKKELSWQRKLKILAEICRGLMYIHKMGIVHRDLTSANCLLNKSIVKICDFGLSRRMTGTAVKDTEAAGTPEWMAPELIRNEPVTEKSDIFSFGVIMWELSTLSKPWKGVPKEKVIHIVANEGARLKIPEGPLQKLIADCWSEPEQRPSCKEILHRLK
AtRaf15	LTVGTRVGIGFFGEVFRGIWNGTDVAIKVFLEQDLTAENMEDFCNEISILSRLRHPNVILFLGACTKPPRLSLITEYMEMGSLYYLLHLSGQKKRLSWRRKLKMLRDICRGLMCIHRMGIVHRDIKSANCLLSNKWTVKICDFGLSRIMTGTTMRDTVSAGTPEWMAPELIRNEPFSEKCDIFSLGVIMWELCTLTRPWEGVPPERVVYAIAYEGARLEIPEGPLGKLIADCWTEPEQRPSCNEILSRLL
AtRaf16	LEDLHELGSGTFGTVYYGKWRGTDVAIKRIKNSCFSGGSSEQARQTKDFWREARILANLHHPNVVAFYGVVPDGPGGTMATVTEYMVNGSLRHVLQRKDRLLDRRKKLMITLDSAFGMEYLHMKNIVHFDLKCDNLLVNLRDPQRPICKVGDFGLSRIKRNTLVSGGVRGTLPWMAPELLNGSSNRVSEKVDVFSFGIVMWEILTGEEPYANLHCGAIIGGIVNNTLRPPVPERCEAEWRKLMEQCWSFDPGVRPSFTEIVERL
AtRaf17	FSNAAMIGKGSFGEIVKAYWRGTPVAVKRILPSLSDDRLVIQDFRHEVDLLVKLRHPNIVQFLGAVTERKPLMLITEYLRGGDLHQYLKEKGGLTPTTAVNFALDIARGMTYLHNEPNVIIHRDLKPRNVLLVNSSADHLKVGDFGLSKLIKVQNSHDVYKMTGETGSYRYMAPEVFKHRRYDKKVDVFSFAMILYEMLEGEPPFANHEPYEAAKHVSDGHRPTFRSKGCTPDLRELIVKCWDADMNQRPSFLDILKRL
AtRaf18	LEELKELGSGTFGTVYHGKWRGSDVAIKRIKKSCFAGRSSEQERLTGEFWGEAEILSKLHHPNVVAFYGVVKDGPGATLATVTEYMVDGSLRHVLVRKDRHLDRRKRLIIAMDAAFGMEYLHAKNIVHFDLKCDNLLVNLKDPSRPICKVGDFGLSKIKRNTLVSGGVRGTLPWMAPELLNGSSSKVSEKVDVFSFGIVLWEILTGEEPYANMHYGAIIGGIVNNTLRPTIPSYCDSDWRILMEECWAPNPTARPSFTEIAGRL
AtRaf19	LFIGNKFASGAHSRIYRGIYKQRAVAVKMVRIPTHKEETRAKLEQQFKSEVALLSRLFHPNIVQFIAACKKPPVYCIITEYMSQGNLRMYLNKKEPYSLSIETVLRLALDISRGMEYLHSQGVIHRDLKSNNLLLNDEMRVKVADFGTSCLETQCREAKGNMGTYRWMAPEMIKEKPYTRKVDVYSFGIVLWELTTALLPFQGMTPVQAAFAVAEKNERPPLPASCQPALAHLIKRCWSENPSKRPDFSNIVAVL
AtRaf20	LEELRELGSGTFGTVYHGKWRGSDVAIKRIKKSCFAGRSSEQERLTGEFWGEAEILSKLHHPNVVAFYGVVKDGPGGTLATVTEYMVDGSLRHVLVRKDRHLDRRKRLIIAMDAAFGMEYLHSKNTVHFDLKCDNLLVNLKDPSRPICKVGDFGLSKIKRNTLVSGGVRGTLPWMAPELLNGSSSKVSEKVDVFSFGIVLWEILTGEEPYANMHYGAIIGGIVNNTLRPTIPGFCDDEWRTLMEECWAPNPMARPSFTEIAGRL
AtRaf21	LKIEKKVASGSYGDLHRGTYCSQEVAIKFLKPDRVNNEMLREFSQEVFIMRKVRHKNVVQFLGACTRSPTLCIVTEFMARGSIYDFLHKQKCAFKLQTLLKVALDVAKGMSYLHQNNIIHRDLKTANLLMDEHGLVKVADFGVARVQIESGVMTAETGTYRWMAPEVIEHKPYNHKADVFSYAIVLWELLTGDIPYAFLTPLQAAVGVVQKGLRPKIPKKTHPKVKGLLERCWHQDPEQRPLFEEIIEML
AtRaf22	LNMGPAFAQGAFGKLYKGTYNGEDVAIKILERPENSPEKAQFMEQQFQQEVSMLANLKHPNIVRFIGACRKPMVWCIVTEYAKGGSVRQFLTRRQNRAVPLKLAVKQALDVARGMAYVHGRNFIHRDLKSDNLLISADKSIKIADFGVARIEVQTEGMTPETGTYRWMAPEMIQHRAYNQKVDVYSFGIVLWELITGLLPFQNMTAVQAAFAVVNRGVRPTVPNDCLPVLSDIMTRCWDANPEVRPCFVEVVKLL
AtRaf23	VRKADGISKGIYQVAKWNGTKVSVKILDKDLYKDSDTINAFKHELTLFEKVRHPNVVQFVGAVTQNVPMMIVSEYHPKGDLGSYLQKKGRLSPAKVLRFALDIARGMNYLHECKPEPVIHCDLKPKNIMLDSGGHLKVAGFGLISFAKLSSDKSKILNHGAHIDPSNYCMAPEVYKDEIFDRSVDSYSFGVVLYEMIEGVQPFHPKPPEEAVKLMCLEGRRPSFKAKSKSCPQEMRELIEECWDTETFVRPTFSEIIVRL

AtRaf24	LEELKELGSGTFGTVYHGKWRGTDVAIKRIKRSCFIGRSSEQERLTSEFWHEAEILSKLHHPNVMAFYGVVKDGPGGTLATVTEYMVNGSLRHVLLSNRHLDRRKRLIIAMDAAFGMEYLHSKSIVHFDLKCDNLLVNLKDPARPICKVGDFGLSKIKRNTLVTGGVRGTLPWMAPELLSGSSSKVSEKVDVFSFGIVLWEILTGEEPYANMHYGAIIGGIVNNTLRPTVPNYCDPEWRMLMEQCWAPDPFVRPAFPEIARRL
AtRaf25	VRKSDGISKGAYQVAKWNGTRVSVKILDKDSYSDPERINAFRHELTLLEKVRHPNVIQFVGAVTQNIPMMIVVEYNPKGDLSVYLQKKGRLSPSKALRFALDIARGMNYLHECKPDPIIHCDLKPKNILLDRGGQLKISGFGMIRLSKISQDKAKVANHKAHIDLSNYYIAPEVYKDEIFDLRVDAHSFGVILYEITEGVPVFHPRPPEEVARMMCLEGKRPVFKTKSRSYPPDIKELIEKCWHPEAGIRPTFSEIIIRL
AtRaf26	LETSNVIARGTYGTVYKGIYDGQDVAVKVLDWEDDGNETTAKTATNRALFRQEVTVWHKLNHPNVTKFVGASMGTTNLNIRSADSKGSLPQQACCVVVEYLPGGTLKQHLIRHKSKKLAFKAVIKLALDLARGLSYLHSEKIVHRDVKTENMLLDAQKNLKIADFGVARVEALNPKDMTGETGTLGYMAPEVIDGKPYNRRCDVYSFGICLWEIYCCDMPYPDLSFVDVSSAVVLHNLRPEIPRCCPTALAGIMKTCWDGNPQKRPEMKEVVKML
AtRaf27	FTQSKEITKGTYCMAMWRGIQVAVKKLDDEVLSDDDQVRKFHDELALLQRLRHPNIVQFLGAVTQSNPMMIVTEYLPRGDLRELLKRKGQLKPATAVRYALDIARGMSYLHEIKGDPIIHRDLEPSNILRDDSGHLKVADFGVSKLVTVKEDKPFTCQDISCRYIAPEVFTSEEYDTKADVFSFALIVQEMIEGRMPFAEKEDSEASEAYAGKHRPLFKAPSKNYPHGLKTLIEECWHEKPAKRPTFREIIKRL
AtRaf28	LHMGPAFAQGAFGKLYRGTYNGEDVAIKLLERSDSNPEKAQALEQQFQQEVSMLAFLKHPNIVRFIGACIKPMVWCIVTEYAKGGSVRQFLTKRQNRAVPLKLAVMQALDVARGMAYVHERNFIHRDLKSDNLLISADRSIKIADFGVARIEVQTEGMTPETGTYRWMAPEMIQHRPYTQKVDVYSFGIVLWELITGLLPFQNMTAVQAAFAVVNRGVRPTVPADCLPVLGEIMTRCWDADPEVRPCFAEIVNLL
AtRaf29	LKIEKKVACGSYGELFRGTYCSQEVAIKILKPERVNAEMLREFSQEVYIMRKVRHKNVVQFIGACTRSPNLCIVTEFMTRGSIYDFLHKHKGVFKIQSLLKVALDVSKGMNYLHQNNIIHRDLKTANLLMDEHEVVKVADFGVARVQTESGVMTAETGTYRWMAPEVIEHKPYDHRADVFSYAIVLWELLTGELPYSYLTPLQAAVGVVQKGLRPKIPKETHPKLTELLEKCWQQDPALRPNFAEIIEML
AtRaf30	LKFGHKIASGSYGDLYKGTYCSQEVAIKVLKPERLDSDLEKEFAQEVFIMRKVRHKNVVQFIGACTKPPHLCIVTEFMPGGSVYDYLHKQKGVFKLPTLFKVAIDICKGMSYLHQNNIIHRDLKAANLLMDENEVVKVADFGVARVKAQTGVMTAETGTYRWMAPEVIEHKPYDHKADVFSYGIVLWELLTGKLPYEYMTPLQAAVGVVQKGLRPTIPKNTHPKLAELLERLWEHDSTQRPDFSEIIEQL
AtRaf31	LFIGSKIGEGAHGKVYQGRYGRQIVAIKVVNRGSKPDQQSSLESRFVREVNMMSRVQHHNLVKFIGACKDPLMVIVTELLPGMSLRKYLTSIRPQLLHLPLALSFALDIARALHCLHANGIIHRDLKPDNLLLTENHKSVKLADFGLAREESVTEMMTAETGTYRWMAPELYSTVTLRQGEKKHYNNKVDVYSFGIVLWELLTNRMPFEGMSNLQAAYAAAFKQERPVMPEGISPSLAFIVQSCWVEDPNMRPSFSQIIRLLNEFL
AtRaf32	LFVGPKIGEGAHAKIYEGKYKNKTVAIKIVKRGESPEEIAKRESRFAREVSMLSRVQHKNLVKFIGACKEPIMVIVTELLLGGTLRKYLVSLRPGSLDIRVAVGYALDIARAMECLHSHGVIHRDLKPESLILTADYKTVKLADFGLAREESLTEMMTAETGTYRWMAPELYSTVTLRHGEKKHYNHKVDAYSFAIVLWELIHNKLPFEGMSNLQAAYAAAFKNVRPSADDLPKDLAMIVTSCWKEDPNDRPNFTEIIQMLLRCL
AtRaf33	LIIKTVLARGTFGTVHRGIYDGQDVAVKLLDWGEEGHRSEAEIVSLRADFAQEVAVWHKLDHPNVTKFIGATMGASGLQLQTESGPLAMPNNICCVVVEYLPGGALKSYLIKNRRRKLTFKIVVQLALDLARGLSYLHSQKIVHRDVKTENMLLDKTRTVKIADFGVARVEASNPNDMTGETGTLGYMAPEVLNGNPYNRKCDVYSFGICLWEIYCCDMPYPDLTFSEVTSAVVRQNLRPDIPRCCPSALAAVMKRCWDANPDKRPEMDEVVPML
AtRaf34	LFVGPKIGEGAHAKVYEGKYKNQTVAIKIVHRGETPEEIAKRDSRFLREVEMLSRVQHKNLVKFIGACKEPVMVIVTELLQGGTLRKYLLNLRPACLETRVAIGFALDIARGMECLHSHGIIHRDLKPENLLLTADHKTVKLADFGLAREESLTEMMTAETGTYRWMAPELYSTVTLRLGEKKHYNHKVDAYSFAIVLWELLHNKLPFEGMSNLQAAYAAAFKNVRPSAESLPEELGDIVTSCWNEDPNARPNFTHIIELL
AtRaf35	LEEIRELGHGTYGSVYHGKWKGSDVAIKRIKASCFAGKPSERERLIEDFWKEALLLSSLHHPNVVSFYGIVRDGPDGSLATVAEFMVNGSLKQFLQKKDRTIDRRKRLIIAMDTAFGMEYLHGKNIVHFDLKCENLLVNMRDPQRPICKIGDLGLSKVKQKTLVSGGVRGTLPWMAPELLSGKSNMVSEKIDVYSFGIVMWELLTGEEPYADMHCASIIGGIVNNALRPKIPQWCDPEWKGLMESCWTSEPTERPSFTEISQKL
AtRaf36	LFFGLKFAHGLYSRLYHGKYEDKAVAVKLITVPDDDDNGCLGARLEKQFTKEVTLLSRLTHPNVIKFVGAYKDPPVYCVLTQYLPEGSLRSFLHKPENRSLPLKKLIEFAIDIARGMEYIHSRRIIHRDLKPENVLIDEEFHLKIADFGIACEEEYCDMLADDPGTYRWMAPEMIKRKPHGRKADVYSFGLVLWEMVAGAIPYEDMNPIQAAFAVVHKNIRPAIPGDCPVAMKALIEQCWSVAPDKRPEFWQIVKVL
AtRaf37	ISIGDFIGEGSSSTVYRGLFRRVVPVSVKIFQPKRTSALSIEQRKKFQREVLLLSKFRHENIVRFIGACIEPKLMIITELMEGNTLQKFMLSVRPKPLDLKLSISFALDIARGMEFLNANGIIHRDLKPSNMLLTGDQKHVKLADFGLAREETKGFMTFEAGTYRWMAPELFSYDTLEIGEKKHYDHKVDVYSFAIVFWELLTNKTPFKGKNNIFVAYAASKNQRPSVENLPEGVVSILQSCWAENPDARPEFKEITYSLTNLLRSL
AtRaf38	LIIKSVIARGTFGTVHRGIYDGQDVAVKLLDWGEEGHRSDAEIASLRAAFTQEVAVWHKLDHPNVTKFIGAAMGTSEMSIQTENGQMGMPSNVCCVVVEYCPGGALKSFLIKTRRRKLAFKVVIQLSLDLARGLSYLHSQKIVHRDVKTENMLLDKSRTLKIADFGVARLEASNPNDMTGETGTLGYMAPEVLNGSPYNRKCDVYSFGICLWEIYCCDMPYPDLSFSEVTSAVVRQNLRPEIPRCCPSSLANVMKRCWDANPEKRPEMEEVVAML
AtRaf39	LEMRNVIARGAYGIVYKGIYDGQDVAVKVLDWGEDGYATTAETSALRASFRQEVAVWHKLDHPNVTRFVGASMGTANLKIPSSAETENSLPQRACCVVVEYIPGGTLKQYLFRNRRKKLAFKVVVQLALDLSRGLSYLHSERIVHRDVKTENMLLDYQRNLKIADFGVARVEAQNPKDMTGETGTLGYMAPEVLDGKPYNRRCDVYSFGICLWEIYCCDMPYPDLSFADVSSAVVRQNLRPDIPRCCPTALATIMKRCWEANPEKRPEMEEVVSLLEAV
AtRaf40	LEDLTELGSGTYGTVYHGTWRGTDVAIKRIRNSCFAGRSSEQERLTKDFWREAQILSNLHHPNVVAFYGIVPDGTGGTLATVTEFMVNGSLRHALLKKDRLLDTRKKIIIAMDAAFGMEYLHSKNIVHFDLKCENLLVNLRDPQRPICKVGDLGLSRIKRNTLVSGGVRGTLPWMAPELLNGSSTRVSEKVDVFSYGISLWEILTGEEPYADMHCGAIIGGIVKNTLRPPIPKSCSPEWKKLMEQCWSVDPDSRPPFTEITCRL
AtRaf41	LFVGPKIGEGAHAKVYEGKYRNQTVAIKIIKRGESPEEIAKRDNRFAREIAMLSKVQHKNLVKFIGACKEPMMVIVTELLLGGTLRKY

	LVSLRPKRLDIRLAVGFALDIARAMECLHSHGIIHRDLKPENLILSADHKTVKLADFGLAREESLTEMMTAETGTYRWMAPELYSTVT<br>LRQGEKKHYNHKVDAYSFAIVLWELILNKLPFEGMSNLQAAYAAAFKNLRPSAEDLPGDLEMIVTSCWKEDPNERPNFTEIIQMLLRY<br>L
AtRaf42	LEELRELGSGTFGTVYHGKWRGTDVAIKRINDRCFAGKPSEQERMIDDFWNEAQNLAGLHHPNVVAFYGVVLDSPGGSVATVTEYM<br>VNGSLRNALQKNVRNFDRCKRQLIAMDIAFGMEYLHGKKIVHFDLKSDNLLVNLRDPHRPICKVGDLGLSKVKCQTLISGGVRGTLP<br>WMAPELLNGTSSLVSEKVDVFSFGIVLWELFTGEEPYADLHYGAIIGGIVSNTLRPQIPDFCDMDWKLLMERCWSAEPSERPSFTEIVN<br>EL
AtRaf43	LSYGDRFAHGKYSQIYHGEYEGKAVALKIITAPEDSDDIFLGARLEKEFIVEATLLSRLSHPNVVKFVGVNTGNCIITEYVPRGSLRSYL<br>HKLEQKSLPLEQLIDFGLDIAKGMEYIHSREIVHQDLKPENVLIDNDFHLKIADFGIACEEEYCDVLGDNIGTYRWMAPEVLKRIPHGR<br>KCDVYSFGLLLWEMVAGALPYEEMKFAEQIAYAVIYKKIRPVIPTDCPAAMKELIERCWSSQTDKRPEFWQIVKVL
AtRaf44	IMRGEMIGEGGNSIVYKGRLKNIVPVAVKIVQPGKTSAVSIQDKQQFQKEVLVLSSMKHENIVRFVGACIEPQLMIVTELVRGGTLQRF<br>MLNSRPSPLDLKVSLSFALDISRAMEYLHSKGIIHRDLNPRNVLVTGDMKHVKLADFGLAREKTLGGMTCEAGTYRWMAPEVCSREP<br>LRIGEKKHYDQKIDVYSFALIFWSLLTNKTPFSEIPSISIPYFVNQGKRPSLSNIPDEVVPILECCWAADSKTRLEFKDITISL
AtRaf45	VVVGEMIGEGAYSIVYKGLLRNQFPVAVKIMDPSTTSAVTKAHKKTFQKEVLLLSKMKHDNIVKFVGACIEPQLIIVTELVEGGTLQR<br>FMHSRPGPLDLKMSLSFALDISRAMEFVHSNGIIHRDLNPRNLLVTGDLKHVKLADFGIAREETRGGMTCEAGTSKWMAPEVYSPEPL<br>RVGEKKEYDHKADIYSFAIVLWQLVTNEEPFPDVPNSLFVPYLVSQGRRPILTKTPDVFVPIVESCWAQDPDARPEFKEISVML
AtRaf46	VRKVDGISKGTYQVAKWNGTRVSVKIFDKDSYSDPERVNAFTNELTLLAKARHPNIVQFVGAVTQNLPMMIVVECNPKGDLSVYLQ<br>KKGRLSPSKALRFALDIARGMNYLHECKPDPIIHCELMPKNILLDRGGQLKISGFGLIKLSKIGEDSAKVVNHEAQIDKSNYYIAPEIYK<br>DEVFDKRADVHSFGVILYELTEGVSLFHPKPPEEVAESICIEGKRPTIRTKSKSYPPELKELIEECWHPEISVRPIFSEIIIRL
AtRaf47	FSNSVKISKGTFNKASWRGIDVAVKTFGEEMFTDEDKVNAFRDELALLQKIRHPNVVQFLGAVTQSTPMMIVTEYLPKGDLRQYLDR<br>KGPLMPAHAVKFALEIARGMNYLHEHKPEAIIHCDLEPPNILRDDSGHLKVADFGVSKLLVVKKTVKKDRPVVTCLDSSWRYMAPEV<br>YRNEEYDTKVDVFSFALILQEMIEGCEPFHEIEDREVPKAYIEDERPPFNAPTKSYPFGLQELIQDCWDKEASKRPTFRVIISTLELI
AtRaf48	LDMKHVLAHGTYGTVYRGVYAGQEVAVKVLDWGEDGYATPAETTALRASFEQEVAVWQKLDHPNVTKFIGASMGTSDLRIPPAGD<br>TGGRGNGAHPARACCVVVEYVAGGTLKKFLIKKYRAKLPIKDVIQLALDLARGLSYLHSKAIVHRDVKSENMLLQPNKTLKIADFGV<br>ARVEAQNPQDMTGETGTLGYMAPEVLEGKPYNRKCDVYSFGVCLWEIYCCDMPYADCSFAEISHAVVHRNLRPEIPKCCPHAVANI<br>MKRCWDPNPDRRPEMEEVVKLLEAI
AtZIK1	GRFREVLGKGAMKTVYKAFDQVLGMEVAWNQVKLNEVFRSPEPLQRLYSEVHLLKNLNHESIIRYCTSWIDVNRRTFNFITELFTSGT<br>LREYRRKYQKVDIRAIKSWARQILNGLAYLHGHDPPVIHRDLKCDNIFVNGHLGQVKIGDLGLAAILRGSQNAHSVIGTPEFMAPELY<br>EEDYNELVDIYSFGMCVLEMLTGEYPYSECTNPAQIYKKVTSGKLPDSFHLIQHTEAQRFVGKCLETVSRRLPAKELLADPFL
AtZIK2	GRFAEILGRGAMKTVYKAIDEKLGIEVAWSQVKLKEVLRSSVDLQRLYSEVHLLSTLNHKSIIRFYTSWIDVHNHTLNFITELFTSGTLR<br>QYKNKYLRIDIRAIKSWARQILEGLVYLHEHDPPVIHRDLKCDNIFVNGHLGQVKIGDLGLARMLRDCHSAHSIIGTPEFMAPELYEEN<br>YNELIDVYSFGMCFLEMITSEFPYSECNHPAQIYKKVVGGKLPGAFYRVGDIEAQRFIGKCLVSASKRVSAKELLQDPFL
AtZIK3	GRYDEILGKGASKTVYRAFDEYEGIEVAWNQVKLRNFTRNPEELEKFFREIHLLKTLNHQNIMKFYTSWVDTNNLSINFVTELFTSGTL<br>RQYRLRHRRVNIRAVKQWCKQILKGLLYLHSRSPPIIHRDLKCDNIFINGNQGEVKIGDLGLAAILRKSHAVRCVGTPEFMAPEVYDEE<br>YNELVDVYAFGMCVLEMVTFDYPYSECTHPAQIYKKVTSGKKPEAFYLVKDPEVREFVEKCLANVTCRLTALELLQDPFL
AtZIK4	GRYNEVLGKGASKTVYRAFDEYEGIEVAWNQVKLYDFLQSPEDLERLYCEIHLLKTLKHKNIMKFYTSWVDTANRNINFVTELFTSG<br>TLRQYRLRHKRVNIRAMKHWCRQILRGLHYLHSHDPPVIHRDLKCDNIFVNGNQGEVKIGDLGLAAILRKSHAAHCVGTPEFMAPEV<br>YEEAYNELVDIYSFGMCILEMVTFDYPYSECTHPAQIYKKVMSGKKPDALYKVKDPEVKCFIEKCLATVSLRVSARELLDDPFL
AtZIK5	IRYKEVIGKGAFKTVYKAFDEVDGIEVAWNQVRIDDVLQSPNCLERLYSEVRLLKSLKHNNIIRFYNSWIDDKNKTVNIITELFTSGSLR<br>HYRKKHRKVNMKAVKNWARQILMGLRYLHGQEPPIIHRDLKCDNIFINGNHGEVKIGDLGLATVMEQANAKSVIGTPEFMAPELYD<br>ENYNELADIYSFGMCMLEMVTFDYPYCECKNSAQIYKKVSSGIKPASLSRVKDPEVKQFIEKCLLPASERLSAKELLLDPFL
AtZIK6	IRYDDVLGRGAFKTVYKAFDEVDGIEVAWNLVSIEDVMQMPGQLERLYSEVHLLKALKHENIIKLFYSWVDEKNKTINMITELFTSGS<br>LRVYRKKHRKVDPKAIKNWARQILKGLNYLHSQNPPVIHRDLKCDNIFVNGNTGEVKIGDLGLATVLQQPTARSVIGTPEFMAPELYE<br>EEYNELVDIYSFGMCMLEMVTCEYPYNECRNQAQIYKKVTSNIKPQSLGKVDDPQVRQFIEKCLLPASSRPTALELSKDPFL
AtZIK7	TQLVDIFGFKGFDEVDGIEVAWNQVRIDDLLQSPDCLERLYSEVRLLKSLKHKNIIRFYNSWIDDKNKTVNIITELFTSGSLRQYRKKHR<br>KVNMKAVKCWARQILTGLKYLHSQDPPIIHRDIKCDNIFINGNHGEVKIGDLGLATVMEQANAKSVIGTPEFMAPELYDENYNELADI<br>YSFGMCMLEMVTFEYPYCECRNSAQIYKKVSSGIKPASLSKVKDPEVMKFIEKCLLPASERLSAEELLLDSFL
AtZIK8	GRYGELLGSGAVKKVYRAFDQEEGIEVAWNQVKLRCFSDDPAMTERLYSEVRLLKNLKNSNIITLYKVWRDERNNTLNFITEICTSGN<br>LREYRKKHRHVSMRALKKWSKQILKGLDYLHTHDPCIIHRDLNCSNIFVNGNIGQVKIGDLGLAAIVGKNHLAHSILGTPEFMAPELY<br>EENYTEMVDIYSYGMCVLELVSLEIPYSECDSVAKIYKRVSKGLKPEALNKVNDPEAKAFIEKCIAQPRARPSAAELLCDPFF
AtZIK9	MKFYASWVDTDNRNINFVTEMFTSGTLRQYRLKHKRVNIRAVKNWCRQILRGLNYLHTHDPPVIHRDLKCDNIFINGNQGEVKIGDL<br>GLAACLQHSHAAHCVGTPEFMAPEVYKEEYNQLVDIYSFGMCVLEMVTFDYPYSECSHPAQIYKRVISGKKPDGLDKVKDPEVRGFI<br>EKCLATVSLRLSACELLDDH
AtZIK10	IRYNDVLGRGAFKTVYKAFDEVEGIEVAWNLMSIEDVLQMPGQLDRLYSEVHLLNSLKHDNIIKLFYSWVDDHNKSINMITELFTSGS<br>LTLYRKKHRKVDPKAIMNWARQILKGLHYLHSQTPPVIHRDLKCDNIFVNGNTGKVKIGDLGLAAVMQQPTARSVIGTPEFMAPELY<br>EEEYNELVDIYSFGMCMLEMVTCEYPYRECRNQAQIYKKVTSGIKPQSLSKVDDPQVKQFIEKCLLPAPSRPTALELLKDQLL
AtZIK11	GRYKEVLGKGAFKEVYRAFDQLEGIEVAWNQVKLDDKFCSSEDLDRLYSEVHLLKTLKHKSIIKFYTSWIDHQHMTINLITEVFTSGN<br>LRQYRKKHKCVDLRALKKWSRQILEGLVYLHSHDPPVIHRDLKCDNIFINGNQGEVKIGDLGLAAILHRARSAHSVIGTPEFMAPELY

	EEDYNVLVDIYAFGMCLLELVTFEYPYSECTNAAQIYRKVTSGIKPAALLNVTDPQVRAFIEKCIAKVSQRLSAKELLDDPFL
BdMAPKKK1	YMLGDEIGKGAYGRVYKGLDLENGDFVAIKQVSLENIPQEDLNIIMQEIDLLKNLNHKNIVKYLGSLKTKSHLHIILEYVENGSLANIIK<br>PNKFGPFPESLAAVYIAQVLEGLVYLHEQGVIHRDIKGANILTTKEGLVKLADFGVATKLTEADVNTHSVVGTPYWMAPEVIEMSGV<br>CAASDIWSVGCTVIELLTCVPPYYELQPMPALFRIVQDVQPPIPEGFSPEINDFLRQCFQKDAIQRPDAKTLLMHPWL
BdMAPKKK2	LEELRELGSGTFGTVYHGKWRGSDVAIKRIKKSCFTGRSSELERLANEFWREAEILSKLHHPNVVAFYGVVKDGPGGTLATVTEFMV<br>NGSLRHVLQRNKNLDRRKRLIIAMDAAFGLEYLHSKNIVHFDLKCDNLLVNLKDQSRPICKVGDFGLSKIKRNTLVSGGVRGTLPWM<br>APELLNGGSNKVSEKVDVFSFGIVMWEILTGEEPYANMHYGAIIGGIVNNTLRPHVPASCDPEWRRLMEQCWAPDPAQRPAFTEIAGR<br>L
BdMAPKKK3	LEEIRELGSGTYGAVFHGKWRGCDVAIKRIKASCFAGRPSERERLIADFWKEAQILSSLHHPNVVSFYGVVRDGPDGSLATVTEFMING<br>SLKQFLRKKDRTIDRRKRVILAMDAAFGMEYLHGKNIVHFDLKCENLLVNMRDPQRPICKIGDLGLSKVKQHTLVSGGVRGTLPWM<br>APELLSGKSNMVSDKIDVYSFGIVMWELLTGDEPYSDMRAAEIIGGIVNNSLRPQIPSWCDPEWKSLMEGSWAGEPAERPSFTEISQRL
BdMAPKKK4	LEELRELGSGTFGTVYHGKWRGSDVAIKRINDRCFAGKASEEQRMKTDFWNEARKLASLHHPNVVAFYGVVLDGPGGSVATVTEY<br>MANGSLRQALQRHDKIFDRRRRLVIVMDVAFGMEYLHGKNIVHFDLKSDNLLVNLRDPQRPICKVGDLGLSKVKCQTLISGGVRGTL<br>PWMAPELLNGSSSLVSEKVDVFSFGIVMWELLTGEEPYADLHYGAIIGGIVNNTLRPEVPESCDPQWRSLMEQCWSAEPSERPSFTEV<br>VKRL
BdMAPKKK5	IAIGERIGLGSFGEVYRGEWHGTEVAVKKFLQQDISSDALDEFRAEVRIMKRLRHPNVVLFMGAITRVPNLSIVTEFLPRGSLFRLIHRP<br>NNQLDEKRRLRMALDVARGMNYLHNCTPVIVHRDLKSPNLLVDKNWVVKVCDFGLSRMKNNTFLSSRSTAGTAEWMAPEVLRNEP<br>SDEKCDVFSYGVILWELCTLQQPWEGMNAMQVVGAVGFQSRRLDIPDNTDPAVAEIITQCWQTDPRKRPSFADIMAAL
BdMAPKKK6	VIGERIGLGSYGEVYRADWNGTEVAVKKFLDQDFYGDALDEFRSEVRIMRRLRHPNIVLFMGAVTRPPNLSIVSEYLPRGSLYKILHRP<br>SCQIDEKRRIKMAIDVAKGMNCLHTSVPTIVHRDLKSPNLLVDNNWTVKVCDFGLSRLKHSTFLSSKSTAGTPEWMAPEVLRNEQSN<br>EKCDVYSFGVILWELATLRMPWSGMNPMQVVGAVGFQDRRLDIPKEVDPLVARIIWECWQKDPNLRPSFAQLTSAL
BdMAPKKK7	ITLGERVGLGSFGEVYRGEWHGTEVAVKKFLQQDISSDILEELKAEVRIMKRLRHPNVVLFMGAVTRVPNLSILTEFLPRGSLFRLIRRP<br>NNQLDERKRIRMALDVARGMNYLHNCTPVVVHRDLKSPNLLVDKNWVVKVCDFGLSRIKHSTFLSSRSTAGTAEWMAPEVLRNEPS<br>DEKCDVFSYGVILWELCTLLQPWEGMNPMQVVGAVGFQQRRLDIPADVDPAVAEIIQRCWQTDPKMRPSFSEIMAAL
BdMAPKKK8	WKKGKLIGRGTFGHVYVGFNSDSGEMCAMKEVTLFLDDSKSKESAKQLGQEISLLSRLQHPNIVRYYGSETVDDKLYIYLEYVSGGSI<br>HKLLQEYGQLGEPAMRSYTQQILSGLAYLHAKNTVHRDIKGANILVDPSGRVKLADFGMAKHINGHQCPFSFKGSPYWMAPEVIKSS<br>NGGCNLAVDIWSLGCTVLEMATSKPPWSQYEGIAAMFKIGNSKELPPIPDHLSEQGKDFIRKCLQRDPSQRPTAMELLQHPFI
BdMAPKKK9	LEELQELGSGTFGTVYHGKWRGSDVAIKRINDRCFAGKPSEQEKMRNDFWNEASNLADLHHPNVVAFYGVVLDGPGGSIATVTEYM<br>VNGSLRTALLKNAKSLDRRKRLIIAMDTAFGMEYLHNKNIVHFDLKSDNLLVNLRDPQRPICKVGDLGLSKVKCQTLISGGVRGTLP<br>WMAPELLNGSSSLVSEKVDVFSFGIVLWELLTGEEPYADLHYGVIIGGIVSNTLRPPVPDSCDLEWKSLMEQCWATEPSERPSFTQIAV<br>RL
BdMAPKKK10	LTIGTRVGIGFFGEVFRGIWNGTDVAIKVFLEQDLTTENMEDFCNEIYILSRLRHPNVILFLGACMVPPHLSMVTEYMEMGSLYYLIHM<br>SGQKKKLSWRRRLKIIRDICRGLMCIHRMKIVHRDLKSANCLVNKHWTVKICDFGLSRAMTDSPMTDNSSAGTPEWMAPELIRNEPFS<br>EKCDIFSLGVIMWELCTLSRPWDGISPVQVVYAVANEGSRLEIPEGPLGKLIADCWAEPQDRPSCQEILTRL
BdMAPKKK11	WKKGKLIGHGTFGHVYVGFNSDRGEMCAMKEVTLFSDDPKSKESARQLGQEILVLSRLQHPNIVRYYGSETVDNKLYIYLEYVSGGS<br>IHKLLQEYGRFGEQAIRSYTKQIRLGLAYLHAKNTVHRDIKGANILVDPNGRVKLADFGMAKHINGQQCPFSFKGSPYWMAPEVIKNS<br>TGCNLAVDVWSLGCTVLEMATSKPPWSQYEGIAAMFKIGNSKELPPIPDHLSEEGKDFIRQCLQRDPSSRPTAVDLLQHSFI
BdMAPKKK12	LVIDERIGIGSYGEVYHADWNGTEVAVKKFLDQEFYGDALDEFRCEVRIMRRLRHPNIVLFMGAVTRPPHLSIVSEYLPRGSLYKIIHR<br>PNCQIDEKRRIRMALDVARGMNCLHTSVPTIVHRDLKSPNLLVDDNWTVKVCDFGLSRLKHSTFLSSRSTAGTPEWMAPEVLRNEQS<br>NEKCDIYSFGVILWELATLRKPWQGMNQMQVVGAVGFQDRRLDIPKEVDPIVASIIRDCWQKDPNLRPSFSQLTSYL
BdMAPKKK13	ITVGTRVGIGFFGEVFRGVWNGTDVAIKVFLEQDLTMENMEDFCNEISILSRLRHPNVILFLGACMKPPHLSLVTEYMEMGSLYYLIHT<br>SGNKGKLSWRRKLKMLRDICRGLMCMHRLKIVHRDLKSANCLVNKYWTVKLCDFGLSRVMLDSAMRDNSSAGTPEWMAPELIRNE<br>PFTEKCDIFSLGVIMWELCTLSRPWAGKPPVQVVYSVANEGARLEIPDGPLRSLISDCWAEPDKRPSCQEILTRL
BdMAPKKK14	LQIGERIGLGSYGEVYHADWNGTEVAVKKFLDQDLSGVALEQFKCEVRIMSRLRHPNVVLFLGYVTQPPNLSILTEYLPRGSLFRLLH<br>RPNSKVDETRRLKMALDVAKGMNYLHASHPTIVHRDLKSPNLLVDKNWVVKVSDFGMSRLKHHTFLSSKSTAGTPEWMAPEVLRN<br>EPANEMCDVYSFGVILWELATMRVPWSGLNPMQVVGAVGFQNRRLDIPKEVDPVVASIILSCWDNDPSKRPSFSQLLSPL
BdMAPKKK15	LVIGEQVGQGSCGTVYHALWYGSDVGVKVFSRQEYSEEVIQAFRQEVSLMKKLRHPNILLFMGAVTSPHRLCIVTEFLPRGSLFRLLQ<br>RSTTKLDWRRRVHMALDVARGMNYLHHYSPPIIHRDLKSSNLLVDKNWTVKVADFGLSRLKRETYLTTKTGKGTPQWMAPEVLRN<br>EPSDEKSDVYSYGVILWELVTQKIPWENLNSMQVIGAVGFMNQRLEIPSETDPYWTSLILSCWETDPQSRPSFQELLEKL
BdMAPKKK16	GRYNDVLGKGASKTVYRAFDEYQGMEVAWNQVKLHDFLQSPEDLERLYCEIHLLKTLKHRNIMKFYTSWVDVSGRNINFITEMFTS<br>GTLRQYRQRHRKVNIWAVKHWCRQILSGLLYLHSHDPPIIHRDLKCDNIFVNGNQGEVKIGDLGLAAILRKSHAVHCVGTPEFMAPE<br>VYEEEYNELVDIYSFGMCVLEMVTFEYPYSECTHPVQIYKKVISGTKPEALYKVKDPMVRQFVEKCLTTASRRLPARELLNDP
BdMAPKKK17	LVLKEKIGAGSFGTVHRADWHGSDVAVKILMEQDYHLDRFREFMREVAIMKSLRHPNIVLFMGAVTEPPNLSIVTEYLSRGSLYKLLH<br>RSGAREVLDERRRLNMAFDVAKGMNYLHRRSPPIVHRDLKSPNLLVDKKYTVKVCDFGLSRLKANTFLSSKSLAGTPEWMAPEVLR<br>DEPSNEKSDVYSFAVILWELMTLQQPWCNLNPAQVVAAVGFKGRRLEIPKDLNPQVAALIESCWANEPWRRPSFANIMETL
BdMAPKKK18	LAVREKIGAGSFGTVHRADWNGSDVAVKILMDQDLHPERLKEFLREVAIMKSLRHPNIVLLMGAVTQPPNLSIVTEYLSRGNLYRLLH<br>RHGARENLDERRRLSMAFDVAKGMNYLHKRNPPIVHRDLKSPNLLVDKKYTVKVCDFGLSRLKANTFLSSKTAAGTPEWMAPEVLR

	DEPSNEKSDVYSFAVILWELMTLQQPWSNLNPAQVVAAVGFRGRRPEIPSSVDPKVAAIIESCWAKEPWRRPSFTSIMESL
BdMAPKKK19	WQKRKLIGSGTYGCVYEATNRHTGALCAMKEVNIIPDDAKSVESMKQLDQEIKFLSQFKHENIVQYYGSETIDDRFYIYLEYVHPGSI<br>NKYINQHCGAMTESVVRNFTRHILKGLAFLHSQKIMHRDIKGANLLVDVNGVVKLADFGMAKHLSTAAPNLSLKGTPYWMAPEVV<br>QATLVKDVGYDLAVDIWSLGCTIIEMFTGKPPWSGLEGPAAMFKVLNKDPSVPDNLSPEGKDFLRGCFKRNPSERPTASKLLEHPFV
BdMAPKKK20	LELKERVGAGSFGTVHRADWHGSDVAVKVLTDQDVGEAQLKEFLREISIMKRVRHPNVVLFMGAVTKCPHLSIVTEYLPRGSLFRLI<br>NKAAGGEMLDLRRRLRMALDVAKGINYLHCLNPPIVHWDLKTPNMLVDKNWSVKVGDFGLSRFKATTFISSKSVAGTPEWMAPEFL<br>RGEPSNEKCDVYSFGVILWELVTMQQPWSGLGPAQVVGAVAFQNRRLPIPKDTIPELAALVESCWDDDPRQRPSFSSIVDTL
BdMAPKKK21	LNFGNKVASGSYGDLYRGTYCSQDVAIKVLKPERVNADMQREFAQEVYIMRKVRHKNVVQFIGACTKPPRLCIVTEYMSGGSVYDY<br>LHKHKGVFKLPALVGVAIDVSKGMSYLHQNNIIHRDLKTANLLMDENGMVKVADFGVARVKVQSGVMTAETGTYRWMAPEVIEH<br>KPYDHKADVFSFGILMWELLTGKIPYEYLTPLQAAVGVVQKGLRPTVPKNAHAKLGELLQKCWQQDPTQRPDFSEILETL
BdMAPKKK22	LRFYRQSYKAFDQLEGLEVAWNQIKVGDILRNNDDLERLRSEVRLLKTLKHKNVIKFYNSWLDKKNNNINFITEVFTSGTLRQYRIKH<br>KKVDIRALKKWSRQILSGLVYLHSHDPPIIHRDLKCDNIFVNGNQGEVKIGDLGLATILDNARSAHSIIGTPEFMAPELYDEEYNELVDI<br>YAFGMCLLELVTFEYPYCECSNAAQIYKKVSDGEKPGSLAKIEDPEVKFFIEKCITQASQRLSAEELLVDPF
BdMAPKKK23	WRKGEMIGSGAFGQVYLGMNLDTGELLAVKQVLIGSSNATREKAQAHIRELEEEVKLLKNLSHPNIVRYLGTVREEDTLNILLEFVPG<br>GSIQSLLGKLGSFPEAVIRKYTKQILQGLEYLHNNAIIHRDIKGANILVDNKGCIKLADFGASKQVAKLATMTAAKTMKGTPHWMAPE<br>VIVGSGHTFSADIWSVGCTVIEMATGKPPWSQQYQEVALLFHVGTTKSHPPIPEHISPEAKDFLLKCLQKEPELRSTASDLLKHPFV
BdMAPKKK24	LEDLREMGSGAFGTVFHGKWRGTDVAIKRIKNSCFSHPSSQADKLITEFWREAAIISKLHHPNILALYGIVKNGPGGTLGTVTEFMVNG<br>SLKKVLSRKDKYLDWRKRILVAMDAAIGMEYLHSKDIVHFDLKCDNLLVNIKDPSRPICKVADFGLSKMKQATLVSGGMRGTLPWM<br>APELLTMSGTKVSEKVDVYSFGVVMWEILTGEDPYDGMHYGGVIGGILSNTLRPPVPTSCKPEWRKLMEQCWSTEPGRRPSFSEVAT<br>GL
BdMAPKKK25	WRKGDLIGSGAFGQVYLGMDLDSGELLAVKQVLIGSSNSTRDKAQAHIRELEDEVKLLKNLSHLNIVRYIGTVREEDSLNILLEFVPG<br>GSIQSLLGRLGAFPEPVIRKYTKQILHGLEYLHRNGIIHRDIKGANILVDNKGCIKLADFGASKQVEKLATAAKTMKGTPYWMAPEVIV<br>GSGHDFSADIWSVGCTVIEMATGKTPWNQEIQEVSLLYYVGTTKSHPPIPEHLSPEAKDFLLKCLQKEPELRSSASNLLQHPFV
BdMAPKKK26	WQKGKLLGSGTFGCVYEATNRNTGALCAMKEVNIIPDDAKSAESLKQLEQEIKFLSQFKHENIVQYYGSDTIEDRFYIYLEYVHPGSIN<br>KYVKQHYGAITESVVRNFTRHILRGLAFLHGQKIMHRDIKGANLLVDINGVVKLADFGMATHLSTAAPNLSLKGTPYWMAPEMVQA<br>TLSKDVGYDLAVDIWSLGCTIIEMFDGKPPWSDLEGPAAMFKVLHKDPPIPENLSHEGQDFLQCCFKRNAAERPAAIELLDHPFI
BdMAPKKK27	WKKGKLLGSGTFGQVYLGFNSEGGQMCAIKEVKVIADDSNSKECLRQLNQEMLLLNQLSHPNIVQYYGSELSSETLSVYLEFVSGGSI<br>HKLLQEYGPFGETVLRNYTAQILSGLAYLHGRNTVHRDIKGANILVDPNGDIKLADFGMAKHISAYTSIKSFKGSPYWMAPEVIMNTN<br>GYSLSVDIWSLGCTILEMATARPPWSQYEGVAAIFKIGNSKDIPDVPDHLSSEAKSFLKLCLQRDPAARPTAAQLIDHPWV
BdMAPKKK28	MRYNEVLGRGAFKTVYKAFDEVEGIEVAWNQVNIDEVMQCPDNLERLYSEVHLLKSLKHKNVMKFCNYWFDDQKKTINVITELFTS<br>GSLRHYRRKHPRVDLKAIKNWARQILHGLDYLHSHQPPVIHRDLKCDNIFVNGNHGEVKIGDLGLATIMRTPKARSVIGTPEFMAPEL<br>YDENYDELVDIYSFGMCMLEMFTLEYPYSECKNPAQIFKKVSKGVKPAALSKIANPEVKHFIEKCLVPSSERLSAKELLQDSF
BdMAPKKK29	WMRGALLGSGSFGMVYEGISDEGAFFAVKEVSLLDQGSNAQQSILALEQEIALLSQFEHENIVQYYGTDKEESKLYIFIELVTQGSLSS<br>LYQKYKLRDSQVSAYTRQILNGLVYLHERNVVHRDIKCANILVHANGSVKLADFGLAKEMSKINMLRSCKGSVYWMAPEVVNPRKT<br>YGPAADMWSLGCTVLEMLTRQIPYPDVEWTNAFFMIGRGERPPIPSYLSKEAQDFISQCVRVDPEERPSASQLLAHPFV
BdMAPKKK30	LKFGNMVASGSNGDLYRGSYCSQDVAIKVVRPERISADMYRDFAQEVYIMRKVRHKNVVQFIGACTRQPNLYIITDFMSGGSVYDCL<br>HKNSAFKLPEILRVATDISKGMNYLHQNNIIHRDLKTANLLMDENKVVKVADFGVSRVKDQSGVMTAETGTYRWMAPEVIEHRPYD<br>HKADVYSFGIVLWELLTGKIPYGQLTPMQAAVGVVQKGIRPIIPKDTHPKLADLVQKCWHGDSAERPEFSQILEIL
BdMAPKKK31	LLVGHRFASGAYSRLYKGVYDDKPVAIKFIRQPDDDDNGKIAAKLEKQYNTEINALSHLYHKNVIKLVAAYKCEPVFYILTEFLPGGS<br>LRSYLHSTQHHPIPLEKIISIALDIARGLEYIHSQGVVHRDIKPENILFDENFNVKIADFGIACEETLCDLLVQDEGTYRWMAPEMLKRK<br>AYNRKVDVYSFGLILWEMVSGRLPYDNMIPFQVAFAVAHYNMKPILAPDCPKALRPLITQCCAFHPDKRPDFWHIVKIL
BdMAPKKK32	LLIGHRFASGAYSRLFHGIYKEQPVAVKFIRLPDDGEDTELAARLEKQFTTEVTILSRLDHHNVIKLVGACSCPPVYCVITEFLSGGSLR<br>AFLRKLECKSLPLEKIISIALDIAHGMEYIHSQGVIHRDVKPENILFDGEYCAKVVDFGVAFEDVYCNTLEDDPGTYRWMAPEMCKRK<br>PYGRKVDVYSFGLLLWELVSGSIPYEEMTPVQAAFAVVNKNLRPVVPSSCPAPLRQLMEQCWSSQPDKRPEFSEVVPIL
BdMAPKKK33	FRRGEEVTKGYYIAKWYGSKVFVKILDKESFSDCDSIDSFKHELTLLEKARHPNLVQFVGAVTQNVPLMIVSEYHQNGDLASYLETKG<br>RLQSYKAIRFALDIARGLNYLHECKPEPIIHGDLSPKNIVRDDEGTLKVAGFGSFGLIKVSEDKLRMARPVSKFDSVYVAPEIYRNETFD<br>RSVDTFAFGLILYEMIEGTPAFHPKPPEEAAKMICLEGLRPLFKNKPKSYPEDVKELIQECWDTTPSVRPTFSDIIERL
BdMAPKKK34	WNRGVLLGSGSFGTVYEGISDEGVFFAVKEVCVSDQGSNAQQCIFQLEQEIALLSQFEHENIVHYYGTDKEDSKLYIFLELVTQGSLVS<br>LYQKYRLRDTHVSAYTRQILNGLTYLHERNIVHRDIKCANILVHANGSVKLADFGLAKEATKLNMLKSCKGTVYWMAPEVVNPKKT<br>YGPAADIWSLGCTVLEMLTRQLPYPDLEWTQALYRIGKGEPPQIPNVLSRDARDFISQCVKPNPEDRPSASKLLDHPFV
BdMAPKKK35	LLIGHRFASGAHSRLFHGIYQEQPVAVKFIRLPDDEEEAELSAQLEKQFSTEITMLSHLHHRNVIKLVGACSSPPVFCVLTEFLSGGSLR<br>AFLHKQEHKSLPLEKIISVGLDIAHGMAYIHSQGVVHRDVKPENIIFDGECCAKIVDFGIACEEAYCDPLANDPGTFRWMAPEMMKHK<br>PYGRKVDVYSFGLILWEMLTGSVPYDDLTPFQAAFAVFDKNVRPTIPVSCPAALRLLIEQCWALQPDKRPEFWQIVQLL
BdMAPKKK36	FRKGDEVLTGTYQVAKWNGTKVSVKILDRESYCDQEAINSFRHELTVFEKVRHPNVVQFVGAVTQNIPMMIVSEYHANADLASYIQR<br>KGRLHAQKVLRYALDIARGMTYLHQCKPDPIIHCDLKPKNIFLDNGGQMKVGGFGLTRLLKIAPDKVKLANHEALVDTFSYYTAPEL<br>HRNELFDSSVDAYAFGFILFEMVEGLPHANGKASEESSHMQPRYDGMRPSLKNKLKGYPADFKALIEECWDTHTMARPTFSEIIIRL

BdMAPKKK37	LDMGAPFAQGAFGKLYRGTYIGEDVAVKLLEKPENDTERARSLEQQFVQEVMMLSTLRHPNIVRFIGACRKSIVWCIVTEYAKGGSVRQFLARRQNKAVPLRLAVKQALDVARGMAYVHALGFIHRDLKSDNLLIAADRSIKIADFGVARIEVKTEGMTPETGTYRWMAPEMIQHRPYDHKVDVYSFGIVLWELITGMLPFTKMTAVQAAFAVVNKGARPVIPHDCLPSLSHIMTRCWDANPEVRPPFTEIVCML
BdMAPKKK38	LHMGMPFAQGAFGKLYRGTYNGMDVAIKLLERPEAAPVQAQLLEQQFVQEVMMLATLRHPNIVKFIGACRKPMVWCIVTEYAKGGSVRNFLTRRQNRSVPLKLAVKQALDVARGMAYVHGLGFIHRDLKSDNLLISGDKSIKIADFGVARIEVKTEGMTPETGTYRWMAPEMIQHRPYNQKVDVYSFGIVLWELITGTLPFAKMTAVQAAFAVVNKGVRPTIPHDCLPALGEIMTRCWDANPDVRPPFTDVVRML
BdMAPKKK39	LKFEQKLAAGSFGDLYHGTYCSQDVAIKVLKPERVSVDMLREFAQEVYIMKKVRHKNVVQFIGACTRPPILCIVTEFMRGGSIFDYIYNHRGTFQLVDVLRIASDVSKGMSYLHQINIIHRDLKTANLLMDDKVVKVADFGVARVKDQSGVMTAETGTYRWMAPEVIEHSPYDHRADVFSFGVVLWELLAGKLPYEDMTPLQAAVAVVQKDLRPTIPADTHPMLIGLLQKCWQRDPALRPTFAEILDIL
BdMAPKKK40	FTNGKDLSKGTFRKATWRGIPVAVKKLDDDVINDENKVQAFRDELDVLQLIRHPNVVQFLGAVTQSNPMMIVMEFMPKGDLRKHLNKKGALEPSYAVKLALDIARGMSYLHEHKPQSIIHRDLEPSNILRDDTGHLKVADFDLCKMLKWRRKVREEKPVTSVGNACRYVAPEVLRTEEYDNKVDVFSFGLILQEMIEGCLPFYDKKIDEIEKAHSSKERPAFRAPPKHYAHGLKELIEQCWSENPADRPDFRVVIDRL
BdMAPKKK41	LEIRYVIAQGTYGTVYRGTYDGQDVAVKLLDWGEDGFATEAETAALRTSFKQEVAVWHKLSHPNVTKFVGASMGTADLKIPANDSGARANLPARACCVVVEYLAGGTLKQYLIKNRRRKLAYKVVVQLALDLSRGLSYLHSRKIVHRDVKTENMLLDTQRNLKIADFGVARVEAQNPKDMTGATGTLGYMAPEVLDGKPYNRKCDVYSFGICLWEIYCCDMPYPDLSFADVSSAVVHQNLRPDVPRCCPSAFANIMRKCWDANPDKRPDMDEVVQLM
BdMAPKKK42	LFIGNKFAAGANSRIYRGIYKQRAVAVKMVRIPERDEARRALLEDQFNSEVAFLSRLYHPNIVQFIAACKKPPVYCIITEYMSQGTLRMYLNKKDPYSLSPETILKLALDISRGMEYLHAQGVIHRDLKSQNLLLNDEMRVKVADFGTSCLETRCQATKGNKGTYRWMAPEMIKEKPYTRKVDVYSFGIVLWELTTCLLPFQGMTPVQAAYAASEKNLRPPLSSSCPPVLNNLIKRCWSANPARRPEFSYIVSVL
BdMAPKKK43	MFVGSKIGEGAHGKVYKGKYGDKIVAIKVLNSGSTPEERATLEARFIREVNMMCRVKHDNLVKFIGACKEPLMVIVSELLPGMSLKNYLNSIRPSQLDIHTAIGYALNIARALECLHANGIIHRDLKPDNLLLTANRKKVKLTDFGLAREETVTEMMTAETGTYRWMAPELYSTVTLQRGEKKHYTNKVDVYSFGIVLWELLTNKMPFEGMSNLQAAYAAAFKQVRPPFPEETPQELVFIVQSCWVEDPTLRPSFSQIIRMLDAFL
BdMAPKKK44	WKRGKLLGSGTFGQVYLGFNSENGQFCAIKEVQVISDDPHSKERLKQLNQEIDMLKQPSHPNIVQYYGSEMTEDTLSIYLEYVSGGSIHKLLREYGPFKEPVIRNYTGQILSGLAYLHGKNTVHRDIKGANILVGPNGEVKLADFGMAKHISSFAEIRSFKGSPYWMAPEVIMNSKGYSLAVDIWSLGCTIIEMATARPPWHQYEGVAAIFKIANSKDIPEIPDIFSEEGKSFLQMCLKRDPAARASASQLMDHPFV
BdMAPKKK45	LFVGPRIGEGGHAKVYEGKYKNQNVAIKIVHKGDTPEEVVKRQGRFLREVTMLSRVQHKNLVKFIGACLEPVMVVVTELLVGGSLRKYLVSLRPRNLEPRTAVGFALDIARAMECLHAHGIIHRDLKPENLLLTADQRTVKLVDLGLAREETLTEMMTAETGTYRWMAPELYSTVTLRHGEKKHYNHKVDVYSFAIVLWELLHNRLPFEGMSNLQAAYAAAFKNIRPSADNLPEELSEILTSCWKEDPNERPNFTQIVQML
BdMAPKKK46	LVIKGVIARGTFGTVHRGVYDGQDVAVKLLDWGEDGHRSEQEITALRSAFAQEVAVWHKLDHPNVTKFIGAIMGARDLNVQTEHGHLGMPSNICCVVVEYLAGGALKNFLIKNRRRKLAFKVVVQLALDLARGLSYLHSEKIVHRDVKTENMLLDKTRTVKIADFGVARVEASNPSDMTGETGTLGYMAPEVLNGHPYNRKCDVYSFGICLWEIYCCDMPYPDLSFSEVTSAVVRQNLRPEIPRCCPSALANVMKRCWDANPDKRPEMAEVVSLI
BdMAPKKK47	LVVRGVIARGTFGTVHRGVYDGHDVAVKLLDWGEDGHRSEQEVTAVRAAFSQEVTVWHKLDHPNVTKFIGAIMGARDLNIQTENGNIGMPTNVCCVVVEYLPGGALKTFLIKNRRRKLAFKVVVQIALDLARGLSYLHSKKIVHRDVKTENMLLDKTRTVKIADFGVARHEAANPSDMTGETGTLGYMAPEVLNGNPYNRKCDVYSYGICLWEVYCCDMPYADLSFSEVTSAVVRQNLRPEIPRCCPSSFANVMKRCWDANPDKRPEMAEVVSML
BdMAPKKK48	LEIRAKFASGRHSRVYSGRYAGREVAIKMVSQPEEDAALAAELERQFASEVALLLRLRHQNIISFVAACKKPPVFCIITEYMAGGSLRKYLHQQEPYSVPIELVLKLALDIARGMSYLHSQGILHRDLKSENILLGEDMSVKVADFGISCLESQCGSGKGFTGTYRWMAPEMIKEKNHTRKVDVYSFGIVLWEILTSLVPFSEMTPEQAAIAVALKNARPPLPASCPLAMSHLISQCWATNPERRPQFDDIVAIL
BdMAPKKK49	FTKAIIIGKGSFGEILKANWRGTPIAVKRILPSLSDDRLVIQDFKHEVNLLIKLRHPNIVQFLGAVTETKPLMLITEFLRGGDLHQYLKEKGSLSPVTAVNFALDIARGMAYLHNEPNVIIHRDLKPRNILLVNTAANHLKVGDFGLSKIIKSQHANDVYKMTGETGSYRYMAPEVFKHRKYDRKVDIFSFAMILYEMLEGDPPFSNYEPYEAAKYVADGHRPAFRSKGHISELKDLTELCWDADINLRPSFLEILKRL
BdMAPKKK50	TKLEELLGKGAMKTVYKGFDEVRGVEVAWNQANLADVLRTPDALHRIYSEVHLLSTLRHDSIIAFHASWLSTTTSSSSSSPRAGAGGGRTFNFITELFSSGTLRSYRLRYPRVSLRAVRGWARQILRGLAYLHGHDPPVIHRDLKCDNLFVNGHQGTVKIGDLGLAAVLRGARASAHSVIGTPEFMAPEMYDEDYGVLVDVYSFGMCVLEMLTAEYPYSECCNPAQIYKKVTSGKLPDAFYRVEDDEARRFIGRCLVAASARPSAQELLLDPF
BdMAPKKK51	LRRLRTLGRGASGAVVWLASDDASGELLAVKSGSAARLQREGRVLAGLCSPHIVPCLGSRAAPGGEYQLFLEFAPRGSLADEAARSTGGRLAERAIQGYAADVARGLAYLHGNSLVHGDVKARNVMVGADGRAKLADFGCARSTTATDSGRPIGGTPAFMAPEVARGEEQGPAADVWALGCTVVEMATGRAPWSDMDDVFAAVHRIGYTDAVPELPGWLSPDAKDFLGKCLARNPRHRPTAAQLLEHPFL
BdMAPKKK52	WTRLRTLGCGASGAVVSLATDAASGELFAVKSVRGAADADLLSREQGILSGLSSPHIVRCIGGLGEDRDGSYHLFLEFAPGGSLADEVARNGGFLEEHAIRAYAADVLRGLAYIHGESLVHGDVKARNVVIGVDGRAKIADFGCARALDSTRPIGGTPAFMAPEVARGEEQGPAADVWALGCTIIEMATGRAPWSDMDDVLAAVHRIGYTDAVPEVPMWMSAEAKNFLAMCFARNARDRCTAEQLLEHPFV
BdMAPKKK53	WTRVRTLGRGASGAVVSLAAADLSGALFAVKSARAAGAEQLRREGDILSGLSSPHVLPCLGFRADSGECQLFLEFAPGGSVADVAERSGGRLEECAIRAYAADVARGLAYLHGMSLVHGDLKGRNVVVGADGRAKLADFGCARTVDSDRPIGGTPAFMAPEVARGEEQGPAADVWALGCTVVEMATGRAPWSDMDDVLAAMHRIGYTDAVPEVPGWLSAEAKHFLAMCFARDARNRCTAAQLLEHPFL
BdMAPKKK54	GRYADVLGLGSVKKVYRAFDQEEGIEVAWNRVRLRALADRDPGMVDRLHAEVRLLRSLSHDHIIGFHKVWLDRDAGVLSFITEVCNSGSLREYRARHRHVSVKALKKWARQILLGLHHLHTHDPCIIHRDLNCSNVFINGNTGQVKIGDLGLAAIVDKTHVAHTILGTPEFMAP

	ELYTETYTESVDIYSYGMCVLEMVTREMPYAECESVVQIYHSVTRGVPPAALRRLKDPELRGFIDRCIGQPRNRPTAAELLLDPF
BdMAPKKK55	LRRVRTLGRGASGAVVWLASDEASGELLAVKSAAAAGGAARLLEREGCVLTGLCSPHIVPCLGSRAAEECGEYQLFLEFAPGGSLADEAAKSAGGRLPEPAIRAYAGDVARGLEYLHARSLVHGDVKARNVVIGGDGRARLTDFGCARPVDSLLPMGGTPAFMAPEVARGEEQGTASDVWALGCTVVEMATGRAPWSDMSDLFAAVHRIGYTADVPEVPGWLSAEAKDFLDGCFRRTPGDRSTAAQLLDHPFI
BdMAPKKK56	MGSSKKMAQLNTEIQLLKTLKHQNIEKSFASWIDEDNKTVNIITELFTSGSLRQYRKKHKKVSIKAMRRWAVQILTGLEYLHSQEPAIIHRDLKCDNIFINGNGGTVKIGDFGLATFLQQQKTKSIKGTLEFMAPELFTGVYNELVDIYSFGMCMLEMVTCEYPYSECQGMGHIYKKISEGKKPAALSKVEDAELRSFIEICLAPVAERLPASELLRSSF
BdMAPKKK57	WRRGPVIGRGASATVSIATDALTGEAFAVKSVGIALAGALRREQSVLSALSSPYVVSCVGSSGASDDGRSYELFLELAPGGSLADEMKRCGGRCEEALVASRAGDVLRGLAHVHGAGIAHCDVKARNVLLGADGRAKLADFGCARWTTKAKEDGIGNAIMGTPMFMSPEAARGEAQGAAADIWALGCTVIEMATGGAPWRFSSPVAALHHVAHSGDVPEAPSWFSEQGKDFLARCLVRDPAQRWTAEQLLEHPFV
BdMAPKKK58	WKRVRTLGRGASGAEVFLAADDVSGELFAVKSAGAGATLRREQEIMAGLRSPNVLSCIGGRVGHDGSYQLFLEFAPGGSLADAVRSGAGGRLEERVVRAYAEDMAAGLAYLHGAGLVHGDVKPRNVVIGGDGRAKLADFGCSRKTDSRGPILGGTPAFMAPEVARGEEQGPAADIWALGCTVVEMATGRAPWNGTGMEDDGVLAALHRIGYTDDAVPEVPKWLSADAKDFLARCLTRRPSDRCTAAQLLEHPFL
BdMAPKKK59	LTRLRTLGRGSSGAVVSLFAADGELLAVKSAATGGAAQLRREGGILASLCSPYVLPCLGSRAAAGGEYQLLLEFAPGGSLADEVARNGGRLEEPAVRAYAGHVARGLAYLHGESMVHGDVKARNVVIGADGWAKLADFGCARRCPGPGPGPILGGTPAFMAPEVARGEEQGPAADVWALGCIIIEMATGRAPWADMDAGCGAADVISAVRRIGYTDAVPEAPERMSPDAKDFLDKCLRRSAGERWTAAQLLEHPFL
BdMAPKKK60	WTRGKCIGKGAFGTVHSAVDRATGRAFAVKSVDAKQGGAMAMACLESEIRILRRLRSPYVVAYLGDDATAASRNLHMELVPGGTAADAAKGGLGERAARRVLRRVASALQYLHDVAGVVHGDVKGRNVLLGSGIDSTKLADFGAARLVSEPAPRGPRGTPAWMAPEVARGGAPTPASDVWSLGCTAVELLTGKRPWSETGGWLEVGAMLLLIGFGGKRPEFPAAACVSDSCRDFLDKCLRRDAGERWSCEQLL
BdMAPKKK61	WTRGPAIGRGSSAVVSLAVDRVTGVVFAVKSVGAGRAAELRREQSILRGLSSPYVVRCLGYVADGSGEMLMEYAAGGSLADEIRRCGGRCPEALIRCRARDVLRGLAHAHGAGVAHCDVKARNVLVGSHGRAMLADFGCARRIVASSREPQLMGMGGTPMFMAPEAARGEERGAAADVWAVGCTVIEMATGGAPWRSRFADPVAALHHVAFSGEAPELPPWLSEAGKEFLGRCLRQDPRERWTAEQLLEHPWF
BdMAPKKK62	LALGEQVGHGSCGTVYHALWYGSDVAVKVFSKQDYSEEMIQTFRQEVSLMKKLRHPNIILFMGAVASQQRLCIVTEYLPRGSLFSLLRRTTGKLDPRRRIHMAIDIARGMNYLHNCSPTIVHRDLKSSNLLVDKNWNVKVADFGLSRLKVETFLSTKTGKGTPQWMAPEVLRNEPSNEKSDVYSFGVVLWELVTEKIPWDNLNIMQVIGAVGFMDQRLEIPSGMDPQWASMIESCWDSDPQRRPSFQELLERL
BdMAPKKK63	LVIGEQVGQGSCGTVYHALWYGSDVAVKVFSKQEYSEEMINTFRQEVSLMKKLRHPNIILFMGAAASQQQLCIVTEFLPRGSLFRLLQKNTGKLDPRRRVNMAIDIARGMNYLHNSIPTVVHRDLKSSNLLVDKNWTVKVADFGLSRLKLETFLTTKTGKGTPQWMAPEVLRSEPSNEKSDVYSYGVVLWELITQKVPWDTLNTMQVIGAVGFMDHRLEIPSDADPQWSSMIESCWVSDPQRRPSFRELLERL
BdMAPKKK64	LEELRELGSGTFGTVYHGKWRGTDVAIKRIKKSCFAGRSSEQEKLTKDFWREAQILSKLHHPNVVAFYGVVPDGTGGTLATVAEFMVNGSLRNVLLRKDRTLDRRRKLIIAMDAAFGMEYLHSKSIVHFDLKCDNLLVNLRDPQRPICKVGDFGLSRIKRNTLVSGGVRGTLPWMAPELLNGSSSRVSEKVDVFSFGIVLWEILTGEEPYANMHCGAIIGGIVNNSLRPPIPETCEPEWRSLMEQCWSANPDVRPSFTKVTDRL
BdMAPKKK65	LEELRELGSGTFGTVYHGKWRGTDVAIKRIKKSCFTGRSSEQERLAHEFWREAEILSKLHHPNVVAFYGVVKDGPGGTLATLTEFMVNGSLRHVLQRKDKSPDLRKRLIIAMDAAFGMEYLHSKNIVHFDLKCDNLLVNLRDPSRPICKVS
BdMAPKKK66	LEIRYVIAQGTYGTVYRGTYDGQDVAVKLLDWGEDGFATEAETAALRSSFKQEVAVWHKLSHPNVTKFVGASMGTTDLKIPANDTGARANLPVRACCVVVEYLAGGTLKQYLIKNRRRKLAYKVVVQLALDLSRGLSYLHSQKIVHRDVKTENMLLDTQRNLKIADFGVARVEAQNPKDMTGATGTLGYMAPEVLDGKPYNRKCDVYSFGICLWEIYCCDMPYPDLSFADVSSAVVHQNLRPDVPRCCPSAFANIMRKCWDANPDKRPDMDEVVQLM
BdMAPKKK67	WKKGKLLGSGTFGQVYLGFNSESGHFCAIKEVQVILDDPHSKERLRQLNQEVDLLRQLSDRNIVQYYGSQLTDEALSIYLEYVSGGSIHKLLRDYGPFKEPVIRNYTRQILSGLAYLHGRNTMHRDIKGANILVGPTGDVKLADFGLAKDITSFAEISSFRGSPYWMAPEAVMHSKGYSLAVDIWSLGCTVIEMATARHPWHPLEDVPALFKIANSKDIPEIPESISKEGKDFLSLCLKRDPLERPSATQLLDHPFV
BdMAPKKK68	WTRVRTLGRGASGAEVFLAADDASGELFAVKSASAAAMGAAAALRREQGIMAGLRSPNVLSCIGGRGGRDGSYQLFLEFAPGGSLADGGRLEESAVRAYAADLATGLAYLHGAGLVHGDVKPRNVVIGGDGCAKLADFGCSRKADSCAPILGGTPAFMAPEVARGEEQGPAADIWALGCTVVEMATGRAPWNGTGMDDDGVLAALHRIGYTYAVPQVPQWLTAEAKDFLSRCLVRRPGDRCTAAQLLEHPFL
BdMAPKKK69	WTRVRTLGRGASGAEVFLAADDASGELFAVKSASAAARGAAATLRREQGIMAGLRSPNVLSCIGGRGGHDGSYQLFLEFAPGGSLADGGRLEERAVRAYAADVATGLAYLHGVGLVHGDVKPRNVVIGSDGRAKLADFGCSRKAGSCMPILGGTPAFMAPEVARGEEQGPAADIWALGCTVVEMATGRAPWNGTGMDDDGVLAALHRIGYTDAVPQVPQWLSADAKDFLARCLIRRPSDRCTAAQLLEHPFL
BdMAPKKK70	WVKGGHIGSGSFGSVYEAMSDDGFFFAVKEVSLIDQGINAKQRIIQLEHEVSLLSRLEHDNIVQYYGTDKEDGKLYIFLELVSQGSLAALYQRYCLQDSQVSAYTRQILNGLNYLHQRNVLHRDIKCANILVDANGSVKLADFGLAKEMSILSQARSSKGTVFWMAPEVAKAKPHGPPADIWSLGCTVLEMLTCKVPYPDMEWTHALLKIGRGIPPKIPDKLSEDARDFIAKCVQANPKDRPSAAQLFDHPFV
BdMAPKKK71	LDIAENLASGSRGDTLRGTYGGEEVFVKFVSSEDPSQIVSKEFKQEILMLREVDHANIIRLIGSCTKEPQFCMMTEYMSGGSLFDFLKNEHNVLDLPMILKFALDICRGMAYLHQKGIIHRDLKSANLLIDKYQVVKVAHFGLSRYQDQEGVMTAETGTYRWMAPEVMNHQHYGHAADVYSFAIVLWELMTRKIPYDTLTTLQAAVEVLKGMRPPLPENAHPRLLTLMQRCWDASPSKRPSFSDAITEL
BdMAPKKK72	LSIIKKLASGSCGHTFLGTYGGEEVSVKVLRSADATQILWKEFKQEILMLREVYHANIIRSIGSCIKPPHFYIITEYMSGGSLFDFLHNKHNVLDLPMILKFALDICRGMAYLHQKGIIHRDLKSANLLMDKDHVVKVADFGLSRYQDREGVMTAETGTYRWMAPEVMKHQQYGPAADVYSFAIVLWELMTSKMPYDTINPIQAAFNVWQGMRPQIPKNAHPRLLTLMQRCWDASPSKCPPFSDAIAEL

BdMAPKKK73	LTKGERIASGTSADLIVNHENIIKYYGACTNHPNYCIVTEYMPEGNLYEFLHKQKHLLDLREILRIAISISKGMEYLHRNNIIHRDLKTANVLKGYGQALKIEGFNVAILGSQEDQMTAETGTYRWMAPEVINHKPYDHKADVFSFAIVLWELVTLKVPYDKMTPLQAALGVRQGFRLEIPPRVHPGLSKLIQQCWDEDPNARPVFGEIIIQL
BdMAPKKK74	FLFHRYRGTYKGSDVAIKMLRVAHLNNASEVEFLQEVLILRSVNHENILQFYGASTRHPNCCIVTEYMPEGNLYEFLHKQNDLLEINEILRIAISISKGMEYLHRNNIIHRDLKTANVLKGYGQVLKIADFGVSRIGSQEGQMTAETGTYRWMAPEIIDHKPYDHKADVFSFAIVLWELITLKVPYDDMTPLQAALGVRQGFRLQIPSGTHPGLSKLIRQCWDEDPEIRPAFGEIITQL
BdMAPKKK75	LKMGDMIASGSCGDLFHGTYFGEDVAVKVLKAEHLNKNVWNEFTQEVYILREVCHTNVVRFIGACTKPPKFCIITEYMSGGSLYDFVHKQRNVLDLPTLLKFACDVCRGMCYLHQRGIIHRDLKTANLLMDKDHVVKVADFGVARFQDQGGIMTAETGTYRWMAPEVINHQPYDNKADVFSFAIVLWELIASKIPYDTMTPLQAAVGVRQGLRPGLPENTHPKLLDLLQRCWETIPSNRPSFPDILTEL
OsMAPKKK1	LVIGERIGLGSYGEVYRADWNGTEVAVKKFLDQDFYGDALDEFRSEVRIMRRLRHPNIVLFMGAVTRPPNLSIVSEYLPRGSLYKILHRPNCQIDEKRRIKMALDVAKGMNCLHISVPTIVHRDLKSPNLLVDNNWNVKVCDFGLSRLKHSTFLSSKSTAGTPEWMAPEVLRNEQSNEKCDVYSFGVILWELATLRMPWSGMNPMQVVGAVGFQDKRLDIPKEIDPLVARIIWECWQKDPNLRPSFAQLTSAL
OsMAPKKK2	LLIGERIGLGSYGEVYHADWNGTEVAVKKFLDQEFYGDALAEFRCEVRIMRRLRHPNIVLFMGAVTRPPHLSIVSEYLPRGSLYTIIHRPDCQIDEKCRIKMALDVARGMNCLHTSVPTIVHRDLKSPNLLVDNNYIYRSVILDFHV
OsMAPKKK3	LVLKEKIGAGSFGTVHRADWNGSDVAVKILMEQDFHPERLKEFLREVAIMKSLRHPNIVLFMGAVTQPPKLSIVTEYLSRGSLYRILHKHGARENLDEKRRLSMAFDVAKGMNYLHKRNPPIVHRDLKSPNLLVDKKYTVKVCDFGLSRLKANTFLSSKTAAGTPEWMAPEVIRDEPSNEKSDVYSFGVILWELMTLQQPWSTLNPAQVVAAVGFNGRRLEIPSSVDPKVAAIMESCWTKEPWRRPSFASIMESL
OsMAPKKK4	LHIGERIGLGSYGEVYHADWNGTEVAVKKFLDQDLSGVALDQFKCEVGIMSRLRHPNVVLFLGYVTQPPNLSILTEYLPRGSLYRLLHRPNSQIDETRRLKMALDVAKGMNYLHASHPTIVHRDLKSPNLLVDKNWVVKVSDFGMSRLKHHTFLSSKSTAGTPEWMAPEVLRNEPSNEKCDVYSFGVILWELATMRVPWSGLNPMQVVGAVGFQNRRLEIPKEIDPLVATIISSCWENDPSKRPSFSQLLSPL
OsMAPKKK5	LVIGEQIGQGSCGTVYHALWYGSDVAVKVFSKQEYSEEVIQTFRQEVSLMKKLRHPNILLFMGAVTSPQRLCIVTEFLPRGSLFRLLQRNNTKLDWRRRVHMALDIARGMNYLHHFSPLIIHRDLKSSNLLVDKNWTVKVADFGLSRLKRETFLTTKTGKGTPQWMAPEVLRNEPSDEKSDVYSYGVILWELVTQKIPWENLNSMQVIGAVGFMNHRLEIPSETDPQWTSLILSCWETDSQLRPSFQQLLERL
OsMAPKKK6	ITIGERIGLGSFGEVYRGEWHGTEVAVKKFLQQDISSDALEEFRTEVRIIKRLRHPNVVLFMGAITRVPNLSIVTEFLPRGSLFRLIHRPNNQLDERKRLRMALDVARGMNYLHNCTPVIVHRDLKSPNLLVDKNWVVKVCDFGLSKMKNKTFLSSRSTAGTAEWMAPEVLRNEPSDEKCDVFSYGVILWELCTLLQPWEGMNAMQVVGAVGFQNRRLDIPDNTDPAIAEIIAKCWQTDPKLRPSFADIMASL
OsMAPKKK7	ITLGERVGLGSFGEVYKGEWHGTEVAVKKFLQQDISSDALDEFRTEFQIMKRLRHPNVVLFMGAVTRVPNLSIVTEFLPRGSLFRLIHRPNNQLDERRRLRMALDVARGMNYLHNCSPVVVHRDLKSPNLLVDKNWVVKVCDFGLSRMKNSTFLSSRSTAGTAEWMAPEVLRNEPSDEKCDVFSYGVILWELFTLLQPWEGMNPMQVVGAVGFQQRRLDIPAHVDPTIAEIIRRCWQTDPKMRPSFSEIMSSL
OsMAPKKK8	WKKGKLLGSGTFGQVYQGFNSEGGQMCAIKEVKVISDDSNSKECLRQLHQEIVLLSQLSHPNIVQYYGSDLSSETLSVYLEYVSGGSIHKLLQEYGAFGEAVLRNYTAQILSGLAYLHGRNTVHRDIKGANILVDPNGDIKLADFGMAKHISAHTSIKSFKGSPYWMAPEVIMNTNGYSLSVDIWSLGCTIIEMATARPPWIQYEGVAAIFKIGNSKDIPDIPDHLSFEAKNFLKLCLQRDPAARPTAAQLMEHPFV
OsMAPKKK9	WKKGKLVGRGTFGHVYIGFNSDKGEMCAMKEVTLFSDDPKSKESAKQLCQEILLLNRLQHPNIVRYYGSEMVDDKLYIYLEYVSGGSIHKLLQEYGQFGEPAIRSYTKQILLGLAYLHAKNTVHRDIKGANILVDPNGRVKLADFGMAKHINGQQCAFSFKGSPYWMAPEVIKNSNGCNLAVDIWSLGCTVLEMATSKPPWSQYEGIAAVFKIGNSKELPPIPDHLSEEGRDFIRQCLQRNPSSRPTAVDLLQHSFI
OsMAPKKK10	WKKGKLIGRGTFGHVYVGFNSDSGEMCAMKEVTLFLDDPKSKESAKQLGQEISLLSRLQHPNIVQYYGSETVDDKLYIYLEYVSGGSIHKLLQEYGQLGEQAIRSYTQQILSGLAYLHAKNTVHRDIKGANILVDPSGRVKLADFGMAKHINGQQCPFSFKGSPYWMAPEVIKNSNGCNLAVDIWSLGCTVLEMATSKPPWSQYEGIAAMFKIGNSKELPPIPDHLSEPGKDFIRKCLQRDPSQRPTAMELLQHPFV
OsMAPKKK11	WQKGRLLGSGTFGCVYEATNRQTGALCAMKEVNIIPDDAKSAESLKQLEQEIKFLSQFKHENIVQYYGSDTFEDRFYIYLEYVHPGSINKYVKQHYGAMTESVVRNFTRHILRGLAFLHGQKIMHRDIKGANLLVDVSGVVKLADFGMAKHLSTAAPNLSLKGTPYWMAPEMVQATLNKDVGYDLAVDIWSLGCTIIEMFNGKPPWSDLEGPAAMFRVLHKDPPIPDNLSHEGKDFLQFCFKRNPAERPTASELLEHPFI
OsMAPKKK12	LILKEKIGAGSFGTVHRADWNGSDVAVKILMEQDFHPDRFREFMREVAIMKSLRHPNIVLFMGAVTEPPNLSIVTEYLSRGSLYKLLHRSGAKEVLDERRRLNMAFDVAKGMNYLHKRSPPIVHRDLKSPNLLVDKKYTVKVCDFGLSRLKANTFLSSKSLAGTPEWMAPEVLRDEPSNEKSDVYSFGVILWELMTMQQPWCNLNPAQVVAAVGFKGRRLDIPKDLNPQVAALIESCWAKIILSGYRLL
OsMAPKKK13	WRKGELIGSGAFGQVYLGMNLDTGELLAVKQVLIGSNNATREKAQAHIRELEEEVKLLKNLSHPNIVKRYLGTVREEDTLNILLEFVPGGSIQSLLGKLGSFPEAVIRKYTKQILQGLEYLHNNAIIHRDIKGANILVDNKGCIKLADFGASKQVAKLATITAAKTMKGTPHWMAPEVIVGSGHNFSADIWSVGCTVIEMATGKPPWSQQYQEVALLFHVGTTKSHPPIPEHLSPEAKDFLLKCLQKEPELRSTASDLLKHPFV
OsMAPKKK14	IELKERVGAGSFGTVYRADWHGSDVAVKVLTDQDVGEAQLKEFLREIAIMKRVRHPNVVLFMGAVTKCPHLSIVTEYLPRGSLFRLINKASAGEMLDLRRRLRMALDVAKGINYLHCLNPPIVHWDLKTPNMLVDKNWSVKVGDFGLSRFKANTFISSKSVAGTPEWMAPEFLRGEPSNEKCDVYSFGVILWELMTMQQPWNGLSPAQVVGAVAFQNRRLPIPQETVPELAALVESCWDDDPRQRPSFSSIVDTL
OsMAPKKK15	WRKGDLLGSGAFGSVFLGMDLDSGELLAVKQVLIGSSNATREKAQGHIRELEDEVKLLKNLSHPNIVRYIGTVREENSLNILLEFVPGGSIQSLLGRLGSFPEAVIRKYTKQILHGLEYLHRNGIIHRDIKGANILVDNKGCIKLADFGASKQVEKLATTAKTMKGTPYWMAPEVIVGSGHDFSADIWSVGCTVIEMATGKTPWNQEIQEVSLLYYVGTTKSHPPIPEHLSPEAKDFLLKCLQKEPELRSTASDLLLHPFV
OsMAPKKK16	WKKGKLLGSGTFGQVYLGFNSENGQFCAIKEVQVISDDPHSKERLKQLNQEIDMLRQLSHPNIVQYYGSEMTDDALSIYLEFVSGGSIHKLLREYGPFKEPVIRNYTGQILSGLAYLHGRNTVHRDIKGANILVGPNGEVKLADFGMAKHISSFAEIRSFKGSPYWMAPEVIMNGRGYHLPVDIWSLGCTIIEMATAKPPWHKYEGVAAIFKIANSKEIPEIPDSFSEEGKSFLQMCLKRDPASRFTATQLMDHPFV
OsMAPKKK17	LKFGNKVASGSYGDLYRGTYCSQDVAIKVLKPERINADMQREFAQEVYIMRKVRHKNVVQFIGACTKPPNLCIVTEYMSGGSVYDYLHKHKGVFKLPALLGVVMDVSKGMSYLHQNNIIHRDLKTANLLMDENGTVKVADFGVARVKAQSGVMTAETGTYRWMAPEVIEHK

	PYDHKADVFSFGILMWELLTGKIPYEYLTPLQAAVGVVQKGLRPTIPKNAHAKLSELLQKCWQQEPAERPDFSEILETL
OsMAPKKK18	WQKGKLIGSGTFGCVYEAANRHTGALCAMKEVNIIPDDAKSAESLKQLEQEIKFLSQFKHENIVQYYGSEYIEDRFYIYLEYVHPGSIN<br>KYVNQHCGAMTESVIRSFTRHILKGLAFLHSQKIMHRDIKGANLLVDVNGVVKLADFGMAKHLSTAAPNLSLKGTPYWMAPEVVQA<br>TLVKDVGYDLAVDIWSLGCTIIEMFTGKPPWSGLEGPAAMFKVLHKDPSIPDSLSPEGKEFLRCCFRRNPAERPTASKLLEHPFV
OsMAPKKK19	WKRGKLLGSGTFGQVYLGFNSENGQFCAIKEVQVFLDDSHSKERLRQLNQEIDMLKQLSHQNIVQYYGSELADEALSIYLEYVSGGSI<br>HKLLREYGPFKEPVIRNYTRQILSGLAYLHGRNTVHRDIKGANILVGPNGEVKLADFGMAKHVTSFAEIRSFRGSPYWMAPEVVMNN<br>KGYNLAVDIWSLGCTIIEMATAKHPWYPYEDVAAIFKIANSKDIPEIPDCFSKEGKDFLSLCLKRDPVQRPSAASLLGHPFV
OsMAPKKK20	GRYNDVLGKGASKTVYRAFDEYQGMEVAWNQVKLHDFLQSPEDLERLYCEIHLLKTLKHRNIMKFYTSWVDVSRRNINFITEMFTS<br>GTLRQYRQKHMRVNIWAVKHWCRQILSGLLYLHSHDPPIIHRDLKCDNIFVNGNQGEVKIGDLGLAAILRKSHAVHCVGTPEFMAPE<br>VYEEEYNELVDIYSFGMCVLEMVTFEYPYSECTHPVQIYKKVISGTKPEALYKVKDPMVRQFVEKCLATASRRLSARELLKDPFL
OsMAPKKK21	LEELRELGSGTFGTVYHGKWRGSDVAIKRIKKSCFTGRSSELERLANEFWREAEILSKLHHPNVVAFYGVVKDGPGGTLATVTEFMV<br>NGSLRHVLQRKDKYLDRRKRLIIAMDAAFGLEYLHSKNIVHFDLKCDNLLVNLKDQSRPICKVGDFGLSKIKRNTLVSGGVRGTLPW<br>MAPELLNGSSNKVSEKVDVFSFGIVMWEILTGEEPYANMHYGAIIGGIVNNTLRPPVPASCDPEWRRLMEQCWAPDPSQRPAFTEIAG<br>RL
OsMAPKKK22	WMRGALLGSGSFGMVYEGISDEGAFFAVKEVSLLDQGSNAQQSILALEQEIALLSQFEHENIVQYYGTDKEESKLYIFIELVTQGSLSS<br>LYQKYKLRDSQVSAYTRQILNGLVYLHERNVVHRDIKCANILVHANGSVKLADFGLAKEMSKINMLRSCKGSVYWMAPEVVNPKK<br>TYGPQADIWSLGCTVLEMLTRNIPYPNVEWTNAFFMIGKGERPQIPSYLSKDAQDFISQCVQVDPEQRPSASQLMSHPFV
OsMAPKKK23	LEELRELGSGTFGTVYHGKWRGTDVAIKRIKKSCFAGRSSEQEKLTKDFWREAQILSKLHHPNVVAFYGVVPDGTGGTLATVTEFMV<br>NGSLRNVLLRKDRMLDRRKRLIIAMDAAFGMEYLHSKSIVHFDLKCDNLLVNLRDPQRPICKVGDFGLSRIKRNTLVSGGVRGTLPW<br>MAPELLNGSSSRVSEKVDVFSFGIALWEILTGEEPYANMHCGAIIGGIVNNTLRPPIPKNCEPEWRQLMEQCWSADPDIRPSFTEVTDRL
OsMAPKKK24	YMLGDEIGKGAYGRVYKGLDLENGDFVAIKQVSLENIPQEDLNIIMQEIDLLKNLNHKNIVKYLGSLKTRSHLHIILEYVENGSLANIIK<br>PNKFGPFPESLVAVYIAQVLEGLVYLHEQGVIHRDIKGANILTTKEGLVKLADFGVATKLTEADINTHSVVGTPYWMAPEVIEMSGVC<br>AASDIWSVGCTVIELLTCAPPYYDLQPMPALFRIVQDVHPPIPEGLSPEITDFLRQCFQKDSIQRPDAKTLLMHPWL
OsMAPKKK25	LFVGPRIGEGAHAKVYEGKYKNQNVAIKIVHKGDTPEEMVKREGRFLREVTMLSRVQHKNLVKFIGACLEPVMVVVTELLVGGSLR<br>KYLVGLRPRSLEPRVAVGFALDIARAMECLHAHAIIHRDLKPENLLLTADQRTVKLVDLGLAREETLTEMMTAETGTYRWMAPELYS<br>TVTLRHGEKKHYNHKVDVYSFAIVLWELLHNRLPFEGMSNLQAAYAAAFKNIRPSADNLPEELSEILTTCWKEEPNERPNFTQIVQML
OsMAPKKK26	LKFERKLASGSFGDLYHGTYCSQDVAIKVLKPERVSVDMLREFAQEVYIMKKVRHKNVVQFIGACTRPPILCIVTEFMRGGSIFDFLYN<br>FRGTFQLPDVLRIASDVSKGMNYLHQINIVHRDLKTANLLMDDQVVKVADFGVARVKDQSGVMTAETGTYRWMAPEVIEHLPYDQ<br>RADVFSFGIVIWELLTGKLPYEDMTPLQAAVAVVQKDLRPIIPADTHPMLAGLLQKCWQKDPALRPTFSEILDIL
OsMAPKKK27	LEIRYVIAQGTYGTVYRGTYDGQDVAVKLLDWGEDGFATEAETAALRTSFKQEVAVWHKLSHPNVTKFVGASMGTTDLKIPTNNSN<br>AGARTNLPARACCVVVEYLAGGTLKQYLIKNSRRKLAYKVVVQLALDLARGLSYLHSRKIVHRDVKTENMLLDTQRNLKIADFGVA<br>RVEAQNPKDMTGATGTLGYMAPEVLDGKPYNRKCDVYSFGICLWEIYCCDMPYPDLSFADVSSAVVHQNLRPDVPRCCPSAFANIM<br>RKCWDANPDKRPDMDEVVQLLEAL
OsMAPKKK28	WNRGMLLGSGSFGTVFEGISDEGVFFAVKEVCLCDQGSNAQQCIFQLEQEIALLSQFEHENIVQYYGTDKEDSKLYIFLELVTQGSLAS<br>LYQKYRLRDTHVSAYTRQILNGLTYLHERNIVHRDIKCANILVHANGSVKLADFGLAKEITKFNVLKSCKGTVYWMAPEVVNPKTTY<br>GPEADIWSLGCTVLEMLTRQLPYPGLEWTQALYRIGKGEPPAIPNCLSRDARDFISQCVKPNPQDRPSAAKLLEHPFV
OsMAPKKK29	IRYNEVLGRGAMKTVYKAFDEVEGIEVAWSQVEIDEVMQSPDNLERLYSEVHLLKSLKHENVMKFYNYWVDDQKKTINVITELFTS<br>GSLRQYRQKHPRVDLKAIKNWARQVLRGLDYLHTHQPPIIHRDLKCDNIFVNGNHGEVKIGDLGLATVMLTPRAKSVIGTPEFMAPEL<br>YDENYDELVDIYSFGMCMLEMFTLEYPYSECTNAAQIFKKVSKGVKPAALAKITNIQAKQFIDKCLVPASERLSAKELLQDPFL
OsMAPKKK30	LKFGTKVASGSNGDLFRGSYCSQDVAIKVVRPERISADMYRDFAQEVYIMRKVRHRNVVQFIGACTRQPNLYIVTDFMSGGSLHDYL<br>HKKNNSFKLSEILRVATDISKGMNYLHQNNIIHRDLKTANLLMDENKVVKVADFGVARVKDQSGVMTAETGTYRWMAPEVIEHKPY<br>DHKADVFSFGIVLWELLTGKIPYEYLTPLQAAIGVVQKGLRPTIPKDTHPKLSELLQKCWHRDPAERPDFSQILEIL
OsMAPKKK31	LVIRGVIARGTFGTVHRGVYDGQDVAVKMLDWGEDGHRSEREISSLRAAFAQEVAVWHKLDHPNVTKFIGAIMGARDLNIQTEHGH<br>FGMPSNICCVVVEYLAGGALKNFLIKNRRRKLAYKVVVQLALDLARGLSYLHSKKIVHRDVKTENMLLDKSRTVKIADFGVARIEAS<br>NPSDMTGETGTLGYMAPEVLNGHPYNRKCDVYSFGICLWEIYCCDMPYPDLSFSEVTSAVVRQNLRPEIPRCCPSSLANVMKRCWDA<br>NPDKRPAMAEVVSML
OsMAPKKK32	LHIGMPFAQGAFGKLYRGTYNGGDVAIKLLERPEADPEKAQLLEQQFVQEVMMLATLRHSNIVKFVGACRKPMVWCIVTEYAKGGS<br>VRNFLNRRQNRSVPLKLAVKQALDVARGMAYVHGLGFIHRDLKSDNLLISGDKSIKIADFGVARIEVKTEGMTPETGTYRWMAPEVI<br>QHRPYDQKVDVYSFGIVLWELVTGNLPFANMTAVQAAFAVVNKGVRPAIPHDCLPALAEIMTRCWDANPDARPPFTEVVRML
OsMAPKKK33	LDMGVPFAQGAFGKLYRGTYNGEDVAIKLLEKPENDPERAQALEQQFVQEVMMLSRLRHPNIVRFIGACRKSIVWCIITEYAKGGSV<br>RQFLARRQNKSVPLRLAVKQALDIARGMAYVHALGFIHRDLKSDNLLIAADKSIKIADFGVARIEVKTEGMTPETGTYRWMAPEMIQ<br>HRPYDHKVDVYSFGIVLWELITGMLPFTNMTAVQAAFAVVNKGARPVIPQDCLPALSHIMTLCWDANPEVRPAFTDIVCML
OsMAPKKK34	LVVRGVIARGTFGTVHRGVYDGHDVAVKLLDWGEDGHRSEQDIAALRAAFSQEVSVWHKLDHPNVTKFIGAIMGARDLDIQTESGH<br>LAMPSNICCVVVEYLAGGSLKGFLIKNRRKKLAFKVVVQIALDLARGLSYLHSKKIVHRDVKTENMLLDKTRTVKIADFGVARLEAS<br>NPSDMTGETGTLGYMAPEVLNGSPYNRKCDVYSFGICLWEIYCCDMPYPDLSFSEVTSAVVRQNLRPEMPRCCPSSLANVMKRCWD<br>ANPDKRPEMAEVVSML

OsMAPKKK35	LEEIRELGSGTYGAVYHGKWRGCDVAIKRIKASCFAGRPSERERLIADFWKEAQILSSLHHPNVVSFYGVVRDGPDGSLATVTEFMINGSLKQFLRKKDRTIDRRKRVILAMDAAFGMEYLHGKNIVHFDLKCENLLVNMRDPQRPICKIGDLGLSKVKQHTLVSGGVRGTLPWMAPELLSGKSNMVSEKIDVYSFGIVMWELLTGEEPYSDMRAAAIIGGIVNNSLRPQIPSWCDPEWKSLMENCWASEPADRPSFTEISQRL
OsMAPKKK36	GRYTEVLGKGAFKTVYKAFDQLEGLEVAWNQIKVGDILRNNDDLERLRSEVRLLKTLKHKNIIKFYNSWLDKKNNNINFITEVFTSGTLRQYRIKHKKVDVRALKKWSRQILSGLVYLHSHDPPVIHRDLKCDNIFVNGNQGEVKIGDLGLATILDNARSAHSIIGTPEFMAPELYDEEYNELVDIYAFGMCLLELVTFEYPYCECSNAAQIYKKVSDGEKPSSLAKIEDPEVRFFIEKCIAKASQRLSAQELLMDPFL
OsMAPKKK37	LHMGMPFAQGAFGKLYKGTYNGEDVAIKLLERPEADPERAGLMEQQFVQEVMMLATLRHPNIVKFIGACRKPMVWCIVTEYAKGGSVRQFLMKRQNRSVPLKLAVKQALDVARGMAYVHALGFIHRDLKSDNLLISGDKSIKIADFGVARIEVKTEGMTPETGTYRWMAPEMIQHRPYDQKVDVYSFGIVLWELITGMLPFANMTAVQAAFAVVNKGVRPAIPQDCLPVLSEIMTRCWDPNPDVRPPFTEVVRML
OsMAPKKK38	LDMGAPFAQGAFGKLYRGTYNGEDVAIKLLEKPENDPERAQLMEQQFVQEVMMLSTLRHPNIVRFIGACRKSIVWCIITEYAKGGSVRQFLARRQNKSVPLGLAVKQALDVARGMAYVHALRFIHRDLKSDNLLISADKSIKIADFGVARIEVQTEGMTPETGTYRWMAPEMIQHRPYDHKVDVYSFGIVLWELITGMLPFTNMTAVQAAFAVVNRGSRPAIPQDCVDSLSKIMTCCWDANPEVRPSFAEIVVML
OsMAPKKK39	LEELRELGSGTFGTVYHGKWRGSDVAIKRINDRCFAGKASEQERMRTDFWNEADKLASLHHPNVVAFYGVVLDGPGGSVATVTEYMANGSLRQALQRHEKIFDRRRRLLIAMDVAFGMEYLHEKNIVHFDLKSDNLLVNLRDPQHPICKVGDLGLSKVKCQTLISGGVRGTLPWMAPELLNGSSSLVSEKVDVFSFGIVMWELLTGEEPYAELHYGAIIGGIVNNTLRPPVPESCDPRWRSLMEQCWSSEPSERPSFTEVGKRL
OsMAPKKK40	LTIGTRVGIGFFGEVFRGIWNGTDVAIKVFLEQDLTTENMEDFCNEIYILSRLRHPNVILFLGACMVPPHLSMVTEYMEMGSLYYLIHMSGQKKKLSWRRRLKIVRDICRGLMCIHRMKIVHRDLKSANCLVNKHWTVKICDFGLSRVMTDSPMTDNSSAGTPEWMAPELIRNEPFTEKCDIFSLGVIMWELCTLSRPWDGISPVQVVYTVANEGSRLEIPEGPLGKLIADCWAEPQDRPSCQEILTRLL
OsMAPKKK41	LEELQELGSGTFGTVYHGKWRGSDVAIKRINDRCFAGKPSEQDKMRNDFWNEASKLADLHHPNVVAFYGVVLDGPGGSIATVTEYMVNGSLRTALLKNAKTLDKRKRLIIAMDTAFGMEYLHNKNIVHFDLKSDNLLVNLRDPQRPICKVGDLGLSKVKCQTLISGGVRGTLPWMAPELLNGSSSLVSEKVDVFSFGIVLWELLTGEEPYADLHYGVIIGGIVSNTLRPAVPDSCDPEWRSLMEQCWSTEPSERPTFTEIAGRL
OsMAPKKK42	LFIGSKIGEGAHGKVYKGKYGEQIVAIKVLNNGTTPEEKATLEARFIREVNMMCKVKHDNLVKFIGACKEPLMVIVSELLPGMSLKNYLNSLRPSQLDIHTAIGYALDIAHAMECLHANGIIHRDLKPDNLLLTANRKKLKLTDFGLAREETVTEMMTAETGTYRWMAPELYSTVTLQRGEKKHYTNKVDVYSFGIVLWELLTNKMPFEGMSNLQAAYAAAFKQARPPLPEETPQELVFIVQSCWVEDPAMRPSFSQIIRMLDAFL
OsMAPKKK43	LDIQEKVASGTYGDLYRGTYFGEDVAIKVLKSDRLNENMQEEFNEEVFIMRKIRHKNIVRFLGACTKSPTLCIVTEFMKNGSVYDYLHKRKGSFKLPSLLKAAVDISKGMNYLHQNKIIHRDLKTANLLMDEHELIKVADFGVARVKAESGIMTAETGTYRWMAPEVIEHKPYDSKADVFSFGVVLWELLTGKIPHEFLTPLQAAIGVVQEGLRPVIPKATDPKLALLLESCWQQNAVNRPDFVQILQKL
OsMAPKKK44	ITVGTRVGIGFFGEVFRGIWNGTDVAIKLFLEQDLTTENMEDFCNEISILSRLRHPNVILFLGACMKPPHLSLVTEYMEMGSLYYLIHASGQKGKLSWRRRLKMLRDICRGLMCMHRLKIVHRDLKSANCLVNKHWAVKLCDFGLSRVMSNSAMNDNSSAGTPEWMAPELIRNEPFTEKCDIFSLGVIMWELCTLSRPWEGIPSVQVVYNVANEGARLEIPDGPLGSLIADCWAEPDKRPGCQEILTRLL
OsMAPKKK45	LFIGNKFASGANSRIYRGIYKQRAVAVKMVRIPERDEARRAVLEDQFNSEVAFLSRLYHPNIVQFIAACKKPPVYCIITEYMSQGTLRMYLNKKDPYSLSSETILKLALDISRGMEYLHAQGVIHRDLKSQNLLLNDEMRVKVADFGTSCLETACQATKGNKGTYRWMAPEMTKEKPYTRKVDVYSFGIVLWELTTCLLPFQGMTPVQAAYAASEKNLRPPLSTSCSPVLNNLIKRCWSANPARRPEFSYIVSVL
OsMAPKKK46	LRYREIIGSGSSKTVYKAFDAVDGIEVAWGKVEINERIMGSSKELQRLRTEIQLLKSLQHKHILKLYASWVDTNRRTVNIVTELFTSGNLREYRTKHKKVDMKAMRRWAKQILTGLEYLHSQKPPIIHRDLKCDNIFINGNHGKVKIGDFGLAMVMQQRKTRSIQGTIEFMAPELFGENYNELVDIYSFGMCMLEMVTCECPYSECKGFIQIYKKITEGVKPAALSKVKDAEVRGFIESCLASVSDRLPASELLKSPFL
OsMAPKKK47	GRFNEILGKGSSKIVYRGFDEWRGVEVAWNQVRLRDVVRGGGELERFYGEVHLLAALRHRGIVRLHAYWVDAPRRALNFVTELFVSGTLRQYRERHRRVSAAAVRRWCAQILDGLAYLHAHSPPIIHRDLKCDNIFVNGNQGEVKIGDLGLAAFRRGGGHARCVGTPEFMAPEVYDESYDELADVYSFGMCVLEMVTLDYPYSECSNPIQIYKRVISGIKPAALYRVSDPVVRQFIERCLAPAARRPAARELLDDPFL
OsMAPKKK48	LEIRTKFATGRHSRVYSGRYAARDVAIKMVSQPEEDAALAAELERQFASEVALLLRLRHPNIISFVAACKKPPVFCIITEYMAGGSLRKYLHQQEPHSVPIELVLKLSLEIARGMSYLHSQGILHRDLKSENILLDGDMSVKVADFGISCLESQCGSGKGFTGTYRWMAPEMIKEKHHTRKVDVYSFGIVLWEILTALVPFSEMTPEQAAVAVALKNARPPLPPSCPVAISHLITQCWATNPDRRPQFDDIVAIL
OsMAPKKK49	LLIGQRFASGAYSRLFHGIYKEQPVAVKFIRQPDEEEDAELAAKLEKQFTAEVTILARLHHRNVIKLIGACNAPPVFCVITEFLCGGSLRAFLRKLQRQKLPLEKIICIALDIAHGLEYIHSQRVIHRDVKPENILFDGECCAKVVDFGVACEEVYCNSLEDDPGTYRWMAPEMYKRKPYGRKVDVYSFGLVLWELFSGSIPYEEMTPLQAAFAVVNKNLRPVVPSSCPAQLRLLIEQCWSCQPEKRPEFSQVVQIL
OsMAPKKK50	GRLSEVLGKGAMKTVYRGFDELRGVEVAWNQATISDVLRTPDALHRMYAEVSLLADLRHDAIIAFHASWVHPSRRTFNFITELFSSGTLRSYRLRYPRVSRRAVAAWARAILRGLAYLHSRGVIHRDLKCDNIFVNGHLGQVKIGDLGLAAVLRGCTSARSVIGTPEFMAPEMYDECYGVGVDVYSFGMCMLEMLTNEYPYSECDNPAQIYKKVTAGKLPDAFYLLTDADARRFIGRCLVDAAHRPSAEELLLDPFL
OsMAPKKK51	LLIGHRFASGAHSRLFHGIYKEQPVAVKFIRQPEDEEDAELAAQLEKQFNTEVTTLSRLNHPNVIKLIGACSSPPVFCVITEFLSGGSLRTFLHKQEHKSLPLEKIISIGLDIANGIGYIHSQGVVHRDVKPENIIFDSEFCAKIVDFGISCEEAECDPLANDTGTFRWMAPEMMKHKPYGRKVDVYSFGLILWEMFTGSVPYEDLNPFQAAFAVFDKNERPVIPSSCPAPLRLLIEQCWASQPDKRPEFWQIVQIL
OsMAPKKK52	IRYDEIVGSGAVKTVYKAFDKLEGVEVAWSQSRIDDSVMGSSKKMKQLNTEIQLLKTLKHKNIEKMFASWVDGEKKTVNIITELFTSGSLTQYRRKHKKVNMKAMKRWAIQILTGLEYLHSQKPAIIHRDLKCDNIFINGNHGKVKIGDFGLATFMQQQKKSIKGTLEFMAPELLTGHYNELVDIYSFGMCMLEMVTCEYPYSECQGMAHIFKKIDEGKKPAAFYKIKDAEVRSFIENCLAPVENRMSATELLKSSFL

OsMAPKKK53	GRLSEVLGKGAMKTVYRGFDELRGVEVAWNQATISDVLRTPDALHRMYAEVSLLADLRHDAIIAFHASWVHPSRRTFNFITELFSSGT<br>LRSYRLRYPRVSRRAVAAWARAILRGLAYLHARGVIHRDLKCDNIFVNGHLGQVKIGDLGLAAVLRGCASARSVIGTPEFMAPEMYD<br>ECYGVGVDVYSFGMCMLEMLTNEYPYSECDNPAQIYKKVTAGKLPDAFYRLTDADARRFIGRCLVDAAHRPSAEELLLDPFL
OsMAPKKK54	LEDLREIGSGSFGTVFHGRWKGTDVAIKRIKNSCFMYPSSQADKLITEFWREAAIISKLHHPNVLALYGIVNNGPGGTLATVTEFMING<br>SLKKVLLHKNKYLDWRKRIMVAKDAAIGMEYLHSKDIVHFDLKCDNLLVNIKDPSRPICKVADFGLSKMKQATLVSGGMRGTLPW<br>MAPELLTMSGTKVSEKIDVYSFGIVMWEILTGEDPYDGMHYGGVIGGILSNTLRPLVPTSCNLEWRKLMEQCWSTEPERRPSFAEVAT<br>RL
OsMAPKKK55	WTRVRTLGRGASGAVVSLAADDRSGALFAVKSAAAAAAAEQLVREGRILSGLRSPHVLPCLGFRAEAGGECQLFLEFAPGGSLADVV<br>ARSGGRLDECAIRAYAADVARGLAYLHGMSLVHGDVKGRNVVVGADGRAKIADFGCARTVGSDRPIGGTPAFMAPEVARGEEQEP<br>AADVWALGCTVIEMATGRAPWSDMEDILSAVRRIGYTDAVPEVPEWLSAEAKDFLARCFARNPRERWTSSQLLEHPFL
OsMAPKKK56	GRYTEVLGKGAFKTVYKAFDQLEGLEVAWNQIKVGDILRNNDDLERLRSEVRLLKTLKHKNIIKFYNSWLDKKNNNINFITEVFTSGT<br>LRQYRIKHKKVDVRALKKWSRQILSGLVYLHSHDPPVIHRDLKCDNIFVNGNQGEVKIGDLGLATILDNARSAHSIIGTPEFMAPELYD<br>EEYNELVDIYAFGMCLLELVTFEYPYCECSNAAQIYKKVSDGEKPSSLAKIEDPEVRFFIEKCIAKASQRLSAQELLMDPFL
OsMAPKKK57	LTRVRTLGRGASGAVVSLFAAGDDELLAVKSAAGPAGAAQLRREAGILASLCSPHVLPCFGFGAVAGGEYGLLLEFAPGGSLADEVA<br>RNGGRLEEDDVRAYAADVASGLAYLHGVGMVHGDVKGRNVVIGANGRAKLADFGCARRADSAGPIGGTPAFMAPEVARGEEQGP<br>AADVWALGCTVIEMATGRAPWSGVDDVVAAVRLIGFTDAVPEPPEWLSPEANDFLDKCLRRRAGERWTAAQLLEHPFL
OsMAPKKK58	LLLGPKIASGSNSRIHRGMYGEQPVAVKIMHAPVGDDDDDVQVRREMEAQFDAEVSLLSRLRHPNVVRLVGVCREPEVYWIITELMR<br>RGTLSAYLHGREPYSLPPETIVRLALDVARGMEYLHARGVVHRDLKPENLMLDGGGRVKVADLGTSCLEATCRGDKCSSKAGTFRW<br>MAPEMIHDKRCNRKVDVYSFGLVLWELTTCLVPFQNLSPVQVAYSVCDRDARPPLSPSCPPAINSLIKRCWSTEPARRPEFKQIVSVL
OsMAPKKK59	LEIGHVVEHGDHGTLFRGKYYSQDVAVKLLDWGAEGDSSEDQIAHFRTSLKEVVAVWHEFNHPNITKFIGASMGTTNLNIPKDIPDHS<br>SRKGARTDLPDRACCVVVEYLTGGTLKQHLIKHYRKNKKLLYEEVVRLALDLARGLSFLHSKKIVHRDVKSENMLLDPQLNLKIADF<br>GVARLVEAQDPKDLTRTTGTLGYMAPEVLDGKPYNRKCDVYSFGICLWETYCCDMPYGPYSDLSFADFSSFVVHKNLRPEIPDCCPS<br>AMASIMRRCWDANPEVRPEMEEVVRLLESL
OsMAPKKK60	LDIQNQVAHGTFGVVYRGTYDGHDVAVKVLDWGQEGQESTAKHREAFEKEVAVWQKLDHPNVTKFVGASMGTSHLKIPSAKAESR<br>SSSVGGGSAGGGGGQRCVVVVEYQHGGTLKTLLYKHRDKKLPYKKVVQLALDMARGLRYLHGEKIVHRDVKAENMLLDRKKTLK<br>IADFGVARVEAGADGDDMTGQTGTIGYMAPEVLQGRAYDHKCDVYSFGVLLWETYCCAMAYPNYSLADISYHVDKLGIRPDIPRCC<br>PKAMADIMARCWDANPDNRPEMSEVVALL
OsMAPKKK61	LLVGHKFASGAYSRLYKGLYDDKPVAIKFIRQPDDDDNGKMAAKLEKQYNSEVNALSHLYHKNVIKLVAAYKCPPVFYIITEFLPGG<br>SLRSYLNSTEHHPIPLEKIISIALDVACGLEYIHSQGVVHRDIKPENILFDENFCVKIADFGIACEESMCDVLVEDEGTYRWMAPEMIKR<br>KAYNRKVDVYSFGLLLWEMISGRIPFDDLTPLQAAYAVATRHARPVIPPECPMALRPLIEQCCSLQPEKRPDFWQIVKIL
OsMAPKKK62	LRRLRTLGRGASGAVVWLASDDASGELLAVKSAAGEGGAEQLRREGRVMSGLCSPHIVPCLGSRAAAGGEYQLFLEFAPGGSLADE<br>AARSGGRLAERAISAYAADVARALAYLHGNSLVHGDVKARNIMVGADGRAKLADFGCARRTDSERPIGGTPAFMAPEVARGEEQGP<br>AADVWALGCTIIEMATGRVPWSDMDDVFSAVHRIGYTDAVPEIPEWLSPEAKNFLSRCFTRNPSDRPTAAQLLEHPFL
OsMAPKKK63	WTRLRTLGRGASGAVVSLAEDGASGELFAVKTAAAAEAAMLRRERGMMSGLSSPHVVPCIGGGDGPDGSYNLFLEFAPGGSLANEV<br>ARDGGRLEERAIRVYAADVLRGLTYLHGMSLVHGDVKADNIVIGVDGLAKLADFGCAKTMDSERPVSGTPAFMAPEVARGEEQGPA<br>ADVWALGCTVIEMATGRAPWSDMDDVLAAVHRIGYTDAVPEVPVWLSAEAKDFLAMCFARNAGDRSTAAQLLEHPFV
OsMAPKKK64	GRYADVLGLGSVKKVYRGFDQEEGIEVAWNRVRLRALADRDPAMVERLHAEVRLLRSLHHEHIIGFHKVWLDRDAGVLNFITEVCT<br>SGSLREYRDRHRHVSVKALKKWARQILLGLDHLHTHDPCIIHRDLNCSNVFINGNTGQVKIGDLGLAAIVDKTHVAHTILGTPEFMAP<br>ELYTETYTESVDIYSYGMCVLEMVTREMPYAECDSVVQIYHSVTRGVPPAALKRIRDPELRAFIERCIGQPRNRPSAAELLRDPFF
OsMAPKKK65	FSKAVIIGKGSFGEILKANWRGTPIAVKRILPSLSDDRLVIQDFKHEVNLLIKLRHPNIVQFLGAVTETKPLMLVTEFLRGGDLHQYLKE<br>KGALAPATAVNFALDIARGMAYLHNEPNVVIHRDLKPRNILLVNSAANHLKVGDFGLSKIIKAQHANDVYKMTGETGSYRYMAPEV<br>FKHRKYDKKVDVFSFAMILYEMLEGDPPFSNYEPYEAAKYVGEGHRPPFRSKGFTNELKELIELCWSGDIHLRPSFLEILKRL
OsMAPKKK66	WTRLRSIGHGASGATVSLAADDASGELFVVKSAGDAVAATARQQLRREWSVMSGLSSPHVLRCLGFVQAAAGAGGEHQLLLEYAP<br>GGSLADVVARNGDRLDESAFRAYAADVLRGLDYLHEKLVVHGDVKGSNVLVGADGRAKLADFGCARVVMPGGSKQPVLGGTPAF<br>MAPEVARGEEQGPAADVWALGCTVIEMATGRAPWSDMDDVLAALRMIGYTDAVPDLPPWLSPEAKDFLRRCMQRRAGDRPTAAQ<br>LLQHPFV
OsMAPKKK67	WTRLRSVGRGASGAVVSLAANDVSGELFVIKSAGEGAARQQLRREWSVMSGLSSPHVLKCLGFVQASGGCGGGEHQLFLEYAPGGS<br>LADVVARNGGRLDEGAVRTYAADVLRGLDYLHGKLVVHGDVKGSNVLVGADGRAKLTDFGCARVAMPGGSKQPVLGGTPAFMA<br>PEVARGEEQGLAADVWALGCTVIEMATGRAPWSDMDNVLPALHKIGYTDAVPDLPRWLSPEAKDFLRGCLQRRAGDRPTAAQLLQ<br>HPFI
OsMAPKKK68	LSIRSVVAQGYHGTLFRADYGGHDVAVKVLDWREDGYSTPEQIAHLRASLADLAAVWHSFEHPNVARFFGASMGTTDLNIPAAASA<br>AGGGEQRNTGEKPPADRACCVVVEFLGGGTLKKYLIEHYRSKLPYGEVVRLALSMARGLSFLHANNIVHRDVKTENMLFLGGGGGG<br>DLKIADFGVARVEARDPREMTGATGTVGYMAPEVLVGKPYNRKCDVYSFGICLWETYCCEMPFTFGLSVAEASAAVAQRGMRPPIP<br>PCCPPAMARVMARCWDADPAARPEMEEVVRMLEAL
OsMAPKKK69	WTRVRTLGRGASGAEVFLAADDASGELFAVKSVGAAGAAALRREQGVMAGLSSPHVVPCIGGRVGRDGSYQMFLEFAPGGSLADV<br>AARCGGRMEECAVGEYAVDVARGLAYLHGMGLVHGDVKARNVVIGGDGRAKLADFGCARWADSGRPIGGTPAFMAPEVARGEEQ<br>SPAADVWALGCTVIEMATGRAPWSDMDDVLAAVHRIGYTEAVPEVPGWLSADAKDFLARCLQRRPIDRSTAAQLLEHPFV
OsMAPKKK70	LRRVRTLGRGASGAVVWLASDDDSGELMAVKSASAGGAAAQLRREGRVLSGLCSPHIVPCLGSRAAAGGEYQLFLEFAPGGSLADE

	AARNGGCLPEPAIRAYAADVARGLAYLHGNSLVHGDVKARNVVIGSDGRARLTDFGCARVMDSAGPIGGTPAFMAPEVARGEEQGP AADVWALGCTIIEMATGRAPWSDMDDILAAVHRIGYTNAVPEVPGWLSAEAKDFLDGCFERNASDRSTAAQLLEHPFV
OsMAPKKK71	WRRGPVIGRGATATVSIATDRRTGGVFAVKSVDVARAGALRREQGMLSALASPFVVPCVGSGVSAAVDGSGGACYDLFLEYAPGGS LADEIKRCGGRCEEPLIRSRVGDVLRGLAYVHAAGIAHCDVKGRNVLVGADGRAMLADFGCARWMAAEDCNAGGVTIRGTPMFLA PEAARGEAQGTAADIWALGCTVIEMATGGAPWPRFADPVAALHHVAHSVDVPESPAWFSAEGKDFLARCLIRDPAKRWTAEQLLEH PFV
OsMAPKKK72	FTNGNGISKGTFRKATWRGILVAVKKLDDDLIMDENKVQAFRDELDVLQLIRHPNVVQFLGAVTQSSPMMIVMEFMPKGDLRKHLS RKGALEPSYAVKLALDIARGMNYLHEHKPQAIIHRDLEPSNILRDDTGHLKVADFDLCKMLKWRRKVREEKAVTSPGNACRYVAPE VLRNEEYDTKVDVFSFALILQEMIEGCLPFYDKKNNEIEKAHNSKERPPFRAPPKHYAYGLRELIEQCWSENPASRPDFRTIIEQLSYI
OsMAPKKK73	WVRGKCVGRGAFGAVHVAVDRATGRAFAVKSVEAKGGAPAAAMACLESEIRILRRLSSPYVVEYLGDDGDAATTRNLHMELVPGG SAAAAAAMGGLGERGARGVVRRVAAALRYLHDVAGVVHGDVKGRNVLVGCDGDGRGAKLADFGAARLVSDAAVSRGPRGTPA WMAPEVARGGAPTPASDVWSLGCTAVELITGKRPWSELGGASEVGELLFLIGFGGKRPELPACASDSCRDFLDKCLRRDAGERWTCD QLLRHPFL
OsMAPKKK74	FKKGDEVVKGVYQVAKWNGTKVHVKILDRECYCDQEVINSFRHELTVLEKVRHPNVVQFVGAVTQNIPMMIISEYLPNGDLSSCIPR KGKLHGQKVLKYGLEIARGMTYLHQCKPDPIIHCDLKPKNIFLDSGGQLKIAGFGLTRLSKISPGRVKLADHESMVDSFSHYTAPELYR NEIFDASVDAFSFGFILYEMVEGTHTVHGKSSEESGHTIRYDGMRPSLKNKLRGYPPDFKALIEECWDTQGIARPTFSEIIIRL
OsMAPKKK75	FRRGEEVTKGHYVARWYGSKVFVKILDKDSFSDANSINEFKHELTLLEKARHPNLVQFVGAVTQNVPMMIVSEYHQKGDLASYLET KGRLQPYKAIRFSLDIARGLNYLHECKPEPIIHGNLSTKSIVRDDEGKLKVAGFGSRSLIKVSEDNPQMDQTTSKFNSVYTAPEMYRNG TFDRSVDVFAFGLILYEMIEGTHAFHPKPPEEAAKMICLEGMRPPFKNKPKYYPDDLRELIQECWDPTPSVRPTFEEIIVRLNKISTSF
