# Supplementary material for: Genome-wide exploration of the molecular evolution and regulatory network of mitogen-activated protein kinase cascades upon multiple stresses in Brachypodium distachyon
Source: BMC Genomics. 2015 Mar 24;16(1):228. doi: 10.1186/s12864-015-1452-1 (PMC4404688; doi:10.1186/s12864-015-1452-1)
Supplement: Additional file 5: — Orthologs of MAPK cascade kinase genes among B. distachyon, A. thaliana and O. sativa. [file 12864_2015_1452_MOESM5_ESM.pdf]

Additional file 5. Orthologs of MAPK cascade kinase genes among *B. distachyon, A. thaliana and O. sativa*

	B. distachyon genes		*O. sativa* genes		*Arabidopsis* genes	
	gene name	gene model	gene name	gene model	gene name	gene model
MAPK	BdMPK3	Bradi1g65810	OsMPK3	LOC_Os03g17700	AtMPK3	At3g45640
	BdMPK4	Bradi3g32000	OsMPK4	LOC_Os10g38950	AtMPK4	At4g01370
	BdMPK6	Bradi1g49100	OsMPK6	LOC_Os06g06090	AtMPK6	At2g43790
	BdMPK7-1<br>BdMPK7-2	Bradi1g34030<br>Bradi4g24912	OsMPK7	LOC_Os06g48590	N/A	N/A
	BdMPK11	Bradi3g16560	OsMPK2	LOC_Os08g06060	AtMPK4<br>AtMPK5<br>AtMPK11	At4g01370<br>At4g11330<br>At1g01560
	BdMPK14	Bradi3g03780	OsMPK14	LOC_Os02g05480	AtMPK1<br>AtMAPK2	At1g10210<br>At1g59580
	BdMPK16	Bradi2g36470	OsMPK15<br>OsMPK16	LOC_Os05g05160<br>LOC_Os11g17080	AtMPK16	At5g19010
	BdMPK17	Bradi1g34700	OsMPK17-1<br>OsMPK17-2	LOC_Os06g49430<br>LOC_Os02g04230	N/A	N/A
	BdMPK20-1	Bradi2g44350	OsMPK20-1	LOC_Os01g43910	N/A	N/A
	BdMPK20-2	Bradi2g15317	OsMPK20-2	LOC_Os05g50560	N/A	N/A
	BdMPK20-3	Bradi1g41780	OsMPK20-3	LOC_Os06g26340	AtMPK20	At2g42880
	BdMPK20-4	Bradi2g45870	OsMPK20-4	LOC_Os01g47530	N/A	N/A
	BdMPK20-5	Bradi2g16337	OsMPK20-5	LOC_Os05g49140	N/A	N/A
	BdMPK21-1	Bradi2g15620	OsMPK21-1	LOC_Os05g50120	N/A	N/A
	BdMPK21-2	Bradi2g45010	OsMPK21-2	LOC_Os01g45620	N/A	N/A
MAPKK	BdMKK1	Bradi1g51000	OsMKK1	LOC_Os06g05520	AtMKK1<br>AtMKK2	At4g26070<br>At4g29810
	BdMKK3-1<br>BdMKK3-2<br>BdMKK3-3	Bradi4g39490<br>Bradi1g41860<br>Bradi3g11260	OsMKK3	LOC_Os06g27890	AtMKK3	At5g40440
	BdMKK4	Bradi3g53650	OsMKK4	LOC_Os02g54600	AtMKK4	At1g51660
	BdMKK5	Bradi1g46880	OsMKK5	LOC_Os06g09180	AtMKK5	At3g21220
	BdMKK6	Bradi1g75150	OsMKK6	LOC_Os01g32660	AtMKK6	At5g56580
	BdMKK10-1	Bradi1g11525	OsMKK10-3	LOC_Os03g50550	N/A	N/A
	BdMKK10-2	Bradi1g69400	OsMKK10-2	LOC_Os03g12390	N/A	N/A
	BdMKK10-3	Bradi1g10800	OsMKK10-3	LOC_Os03g50550	N/A	N/A
MAPKKK	BdMAPKKK1	Bradi5g24870	OsMAPKKK24	LOC_Os04g56530	AtMAPKKK6<br>AtMAPKKK7	At3g07980<br>At3g13530
	BdMAPKKK2	Bradi1g28950	OsMAPKKK21	LOC_Os07g25680	N/A	N/A
	BdMAPKKK3	Bradi3g60210	OsMAPKKK35	LOC_Os02g54510	AtRaf35	At5g57610
	BdMAPKKK4	Bradi1g47570	OsMAPKKK39	LOC_Os06g08280	N/A	N/A
	BdMAPKKK5	Bradi3g59510	OsMAPKKK6	LOC_Os02g50970	AtRaf5	At1g73660

BdMAPKKK6	Bradi1g74480	OsMAPKKK1	LOC_Os03g06410	AtEDR1	At1g08720
BdMAPKKK7	Bradi1g45040	OsMAPKKK7	LOC_Os06g12590	AtRaf4	At1g18160
BdMAPKKK8	Bradi5g18180	OsMAPKKK10	LOC_Os04g47240	AtMAPKKK4	At1g63700
BdMAPKKK9	Bradi1g30720	OsMAPKKK41	LOC_Os06g43840	AtRaf42	At3g46920
BdMAPKKK10	Bradi2g46340	OsMAPKKK40	LOC_Os01g48330	AtRaf15	At3g58640
BdMAPKKK11	Bradi3g51380	OsMAPKKK9	LOC_Os02g44642	AtMAPKKK4	At1g63700
BdMAPKKK12	Bradi3g27120	OsMAPKKK2	LOC_Os10g29540	AtEDR1	At1g08720
BdMAPKKK13	Bradi3g09170	OsMAPKKK44	LOC_Os02g14530	AtRaf13	At2g31010
BdMAPKKK14	Bradi3g08260	OsMAPKKK4	LOC_Os02g12810	AtRaf3	At5g11850
BdMAPKKK15	Bradi4g04470	OsMAPKKK5	LOC_Os12g37570	N/A	N/A
BdMAPKKK16	Bradi1g23970	OsMAPKKK20	LOC_Os07g38530	AtZIK4	AT3G04910
BdMAPKKK17	Bradi4g38400	OsMAPKKK12	LOC_Os09g39320	AtCTR1	At5g03730
BdMAPKKK18	Bradi3g44710	OsMAPKKK3	LOC_Os02g32610	AtCTR1	At5g03730
BdMAPKKK19	Bradi1g07650	OsMAPKKK18	LOC_Os03g55560	AtMAPKKK5	At5g66850
BdMAPKKK20	Bradi5g21330	OsMAPKKK14	LOC_Os04g52140	AtRaf6	At4g24480
BdMAPKKK21	Bradi4g36880	OsMAPKKK17	LOC_Os09g37230	AtRaf30	At4g38470
BdMAPKKK22	Bradi2g39350	OsMAPKKK36<br>OsMAPKKK56	LOC_Os05g01780<br>LOC_Os05g01780	AtZIK11	AT3G48260
BdMAPKKK23	Bradi4g29500	OsMAPKKK13	LOC_Os09g21510	N/A	N/A
BdMAPKKK24	Bradi1g60340	OsMAPKKK54	LOC_Os03g28300	N/A	N/A
BdMAPKKK25	Bradi3g36080	OsMAPKKK15	LOC_Os08g32600	N/A	N/A
BdMAPKKK26	Bradi1g58810	OsMAPKKK11	LOC_Os07g02780	AtMAPKKK5	At5g66850
BdMAPKKK27	Bradi4g22760	OsMAPKKK8	LOC_Os11g10100	AtMAPKKK3	At1g53570
BdMAPKKK28	Bradi3g51460	OsMAPKKK29	LOC_Os02g45130	N/A	N/A
BdMAPKKK29	Bradi1g10970	OsMAPKKK22	LOC_Os03g49640	N/A	N/A
BdMAPKKK30	Bradi3g01850	OsMAPKKK30	LOC_Os02g02780	N/A	N/A
BdMAPKKK31	Bradi2g06260	OsMAPKKK61	LOC_Os01g10450	AtRaf36	At5g58950
BdMAPKKK32	Bradi2g19590	OsMAPKKK49	LOC_Os05g44290	AtRaf43	At3g46930
BdMAPKKK33	Bradi3g48360	OsMAPKKK75	LOC_Os02g39560	N/A	N/A
BdMAPKKK34	Bradi1g67400	OsMAPKKK28	LOC_Os03g15570	N/A	N/A
BdMAPKKK35	Bradi2g49700	OsMAPKKK51	LOC_Os01g54350	N/A	N/A
BdMAPKKK36	Bradi2g57470	OsMAPKKK74	LOC_Os01g66860	N/A	N/A
BdMAPKKK37	Bradi3g05520	OsMAPKKK33	LOC_Os02g07790	AtRaf22	At2g24360
BdMAPKKK38	Bradi3g18150	OsMAPKKK32	LOC_Os08g12750	AtRaf28	At4g31170
BdMAPKKK39	Bradi1g28110	OsMAPKKK26	LOC_Os07g29330	N/A	N/A
BdMAPKKK40	Bradi2g49790	OsMAPKKK72	LOC_Os01g54480	N/A	N/A
BdMAPKKK41	Bradi1g14000	OsMAPKKK27	LOC_Os03g43760	N/A	N/A
BdMAPKKK42	Bradi1g35350	OsMAPKKK45	LOC_Os06g43030	AtRaf19	At1g62400
BdMAPKKK43	Bradi1g04080	OsMAPKKK42	LOC_Os03g60150	AtRaf31	At5g01850
BdMAPKKK44	Bradi5g10670	OsMAPKKK16	LOC_Os04g35700	N/A	N/A
BdMAPKKK45	Bradi3g47600	OsMAPKKK25	LOC_Os02g38080	AtRaf32<br>AtRaf34	At5g40540<br>At5g50180

				AtRaf41	At3g27560
BdMAPKKK46	Bradi2g44910	OsMAPKKK31	LOC_Os01g45380	AtRaf33	At5g50000
BdMAPKKK47	Bradi2g15560			AtRaf38	At3g01490
BdMAPKKK48	Bradi2g00670	OsMAPKKK48	LOC_Os01g01740	AtRaf19	At1g62400
BdMAPKKK49	Bradi1g20390	OsMAPKKK65	LOC_Os07g43900	AtRaf17	At1g14000
BdMAPKKK50	Bradi4g44430	OsMAPKKK50	LOC_Os12g02250	AtZIK1	AT3G51630
		OsMAPKKK53	LOC_Os11g02305	AtZIK2	AT5G58350
BdMAPKKK51	Bradi2g47510	OsMAPKKK62	LOC_Os01g50420	N/A	N/A
BdMAPKKK52	Bradi2g47480	OsMAPKKK63	LOC_Os01g50370	N/A	N/A
BdMAPKKK53	Bradi2g47490	OsMAPKKK55	LOC_Os01g50400	N/A	N/A
BdMAPKKK54	Bradi1g23320	OsMAPKKK64	LOC_Os07g39520	AtZIK8	AT5G55560
BdMAPKKK55	Bradi2g47500	OsMAPKKK70	LOC_Os01g50410	N/A	N/A
BdMAPKKK56	Bradi4g41940	OsMAPKKK52	LOC_Os12g06490	N/A	N/A
BdMAPKKK57	Bradi3g10890	OsMAPKKK71	LOC_Os02g21700	N/A	N/A
BdMAPKKK59	Bradi2g17830	OsMAPKKK57	LOC_Os05g46750	N/A	N/A
BdMAPKKK60	Bradi1g65500	OsMAPKKK73	LOC_Os03g18170	AtMAPKKK13	AT1G07150
				AtMAPKKK14	AT2G30040
BdMAPKKK64	Bradi4g02900	OsMAPKKK23	LOC_Os12g40279	AtRaf40	At3g24720
BdMAPKKK66	Bradi1g14010	OsMAPKKK27	LOC_Os03g43760	N/A	N/A
BdMAPKKK67	Bradi3g45790	OsMAPKKK19	LOC_Os02g35010	N/A	N/A
