# Supplementary material for: Genome-wide exploration of the molecular evolution and regulatory network of mitogen-activated protein kinase cascades upon multiple stresses in Brachypodium distachyon
Source: BMC Genomics. 2015 Mar 24;16(1):228. doi: 10.1186/s12864-015-1452-1 (PMC4404688; doi:10.1186/s12864-015-1452-1)
Supplement: Additional file 6: — Alignment of MAPKKKs from O. sativa, A. thaliana and B. distachyon. [file 12864_2015_1452_MOESM6_ESM.pdf]

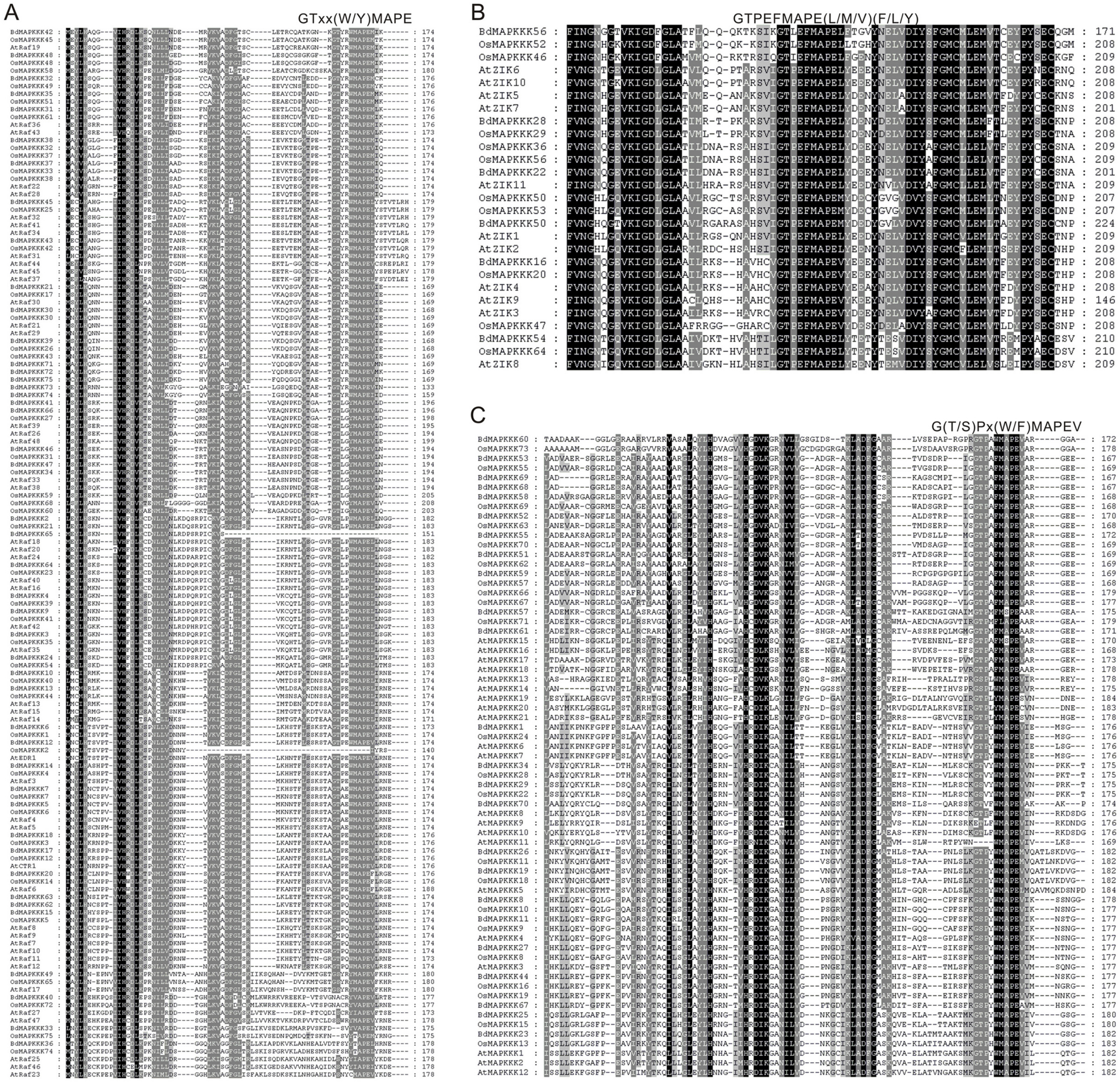
A
GTxx(W/Y)MAPE
B
GTPEFMAPE(L/M/V)(F/L/Y)
C
G(T/S)Px(W/F)MAPEV
