# Supplementary material for: Genome-wide exploration of the molecular evolution and regulatory network of mitogen-activated protein kinase cascades upon multiple stresses in Brachypodium distachyon
Source: BMC Genomics. 2015 Mar 24;16(1):228. doi: 10.1186/s12864-015-1452-1 (PMC4404688; doi:10.1186/s12864-015-1452-1)
Supplement: Additional file 7: — The duplicated genes in O. sativa, A. thaliana and B. distachyon chromosomes. [file 12864_2015_1452_MOESM7_ESM.pdf]

Additional file 7 The duplicated genes in *O. sativa, A. thaliana* and *B. distachyon* chromosomes.

Gene			Syntenic regions		
gene name	gene model	location	gene name	gene model	location
BdMPK3	Bradi1g65810	64739946-64742370	AtMPK3	At3g45640	16756918-16758476
BdMPK3	Bradi1g65810	64739946-64742370	OsMPK3	LOC_Os03g17700	9847700-9850473
BdMPK4	Bradi3g32000	34209148-34213422	OsMPK4	LOC_Os10g38950	20684835-20687729
BdMPK4	Bradi3g32000	34209262-34212267	AtMPK11	At1g01560	202345-204189
BdMPK6	Bradi1g49100	47836748-47843578	AtMPK6	AT2G43790	18138477-18140693
BdMPK6	Bradi1g49100	47836748-47843578	OsMPK6	LOC_Os06g06090	2806543-2813004
BdMPK7-1	Bradi1g34030	29632402-29634667	BdMPK14	Bradi3g03780	2521821-2525826
BdMPK7-1	Bradi1g34030	29632402-29634667	OsMPK7	LOC_Os06g48590	29398386-29402535
BdMPK11	Bradi3g16560	14742468-14745425	AtMPK4	At4g01370	567219-568889
BdMPK11	Bradi3g16560	14742468-14745425		LOC_Os08g06060	3307520-3310590
BdMPK14	Bradi3g03780	2521821-2525826	BdMPK7-1	Bradi1g34030	29632402-29634667
BdMPK14	Bradi3g03780	2521821-2525826	AtMPK1	At1g10210	3349579-3350776
BdMPK14	Bradi3g03780	2521821-2525826	OsMPK14	LOC_Os02g05480	2643056-2646360
BdMPK16	Bradi2g36470	36786589-36792652	AtMPK16	AT5G19010	6347676-6345096
BdMPK16	Bradi2g36470	36786589-36792652		LOC_Os05g05160	2511542-2518105
BdMPK17	Bradi1g34700	30307750-30312635	AtMPK9	AT3G18040	6174800-6178150
BdMPK17	Bradi1g34700	30307750-30312635	OsMPK17-1	LOC_Os06g49430	29949898-29954873
BdMPK20-1	Bradi2g44350	44852084-44858179	BdMPK20-4	Bradi2g45870	46302880-46308387
BdMPK20-1	Bradi2g44350	44852084-44858179		Bradi2g15320	13688732-13693325
BdMPK20-1	Bradi2g44350	44852084-44858179	AtMPK20	AT2G42880	17843947-17840572
BdMPK20-1	Bradi2g44350	44852084-44858179	OsMPK20-1	LOC_Os01g43910	25152108-25158338
BdMPK20-3	Bradi1g41780	38419711-38433437	OsMPK20-3	LOC_Os06g26340	15414829-15423582
BdMPK20-4	Bradi2g45870	46302880-46308387	OsMPK20-4	LOC_Os01g47530	27171290-27178237
BdMPK20-4	Bradi2g45870	46302880-46308387	BdMPK20-1	Bradi2g44350	44852084-44858179
BdMPK20-4	Bradi2g45870	46302880-46308387		Bradi2g16340	14412074-14416456
BdMPK20-4	Bradi2g45870	46302880-46308387		Bradi2g15320	13688732-13693325
BdMPK21-1	Bradi2g15620	13875717-13880863	BdMPK21-2	Bradi2g45010	45394037-45403577
BdMPK21-1	Bradi2g15620	13875717-13880863	OsMPK21-1	LOC_Os05g50120	28721012-28726493
BdMPK21-2	Bradi2g45010	45394037-45403577	BdMPK21-1	Bradi2g15620	13875717-13880863
BdMPK21-2	Bradi2g45010	45394037-45403577	OsMPK21-2	LOC_Os01g45620	25917636-25922976
AtMPK1	At1g10210	3349579-3350776	AtMPK2	At1g59580	21884521-21885743
AtMPK1	At1g10210	3349579-3350776	BdMPK14	Bradi3g03780	2521821-2525826
AtMPK2	At1g59580	21884521-21885743	AtMPK1	At1g10210	3349579-3350776
AtMPK3	At3g45640	16756918-16758476	BdMPK3	Bradi1g65810	64739946-64742370
AtMPK4	At4g01370	567219-568889	AtMPK11	At1g01560	202345-204189
AtMPK4	At4g01370	567219-568889	AtMPK12	At2g46070	18947770-18946134
AtMPK4	At4g01370	567219-568889	AtMPK5	At4g11330	6892143-6893845
AtMPK4	At4g01370	567219-568889	BdMPK11	Bradi3g16560	14742468-14745425
AtMPK5	At4g11330	6892143-6893845	AtMPK12	At2g46070	18947770-18946134
AtMPK5	At4g11330	6892143-6893845	AtMPK4	At4g01370	567219-568889
AtMPK6	At2g43790	18138477-18140693	AtMPK10	At3g59790	22092448-22094240
AtMPK6	AT2G43790	18138477-18140693	BdMPK6	Bradi1g49100	47836748-47843578
AtMPK7	At2g18170	7909374-7908178	AtMPK14	At4g36450	17211413-17210245

AtMPK8	At1g18150	6247582-6244641	AtMPK15	At1g73670	27700212-27703168
AtMPK9	At3g18040	6174800-6178150	AtMPK15	At1g73670	27700212-27703168
AtMPK9	AT3G18040	6174800-6178150	BdMPK17	Bradi1g34700	30307750-30312635
AtMPK10	At3g59790	22092448-22094240	AtMPK6	At2g43790	18138477-18140693
AtMPK11	At1g01560	202345-204189	AtMPK4	At4g01370	567219-568889
AtMPK11	At1g01560	202345-204189	AtMPK12	At2g46070	18947770-18946134
AtMPK11	AT1G01560	202345-204189	BdMPK4	Bradi3g32000	34209148-34213422
AtMPK12	At2g46070	18947770-18946134	AtMPK4	At4g01370	567219-568889
AtMPK12	At2g46070	18947770-18946134	AtMPK5	At4g11330	6892143-6893845
AtMPK12	At2g46070	18947770-18946134	AtMPK11	At1g01560	202345-204189
AtMPK14	At4g36450	17211413-17210245	AtMPK7	At2g18170	7909374-7908178
AtMPK15	At1g73670	27700212-27703168	AtMPK8	At1g18150	6247582-6244641
AtMPK15	At1g73670	27700212-27703168	AtMPK9	At3g18040	6174800-6178150
AtMPK16	AT5G19010	6347676-6345096	BdMPK16	Bradi2g36470	36786589-36792652
AtMPK18	At1g53510	19974158-19970961	AtMPK19	At3g14720	4946057-4948906
AtMPK19	At3g14720	4946057-4948906	AtMPK18	At1g53510	19974158-19970961
AtMPK20	AT2G42880	17843947-17840572	BdMPK20-1	Bradi2g44350	44852084-44858179
OsMPK3	LOC_Os03g17700	9847700-9850473	BdMPK3	Bradi1g65810	64739946-64742370
OsMPK4	LOC_Os10g38950	20684835-20687729	BdMPK4	Bradi3g32000	34209148-34213422
OsMPK6	LOC_Os06g06090	2806543-2813004	BdMPK6	Bradi1g49100	47836748-47843578
OsMPK7	LOC_Os06g48590	29398386-29402535	OsMPK14	LOC_Os02g05480	2643056-2646360
OsMPK7	LOC_Os06g48590	29398386-29402535	BdMPK7-1	Bradi1g34030	29632402-29634667
OsMPK14	LOC_Os02g05480	2643056-2646360	OsMPK7	LOC_Os06g48590	29398386-29402535
OsMPK14	LOC_Os02g05480	2643056-2646360	BdMPK14	Bradi3g03780	2521821-2525826
OsMPK17-1	LOC_Os06g49430	29949898-29954873	OsMPK17-2	LOC_Os02g04230	1850981-1858243
OsMPK17-1	LOC_Os06g49430	29949898-29954873	BdMPK17	Bradi1g34700	30307750-30312635
OsMPK17-2	LOC_Os02g04230	1850981-1858243	OsMPK17-1	LOC_Os06g49430	29949898-29954873
OsMPK20-1	LOC_Os01g43910	25152108-25158338	OsMPK20-4	LOC_Os01g47530	27171290-27178237
OsMPK20-1	LOC_Os01g43910	25152108-25158338	BdMPK20-1	Bradi2g44350	44852084-44858179
OsMPK20-3	LOC_Os06g26340	15414829-15423582	BdMPK20-3	Bradi1g41780	38419711-38433437
OsMPK20-4	LOC_Os01g47530	27171290-27178237	OsMPK20-5	LOC_Os05g49140	28188894-28194022
OsMPK20-4	LOC_Os01g47530	27171290-27178237	OsMPK20-1	LOC_Os01g43910	25152108-25158338
OsMPK20-4	LOC_Os01g47530	27171290-27178237	BdMPK20-4	Bradi2g45870	46302880-46308387
OsMPK20-5	LOC_Os05g49140	28188894-28194022	OsMPK20-4	LOC_Os01g47530	27171290-27178237
OsMPK21-1	LOC_Os05g50120	28721012-28726493	OsMPK21-2	LOC_Os01g45620	25917636-25922976
OsMPK21-1	LOC_Os05g50120	28721012-28726493	BdMPK21-1	Bradi2g15620	13875717-13880863
OsMPK21-2	LOC_Os01g45620	25917636-25922976	OsMPK21-1	LOC_Os05g50120	28721012-28726493
OsMPK21-2	LOC_Os01g45620	25917636-25922976	BdMPK21-2	Bradi2g45010	45394037-45403577
BdMKK1	Bradi1g51000	49443017-49446304	AtMKK2	At4g29810	14595241-14593299
BdMKK1	Bradi1g51000	49443110-49445886	OsMKK1	LOC_Os06g05520	2498863-2502887
BdMKK3-2	Bradi1g41860	38580168-38590500	OsMKK3	LOC_Os06g27890	15811009-15821885
BdMKK3-2	Bradi1g41860	38580168-38590500	AtMKK3	AT5G40440	16182149-16184513
BdMKK4	Bradi3g53650	54258852-54260320	BdMKK5	Bradi1g46880	45433080-45434736
BdMKK4	Bradi3g53650	54258852-54259925	OsMKK4	LOC_Os02g54600	33442070-33443948
BdMKK4	Bradi3g53650	54258852-54259925	OsMKK5	LOC_Os06g09180	4616608-4618145
BdMKK5	Bradi1g46880	45433080-45434736	OsMKK6	LOC_Os06g09180	4616608-4618145

BdMKK5	Bradi1g46880	45433080-45434736	BdMKK4	Bradi3g53650	54258852-54260320
BdMKK5	Bradi1g46880	45433080-45434736	AtMKK4	At1g51660	19154575-19155675
BdMKK5	Bradi1g46880	45434682-45433651	OsMKK4	LOC_Os02g54600	33442070-33443948
BdMKK5	Bradi1g46880	45434682-45433651	OsMKK5	LOC_Os06g09180	4616608-4618145
BdMKK6	Bradi1g75150	72118481-72123498	OsMKK5	LOC_Os01g32660	17904834-17910037
BdMKK6	Bradi1g75150	72118481-72123498	AtMKK6	At5g56580	22906620-22904851
BdMKK10-2	Bradi1g69400	67832493-67833815	OsMKK10-2	LOC_Os03g12390	6545153-6546478
BdMKK10-2	Bradi1g69400	67832493-67833815	OsMKK10-3	LOC_Os03g50550	28859516-28862894
BdMKK10-3	Bradi1g10800	7865287-7866321	OsMKK10-3	LOC_Os03g50550	28859516-28862894
BdMKK10-3	Bradi1g10800	7865287-7866321	AtMKK9	AT1G73500	27640351-27639419
BdMKK10-4	Bradi1g10770	7809375-7810400	OsMKK10-3	LOC_Os03g50550	28859516-28862894
AtMKK1	At4g26070	13217797-13219695	AtMKK2	At4g29810	14595241-14593299
AtMKK2	At4g29810	14595241-14593299	AtMKK1	At4g26070	13217797-13219695
AtMKK2	At4g29810	14595241-14593299	BdMKK1	Bradi1g51000	49443017-49446304
AtMKK3	At5g40440	16182149-16184513	BdMKK3-2	Bradi1g41860	38580168-38590500
AtMKK4	At1g51660	19154575-19155675	AtMKK5	AT3G21220	7445917-7446963
AtMKK4	At1g51660	19154575-19155675	BdMKK5	Bradi1g46880	45433080-45434736
AtMKK5	At3g21220	7445917-7446963	AtMKK4	At1g51660	19154575-19155675
AtMKK6	At5g56580	22906620-22904851	BdMKK6	Bradi1g75150	72118481-72123498
AtMKK7	At1g18350	6315686-6316609	AtMKK9	AT1G73500	27640351-27639419
AtMKK9	At1g73500	27640351-27639419	AtMKK7	At1g18350	6315686-6316609
AtMKK9	At1g73500	27640351-27639419	BdMKK10-3	Bradi1g10800	7865287-7866321
OsMKK1	LOC_Os06g05520	2498863-2502887	BdMKK1	Bradi1g51000	49443017-49446304
OsMKK3	LOC_Os06g27890	15811009-15821885	BdMKK3-2	Bradi1g41860	38580168-38590500
OsMKK4	LOC_Os02g54600	33442070-33443948	OsMKK5	LOC_Os06g09180	4616608-4618145
OsMKK4	LOC_Os02g54600	33442070-33443948	BdMKK4	Bradi3g53650	54258852-54259925
OsMKK4	LOC_Os02g54600	33442070-33443948	BdMKK5	Bradi1g46880	45434682-45433651
OsMKK5	LOC_Os06g09180	4616608-4618145	BdMKK5	Bradi1g46880	45434682-45433651
OsMKK5	LOC_Os06g09180	4616608-4618145	BdMKK4	Bradi3g53650	54258852-54260320
OsMKK5	LOC_Os01g32660	17904834-17910037	BdMKK6	Bradi1g75150	72118481-72123498
OsMKK5	LOC_Os06g09180	4616608-4618145	OsMKK4	LOC_Os02g54600	33442070-33443948
OsMKK6	LOC_Os06g09180	4616608-4618145	BdMKK5	Bradi1g46880	45433080-45434736
OsMKK10-2	LOC_Os03g12390	6545153-6546478	OsMKK10-3	LOC_Os03g50550	28859516-28862894
OsMKK10-2	LOC_Os03g12390	6545153-6546478	BdMKK10-2	Bradi1g69400	67832545-67833567
OsMKK10-3	LOC_Os03g50550	28859516-28862894	OsMKK10-2	LOC_Os03g12390	6545153-6546478
OsMKK10-3	LOC_Os03g50550	28859516-28862894	BdMKK10-4	Bradi1g10770	7809375-7810400
OsMKK10-3	LOC_Os03g50550	28859516-28862894	BdMKK10-2	Bradi1g69400	67832493-67833815
OsMKK10-3	LOC_Os03g50550	28859516-28862894	BdMKK10-3	Bradi1g10800	7865287-7866321
BdMAPKKK1	Bradi5g24870	26381665-26393219	OsMAPKKK24	LOC_Os04g56530	33707455-33719236
BdMAPKKK2	Bradi1g28950	24311845-24322003	OsMAPKKK21	LOC_Os07g25680	14723111-14727159
BdMAPKKK3	Bradi3g60210	59221921-59227497	OsMAPKKK35	LOC_Os02g54510	33387811-33392575
BdMAPKKK4	Bradi1g47570	46211026-46216334	OsMAPKKK39	LOC_Os06g08280	4008139-4013539
BdMAPKKK5	Bradi3g59510	58646176-58654454	OsMAPKKK7	LOC_Os06g12590	6823036-6832862
BdMAPKKK7	Bradi1g45040	43232910-43245317	OsMAPKKK7	LOC_Os06g12590	6823036-6832862
BdMAPKKK10	Bradi2g46340	46786767-46795821	OsMAPKKK40	LOC_Os01g48330	27702476-27692557
BdMAPKKK15	Bradi4g04470	3687248-3696919	OsMAPKKK5	LOC_Os12g37570	23061181-23068420

BdMAPKKK16	Bradi1g23970	19257221-19260591	OsMAPKKK20	LOC_Os07g38530	23145979-23149609
BdMAPKKK21	Bradi4g36880	42043874-42051570	AtRaf21	At2g17700	7685388-7689436
BdMAPKKK21	Bradi4g36880	42043874-42051570	AtRaf29	At4g35780	16946521-16950594
BdMAPKKK22	Bradi2g39350	39411876-39418219	OsMAPKKK56	LOC_Os05g01780	471400-476283
BdMAPKKK23	Bradi4g29500	34951319-34957350	BdMAPKKK25	Bradi3g36080	38320000-38325924
BdMAPKKK23	Bradi4g29500	34951319-34957350	OsMAPKKK13	LOC_Os09g21510	13014100-13015909
BdMAPKKK24	Bradi1g60340	59671548-59677424	OsMAPKKK54	LOC_Os03g28300	16282710-16290482
BdMAPKKK25	Bradi3g36080	38320000-38325924	BdMAPKKK23	Bradi4g29500	34951319-34957350
BdMAPKKK25	Bradi3g36080	38320000-38325924	OsMAPKKK13	LOC_Os09g21510	13014100-13015909
BdMAPKKK28	Bradi3g51460	52493768-52498074	OsMAPKKK29	LOC_Os02g45130	27376763-27372028
BdMAPKKK31	Bradi2g06260	4698348-4702360	OsMAPKKK61	LOC_Os01g10450	5514480-5510516
BdMAPKKK32	Bradi2g19590	17241445-17245595	BdMAPKKK35	Bradi2g49700	49740523-49744032
BdMAPKKK32	Bradi2g19590	17241445-17245595	OsMAPKKK49	LOC_Os05g44290	25771768-25776102
BdMAPKKK33	Bradi3g48360	49832148-49837407	AtRaf46	At3g59830	22102803-22105380
BdMAPKKK33	Bradi3g48360	49832148-49837407	OsMAPKKK75	LOC_Os02g39560	23876495-23881702
BdMAPKKK35	Bradi2g49700	49740523-49744032	BdMAPKKK32	Bradi2g19590	17241445-17245595
BdMAPKKK35	Bradi2g49700	49740523-49744032	OsMAPKKK49	LOC_Os05g44290	25771794-25775926
BdMAPKKK36	Bradi2g57470	55622119-55624811	AtRaf46	At3g59830	22102803-22105380
BdMAPKKK36	Bradi2g57470	55622119-55624811	OsMAPKKK74	LOC_Os01g66860	38818767-38822164
BdMAPKKK40	Bradi2g49790	49806289-49809892	AtRaf27	At4g18950	10375364-10378390
BdMAPKKK40	Bradi2g49790	49806289-49809892	OsMAPKKK72	LOC_Os01g54480	31337161-31332864
BdMAPKKK41	Bradi1g14000	10874042-10878698	Bradi4g02370	Bradi4g02370	1555141-1554617
BdMAPKKK41	Bradi1g14000	10874042-10878698	OsMAPKKK27	LOC_Os03g43760	24476011-24480930
BdMAPKKK41	Bradi1g14000	10874042-10878698	OsMAPKKK59	LOC_Os12g41260	25583204-25586395
BdMAPKKK43	Bradi1g04080	2776045-2779519	OsMAPKKK42	LOC_Os03g60150	34206308-34202415
BdMAPKKK45	Bradi3g47600	49203215-49208033	OsMAPKKK25	LOC_Os02g38080	22999976-23005321
BdMAPKKK46	Bradi2g44910	45294740-45299227	BdMAPKKK47	Bradi2g15560	13839453-13843007
BdMAPKKK46	Bradi2g44910	45294740-45299227	OsMAPKKK31	LOC_Os01g45380	25757795-25762187
BdMAPKKK46	Bradi2g44910	45294740-45299227	OsMAPKKK34	LOC_Os05g50190	28763886-28760152
BdMAPKKK47	Bradi2g15560	13839453-13843007	BdMAPKKK46	Bradi2g44910	45294740-45299227
BdMAPKKK47	Bradi2g15560	13839453-13843007	OsMAPKKK34	LOC_Os05g50190	28763886-28760152
BdMAPKKK49	Bradi1g20390	16330864-16335462	OsMAPKKK65	LOC_Os07g43900	26250364-26245519
BdMAPKKK50	Bradi4g44430	47836156-47839912	AtZIK2	At5g58350	23584789-23587801
BdMAPKKK50	Bradi4g44430	47836156-47839912	OsMAPKKK50	LOC_Os12g02250	705258-698936
BdMAPKKK54	Bradi1g23320	18706049-18708119	OsMAPKKK64	LOC_Os07g39520	23677863-23679948
BdMAPKKK56	Bradi4g41940	45996792-45999204	OsMAPKKK46	LOC_Os11g06140	2929173-2925595
BdMAPKKK56	Bradi4g41940	45996792-45999204	OsMAPKKK52	LOC_Os12g06490	3131907-3128498
BdMAPKKK60	Bradi1g65500	64476929-64478355	AtMAPKKK13	At1g07150	2193941-2195798
BdMAPKKK60	Bradi1g65500	64476929-64478355	AtMAPKKK14	At2g30040	12821710-12823169
BdMAPKKK60	Bradi1g65500	64476929-64478355	OsMAPKKK73	LOC_Os03g18170	10184582-10187474
BdMAPKKK62	Bradi4g09990	9550465-9557023	LOC_Os11g45280	LOC_Os11g45280	27399798-27406608
BdMAPKKK64	Bradi4g02900	2137135-2141974	OsMAPKKK23	LOC_Os12g40279	24925191-24950240
BdMAPKKK70	Bradi3g57740	57407383-57412051	LOC_Os02g53040	LOC_Os02g53040	32461128-32462832
AtMAPKKK1	At1g09000	2891037-2895182	AtMAPKKK2	At1g54960	20499950-20503841
AtMAPKKK2	At1g54960	20499950-20503841	AtMAPKKK1	At1g09000	2891037-2895182
AtMAPKKK2	At1g54960	20499950-20503841	AtMAPKKK7	At3g13530	4411688-4419320

AtMAPKKK7	At3g13530	4411688-4419320	AtMAPKKK2	At1g54960	20499950-20503841
AtMAPKKK13	At1g07150	2193941-2195798	AtMAPKKK14	At2g30040	12821710-12823169
AtMAPKKK13	At1g07150	2193941-2195798	BdMAPKKK60	Bradi1g65500	64476929-64478355
AtMAPKKK14	At2g30040	12821710-12823169	BdMAPKKK60	Bradi1g65500	64476929-64478355
AtMAPKKK14	At2g30040	12821710-12823169	AtMAPKKK13	At1g07150	2193941-2195798
AtMAPKKK15	At5g55090	22356852-22358198	AtMAPKKK16	At4g26890	13511839-13513406
AtMAPKKK16	At4g26890	13511839-13513406	AtMAPKKK15	At5g55090	22356852-22358198
AtMAPKKK17	At2g32510	13798821-13799939	AtMAPKKK18	At1g05100	1469610-1470881
AtMAPKKK18	At1g05100	1469610-1470881	AtMAPKKK17	At2g32510	13798821-13799939
AtMAPKKK19	At5g67080	26772726-26773760	AtMAPKKK20	At3g50310	18648151-18649521
AtMAPKKK19	At5g67080	26772726-26773760	AtMAPKKK21	At4g36950	17422572-17423901
AtMAPKKK20	At3g50310	18648151-18649521	AtMAPKKK19	At5g67080	26772726-26773760
AtMAPKKK21	At4g36950	17422572-17423901	AtMAPKKK19	At5g67080	26772726-26773760
AtRaf4	At1g18160	6248793-6254039	AtRaf5	At1g73660	27691893-27697211
AtRaf5	At1g73660	27691893-27697211	AtRaf4	At1g18160	6248793-6254039
AtRaf7	At3g06620	2062206-2067416	AtRaf10	At5g49470	20063448-20068665
AtRaf10	At5g49470	20063448-20068665	AtRaf7	At3g06620	2062206-2067416
AtRaf15	At3g58640	21686741-21693841	At2g42640	At2g42640	17758532-17764111
AtRaf18	At1g16270	5563087-5568368	AtRaf20	At1g79570	39932578-29938856
AtRaf20	At1g79570	39932578-29938856	AtRaf18	At1g16270	5563087-5568368
AtRaf21	At2g17700	7685388-7689436	BdMAPKKK21	Bradi4g36880	42043874-42051570
AtRaf21	At2g17700	7685388-7689436	AtRaf29	At4g35780	16946520-16950594
AtRaf22	At2g24360	10364451-10366902	AtRaf28	At4g31170	15153182-15155703
AtRaf23	At2g31800	13520325-13523708	AtRaf25	At2g43850	18159182-18162044
AtRaf25	At2g43850	18159182-18162044	AtRaf23	At2g31800	13520325-13523708
AtRaf25	At2g43850	18159182-18162044	AtRaf46	At3g59830	22102803-22105380
AtRaf26	At4g14780	8492832-8494591	AtRaf39	At3g22750	8037216-8039903
AtRaf27	At4g18950	10375364-10378390	BdMAPKKK40	Bradi2g49790	49806289-49809892
AtRaf27	At4g18950	10375364-10378390	OsMAPKKK72	LOC_Os01g54480	31337161-31332864
AtRaf28	At4g31170	15153182-15155703	AtRaf22	At2g24360	10364451-10366902
AtRaf29	At4g35780	16946521-16950594	BdMAPKKK21	Bradi4g36880	42043874-42051570
AtRaf29	At4g35780	16946520-16950594	AtRaf21	At2g17700	7685388-7689436
AtRaf32	At5g40540	16237298-16239601	AtRaf41	At3g27560	10210431-10213004
AtRaf36	At5g58950	23800928-23803676	AtRaf43	At3g46930	17285643-17288032
AtRaf37	At5g66710	26636609-26638794	AtRaf44	At3g50720	18847519-18849430
AtRaf39	At3g22750	8037216-8039903	AtRaf26	At4g14780	8492832-8494591
AtRaf39	At3g22750	8037216-8039903	AtRaf48	At3g63260	23372881-23375007
AtRaf41	At3g27560	10210431-10213004	AtRaf32	At5g40540	16237298-16239601
AtRaf43	At3g46930	17285643-17288032	AtRaf36	At5g58950	23800928-23803676
AtRaf44	At3g50720	18847519-18849430	AtRaf37	At5g66710	26636609-26638794
AtRaf46	At3g59830	22102803-22105380	BdMAPKKK33	Bradi3g48360	49832148-49837407
AtRaf46	At3g59830	22102803-22105380	BdMAPKKK36	Bradi2g57470	55622119-55624811
AtRaf46	At3g59830	22102803-22105380	AtRaf25	At2g43850	18159182-18162044
AtRaf48	At3g63260	23372881-23375007	AtRaf39	At3g22750	8037216-8039903
AtZIK2	At5g58350	23584789-23587801	BdMAPKKK50	Bradi4g44430	47836156-47839912
AtZIK4	At3g04910	1354635-1358219	AtZIK9	At5g28080	10090070-10092402

AtZIK5	At3g18750	6454110-6457512	AtZIK7	At1g49160	18179304-18182391
AtZIK7	At1g49160	18179304-18182391	AtZIK5	At3g18750	6454110-6457512
AtZIK9	At5g28080	10090070-10092402	AtZIK4	At3g04910	1354635-1358219
OsMAPKKK1	LOC_Os03g06410	3201345-3208276	OsMAPKKK2	LOC_Os10g29540	15341888-15350754
OsMAPKKK2	LOC_Os10g29540	15341888-15350754	OsMAPKKK1	LOC_Os03g06410	3201345-3208276
OsMAPKKK5	LOC_Os12g37570	23061642-23065235	LOC_Os11g45280	LOC_Os11g45280	27399450-27406535
OsMAPKKK5	LOC_Os12g37570	23061181-23068420	BdMAPKKK15	Bradi4g04470	3687248-3696919
OsMAPKKK6	LOC_Os02g50970	31171975-31180079	OsMAPKKK7	LOC_Os06g12590	6823036-6832862
OsMAPKKK7	LOC_Os06g12590	6832750-6822979	BdMAPKKK5	Bradi3g59510	58646176-58654454
OsMAPKKK7	LOC_Os06g12590	6823036-6832862	BdMAPKKK7	Bradi1g45040	43232910-43245317
OsMAPKKK7	LOC_Os06g12590	6823036-6832862	OsMAPKKK6	LOC_Os02g50970	31171975-31180079
OsMAPKKK9	LOC_Os02g44642	27048920-27061955	OsMAPKKK10	LOC_Os04g47240	28049246-28056867
OsMAPKKK10	LOC_Os04g47240	28049246-28056867	OsMAPKKK9	LOC_Os02g44642	27048920-27061955
OsMAPKKK11	LOC_Os07g02780	1015354-1021607	OsMAPKKK18	LOC_Os03g55560	31612448-31619532
OsMAPKKK13	LOC_Os09g21510	13010191-13017007	BdMAPKKK23	Bradi4g29500	34951319-34957350
OsMAPKKK13	LOC_Os09g21510	13014100-13015909	BdMAPKKK25	Bradi3g36080	38320000-38325924
OsMAPKKK13	LOC_Os09g21510	13014100-13015909	OsMAPKKK15	LOC_Os08g32600	20186987-20192334
OsMAPKKK15	LOC_Os08g32600	20186987-20192334	OsMAPKKK13	LOC_Os09g21510	13014100-13015909
OsMAPKKK16	LOC_Os04g35700	21752568-21758576	OsMAPKKK19	LOC_Os02g35010	21002439-21008209
OsMAPKKK18	LOC_Os03g55560	31612448-31619532	OsMAPKKK11	LOC_Os07g02780	1015354-1021607
OsMAPKKK19	LOC_Os02g35010	21002439-21008209	OsMAPKKK16	LOC_Os04g35700	21752568-21758576
OsMAPKKK20	LOC_Os07g38530	23145979-23149609	BdMAPKKK16	Bradi1g23970	19257221-19260591
OsMAPKKK21	LOC_Os07g25680	14723111-14727159	BdMAPKKK2	Bradi1g28950	24311845-24322003
OsMAPKKK22	LOC_Os03g49640	28269607-28275709	OsMAPKKK28	LOC_Os03g15570	8573083-8577492
OsMAPKKK23	LOC_Os12g40279	24926341-24930364	Bradi4g02910	Bradi4g02910	2144483-2162831
OsMAPKKK23	LOC_Os12g40279	24925191-24950240	BdMAPKKK64	Bradi4g02900	2137135-2141974
OsMAPKKK24	LOC_Os04g56530	33707455-33719236	BdMAPKKK1	Bradi5g24870	26381665-26393219
OsMAPKKK25	LOC_Os02g38080	22999976-23005321	BdMAPKKK45	Bradi3g47600	49203215-49208033
OsMAPKKK27	LOC_Os03g43760	24476011-24480930	BdMAPKKK41	Bradi1g14000	10874042-10878698
OsMAPKKK27	LOC_Os03g43760	24476060-24480927	OsMAPKKK59	LOC_Os12g41260	25583204-25586395
OsMAPKKK28	LOC_Os03g15570	8573083-8577492	OsMAPKKK22	LOC_Os03g49640	28269607-28275709
OsMAPKKK29	LOC_Os02g45130	27376763-27372028	BdMAPKKK28	Bradi3g51460	52493768-52498074
OsMAPKKK31	LOC_Os01g45380	25757795-25762187	BdMAPKKK46	Bradi2g44910	45294740-45299227
OsMAPKKK31	LOC_Os01g45380	25757795-25762187	OsMAPKKK34	LOC_Os05g50190	28763886-28760152
OsMAPKKK32	LOC_Os08g12750	7553773-7558719	OsMAPKKK37	LOC_Os04g51950	30825563-30829578
OsMAPKKK33	LOC_Os02g07790	4078521-4081527	OsMAPKKK38	LOC_Os06g45300	27384305-27386857
OsMAPKKK34	LOC_Os05g50190	28763886-28760152	BdMAPKKK46	Bradi2g44910	45294740-45299227
OsMAPKKK34	LOC_Os05g50190	28763886-28760152	BdMAPKKK47	Bradi2g15560	13839453-13843007
OsMAPKKK34	LOC_Os05g50190	28763886-28760152	OsMAPKKK31	LOC_Os01g45380	25757795-25762187
OsMAPKKK35	LOC_Os02g54510	33387811-33392575	BdMAPKKK3	Bradi3g60210	59221921-59227497
OsMAPKKK37	LOC_Os04g51950	30825563-30829578	OsMAPKKK32	LOC_Os08g12750	7553773-7558719
OsMAPKKK37	LOC_Os04g51950	30825563-30829578	OsMAPKKK38	LOC_Os06g45300	27384305-27386857
OsMAPKKK38	LOC_Os06g45300	27384305-27386857	OsMAPKKK33	LOC_Os02g07790	4078521-4081527
OsMAPKKK38	LOC_Os06g45300	27384305-27386857	OsMAPKKK37	LOC_Os04g51950	30825563-30829578
OsMAPKKK39	LOC_Os06g08280	4008139-4013539	BdMAPKKK4	Bradi1g47570	46211026-46216334
OsMAPKKK40	LOC_Os01g48330	27702476-27692557	BdMAPKKK10	Bradi2g46340	46786767-46795821

OsMAPKKK42	LOC_Os03g60150	34206308-34202415	BdMAPKKK43	Bradi1g04080	2776045-2779519
OsMAPKKK46	LOC_Os11g06140	2929173-2925595	BdMAPKKK56	Bradi4g41940	45996792-45999204
OsMAPKKK49	LOC_Os05g44290	25771768-25776102	BdMAPKKK32	Bradi2g19590	17241445-17245595
OsMAPKKK49	LOC_Os05g44290	25771794-25775926	BdMAPKKK35	Bradi2g49700	49740523-49744032
OsMAPKKK49	LOC_Os05g44290	25771794-25775926	OsMAPKKK51	LOC_Os01g54350	31275242-31279385
OsMAPKKK50	LOC_Os12g02250	698969-705122	BdMAPKKK50	Bradi4g44430	47836156-47839912
OsMAPKKK50	LOC_Os12g02250	698969-705122	OsMAPKKK53	LOC_Os11g02305	655098-661236
OsMAPKKK51	LOC_Os01g54350	31275242-31279385	OsMAPKKK49	LOC_Os05g44290	25771794-25775926
OsMAPKKK52	LOC_Os12g06490	3131907-3128498	BdMAPKKK56	Bradi4g41940	45996792-45999204
OsMAPKKK53	LOC_Os11g02305	655098-661236	OsMAPKKK50	LOC_Os12g02250	698969-705122
OsMAPKKK54	LOC_Os03g28300	16282710-16290482	BdMAPKKK24	Bradi1g60340	59671548-59677424
OsMAPKKK56	LOC_Os05g01780	471400-476283	BdMAPKKK22	Bradi2g39350	39411876-39418219
OsMAPKKK57	LOC_Os05g46750	27068628-27070015	OsMAPKKK63	LOC_Os01g50370	28921498-28922922
OsMAPKKK59	LOC_Os12g41260	25583204-25586395	BdMAPKKK41	Bradi1g14000	10874042-10878698
OsMAPKKK59	LOC_Os12g41260	25583204-25586395	OsMAPKKK27	LOC_Os03g43760	24476060-24480927
OsMAPKKK61	LOC_Os01g10450	5514480-5510516	BdMAPKKK31	Bradi2g06260	4698348-4702360
OsMAPKKK63	LOC_Os01g50370	28921498-28922922	OsMAPKKK57	LOC_Os05g46750	27068628-27070015
OsMAPKKK64	LOC_Os07g39520	23677863-23679948	BdMAPKKK54	Bradi1g23320	18706049-18708119
OsMAPKKK65	LOC_Os07g43900	26250364-26245519	BdMAPKKK49	Bradi1g20390	16330864-16335462
OsMAPKKK72	LOC_Os01g54480	31337161-31332864	BdMAPKKK40	Bradi2g49790	49806289-49809892
OsMAPKKK72	LOC_Os01g54480	31337161-31332864	AtRaf27	At4g18950	10375364-10378390
OsMAPKKK73	LOC_Os03g18170	10184582-10187474	BdMAPKKK60	Bradi1g65500	64476929-64478355
OsMAPKKK74	LOC_Os01g66860	38818767-38822164	BdMAPKKK36	Bradi2g57470	55622119-55624811
OsMAPKKK75	LOC_Os02g39560	23876495-23881702	BdMAPKKK33	Bradi3g48360	49832148-49837407
