# Supplementary material for: Genome-wide exploration of the molecular evolution and regulatory network of mitogen-activated protein kinase cascades upon multiple stresses in Brachypodium distachyon
Source: BMC Genomics. 2015 Mar 24;16(1):228. doi: 10.1186/s12864-015-1452-1 (PMC4404688; doi:10.1186/s12864-015-1452-1)
Supplement: Additional file 8: — Expression data of the BdMAPK cascade kinase genes after stresses and phytohormone treatment. [file 12864_2015_1452_MOESM8_ESM.pdf]

Additional file 8A-1 Expression data of the *BdMAPK* family genes after abiotic stresses treatment.

	4°C 3h			45°C 3h			PEG 3h			H_2O_2 3h			Nacl 3h		
	fold-change	p-value	up/down	fold-change	p-value	up/down	fold-change	p-value	up/down	fold-change	p-value	up/down	fold-change	p-value	up/down
**BdMPK3**	11.6535	0.0000	up	1.3961	0.0071		1.0638	0.1858		1.3491	0.0009		0.5962	0.0004	
**BdMPK4**	0.7864	0.0412		0.3600	0.0003	down	0.2749	0.0001	down	0.1630	0.0000	down	0.4539	0.0257	
**BdMPK6**	3.0795	0.0000	up	1.1780	0.0051		0.7943	0.0026		0.6764	0.0015		0.2952	0.0005	down
**BdMPK7-1**	1.1811	0.2310		0.3338	0.0106		0.4514	0.0027	down	0.7071	0.0508		0.4718	0.0001	down
**BdMPK7-2**	1.5658	0.5324		0.5369	0.3225		0.5822	0.3719		1.0907	0.8830		0.9282	0.1248	
**BdMPK11**	1.9048	0.0111		0.5376	0.0192		1.2930	0.1073		0.2338	0.0010	down	0.3071	0.0000	down
**BdMPK14**	2.0775	0.0053	up	0.7855	0.1234		1.0552	0.6587		0.5380	0.0195		0.3659	0.0000	down
**BdMPK16**	5.4391	0.0000	up	1.4529	0.0002		1.0625	0.1126		0.3442	0.0000	down	0.4543	0.0151	
**BdMPK17**	9.0092	0.0000	up	1.6331	0.0000		1.6548	0.0001		2.0378	0.0000	up	1.3349	0.0011	
**BdMPK20-1**	2.9210	0.0000	up	1.7503	0.0000		0.9065	0.0098		0.6238	0.0000		0.4215	0.0005	down
**BdMPK20-2**	2.0138	0.0000	up	0.6587	0.0001		0.9915	0.7606		0.3712	0.0000	down	0.3848	0.0000	down
**BdMPK20-3**	2.8412	0.0002	up	1.2559	0.0456		0.4536	0.0045	down	0.9485	0.5115		0.3397	0.0047	down
**BdMPK20-4**	4.1072	0.0000	up	1.3793	0.0014		0.4362	0.0001	down	0.1558	0.0000	down	0.4558	0.0020	down
**BdMPK20-5**	0.9183	0.0972		0.3384	0.0000	down	0.7373	0.0000		0.1937	0.0000	down	0.3856	0.0013	down
**BdMPK21-1**	2.6706	0.0005	up	0.8050	0.0730		0.9666	0.7511		0.4693	0.0020	down	0.3124	0.0000	down

Additional file 8A-2 Expression data of the *BdMAPK* family genes after abiotic stresses treatment.

	**4^oC 6h**			**45^oC 6h**			**PEG 6h**			**H_2O_2 6h**			**Nacl 6h**		
	fold-change	p-value	up/down	fold-change	p-value	up/down	fold-change	p-value	up/down	fold-change	p-value	up/down	fold-change	p-value	up/down
**BdMPK3**	4.9111	0.0000	up	0.9920	0.7231		0.8759	0.0031		1.8955	0.0008		0.6579	0.0001	
**BdMPK4**	2.2720	0.0001	up	1.9707	0.0001		0.7858	0.0208		1.0926	0.0821		1.4817	0.0007	
**BdMPK6**	1.7393	0.0158		1.4292	0.0006		0.8376	0.0173		1.1414	0.0416		0.4800	0.0013	down
**BdMPK7-1**	0.3771	0.0065	down	0.9974	0.9669		0.0850	0.0000	down	0.2447	0.0002	down	0.5061	0.0005	
**BdMPK7-2**	1.4916	0.3921		0.5929	0.2198		0.5306	0.2246		1.7301	0.2754		0.6423	0.0001	
**BdMPK11**	1.4738	0.3089		0.8205	0.4550		0.2633	0.0031	down	0.9717	0.8694		0.7193	0.0018	
**BdMPK14**	1.0932	0.7104		2.3155	0.0012	up	0.5744	0.0106		4.1052	0.0000	up	0.2822	0.0000	down
**BdMPK16**	6.3005	0.0000	up	1.6436	0.0004		0.3863	0.0000	down	1.1578	0.0429		0.9136	0.1458	
**BdMPK17**	7.9386	0.0000	up	2.3749	0.0002	up	1.6433	0.0002		1.6403	0.0002		0.9355	0.0832	
**BdMPK20-1**	1.4241	0.0035		1.7731	0.0002		0.3211	0.0000	down	0.3868	0.0000	down	1.4830	0.0260	
**BdMPK20-2**	0.7645	0.0001		0.3668	0.0015	down	0.0551	0.0000	down	0.4130	0.0000	down	1.4831	0.0347	
**BdMPK20-3**	4.9684	0.0000	up	2.2099	0.0161		0.4628	0.0000	down	5.1672	0.0000	up	0.4179	0.0000	down
**BdMPK20-4**	3.8987	0.0000	up	1.4295	0.0039		0.0275	0.0000	down	0.4642	0.0000	down	1.1548	0.4139	
**BdMPK20-5**	0.4916	0.0000	down	0.5826	0.0000		0.0503	0.0000	down	0.3395	0.0000	down	1.6808	0.0753	
**BdMPK21-1**	6.2327	0.0003	up	1.2577	0.4767		1.2503	0.2612		9.7254	0.0001	up	0.2792	0.0000	down

Additional file 8B-1 Expression data of the *BdMAPKK* family genes after abiotic stresses treatment.

	4°C 3h			45°C 3h			PEG 3h			H_2O_2 3h			Nacl 3h		
	fold-change	p-value	up/down	fold-change	p-value	up/down	fold-change	p-value	up/down	fold-change	p-value	up/down	fold-change	p-value	up/down
**BdMKK1**	1.4958	0.0007		0.5386	0.0001		0.6813	0.0017		0.4863	0.0001	down	0.1803	0.0002	down
**BdMKK3-1**	2.9757	0.0000	up	0.8452	0.1102		0.9204	0.3009		1.7097	0.0005		0.5216	0.0000	
**BdMKK3-2**	5.1906	0.0000	up	2.2720	0.0002	up	1.9955	0.0004		1.7559	0.0008		0.2513	0.0000	down
**BdMKK3-3**	2.8605	0.0009	up	1.9093	0.0050		4.2744	0.0002	up	3.3791	0.0004	up	0.9214	0.0458	
**BdMKK4**	2.9055	0.0280		2.3512	0.0156		1.3667	0.4643		0.8442	0.7323		0.4662	0.0010	down
**BdMKK5**	1.4282	0.4215		1.3858	0.5725		0.7550	0.0249		1.3001	0.3021		0.5750	0.0025	
**BdMKK6**	2.2631	0.0000	up	1.3857	0.0002		0.7017	0.0000		0.2385	0.0000	down	0.5382	0.0000	
**BdMKK10-1**	0.9943	0.9943		0.5090	0.3387		0.5576	0.4187		0.4972	0.6127		0.4603	0.0935	
**BdMKK10-2**	3.8832	0.0000	up	1.3459	0.0002		0.6906	0.0000		3.2667	0.0000	up	0.9575	0.6624	
**BdMKK10-3**	8.0010	0.0095	up	0.0304	0.0280		4.4706	0.0005	up	0.8654	0.3517		0.2882	0.0184	
**BdMKK10-5**	1.5438	0.0000		6.7047	0.0000	up	1.4813	0.0004		1.3804	0.0024		2.0765	0.0000	up

Additional file 8B-2 Expression data of the *BdMAPKK* family genes after abiotic stresses treatment.

	**4°C 6h**			**45°C 6h**			**PEG 6h**			**H_2O_2 6h**			**Nacl 6h**		
	fold-change	p-value	up/down	fold-change	p-value	up/down	fold-change	p-value	up/down	fold-change	p-value	up/down	fold-change	p-value	up/down
**BdMKK1**	0.8014	0.0117		0.4950	0.0028	down	0.1947	0.0000	down	0.6661	0.0002		0.6620	0.0004	
**BdMKK3-1**	1.8033	0.0014		0.5716	0.0001		1.0327	0.5637		4.0481	0.0000	up	0.2134	0.0000	down
**BdMKK3-2**	3.6097	0.0001	up	0.8039	0.0574		0.7926	0.0754		4.4882	0.0001	up	0.1941	0.0000	down
**BdMKK3-3**	1.9408	0.0034		0.3884	0.0001	down	3.2115	0.0001	up	8.8085	0.0000	up	0.4561	0.0000	down
**BdMKK4**	0.0775	0.0153		0.5053	0.4682		0.0706	0.0131		2.5075	0.2819		0.3921	0.0355	
**BdMKK5**	1.0564	0.3027		8.9427	0.0000	up	1.1144	0.0979		8.9362	0.0000	up	0.2402	0.0008	down
**BdMKK6**	1.7737	0.0004		1.3989	0.0083		0.1996	0.0000	down	2.0132	0.0002	up	0.4417	0.0000	down
**BdMKK10-1**	5.1275	0.0589		3.7559	0.6016		0.3579	0.6807		0.8643	0.8994		0.0004	0.1544	
**BdMKK10-2**	1.1456	0.0343		0.9272	0.1487		1.0528	0.2797		7.3364	0.0000	up	0.2757	0.0000	down
**BdMKK10-3**	0.0599	0.2679		0.1086	0.0797		0.0010	0.0019	down	6.5630	0.1186		0.6895	0.2316	
**BdMKK10-5**	0.7758	0.0011		15.8837	0.0000	up	0.3668	0.0002	down	0.8199	0.0311		0.2102	0.0000	down

Additional file 8C-1 Expression data of the *BdMAPKKK* family genes after abiotic stresses treatment.

	4°C 3h			45°C 3h			PEG 3h			H_2O_2 3h			Nacl 3h		
	fold-change	p-value	up/down	fold-change	p-value	up/down	fold-change	p-value	up/down	fold-change	p-value	up/down	fold-change	p-value	up/down
**BdMAPKKK1**	1.4108	0.2861		0.4581	0.0072	down	0.2688	0.0257		0.5878	0.2073		1.1080	0.3329	
**BdMAPKKK2**	1.7019	0.0014		0.4611	0.0000	down	0.4646	0.0006	down	0.0587	0.0000	down	0.4624	0.0012	down
**BdMAPKKK3**	3.3328	0.0028	up	1.1163	0.6412		0.8579	0.7991		0.1138	0.0038	down	0.5833	0.0003	
**BdMAPKKK4**	2.8266	0.0000	up	2.4454	0.0000	up	4.6403	0.0000	up	0.3948	0.0066	down	0.7296	0.0042	
**BdMAPKKK5**	2.3194	0.0186		1.6124	0.4227		0.4627	0.1355		0.0720	0.0035	down	0.7233	0.0000	
**BdMAPKKK6**	5.7655	0.0000	up	7.3109	0.0383		1.0967	0.4818		0.1026	0.0014	down	0.8200	0.0026	
**BdMAPKKK7**	1.2198	0.2238		0.5454	0.0130		0.2937	0.0099	down	0.0313	0.0002	down	0.4243	0.0001	down
**BdMAPKKK8**	0.8470	0.6998		0.3856	0.0458		0.3598	0.0828		0.1890	0.0150		0.6577	0.0019	
**BdMAPKKK9**	1.1796	0.0179		0.3865	0.0002	down	0.3802	0.0004	down	0.0721	0.0001	down	0.3266	0.0006	down
**BdMAPKKK10**	1.5466	0.1173		0.4381	0.0177		0.5863	0.0861		0.1794	0.0013	down	0.8598	0.1452	
**BdMAPKKK11**	1.1466	0.3421		0.4634	0.0027	down	0.3957	0.0014	down	0.1738	0.0002	down	0.6904	0.0483	
**BdMAPKKK12**	3.3001	0.0005	up	1.7640	0.0412		1.1841	0.3053		0.3198	0.0413		0.6438	0.0001	
**BdMAPKKK13**	2.0226	0.1025		0.6982	0.3528		0.7177	0.3874		0.3062	0.0415		0.8482	0.1832	
**BdMAPKKK14**	4.1162	0.0058	up	0.2535	0.0222		0.5082	0.0680		1.1060	0.7277		0.2941	0.0027	down
**BdMAPKKK15**	2.6043	0.0046	up	0.6391	0.0319		0.6067	0.0201		0.2155	0.0096	down	0.7540	0.0026	
**BdMAPKKK16**	2.4008	0.0063	up	0.7971	0.1662		0.9058	0.4975		0.7474	0.1894		0.8282	0.0979	
**BdMAPKKK17**	2.3187	0.0448		0.8817	0.7102		0.7812	0.4436		0.1730	0.0207		0.6081	0.0012	
**BdMAPKKK18**	4.9919	0.0013	up	2.6022	0.0093	up	1.8179	0.0397		0.3580	0.0121		0.4235	0.0000	down
**BdMAPKKK19**	3.0828	0.0003	up	1.4435	0.0228		1.2202	0.0507		0.6284	0.0576		1.1815	0.0098	
**BdMAPKKK20**	1.6994	0.0483		0.4341	0.0270		0.8335	0.4284		0.5560	0.0984		0.5941	0.0001	
**BdMAPKKK21**	15.4844	0.0002	up	5.8730	0.0023	up	1.5745	0.1004		0.8412	0.6082		3.5312	0.0000	up
**BdMAPKKK22**	1.8859	0.1692		0.9909	0.9843		0.8104	0.6624		0.7860	0.5865		1.0145	0.5681	
**BdMAPKKK23**	1.2817	0.1400		1.1540	0.3628		1.4146	0.0147		0.3341	0.0005	down	0.4769	0.0005	down
**BdMAPKKK24**	2.7411	0.0051	up	0.6225	0.0612		0.3326	0.0037	down	0.0676	0.0022	down	0.4598	0.0106	

**BdMAPKKK25**	1.0762	0.4336		0.2311	0.0000	down	0.7290	0.0079		0.5929	0.0335		0.8838	0.0032	
**BdMAPKKK26**	1.8600	0.0249		0.9457	0.4575		1.0775	0.6852		0.4955	0.0195		0.6402	0.0096	
**BdMAPKKK27**	7.8248	0.0005	up	6.5229	0.0004	up	2.6788	0.0040	up	0.9487	0.7592		0.7686	0.0915	
**BdMAPKKK28**	0.6812	0.0116		0.3290	0.0000	down	0.5067	0.0000		0.5365	0.0022		0.5994	0.0007	
**BdMAPKKK29**	2.8850	0.0511		0.6698	0.3687		0.5788	0.2214		0.3786	0.0892		0.7317	0.0560	
**BdMAPKKK30**	18.0785	0.0000	up	9.0442	0.0000	up	3.6875	0.0000	up	1.8431	0.0025		0.7671	0.4743	
**BdMAPKKK31**	2.3833	0.0614		0.2673	0.0152		0.4505	0.0697		0.6270	0.2501		0.9979	0.9763	
**BdMAPKKK32**	1.7289	0.2058		0.4658	0.0837		0.8172	0.5697		0.6954	0.3471		1.0166	0.8903	
**BdMAPKKK33**	2.0077	0.0256		0.6554	0.1170		0.4361	0.0146		0.1614	0.0046	down	0.4486	0.0000	down
**BdMAPKKK34**	3.1428	0.1155		0.6002	0.3486		0.6027	0.3842		1.1613	0.7765		0.9981	0.9727	
**BdMAPKKK35**	3.1714	0.0018	up	2.4843	0.0180		0.7330	0.1175		0.8290	0.6328		1.1405	0.0249	
**BdMAPKKK36**	7.2796	0.0006	up	0.4424	0.0162		0.5817	0.0540		1.5629	0.1691		0.4796	0.0001	down
**BdMAPKKK37**	3.3315	0.0222		1.3679	0.4261		0.7492	0.4441		0.6471	0.3237		0.8489	0.0081	
**BdMAPKKK38**	2.3209	0.0094	up	1.4721	0.1758		0.8602	0.4468		0.4564	0.1099		0.7515	0.0140	
**BdMAPKKK39**	3.9018	0.0098	up	1.2874	0.5007		1.0017	0.9960		0.4025	0.0630		1.4141	0.0212	
**BdMAPKKK40**	2.0211	0.1495		0.6480	0.3178		0.6676	0.3519		0.9063	0.8101		1.0637	0.2378	
**BdMAPKKK41**	1.2491	0.1208		0.6930	0.0144		0.6023	0.0016		1.4637	0.0053		1.2879	0.0013	
**BdMAPKKK42**	3.1126	0.0061	up	0.0512	0.0001	down	0.0045	0.0000	down	0.3051	0.0037	down	0.0811	0.0002	down
**BdMAPKKK43**	3.2795	0.0033	up	0.7665	0.2927		1.4088	0.1465		0.3720	0.0788		0.6836	0.0000	
**BdMAPKKK44**	4.9926	0.0063	up	1.1869	0.5628		0.6127	0.1609		0.3043	0.0120		1.5240	0.0329	
**BdMAPKKK45**	2.9944	0.0079	up	0.4617	0.0200		0.7852	0.3050		1.2858	0.2988		0.7980	0.2126	
**BdMAPKKK46**	2.5077	0.0373		0.8112	0.5233		0.8048	0.4974		0.4057	0.0773		0.7199	0.0368	
**BdMAPKKK47**	2.3455	0.0272		1.5079	0.2199		0.7304	0.2786		0.8723	0.6188		3.3490	0.0000	up
**BdMAPKKK48**	1.0654	0.5941		0.2899	0.0002	down	0.6761	0.0096		0.4663	0.0211		0.5706	0.0005	
**BdMAPKKK49**	3.7778	0.0135		3.0405	0.0218		0.7240	0.3489		0.9123	0.7783		1.6444	0.0020	
**BdMAPKKK50**	1.0418	0.9464		1.8413	0.1144		0.8267	0.6270		0.5771	0.0013		0.6240	0.1327	
**BdMAPKKK51**	9.5601	0.0008	up	4.5122	0.0030	up	1.6988	0.1160		33.0582	0.0001	up	103.9945	0.0000	up
**BdMAPKKK52**	49.0899	0.0000	up	1.5718	0.0100		0.7824	0.1810		2.3534	0.0000	up	4.3787	0.0039	up
**BdMAPKKK53**	3.3627	0.0006	up	78.7300	0.0000	up	10.9649	0.0000	up	12.9934	0.0000	up	14.0156	0.0001	up

**BdMAPKKK54**	2.3747	0.1338		1.7664	0.0094		0.2669	0.0001	down	0.0046	0.0000	down	0.3977	0.0076	down
**BdMAPKKK55**	3.0225	0.0108		1.4900	0.1829		2.3910	0.0211		0.5697	0.0826		1.6666	0.2368	
**BdMAPKKK56**	1.2309	0.6102		1.3594	0.4617		0.3665	0.0526		0.0790	0.0040	down	0.4481	0.0017	down
**BdMAPKKK57**	0.4451	0.0026	down	0.0180	0.0000	down	0.9866	0.9438		2.6549	0.0021	up	0.4998	0.0162	
**BdMAPKKK58**	3.2629	0.0355		2.5461	0.0074	up	15.9661	0.0001	up	11.8081	0.0002	up	1.0075	0.9843	
**BdMAPKKK59**	2.7741	0.0004	up	0.7130	0.0128		1.0146	0.8904		2.8346	0.0007	up	22.7227	0.0009	up
**BdMAPKKK60**	0.2571	0.0035	down	0.1721	0.0001	down	0.0979	0.0844		0.2914	0.0000	down	0.2512	0.0003	down
**BdMAPKKK61**	0.2766	0.0213		0.1808	0.0020	down	0.4258	0.0549		0.7264	0.3220		0.6019	0.0163	
**BdMAPKKK62**	2.1944	0.0047	up	5.7740	0.0003	up	1.0710	0.6265		0.6332	0.0959		1.7133	0.0020	
**BdMAPKKK63**	1.6018	0.0007		2.4238	0.0098	up	1.5012	0.0888		0.8665	0.4293		1.6552	0.0051	
**BdMAPKKK64**	2.4080	0.0234		0.4333	0.0336		0.9368	0.8089		0.8446	0.4917		0.7179	0.1366	
**BdMAPKKK65**	2.5616	0.0000	up	0.7510	0.1909		0.7799	0.0619		0.2079	0.0047	down	0.4620	0.0018	down
**BdMAPKKK66**	0.9911	0.9822		0.5641	0.1936		1.3631	0.3626		1.5144	0.1518		0.1806	0.0000	down
**BdMAPKKK67**	3.2438	0.0000	up	0.6351	0.1206		0.5886	0.0209		0.9983	0.9959		1.4266	0.3216	
**BdMAPKKK68**	1.0993	0.3472		0.9169	0.2501		1.1269	0.2444		0.1731	0.0001	down	0.6500	0.0098	
**BdMAPKKK69**	1.6519	0.0913		5.7863	0.0017	up	2.8639	0.0105		16.1324	0.0002	up	11.8599	0.0000	up
**BdMAPKKK70**	5.5602	0.0020	up	1.3268	0.3222		0.9644	0.8928		0.4757	0.1625		1.8956	0.0001	
**BdMAPKKK71**	1.2343	0.0004		1.2049	0.1130		1.0046	0.4171		3.0369	0.0001	up	0.8391	0.1044	
**BdMAPKKK72**	2.7625	0.0036	up	20.9155	0.0001	up	1.6520	0.0431		3.4298	0.0026	up	0.9040	0.7735	
**BdMAPKKK73**	1.3406	0.3013		0.8911	0.6768		1.5459	0.1525		0.1823	0.0050	down	0.8812	0.5821	
**BdMAPKKK74**	1.1390	0.0724		0.4469	0.0005	down	0.5804	0.0009		0.4826	0.0002	down	0.8903	0.1240	
**BdMAPKKK75**	1.2199	0.2354		0.8382	0.0002		0.6177	0.0046		0.5097	0.0008		1.6524	0.0000	

Additional file 8C-2 Expression data of the *BdMAPKKK* family genes after abiotic stresses treatment.

	4°C 6h			45°C 6h			PEG 6h			H_2O_2 6h			Nacl 6h		
	fold-change	p-value	up/down	fold-change	p-value	up/down	fold-change	p-value	up/down	fold-change	p-value	up/down	fold-change	p-value	up/down
**BdMAPKKK1**	5.5153	0.0086	up	2.0798	0.0399		0.2702	0.0381		5.3520	0.0042	up	0.8052	0.0134	
**BdMAPKKK2**	2.6708	0.0029	up	2.8822	0.0021	up	0.3046	0.0116		0.6449	0.1179		1.6682	0.0002	
**BdMAPKKK3**	2.7702	0.0554		7.4887	0.0025	up	0.3376	0.1031		0.3973	0.1171		2.1256	0.0029	up
**BdMAPKKK4**	0.7652	0.0000		6.9043	0.0001	up	0.6969	0.0000		0.4561	0.0002	down	2.4092	0.0000	up
**BdMAPKKK5**	4.1296	0.0335		11.5528	0.0030	up	0.4315	0.0861		1.4220	0.3610		1.5646	0.0004	
**BdMAPKKK6**	2.3953	0.0011	up	3.5689	0.0229		0.1937	0.0112		0.3925	0.0211		2.1342	0.0019	up
**BdMAPKKK7**	1.4636	0.1401		2.7061	0.0096	up	0.1534	0.0108		0.3676	0.0218		1.4381	0.0037	
**BdMAPKKK8**	4.2186	0.0209		1.4221	0.4420		0.6742	0.4679		3.8294	0.0220		1.3473	0.0006	
**BdMAPKKK9**	0.8841	0.2422		0.8076	0.1225		0.1133	0.0003	down	0.3438	0.0004	down	1.6392	0.0020	
**BdMAPKKK10**	3.3510	0.0000	up	0.9922	0.9482		0.5811	0.0028		1.2902	0.0043		1.7383	0.0192	
**BdMAPKKK11**	2.4703	0.0000	up	1.9362	0.0001		0.5446	0.0242		1.2726	0.0018		1.0343	0.6267	
**BdMAPKKK12**	3.6646	0.0000	up	2.8386	0.0029	up	0.7562	0.0005		0.8270	0.3797		1.8337	0.0005	
**BdMAPKKK13**	2.7498	0.0057	up	1.2220	0.1080		0.5168	0.1218		0.5505	0.0039		1.4107	0.0004	
**BdMAPKKK14**	3.8265	0.0216		0.4883	0.0228		0.5749	0.1284		2.5812	0.0044	up	0.8052	0.0000	
**BdMAPKKK15**	4.4482	0.0005	up	1.4463	0.2047		0.5612	0.0041		0.7869	0.5504		1.6607	0.0010	
**BdMAPKKK16**	3.3186	0.0004	up	1.3990	0.1573		0.8247	0.1446		1.3013	0.3081		1.0639	0.0018	
**BdMAPKKK17**	2.4674	0.0054	up	1.8019	0.0753		0.2205	0.0047	down	0.4953	0.1200		2.2046	0.0017	up
**BdMAPKKK18**	2.1641	0.0024	up	8.0830	0.0001	up	0.5507	0.0077		0.2282	0.0197		1.0623	0.6860	
**BdMAPKKK19**	5.3386	0.0002	up	4.2367	0.0006	up	1.7135	0.0082		1.0023	0.9909		3.1967	0.0000	up
**BdMAPKKK20**	2.8196	0.0000	up	0.7601	0.0242		0.6566	0.0000		1.0654	0.8339		1.1507	0.0118	
**BdMAPKKK21**	24.5059	0.0000	up	26.1910	0.0001	up	2.1620	0.0059	up	1.1284	0.6735		3.2035	0.0020	up
**BdMAPKKK22**	3.7650	0.0388		2.9563	0.0586		1.1368	0.7956		2.2787	0.0996		1.0377	0.7944	
**BdMAPKKK23**	1.6486	0.0062		1.7846	0.0368		1.0843	0.5278		0.5728	0.0274		0.7407	0.0180	
**BdMAPKKK24**	0.7197	0.0711		0.5448	0.0099		0.0865	0.0000	down	0.0470	0.0003	down	0.6499	0.0016	
**BdMAPKKK25**	1.4531	0.0046		0.2541	0.0007	down	0.5695	0.0005		1.2982	0.2700		0.4825	0.0003	down

**BdMAPKKK26**	1.3009	0.0774		1.0970	0.3066		0.5938	0.0000		0.4122	0.0010	down	1.2862	0.0253	
**BdMAPKKK27**	0.9967	0.9762		9.9382	0.0002	up	0.3428	0.0000	down	0.3877	0.0444		1.4785	0.0013	
**BdMAPKKK28**	1.1605	0.6426		0.8663	0.5649		1.4148	0.1677		2.0913	0.0741		0.8152	0.0002	
**BdMAPKKK29**	5.7800	0.0002	up	1.3319	0.0585		0.8718	0.3148		1.2863	0.4307		1.5085	0.0001	
**BdMAPKKK30**	10.7775	0.0000	up	20.6463	0.0000	up	0.7664	0.2635		0.8918	0.1072		0.4583	0.0014	down
**BdMAPKKK31**	6.0997	0.0003	up	0.8980	0.5420		0.8058	0.1946		1.9838	0.0476		1.1482	0.2036	
**BdMAPKKK32**	2.0705	0.0112		1.0327	0.8754		1.1327	0.4309		1.5771	0.0896		1.7244	0.0916	
**BdMAPKKK33**	1.9482	0.0170		0.7405	0.1810		0.2031	0.0005	down	0.4366	0.0364		1.3677	0.0165	
**BdMAPKKK34**	7.0989	0.0073	up	1.1205	0.8103		0.8370	0.6741		2.9693	0.0214		0.9348	0.1629	
**BdMAPKKK35**	3.6920	0.0000	up	6.7243	0.0000	up	0.6648	0.1397		1.2143	0.0345		2.9151	0.0001	up
**BdMAPKKK36**	41.2858	0.0002	up	1.6507	0.1704		0.5103	0.0568		1.7173	0.1387		0.9132	0.3500	
**BdMAPKKK37**	6.2793	0.0000	up	3.8342	0.0013	up	1.1265	0.4330		1.5860	0.0461		1.0201	0.2669	
**BdMAPKKK38**	2.6360	0.0035	up	3.7989	0.0019	up	0.4865	0.0174		0.6473	0.3453		1.6925	0.0002	
**BdMAPKKK39**	6.5931	0.0000	up	2.6502	0.0002	up	1.4034	0.0614		1.3830	0.1365		1.8184	0.0000	
**BdMAPKKK40**	4.0695	0.0000	up	1.8566	0.0062		1.5610	0.0008		2.9001	0.0040	up	1.1540	0.0281	
**BdMAPKKK41**	2.7542	0.0043	up	1.8411	0.0102		1.0353	0.8165		4.9998	0.0003	up	1.2135	0.0245	
**BdMAPKKK42**	0.5215	0.1399		0.9137	0.8350		0.1074	0.0031	down	0.1139	0.0035	down	4.4810	0.0032	up
**BdMAPKKK43**	1.7120	0.0016		2.9715	0.0035	up	0.9772	0.8859		1.0582	0.3926		1.2055	0.0991	
**BdMAPKKK44**	7.3178	0.0010	up	2.5159	0.0339		0.6812	0.0276		0.8340	0.1778		1.4581	0.0073	
**BdMAPKKK45**	4.3505	0.0003	up	0.7399	0.0801		0.8658	0.5499		3.2358	0.0047	up	1.2762	0.0432	
**BdMAPKKK46**	2.4506	0.0030	up	2.0358	0.0399		0.5211	0.0187		0.7537	0.5150		2.4209	0.0171	
**BdMAPKKK47**	3.2757	0.0000	up	3.7856	0.0000	up	1.1203	0.1911		3.2338	0.0001	up	0.7667	0.0103	
**BdMAPKKK48**	0.9361	0.6008		0.2822	0.0023	down	0.6350	0.0943		1.5898	0.1076		0.8485	0.2297	
**BdMAPKKK49**	5.1204	0.0015	up	4.8541	0.0016	up	1.1381	0.5349		2.3621	0.0147		1.3600	0.0055	
**BdMAPKKK50**	0.4111	0.1937		1.5839	0.4193		0.0476	0.0027	down	0.0420	0.0011	down	4.8408	0.0026	up
**BdMAPKKK51**	9.0268	0.0002	up	5.6153	0.0000	up	2.7871	0.0002	up	44.8628	0.0000	up	71.7733	0.0000	up
**BdMAPKKK52**	15.6940	0.0001	up	2.2514	0.0074	up	0.5912	0.1056		0.7082	0.1264		3.4440	0.0085	up
**BdMAPKKK53**	1.7499	0.0046		33.5506	0.0001	up	3.4091	0.0017	up	7.8332	0.0007	up	9.8739	0.0007	up
**BdMAPKKK54**	3.7264	0.0038	up	5.6214	0.0016	up	0.0102	0.0001	down	0.8215	0.4401		0.4655	0.0394	

**BdMAPKKK55**	1.9840	0.0093		30.6414	0.0000	up	0.3934	0.0093	down	0.1068	0.0000	down	0.5423	0.1409	
**BdMAPKKK56**	0.9802	0.9440		3.6310	0.0434		0.4991	0.0689		0.5431	0.0829		1.1911	0.0635	
**BdMAPKKK57**	1.0600	0.6783		5.2932	0.0002	up	0.7272	0.0604		4.7358	0.0002	up	0.9242	0.7960	
**BdMAPKKK58**	2.9329	0.0297		74.5161	0.0004	up	7.7473	0.0018	up	8.8030	0.0010	up	95.7290	0.0003	up
**BdMAPKKK59**	1.9360	0.0031		3.8266	0.0002	up	3.6128	0.0001	up	0.0012	0.0000	down	0.4688	0.0034	down
**BdMAPKKK60**	5.2005	0.0403		7.3681	0.0583		4.3675	0.0589		29.2919	0.0037	up	0.8302	0.3417	
**BdMAPKKK61**	0.1678	0.0006	down	3.1397	0.0009	up	0.1296	0.0000	down	4.1031	0.0006	up	0.7536	0.2396	
**BdMAPKKK62**	3.6019	0.0109		6.3067	0.0020	up	1.6072	0.1312		2.7019	0.0299		1.5347	0.0000	
**BdMAPKKK63**	0.6020	0.0001		1.5316	0.1083		1.0794	0.6196		1.1994	0.4301		4.8071	0.0007	up
**BdMAPKKK64**	4.0324	0.0004	up	0.8608	0.3027		1.4089	0.0219		1.7598	0.0503		0.8452	0.0735	
**BdMAPKKK65**	0.8589	0.4964		1.5073	0.1077		0.1726	0.0009	down	0.2437	0.0026	down	2.6589	0.0000	up
**BdMAPKKK66**	0.8195	0.6337		3.4291	0.0000	up	0.6191	0.2546		2.5745	0.0038	up	0.9479	0.0234	
**BdMAPKKK67**	2.0593	0.0090	up	1.0418	0.8165		1.4489	0.0253		4.8362	0.0013	up	9.6044	0.0000	up
**BdMAPKKK68**	0.1166	0.0017	down	0.8557	0.5484		0.0750	0.0012	down	0.0430	0.0000	down	2.1324	0.0407	
**BdMAPKKK69**	2.2173	0.0854		12.6345	0.0017	up	5.5487	0.0065	up	18.4537	0.0023	up	16.6947	0.0000	up
**BdMAPKKK70**	5.9311	0.0015	up	3.1442	0.0057	up	0.8247	0.3839		0.6632	0.1940		3.7408	0.0000	up
**BdMAPKKK71**	1.0393	0.7611		5.3930	0.0001	up	0.7808	0.1095		5.1465	0.0003	up	0.9416	0.5932	
**BdMAPKKK72**	1.2869	0.1956		22.4148	0.0007	up	1.3383	0.1132		4.8590	0.0000	up	1.6950	0.0127	
**BdMAPKKK73**	0.4089	0.0416		3.7308	0.0252		0.1731	0.0027	down	0.8588	0.6071		1.3749	0.0195	
**BdMAPKKK74**	4.9966	0.0029	up	1.2279	0.5611		1.5951	0.1470		3.6479	0.0061	up	0.9124	0.4248	
**BdMAPKKK75**	6.2199	0.0080	up	1.6066	0.2840		1.1871	0.6431		2.6280	0.0760		1.3824	0.0025	

Additional file 8D Expression data of the *BdMAPK* family genes after heavy metal treatment.

	**Cdcl$_2$ 6h**			**Cdcl$_2$ 12h**			**$PbSO_4$ 6h**			**$PbSO_4$ 12h**			**Zncl$_2$ 6h**			**Zncl$_2$ 12h**		
	fold-change	p-value	up/down	fold-change	p-value	up/down	fold-change	p-value	up/down	fold-change	p-value	up/down	fold-change	p-value	up/down	fold-change	p-value	up/down
**BdMPK3**	3.6243	0.0000	up	4.6020	0.0000	up	1.3618	0.0001		0.4638	0.0000	down	0.6195	0.0000		0.7725	0.0016	
**BdMPK4**	1.7802	0.0001		0.8836	0.6185		2.9350	0.0000	up	0.5947	0.0860		2.0640	0.0000	up	0.9776	0.9312	
**BdMPK6**	2.1157	0.0002	up	1.6969	0.0026		0.9421	0.3676		0.6906	0.0048		0.6028	0.0006		0.8553	0.0752	
**BdMPK7-1**	2.4875	0.0001	up	0.8931	0.0674		1.4694	0.0003		0.2568	0.0000	down	0.6418	0.0002		0.5630	0.0010	
**BdMPK7-2**	2.0798	0.0001	up	2.4847	0.0001	up	0.9596	0.2588		1.0518	0.2278		0.6081	0.0000		1.3237	0.0019	
**BdMPK11**	1.7464	0.0003		0.9719	0.4202		1.9588	0.0032		0.4987	0.0000	down	1.2938	0.0038		0.8559	0.0729	
**BdMPK14**	1.8525	0.0000		1.9502	0.0001		0.8257	0.0000		0.8261	0.0005		0.4516	0.0000	down	0.9812	0.5042	
**BdMPK16**	1.8589	0.0005		0.6045	0.0026		2.1304	0.0002	up	0.4379	0.0004	down	1.5499	0.0010		0.8272	0.0737	
**BdMPK17**	1.3039	0.0022		1.0455	0.2209		0.9286	0.0542		0.6668	0.0003		0.8429	0.0065		0.6796	0.0017	
**BdMPK20-1**	1.9647	0.0003		0.5021	0.0012		2.3604	0.0002	up	0.3780	0.0003	down	1.5845	0.0015		0.7247	0.0090	
**BdMPK20-2**	2.7701	0.0012	up	0.7351	0.0002		3.4798	0.0006	up	0.5229	0.0000		1.8671	0.0069		1.2384	0.0011	
**BdMPK20-3**	2.3559	0.0001	up	0.9309	0.4672		0.8704	0.0142		0.4296	0.0000	down	0.5835	0.0001		0.6530	0.0001	
**BdMPK20-4**	2.0411	0.0020	up	0.6228	0.0000		2.5186	0.0008	up	0.4365	0.0000	down	1.7939	0.0042		0.6679	0.0004	
**BdMPK20-5**	2.6135	0.0004	up	0.9301	0.3386		2.2662	0.0001	up	0.3139	0.0000	down	1.4307	0.0039		0.6734	0.0008	
**BdMPK21-1**	1.1212	0.0145		1.1494	0.0431		0.8247	0.0049		0.9329	0.2218		0.6266	0.0008		1.1460	0.0610	

Additional file 8E Expression data of the *BdMAPKK* family genes after heavy metal treatment.

	**Cdcl$_2$ 6h**			**Cdcl$_2$ 12h**			**PbSO$_4$ 6h**			**PbSO$_4$ 12h**			**Zncl$_2$ 6h**			**Zncl$_2$ 12h**		
	fold-change	p-value	up/down	fold-change	p-value	up/down	fold-change	p-value	up/down	fold-change	p-value	up/down	fold-change	p-value	up/down	fold-change	p-value	up/down
**BdMKK1**	1.7771	0.0000		0.7502	0.1039		2.1849	0.0001	up	0.3525	0.0016	down	1.2345	0.0023		0.6216	0.0284	
**BdMKK3-1**	1.5512	0.0001		1.5512	0.0000		0.5568	0.0000		0.9372	0.0153		0.2983	0.0022	down	0.8800	0.0134	
**BdMKK3-2**	1.5433	0.0003		1.4241	0.0340		0.6713	0.0013		0.9843	0.0361		0.4582	0.0001	down	1.0628	0.0231	
**BdMKK3-3**	3.0541	0.0002	up	2.7296	0.0000	up	0.5501	0.0001		0.6019	0.0000		0.4004	0.0000	down	0.8294	0.0032	
**BdMKK4**	5.3306	0.0023	up	1.6177	0.0955		0.3239	0.0004	down	0.0916	0.0000	down	0.1380	0.0008	down	0.7167	0.2803	
**BdMKK5**	2.3659	0.1567		4.1116	0.0001	up	2.0957	0.4313		0.2746	0.0001	down	0.1842	0.0012	down	1.3997	0.0157	
**BdMKK6**	2.0155	0.0000	up	1.0116	0.4633		1.4506	0.0146		0.5566	0.0000		0.5749	0.0011		0.8785	0.0367	
**BdMKK10-1**	0.0394	0.4934		0.2063	0.0036	down	0.0042	0.2861		0.4505	0.0903		0.2275	0.7477		0.1499	0.1049	
**BdMKK10-2**	1.9628	0.0000		6.8192	0.0000	up	0.6851	0.0006		1.3103	0.0001		0.3738	0.0000	down	0.9615	0.3620	
**BdMKK10-3**	3.0922	0.0299		0.0881	0.2046		0.0552	0.1059		0.1175	0.0013	down	0.2790	0.0265		0.1006	0.0051	down
**BdMKK10-5**	1.4477	0.0003		28.9525	0.0000	up	0.8292	0.0036		0.5139	0.0230		0.4956	0.0000	down	1.6276	0.0010	

Additional file 8F Expression data of the *BdMAPKKK* family genes after heavy metal treatment.

	$Cdcl_2$ 6h			$Cdcl_2$ 12h			$PbSO_4$ 6h			$PbSO_4$ 12h			$Zncl_2$ 6h			$Zncl_2$ 12h		
	fold-change	p-value	up/down	fold-change	p-value	up/down	fold-change	p-value	up/down	fold-change	p-value	up/down	fold-change	p-value	up/down	fold-change	p-value	up/down
**BdMAPKKK1**	1.2431	0.0009		2.4113	0.0000	up	0.7340	0.0003		0.9920	0.8997		0.5372	0.0000		0.9856	0.6969	
**BdMAPKKK2**	0.5551	0.0001		0.5869	0.0080		1.5316	0.0005		0.4769	0.0002	down	0.9351	0.0980		0.4586	0.0001	down
**BdMAPKKK3**	1.4826	0.0413		1.1853	0.0253		1.4670	0.0332		0.3186	0.0000	down	0.7849	0.1032		0.3992	0.0000	down
**BdMAPKKK4**	1.5957	0.0000		1.6998	0.0021		1.0269	0.1499		0.2239	0.0000	down	0.3358	0.0000	down	0.3094	0.0000	down
**BdMAPKKK5**	0.6786	0.0008		0.8640	0.0015		1.3864	0.0004		0.6466	0.0001		0.8380	0.0037		0.7177	0.0002	
**BdMAPKKK6**	1.2113	0.0660		1.1313	0.2111		2.4393	0.0003	up	0.6023	0.0000		1.3101	0.0241		0.5007	0.0000	
**BdMAPKKK7**	0.7970	0.0019		1.3270	0.0001		1.8546	0.0000		0.7912	0.0001		1.0204	0.4580		0.9820	0.4328	
**BdMAPKKK8**	1.0372	0.3508		0.8993	0.0731		1.1396	0.0440		0.6942	0.0010		0.7519	0.0014		0.6699	0.0008	
**BdMAPKKK9**	0.6123	0.0026		0.4200	0.0001	down	2.7405	0.0001	up	0.7063	0.0004		1.6298	0.0019		0.5428	0.0001	
**BdMAPKKK10**	1.5687	0.0385		1.5409	0.0067		1.3047	0.1443		0.9154	0.3629		0.9223	0.6092		0.9030	0.2831	
**BdMAPKKK11**	1.0316	0.7230		1.0723	0.8081		1.0387	0.4309		0.8307	0.2352		0.7785	0.0021		0.7016	0.0551	
**BdMAPKKK12**	0.7622	0.0008		0.7854	0.0000		1.2139	0.0002		0.5949	0.0000		0.8123	0.0001		0.5543	0.0000	
**BdMAPKKK13**	0.9044	0.4314		0.9425	0.2076		0.9953	0.8221		0.8123	0.0073		0.8418	0.0863		0.7496	0.0019	
**BdMAPKKK14**	0.7593	0.0002		1.9528	0.0000		0.6168	0.0000		0.9359	0.1743		0.5289	0.0000		0.9045	0.0005	
**BdMAPKKK15**	0.3999	0.0000	down	0.6646	0.0015		1.2791	0.0008		0.8381	0.0109		1.0563	0.0675		0.6870	0.0006	
**BdMAPKKK16**	1.2060	0.1662		1.5777	0.0000		1.0691	0.0049		0.6373	0.0000		0.6356	0.0001		0.6395	0.0001	
**BdMAPKKK17**	0.5583	0.0004		0.4836	0.0012	down	1.8954	0.0002		0.6995	0.0042		1.4235	0.0024		0.5733	0.0008	
**BdMAPKKK18**	2.0101	0.0074	up	0.7119	0.0980		1.0671	0.6642		0.2126	0.0001	down	0.3333	0.0022	down	0.3019	0.0000	down
**BdMAPKKK19**	1.8074	0.0002		1.4803	0.0135		1.2353	0.0009		0.7141	0.0001		0.8226	0.0006		0.6719	0.0001	
**BdMAPKKK20**	1.4498	0.0012		2.0934	0.0000	up	0.9629	0.3483		0.8327	0.0011		0.7048	0.0003		0.8525	0.0292	
**BdMAPKKK21**	0.5758	0.0005		1.1860	0.0127		1.0947	0.1856		0.9168	0.0581		1.0765	0.1900		0.7595	0.0014	
**BdMAPKKK22**	1.5395	0.0000		2.2935	0.0000	up	0.9354	0.0993		1.0481	0.0482		0.5983	0.0001		0.9577	0.1663	
**BdMAPKKK23**	1.0344	0.5764		0.3937	0.0014	down	1.3292	0.0082		0.5274	0.0000		0.5369	0.0012		0.6852	0.0000	
**BdMAPKKK24**	0.8664	0.0469		0.5330	0.0185		0.9728	0.6372		0.7527	0.1559		0.6898	0.0097		0.6991	0.4082	
**BdMAPKKK25**	2.1476	0.0000	up	0.7424	0.0001		1.2786	0.0005		0.6874	0.0000		0.4492	0.0000	down	0.7937	0.0013	

**BdMAPKKK26**	1.2522	0.0034		0.7072	0.3367		1.3557	0.0121		0.2493	0.0001	down	0.3371	0.0000	down	0.1510	0.0011	down
**BdMAPKKK27**	1.7224	0.0055		2.5835	0.0001	up	0.9752	0.6184		0.3430	0.0000	down	0.3200	0.0000	down	0.4637	0.0001	down
**BdMAPKKK28**	1.2884	0.0025		1.0733	0.5106		1.2115	0.0004		1.0121	0.8921		0.7787	0.0128		1.4799	0.0005	
**BdMAPKKK29**	1.3224	0.0050		1.6630	0.0039		1.1610	0.0336		1.0159	0.8557		0.8203	0.0018		1.0040	0.9633	
**BdMAPKKK30**	2.2543	0.0024	up	5.3243	0.0179		0.2935	0.0003	down	0.4981	0.1667		0.2085	0.0025	down	0.9678	0.9308	
**BdMAPKKK31**	0.9671	0.6392		1.1939	0.0001		0.7750	0.1031		0.6999	0.0037		0.7526	0.0884		0.8706	0.1830	
**BdMAPKKK32**	2.9256	0.0121		3.2823	0.0000	up	1.3585	0.2824		0.8328	0.0214		0.7028	0.2360		0.8561	0.0364	
**BdMAPKKK33**	0.9990	0.9921		0.7010	0.0014		1.3652	0.0069		0.6134	0.0003		1.1474	0.0892		0.6635	0.0001	
**BdMAPKKK34**	1.7751	0.0000		4.5569	0.0000	up	0.7854	0.0002		0.8874	0.0620		0.4305	0.0000	down	0.9407	0.2604	
**BdMAPKKK35**	2.4369	0.0002	up	3.4252	0.0000	up	1.3286	0.0124		0.7442	0.0000		0.7642	0.0141		0.5430	0.0000	
**BdMAPKKK36**	1.3015	0.0198		0.8197	0.0282		1.6239	0.0014		0.3506	0.0000	down	0.9630	0.5769		0.1698	0.0018	down
**BdMAPKKK37**	1.3660	0.0004		1.8116	0.0003		1.2638	0.0121		1.0264	0.4143		0.8541	0.0431		0.8142	0.0029	
**BdMAPKKK38**	1.4730	0.0000		1.5646	0.0001		1.8619	0.0000		0.7215	0.0001		0.9518	0.4930		0.6658	0.0001	
**BdMAPKKK39**	2.0044	0.0000	up	2.3012	0.0003	up	0.8259	0.0101		1.0195	0.8254		0.6909	0.0007		1.2215	0.0546	
**BdMAPKKK40**	1.6241	0.0000		2.8649	0.0001	up	1.2019	0.0007		1.2111	0.0053		0.5927	0.0000		1.3073	0.0016	
**BdMAPKKK41**	1.7448	0.0006		2.6798	0.0000	up	0.9942	0.9189		1.0573	0.0497		0.5081	0.0002		1.0348	0.2697	
**BdMAPKKK42**	2.5215	0.0261		3.9275	0.0000	up	2.6089	0.0342		0.7894	0.5220		0.3328	0.0180		1.3265	0.0687	
**BdMAPKKK43**	1.9688	0.0000		1.7876	0.0008		2.3669	0.0000	up	0.6121	0.0004		0.8700	0.0024		0.5051	0.0001	
**BdMAPKKK44**	1.0241	0.4995		1.7268	0.0039		0.9546	0.7730		0.9493	0.5942		0.5544	0.0005		0.7687	0.0466	
**BdMAPKKK45**	3.0728	0.0001	up	1.8925	0.0346		1.0447	0.6070		0.6493	0.0004		0.6499	0.0048		0.6259	0.0000	
**BdMAPKKK46**	1.6036	0.0816		1.3365	0.0251		3.1941	0.0032	up	1.1902	0.0956		2.7062	0.0055	up	0.9599	0.6963	
**BdMAPKKK47**	1.5001	0.0002		2.6230	0.0001	up	1.1690	0.0056		1.9108	0.0003		0.4798	0.0001	down	1.8125	0.0006	
**BdMAPKKK48**	1.4040	0.0505		0.8355	0.0595		1.4286	0.0201		0.5480	0.0077		0.6171	0.0081		0.4843	0.0001	down
**BdMAPKKK49**	1.8436	0.0002		4.5185	0.0000	up	1.0806	0.2791		1.4479	0.0095		0.5934	0.0004		1.1175	0.0886	
**BdMAPKKK50**	1.4190	0.1216		0.7308	0.1503		0.5174	0.0087		0.1044	0.0002	down	0.8319	0.4164		0.1731	0.0006	down
**BdMAPKKK51**	52.5030	0.0000	up	290.7587	0.0000	up	0.9318	0.0002		0.5352	0.0003		0.4357	0.0000	down	0.4387	0.0001	down
**BdMAPKKK52**	15.9118	0.0000	up	8.8028	0.0003	up	0.6022	0.1171		0.2344	0.0001	down	1.6741	0.0579		0.3486	0.0055	down
**BdMAPKKK53**	41.9267	0.0000	up	909.7584	0.0000	up	0.8914	0.5360		3.8594	0.0001	up	0.6197	0.1004		15.6724	0.0011	up
**BdMAPKKK54**	0.8079	0.3635		2.3529	0.0082	up	45.1552	0.0001	up	0.1338	0.0001	down	0.1086	0.0007	down	0.6022	0.0678	

**BdMAPKKK55**	0.2109	0.0008	down	2.8384	0.0505		0.3788	0.0079	down	0.2825	0.0237		0.1693	0.0004	down	0.5519	0.2054	
**BdMAPKKK56**	0.7210	0.0162		0.3014	0.0004	down	1.4428	0.0056		0.6757	0.0045		1.1786	0.2113		0.9562	0.6327	
**BdMAPKKK57**	8.4316	0.0105		1.7063	0.0415		1.7637	0.1236		0.1351	0.0011	down	0.1564	0.0176		0.2426	0.0010	down
**BdMAPKKK58**	80.5300	0.0000	up	30.4128	0.0000	up	1.3384	0.2049		0.3715	0.0000	down	1.9140	0.0140		0.3268	0.0045	down
**BdMAPKKK59**	2.6465	0.0001	up	216.6854	0.0000	up	0.2402	0.0003	down	3.0613	0.0462		0.0056	0.0000	down	81.6736	0.0000	up
**BdMAPKKK60**	0.1904	0.0002	down	0.3808	0.0003	down	0.0405	0.0000	down	0.3358	0.0057	down	0.4084	0.0018	down	0.3787	0.0290	
**BdMAPKKK61**	1.4190	0.2611		0.7483	0.0999		0.8663	0.4899		0.2096	0.0003	down	0.2864	0.0030	down	0.4659	0.0049	down
**BdMAPKKK62**	0.9220	0.1206		2.6382	0.0000	up	0.6908	0.0001		1.6044	0.0001		0.5929	0.0081		1.5819	0.0533	
**BdMAPKKK63**	2.0576	0.0002	up	1.2090	0.2992		1.3323	0.0404		1.0163	0.8701		0.8348	0.0000		0.5962	0.0053	
**BdMAPKKK64**	1.5549	0.0002		1.6367	0.0001		0.9712	0.3466		0.9346	0.0730		0.6534	0.0001		0.8832	0.0087	
**BdMAPKKK65**	0.8578	0.0470		0.4234	0.0009	down	2.0634	0.0000	up	0.6313	0.0007		1.2950	0.0002		0.5737	0.0004	
**BdMAPKKK66**	1.7215	0.0000		0.5850	0.0044		0.7225	0.0000		0.1097	0.0000	down	0.3977	0.0000	down	0.1405	0.0000	down
**BdMAPKKK67**	3.0110	0.0000	up	2.9927	0.0006	up	1.4060	0.0000		0.7475	0.0193		0.5707	0.0000		0.3765	0.0002	down
**BdMAPKKK68**	0.0430	0.0002	down	0.1258	0.0007	down	0.8152	0.1475		0.2383	0.0068	down	0.6003	0.0163		0.2405	0.0058	down
**BdMAPKKK69**	15.7226	0.0000	up	49.7689	0.0000	up	1.4300	0.0372		0.5575	0.0045		0.4690	0.0013	down	0.6277	0.0282	
**BdMAPKKK70**	1.0122	0.8948		1.0501	0.7323		1.5453	0.0002		1.2908	0.0000		1.1901	0.0635		1.0267	0.2126	
**BdMAPKKK71**	4.9421	0.0001	up	9.3525	0.0000	up	0.9654	0.7607		0.3054	0.0002	down	0.2166	0.0001	down	0.4475	0.0014	down
**BdMAPKKK72**	5.9766	0.0049	up	6.7654	0.0008	up	0.9397	0.6250		0.3602	0.0253		0.3981	0.0021	down	0.5127	0.0699	
**BdMAPKKK73**	0.7570	0.0789		1.5779	0.0227		0.4547	0.0033	down	0.3425	0.0045	down	0.1988	0.0018	down	0.6750	0.0395	
**BdMAPKKK74**	1.9164	0.0001		2.4282	0.0004	up	0.7094	0.0007		0.9179	0.0234		0.6201	0.0007		1.0485	0.1149	
**BdMAPKKK75**	2.0363	0.0002	up	2.9244	0.0000	up	0.4759	0.0000	down	2.6893	0.0001	up	0.8740	0.1856		1.9209	0.0001	

Additional file 8G Expression data of the *BdMAPK* family genes after phytohormone treatment.

	ABA 3h			ABA 6h			JA 3h			JA 6h			SA 3h			SA 6h		
	fold-change	p-value	up/down	fold-change	p-value	up/down	fold-change	p-value	up/down	fold-change	p-value	up/down	fold-change	p-value	up/down	fold-change	p-value	up/down
**BdMPK3**	0.8304	0.0502		0.5477	0.0000		0.2014	0.2541		0.6355	0.0012		3.4890	0.0000	up	4.2512	0.0000	up
**BdMPK4**	0.3248	0.0002	down	1.3210	0.0019		0.1749	0.0000	down	0.3837	0.0010	down	0.3838	0.0006	down	1.7738	0.0002	
**BdMPK6**	0.9488	0.2667		0.5313	0.0204		0.8561	0.0186		0.5075	0.0001		0.7485	0.0006		1.0917	0.1128	
**BdMPK7-1**	1.5517	0.0267		1.2895	0.0429		2.1245	0.0057	up	0.5264	0.0114		0.2865	0.0015	down	0.2027	0.0042	down
**BdMPK7-2**	1.5076	0.5193		0.9864	0.9720		1.9634	0.2784		0.4789	0.1131		0.5883	0.3786		0.9841	0.9799	
**BdMPK11**	0.8250	0.2540		0.3835	0.0128		0.4246	0.0078	down	0.3523	0.0027	down	0.6983	0.0447		0.4376	0.0143	
**BdMPK14**	0.7145	0.0117		0.6775	0.0026		0.6602	0.0278		0.4531	0.0033	down	0.4961	0.0001	down	0.3935	0.0000	down
**BdMPK16**	1.5649	0.0001		0.9208	0.1027		1.1628	0.0632		0.7504	0.0002		1.3117	0.0015		0.5124	0.0008	
**BdMPK17**	2.0983	0.0000	up	1.4842	0.0004		0.7372	0.0002		0.4479	0.0000	down	2.0438	0.0000	up	2.6387	0.0000	up
**BdMPK20-1**	1.1031	0.0010		0.7684	0.0108		1.4820	0.0000		0.3554	0.0215		1.5773	0.0000		0.8679	0.0198	
**BdMPK20-2**	1.7402	0.0000		0.7487	0.0061		1.4645	0.0001		0.3813	0.0012	down	1.6492	0.0002		0.1692	0.0000	down
**BdMPK20-3**	0.8733	0.1567		1.0025	0.0208		1.3437	0.0170		0.4564	0.0068	down	1.2941	0.0298		0.4684	0.0013	down
**BdMPK20-4**	0.5421	0.0001		0.2763	0.0000	down	0.4757	0.0000	down	0.2799	0.0000	down	1.3620	0.0009		0.1394	0.0000	down
**BdMPK20-5**	0.9857	0.3732		0.4788	0.0000	down	2.1693	0.0000	up	1.1240	0.0007		0.1930	0.0000	down	0.0361	0.0000	down
**BdMPK21-1**	1.0989	0.5347		2.2029	0.0086	up	1.9789	0.0010		0.7718	0.1769		1.9662	0.0010		1.8224	0.0233	

Additional file 8H Expression data of the *BdMAPKK* family genes after phytohormone treatment.

	ABA 3h			ABA 6h			JA 3h			JA 6h			SA 3h			SA 6h		
	fold-change	p-value	up/down	fold-change	p-value	up/down	fold-change	p-value	up/down	fold-change	p-value	up/down	fold-change	p-value	up/down	fold-change	p-value	up/down
**BdMKK1**	0.7009	0.0010		0.3915	0.0000	down	0.7274	0.0016		0.3790	0.0000	down	0.8817	0.0380		0.6775	0.0001	
**BdMKK3-1**	1.5681	0.0036		1.0791	0.0443		1.7395	0.0003		0.5553	0.0001		1.8228	0.0017		1.8161	0.0001	
**BdMKK3-2**	2.5613	0.0003	up	0.5837	0.0029		2.2748	0.0002	up	0.2796	0.0002	down	3.2640	0.0001	up	1.1293	0.2162	
**BdMKK3-3**	7.7182	0.0001	up	1.8373	0.0008		2.1846	0.0023	up	0.1927	0.0001	down	9.7501	0.0000	up	3.3051	0.0000	up
**BdMKK4**	1.3858	0.2769		0.1286	0.0350		2.2490	0.3028		0.5185	0.5130		0.6938	0.1419		1.1496	0.8926	
**BdMKK5**	1.0300	0.7325		0.5360	0.0028		0.9095	0.3756		0.3874	0.0003	down	0.4968	0.0084	down	3.2689	0.0000	up
**BdMKK6**	1.1126	0.0699		0.9130	0.1385		0.9166	0.0377		0.2961	0.0009	down	1.3569	0.0003		0.3245	0.0000	down
**BdMKK10-1**	1.4719	0.6253		1.6477	0.4357		0.6712	0.7072		0.7120	0.6230		0.0901	0.2631		0.6234	0.4071	
**BdMKK10-2**	0.8002	0.0000		1.1647	0.0180		4.9562	0.0000	up	2.0235	0.0026	up	5.7786	0.0000	up	5.2924	0.0000	up
**BdMKK10-3**	1.8313	0.0629		1.9432	0.5237		0.0076	0.0000	down	0.1796	0.2067		0.3155	0.6545		9.9166	0.0742	
**BdMKK10-5**	0.4626	0.0006	down	0.5980	0.0006		1.1715	0.0001		0.6599	0.0275		1.6193	0.0865		3.0096	0.0001	up

Additional file 8I Expression data of the *BdMAPKKK* family genes after phytohormone treatment.

	ABA 3h			ABA 6h			JA 3h			JA 6h			SA 3h			SA 6h		
	fold-change	p-value	up/down	fold-change	p-value	up/down	fold-change	p-value	up/down	fold-change	p-value	up/down	fold-change	p-value	up/down	fold-change	p-value	up/down
**BdMAPKKK1**	0.3333	0.0454		0.7072	0.3858		0.4083	0.0039	down	0.8485	0.5362		0.7483	0.0691		0.9185	0.8129	
**BdMAPKKK2**	0.9366	0.4808		1.0635	0.8908		0.3124	0.0000	down	0.8053	0.2564		0.4294	0.0000	down	0.6926	0.1023	
**BdMAPKKK3**	2.6183	0.0321		2.4571	0.0497		0.3994	0.0001	down	1.0457	0.8897		0.9661	0.7310		1.5011	0.2456	
**BdMAPKKK4**	15.9114	0.0000	up	4.7318	0.0000	up	1.0336	0.1638		0.6434	0.1491		0.7269	0.0001		1.2321	0.0690	
**BdMAPKKK5**	0.6731	0.3461		0.8695	0.7855		0.5676	0.1765		0.6652	0.4318		1.3198	0.3788		1.5717	0.2383	
**BdMAPKKK6**	1.6193	0.0007		1.3382	0.0996		0.5660	0.0067		0.5711	0.1063		0.6192	0.0152		0.4290	0.0434	
**BdMAPKKK7**	0.9395	0.7320		1.1396	0.5718		0.5952	0.0189		0.8582	0.6423		0.5979	0.0207		0.8758	0.5993	
**BdMAPKKK8**	0.4459	0.1738		1.0626	0.8868		0.3754	0.0472		0.3551	0.0602		0.3485	0.0459		0.4829	0.1304	
**BdMAPKKK9**	0.8523	0.0612		0.8188	0.0688		0.2845	0.0005	down	0.4866	0.0020	down	0.5229	0.0138		0.4468	0.0136	
**BdMAPKKK10**	0.7328	0.2579		0.8413	0.1575		0.3968	0.0145		0.4083	0.0189		0.5843	0.0673		0.4992	0.0277	
**BdMAPKKK11**	0.5557	0.0311		0.8124	0.0206		0.2674	0.0003	down	0.2867	0.0036	down	0.2224	0.0011	down	0.2039	0.0037	down
**BdMAPKKK12**	1.0826	0.5588		1.4106	0.0000		0.6209	0.0145		0.8534	0.0000		1.0390	0.7535		0.8557	0.1965	
**BdMAPKKK13**	0.8739	0.7103		0.8438	0.1630		0.5294	0.1259		0.7444	0.0197		0.7473	0.4201		0.6911	0.0142	
**BdMAPKKK14**	0.9979	0.9946		1.2914	0.0064		0.8054	0.4912		0.7699	0.0072		0.5430	0.0803		0.6818	0.2084	
**BdMAPKKK15**	0.7773	0.1678		1.1608	0.0677		0.3341	0.0010	down	0.8763	0.1646		1.0852	0.5600		0.9053	0.3782	
**BdMAPKKK16**	0.7492	0.1866		0.8946	0.2772		0.3995	0.0027	down	0.5056	0.0024		0.7321	0.0761		0.8724	0.2153	
**BdMAPKKK17**	1.3086	0.4306		1.1690	0.4558		0.5825	0.1317		0.8477	0.3677		0.9831	0.9553		0.3175	0.0518	
**BdMAPKKK18**	3.1890	0.0045	up	1.4946	0.0083		0.6172	0.0748		0.5368	0.0300		0.4111	0.0588		0.7432	0.2332	
**BdMAPKKK19**	1.3113	0.0839		2.1858	0.0021	up	0.4481	0.0004	down	0.6285	0.0120		1.3975	0.0157		1.8271	0.0115	
**BdMAPKKK20**	0.8258	0.3798		0.9002	0.1654		0.5232	0.0250		0.5556	0.0000		0.4508	0.0128		0.8322	0.0879	
**BdMAPKKK21**	0.9801	0.9267		3.1706	0.0005	up	2.5928	0.0160		5.5680	0.0001	up	4.0392	0.0023	up	7.4201	0.0001	up
**BdMAPKKK22**	0.7061	0.5025		1.3055	0.5603		0.8110	0.5383		0.6632	0.4162		0.6363	0.3659		1.0950	0.8257	
**BdMAPKKK23**	1.6943	0.0122		1.5107	0.0104		0.2991	0.0016	down	0.3389	0.0003	down	0.1161	0.0000	down	0.1067	0.0001	down
**BdMAPKKK24**	0.5788	0.0396		0.0889	0.0003	down	0.3693	0.0056	down	0.1561	0.0006	down	0.1742	0.0008	down	0.0767	0.0001	down
**BdMAPKKK25**	0.6433	0.0029		0.6330	0.0108		0.2792	0.0004	down	0.2667	0.0001	down	0.1183	0.0000	down	0.1584	0.0027	down

**BdMAPKKK26**	1.6724	0.0015		0.8740	0.0278		0.3092	0.0021	down	0.1079	0.0016	down	2.4286	0.0002	up	0.7090	0.1127	
**BdMAPKKK27**	5.6625	0.0004	up	1.4965	0.0005		4.3023	0.0044	up	1.6838	0.0073		4.1754	0.0091	up	1.2491	0.4795	
**BdMAPKKK28**	0.7146	0.0064		1.4187	0.1526		0.5345	0.0018		0.7813	0.2620		0.3737	0.0000	down	0.8102	0.3305	
**BdMAPKKK29**	0.6444	0.3149		0.8808	0.1500		0.4720	0.1201		0.7634	0.0162		0.7477	0.4843		0.8266	0.0912	
**BdMAPKKK30**	2.3066	0.0286		0.2982	0.0199		1.4048	0.0000		0.1822	0.0000	down	0.5150	0.0635		3.1828	0.0006	up
**BdMAPKKK31**	0.4075	0.0505		0.7096	0.0390		0.3291	0.0313		0.4870	0.0043	down	0.5195	0.1134		1.0050	0.9761	
**BdMAPKKK32**	0.9608	0.9157		1.4714	0.0417		0.3724	0.0524		0.6001	0.0177		0.4188	0.0657		0.6148	0.0264	
**BdMAPKKK33**	0.5344	0.0454		0.4275	0.0033	down	0.4564	0.0186		0.6863	0.0491		0.4625	0.0185		0.2163	0.0028	down
**BdMAPKKK34**	0.5280	0.3208		0.6938	0.4050		0.9590	0.9299		0.4060	0.0612		1.0254	0.9639		1.4826	0.3107	
**BdMAPKKK35**	0.7636	0.1934		1.6583	0.0354		1.0150	0.9273		1.0238	0.8437		1.0694	0.7422		2.0408	0.0002	up
**BdMAPKKK36**	0.5225	0.0379		0.9817	0.9440		0.4593	0.0185		1.0455	0.8644		0.4662	0.0200		0.4572	0.0389	
**BdMAPKKK37**	0.7411	0.4310		1.4613	0.0241		0.5552	0.1534		0.9417	0.3725		1.1754	0.6488		2.3958	0.0002	up
**BdMAPKKK38**	1.0583	0.7879		1.0698	0.6871		0.5730	0.0326		0.6941	0.0841		0.6982	0.1342		0.8136	0.3892	
**BdMAPKKK39**	0.9194	0.7904		1.4223	0.0067		0.5922	0.1532		0.8743	0.2905		0.7473	0.4312		0.9082	0.6338	
**BdMAPKKK40**	0.7504	0.5077		1.6730	0.0009		1.0899	0.8421		1.3080	0.0164		1.4131	0.4141		3.3623	0.0005	up
**BdMAPKKK41**	0.6433	0.0343		1.3696	0.0578		0.7315	0.0356		1.0552	0.6887		0.7872	0.0137		1.7505	0.0143	
**BdMAPKKK42**	0.7526	0.5276		1.8139	0.1685		2.8013	0.0395		1.6918	0.2743		6.9559	0.0008	up	0.4438	0.1074	
**BdMAPKKK43**	1.4587	0.1263		1.9717	0.0008		1.0623	0.7651		0.7203	0.0326		0.6145	0.0624		0.6384	0.0587	
**BdMAPKKK44**	0.5128	0.1304		0.7827	0.2913		0.5828	0.1800		1.1529	0.4801		1.3331	0.3945		1.7779	0.0932	
**BdMAPKKK45**	0.9578	0.8707		0.8947	0.4493		0.7660	0.2603		0.6951	0.0400		0.4091	0.0117		0.5696	0.0135	
**BdMAPKKK46**	0.7059	0.3116		1.0030	0.9851		0.6627	0.2343		1.2697	0.1485		0.6732	0.2511		0.6973	0.2057	
**BdMAPKKK47**	0.3781	0.0175		0.7898	0.0042		0.1431	0.0017	down	0.1790	0.0000	down	0.5403	0.0689		1.2468	0.0553	
**BdMAPKKK48**	0.6755	0.0419		0.6550	0.0223		0.5310	0.0038		0.8108	0.1564		0.2106	0.0000	down	0.3543	0.0020	down
**BdMAPKKK49**	0.6020	0.1919		0.8247	0.3696		0.7014	0.3207		0.8781	0.5370		0.9584	0.8968		1.3685	0.2107	
**BdMAPKKK50**	4.6665	0.0010	up	1.2163	0.6115		1.2773	0.3196		0.1864	0.0264		0.8800	0.3201		0.1942	0.0178	
**BdMAPKKK51**	1.0284	0.9226		1.4894	0.0050		30.8565	0.0002	up	11.3941	0.0000	up	27.7630	0.0002	up	35.1312	0.0000	up
**BdMAPKKK52**	2.3627	0.0156		1.4664	0.3315		79.5148	0.0000	up	16.2239	0.0001	up	11.9021	0.0006	up	1.5931	0.1679	
**BdMAPKKK53**	9.5582	0.0003	up	3.4782	0.0004	up	8.1960	0.0000	up	7.6233	0.0001	up	15.2253	0.0000	up	12.2872	0.0000	up
**BdMAPKKK54**	485.1608	0.0000	up	1753.9443	0.0000	up	1.5292	0.2829		10.0388	0.0005	up	491.4942	0.0001	up	2.7340	0.0208	

**BdMAPKKK55**	0.7657	0.3301		0.6206	0.0372		6.3452	0.0013	up	25.0828	0.0003	up	0.7208	0.2793		16.1444	0.0000	up
**BdMAPKKK56**	0.5554	0.1946		0.5617	0.1320		0.2909	0.0432		0.5322	0.0928		0.1861	0.0106		0.2252	0.0222	
**BdMAPKKK57**	1.3125	0.1210		1.1164	0.3848		0.7276	0.2901		0.1451	0.0000	down	0.1175	0.0038	down	0.8553	0.2687	
**BdMAPKKK58**	105.8135	0.0000	up	211.0281	0.0001	up	3.0807	0.0080	up	6.4661	0.0087	up	3.6675	0.0032	up	18.6544	0.0005	up
**BdMAPKKK59**	2.1559	0.0028	up	8.0272	0.0001	up	0.0030	0.0000	down	8.8270	0.0000	up	0.0028	0.0000	down	1.3400	0.1798	
**BdMAPKKK60**	0.4240	0.2594		0.6303	0.7502		0.9996	0.9941		0.5386	0.3547		0.0485	0.0154		0.7036	0.6662	
**BdMAPKKK61**	0.4391	0.0312		0.1348	0.0012	down	0.5115	0.0662		0.6492	0.3388		0.0409	0.0005	down	1.5916	0.0238	
**BdMAPKKK62**	1.4462	0.0656		2.1782	0.0365		0.5912	0.0335		0.5405	0.0794		1.7911	0.0115		2.6233	0.0479	
**BdMAPKKK63**	2.6435	0.0002	up	1.8348	0.0017		4.4816	0.0001	up	1.5855	0.0148		27.8611	0.0000	up	9.7870	0.0001	up
**BdMAPKKK64**	1.0510	0.8483		1.5584	0.0166		0.6566	0.2964		0.8056	0.0882		1.0766	0.7685		1.8540	0.0037	
**BdMAPKKK65**	1.5069	0.0043		1.5436	0.0792		0.6081	0.0001		0.7329	0.2201		0.8073	0.0042		0.2577	0.0177	
**BdMAPKKK66**	1.5780	0.3269		0.6570	0.2078		1.2669	0.3597		0.2896	0.0000	down	0.8292	0.4739		1.6342	0.0014	
**BdMAPKKK67**	0.9825	0.7026		0.5433	0.0262		7.8679	0.0007	up	7.9706	0.0000	up	1.3765	0.0016		1.8403	0.0285	
**BdMAPKKK68**	0.1467	0.0050	down	0.9126	0.7626		0.2924	0.0038	down	0.0722	0.0001	down	0.3610	0.0374		0.5936	0.0107	
**BdMAPKKK69**	6.5310	0.0026	up	27.4913	0.0003	up	2.2873	0.0576		3.6320	0.0103		1.2232	0.4422		3.5478	0.0112	
**BdMAPKKK70**	1.5543	0.2179		1.4600	0.1137		0.4882	0.0478		0.6309	0.0926		1.6356	0.1086		1.1716	0.6950	
**BdMAPKKK71**	0.8535	0.0127		0.5158	0.0049		0.6929	0.0043		0.3513	0.0011	down	0.6121	0.0002		1.4971	0.0279	
**BdMAPKKK72**	1.5776	0.0494		0.7324	0.0071		1.1830	0.3244		0.8037	0.0752		1.2911	0.3627		8.1073	0.0002	up
**BdMAPKKK73**	3.1498	0.0084	up	2.1853	0.0471		1.0952	0.7225		0.9143	0.7507		1.1434	0.6023		0.8358	0.6877	
**BdMAPKKK74**	0.6691	0.0016		1.1118	0.7113		0.6851	0.5515		0.6132	0.1167		0.3335	0.0003	down	0.9082	0.7419	
**BdMAPKKK75**	0.5367	0.0020		0.6938	0.4054		0.3931	0.0265		0.5602	0.1688		0.5684	0.0009		0.6987	0.3580	

Additional file 8J Expression data of the *BdMAPK* family genes after biotic stresses treatment.

	pH14 4h			pH14 12h			Guy11 4h			Guy11 12h			F0968 4h			F0968 12h		
	fold-change	p-value	up/down	fold-change	p-value	up/down	fold-change	p-value	up/down	fold-change	p-value	up/down	fold-change	p-value	up/down	fold-change	p-value	up/down
**BdMPK3**	0.7979	0.0595		0.6917	0.0002		0.9005	0.0433		0.9745	0.4045		0.4990	0.0000	down	0.5982	0.0001	
**BdMPK4**	0.4587	0.0000	down	0.2845	0.0000	down	0.3316	0.0000	down	0.4940	0.0001	down	0.2345	0.0000	down	0.3985	0.0001	down
**BdMPK6**	0.6426	0.0010		0.4205	0.0000	down	0.5785	0.0001		0.8121	0.0093		0.3703	0.0000	down	0.4343	0.0001	down
**BdMPK7-1**	0.4386	0.0011	down	0.3413	0.0008	down	0.2765	0.0001	down	0.6092	0.0222		0.2797	0.0001	down	0.1862	0.0000	down
**BdMPK7-2**	1.0537	0.8439		0.4893	0.0052	down	1.0819	0.5785		0.9895	0.9399		0.3308	0.0010	down	0.3640	0.0081	down
**BdMPK11**	0.3910	0.0034	down	0.2130	0.0014	down	0.2413	0.0001	down	0.4901	0.1006		0.2343	0.0003	down	0.6168	0.0050	
**BdMPK14**	1.5592	0.1470		0.8099	0.3418		0.8486	0.3367		1.2002	0.6321		0.7059	0.0353		0.7307	0.0105	
**BdMPK16**	0.3713	0.0001	down	0.3290	0.0005	down	0.4860	0.0006	down	0.8165	0.0285		0.2041	0.0092	down	0.2420	0.0000	down
**BdMPK17**	1.6681	0.0125		0.8307	0.1936		0.8763	0.5334		1.2736	0.1208		0.6319	0.0446		0.7265	0.0550	
**BdMPK20-1**	1.0285	0.1209		0.4862	0.0249		0.8279	0.0006		1.6843	0.0000		0.6526	0.0004		0.9621	0.1188	
**BdMPK20-2**	0.2495	0.0004	down	0.3546	0.0005	down	0.3568	0.0006	down	0.5251	0.0043		0.2821	0.0002	down	0.3364	0.0005	down
**BdMPK20-3**	0.6164	0.0028		0.2747	0.0000	down	0.6067	0.0011		1.0175	0.8244		0.3800	0.0002	down	0.5323	0.0004	
**BdMPK20-4**	0.7066	0.0056		0.2454	0.0024	down	0.4066	0.0002	down	0.9795	0.8158		0.3016	0.0001	down	0.3662	0.0001	down
**BdMPK20-5**	0.2955	0.0001	down	0.4264	0.0004	down	0.4782	0.0010	down	0.7766	0.0663		0.3501	0.0002	down	0.3593	0.0004	down
**BdMPK21-1**	0.2129	0.0001	down	0.1453	0.0000	down	0.2672	0.0001	down	0.3117	0.0003	down	0.1852	0.0000	down	0.1800	0.0000	down

Additional file 8K Expression data of the *BdMAPKK* family genes after biotic stresses treatment.

	pH14 4h			pH14 12h			Guy11 4h			Guy11 12h			F0968 4h			F0968 12h		
	fold-change	p-value	up/down	fold-change	p-value	up/down	fold-change	p-value	up/down	fold-change	p-value	up/down	fold-change	p-value	up/down	fold-change	p-value	up/down
**BdMKK1**	0.3320	0.0000	down	0.1891	0.0021	down	0.2760	0.0000	down	0.4376	0.0001	down	0.2435	0.0000	down	0.2549	0.0000	down
**BdMKK3-1**	0.8628	0.2745		0.5024	0.0045		0.7228	0.0512		0.9237	0.5354		0.5837	0.0103		0.8372	0.2001	
**BdMKK3-2**	0.8488	0.0476		0.5105	0.0032		0.5965	0.0028		1.1520	0.1680		0.4329	0.0003	down	0.7692	0.1373	
**BdMKK3-3**	0.2753	0.0000	down	0.1091	0.0000	down	0.1804	0.0000	down	0.3046	0.0000	down	0.1315	0.0000	down	0.1858	0.0000	down
**BdMKK4**	0.2185	0.0475		0.3573	0.0127		1.3496	0.5149		1.7446	0.0918		1.3321	0.7642		1.1048	0.8256	
**BdMKK5**	0.3076	0.0004	down	0.2909	0.0003	down	0.2850	0.0003	down	0.5644	0.0052		0.3221	0.0010	down	0.2322	0.0003	down
**BdMKK6**	1.0055	0.9726		0.4283	0.0049	down	0.7114	0.0889		0.9206	0.6571		0.6089	0.0326		0.7533	0.1407	
**BdMKK10-1**	0.1589	0.2668		1.0641	0.9364		1.9835	0.0951		0.0306	0.0018	down	3.9837	0.0206		0.0735	0.0041	down
**BdMKK10-2**	1.3642	0.0016		0.4706	0.0000	down	1.0849	0.0051		1.4490	0.0009		0.7104	0.0000		0.8312	0.0064	
**BdMKK10-3**	0.0436	0.0001	down	0.0570	0.0044	down	0.0037	0.0000	down	0.8376	0.4960		0.0398	0.0206		0.1726	0.0010	down
**BdMKK10-5**	12.9862	0.0000	up	3.5376	0.0002	up	5.4003	0.0001	up	11.4780	0.0000	up	2.8447	0.0054	up	5.2509	0.0001	up

Additional file 8L Expression data of the *BdMAPKKK* family genes after biotic stresses treatment.

	pH14 4h			pH14 12h			Guy11 4h			Guy11 12h			F0968 4h			F0968 12h		
	fold-change	p-value	up/down	fold-change	p-value	up/down	fold-change	p-value	up/down	fold-change	p-value	up/down	fold-change	p-value	up/down	fold-change	p-value	up/down
**BdMAPKKK1**	0.9199	0.7568		0.4189	0.0283		0.4070	0.0186		1.1249	0.0038		0.9624	0.4118		1.7119	0.0297	
**BdMAPKKK2**	0.7554	0.3757		0.3020	0.0040	down	0.3765	0.0028	down	0.8900	0.1435		0.4050	0.0045	down	0.9957	0.9179	
**BdMAPKKK3**	0.6996	0.2561		0.6921	0.2844		0.5051	0.0319		1.3163	0.2942		0.4806	0.0340		0.7632	0.3270	
**BdMAPKKK4**	1.0555	0.7842		0.9994	0.9976		0.6424	0.0600		1.6179	0.0382		0.6785	0.0682		1.4188	0.0887	
**BdMAPKKK5**	2.7292	0.0710		1.4731	0.4410		1.8456	0.2922		4.4481	0.0301		2.4691	0.1301		2.7155	0.0551	
**BdMAPKKK6**	1.6949	0.0755		1.0081	0.9705		0.8533	0.5074		2.3667	0.0169		0.8347	0.4604		2.3085	0.0162	
**BdMAPKKK7**	1.6573	0.2286		0.9343	0.8470		0.8959	0.7262		2.4107	0.0944		1.2786	0.5209		3.0715	0.0193	
**BdMAPKKK8**	0.6763	0.2873		0.5104	0.0750		0.5015	0.0680		0.7578	0.3763		0.6268	0.1682		1.2040	0.6303	
**BdMAPKKK9**	1.8590	0.0688		1.1802	0.5804		1.0685	0.7555		2.2315	0.0161		1.4721	0.1515		2.0361	0.0321	
**BdMAPKKK10**	1.0563	0.8481		0.7581	0.3744		0.6515	0.1965		1.1983	0.5363		0.7633	0.3655		1.2075	0.5162	
**BdMAPKKK11**	0.5880	0.0619		0.3453	0.0093	down	0.3622	0.0065	down	0.7580	0.2650		0.5238	0.0357		1.0881	0.7940	
**BdMAPKKK12**	0.6450	0.1345		0.4282	0.0239		0.3152	0.0078	down	0.8261	0.5871		0.6120	0.2118		0.6576	0.1585	
**BdMAPKKK13**	0.4194	0.0027	down	0.3322	0.0020	down	0.2703	0.0007	down	0.6195	0.0660		0.4276	0.0031	down	0.5915	0.0284	
**BdMAPKKK14**	0.6023	0.2028		0.3871	0.0472		0.1578	0.0109		0.3660	0.0366		0.3144	0.0447		0.3196	0.0360	
**BdMAPKKK15**	0.8324	0.3952		0.3831	0.0080	down	0.3190	0.0055	down	0.9343	0.7440		0.5779	0.0623		0.6229	0.1387	
**BdMAPKKK16**	0.8567	0.0722		0.4571	0.0002	down	0.3280	0.0001	down	0.8668	0.4488		0.6262	0.0626		0.8517	0.1830	
**BdMAPKKK17**	0.7589	0.2364		0.6000	0.0622		0.6357	0.0497		1.6752	0.0246		0.8013	0.2042		1.0047	0.9808	
**BdMAPKKK18**	1.4004	0.0140		0.8410	0.3340		0.5392	0.0020		1.4883	0.1860		0.7808	0.2770		1.2049	0.3757	
**BdMAPKKK19**	0.9696	0.3383		0.4357	0.0000	down	0.3809	0.0000	down	0.7220	0.0108		0.4950	0.0090	down	0.7976	0.0001	
**BdMAPKKK20**	0.8917	0.2983		0.5454	0.0478		0.5416	0.0328		0.9640	0.8350		0.7101	0.0334		0.7675	0.4723	
**BdMAPKKK21**	6.3110	0.0010	up	3.5020	0.0049	up	3.5386	0.0052	up	6.7001	0.0009	up	3.7510	0.0063	up	3.0596	0.0247	
**BdMAPKKK22**	0.8408	0.6232		0.5373	0.0828		0.4719	0.0581		0.8417	0.5375		0.9016	0.7053		0.4904	0.0030	down
**BdMAPKKK23**	0.1433	0.0000	down	0.2606	0.0002	down	0.2249	0.0000	down	0.3470	0.0004	down	0.2318	0.0005	down	0.1301	0.0000	down
**BdMAPKKK24**	0.1915	0.0001	down	0.3254	0.0028	down	0.1608	0.0000	down	0.3479	0.0221		0.9541	0.8785		0.3632	0.0027	down
**BdMAPKKK25**	0.1129	0.0066	down	0.2536	0.0109		0.1684	0.0066	down	0.2452	0.0248		0.3975	0.0639		0.1516	0.0005	down

**BdMAPKKK26**	0.2775	0.0004	down	0.0643	0.0000	down	0.1948	0.0008	down	1.0908	0.4819		0.1702	0.0001	down	0.4176	0.0304	
**BdMAPKKK27**	1.7584	0.1327		1.0513	0.8752		0.6295	0.2001		1.5834	0.1986		0.8122	0.6565		0.9673	0.9311	
**BdMAPKKK28**	0.3444	0.0195		0.3489	0.0243		0.2622	0.0115		0.2787	0.0086	down	0.3764	0.0380		0.2628	0.0169	
**BdMAPKKK29**	0.7312	0.0164		0.3360	0.0003	down	0.3055	0.0002	down	0.7594	0.1689		0.4756	0.0314		0.4741	0.0491	
**BdMAPKKK30**	0.1871	0.0163		0.2316	0.0014	down	0.0550	0.0002	down	0.1988	0.0010	down	0.1017	0.0002	down	0.1375	0.0069	down
**BdMAPKKK31**	0.2488	0.0002	down	0.1853	0.0001	down	0.1611	0.0000	down	0.2406	0.0002	down	0.1888	0.0013	down	0.1956	0.0000	down
**BdMAPKKK32**	0.5822	0.0402		0.3119	0.0033	down	0.2345	0.0013	down	0.6124	0.0590		0.3981	0.0071	down	0.4047	0.0230	
**BdMAPKKK33**	0.7227	0.1497		0.6004	0.1070		0.4347	0.0104		1.3081	0.3283		0.8287	0.4496		0.7339	0.3064	
**BdMAPKKK34**	0.4499	0.0348		0.3545	0.0152		0.2492	0.0041	down	0.6304	0.0954		0.4224	0.0106		0.3365	0.0011	down
**BdMAPKKK35**	1.0858	0.8470		0.8355	0.6828		0.7830	0.5319		1.3837	0.4561		0.8002	0.6479		0.9675	0.9362	
**BdMAPKKK36**	1.7503	0.1477		1.2379	0.5042		0.7940	0.6127		2.5827	0.0235		1.6105	0.2354		0.8283	0.5197	
**BdMAPKKK37**	1.3712	0.2776		0.6505	0.1710		0.6604	0.1699		1.6682	0.1327		0.7890	0.3946		0.7095	0.2875	
**BdMAPKKK38**	0.8969	0.7639		0.5548	0.1746		0.5125	0.1112		1.1406	0.7267		0.7133	0.3977		0.8506	0.6639	
**BdMAPKKK39**	1.7429	0.1710		0.7642	0.4660		0.7652	0.4625		1.9593	0.1573		0.7793	0.5313		1.0909	0.8056	
**BdMAPKKK40**	0.9827	0.9564		0.5655	0.1276		0.4407	0.0526		1.1232	0.7229		0.9830	0.9571		0.7822	0.4558	
**BdMAPKKK41**	0.6056	0.0007		0.4745	0.0000	down	0.4066	0.0007	down	0.6641	0.0007		0.4971	0.0001	down	0.4047	0.0003	down
**BdMAPKKK42**	2.0106	0.0052	up	0.1903	0.0001	down	0.3515	0.0003	down	0.0511	0.0005	down	1.3517	0.0292		1.1671	0.3964	
**BdMAPKKK43**	1.1464	0.6679		1.0385	0.9055		0.9268	0.8335		1.3491	0.3684		0.9200	0.7953		0.9632	0.9354	
**BdMAPKKK44**	2.0902	0.0047	up	0.8502	0.2699		1.0384	0.8516		2.2071	0.0117		1.5517	0.0025		1.4790	0.0255	
**BdMAPKKK45**	0.8037	0.4784		0.7277	0.3192		0.5486	0.1005		0.6304	0.1899		1.0009	0.9975		0.6927	0.2971	
**BdMAPKKK46**	0.7209	0.0686		0.3921	0.0042	down	0.4480	0.0029	down	0.9914	0.9476		0.6569	0.0291		0.5669	0.0250	
**BdMAPKKK47**	1.7628	0.1599		0.7247	0.3723		0.9051	0.7448		1.8695	0.0999		1.5768	0.1866		1.2873	0.4411	
**BdMAPKKK48**	0.4742	0.0070	down	0.5184	0.0070		0.3805	0.0041	down	0.8953	0.4551		0.6706	0.0471		0.4619	0.0350	
**BdMAPKKK49**	1.4751	0.1665		0.6873	0.1673		0.6342	0.1232		1.7989	0.0649		0.9847	0.9544		0.9396	0.8296	
**BdMAPKKK50**	0.2115	0.0081	down	0.2514	0.0207		0.1167	0.0031	down	0.2832	0.0285		0.4330	0.0588		0.4457	0.0733	
**BdMAPKKK51**	15.6062	0.0000	up	7.7397	0.0003	up	5.8440	0.0001	up	20.6489	0.0000	up	2.0921	0.0047	up	1.2613	0.1569	
**BdMAPKKK52**	5.5717	0.0014	up	1.5404	0.0084		0.0800	0.0000	down	0.8214	0.1441		1.1264	0.3964		0.1605	0.0049	down
**BdMAPKKK53**	7.5337	0.0003	up	10.8535	0.0000	up	3.4463	0.0255		11.8390	0.0004	up	11.6055	0.0000	up	1.7144	0.0450	
**BdMAPKKK54**	0.3622	0.0062	down	0.1047	0.0012	down	0.1821	0.0007	down	0.4404	0.0213		0.2119	0.0016	down	2.6019	0.0028	up

**BdMAPKKK55**	2.4710	0.0077	up	2.3554	0.0023	up	1.7998	0.0189		8.5951	0.0049	up	2.5588	0.0021	up	2.5857	0.0038	up
**BdMAPKKK56**	0.2944	0.0000	down	0.1762	0.0000	down	0.1309	0.0002	down	0.4181	0.0000	down	0.2500	0.0000	down	0.2136	0.0000	down
**BdMAPKKK57**	0.4722	0.0023	down	0.2307	0.0004	down	0.1694	0.0010	down	0.5287	0.0010		1.4192	0.0338		0.1073	0.0005	down
**BdMAPKKK58**	59.4767	0.0002	up	123.5010	0.0000	up	74.5925	0.0002	up	217.7818	0.0000	up	87.4904	0.0000	up	66.3592	0.0000	up
**BdMAPKKK59**	67.3139	0.0000	up	26.2491	0.0000	up	20.0294	0.0000	up	0.4901	0.0004	down	14.5715	0.0000	up	0.0702	0.0000	down
**BdMAPKKK60**	0.2995	0.0000	down	0.3934	0.0028	down	0.1481	0.0210		0.2221	0.0001	down	0.0005	0.0000	down	0.1005	0.0000	down
**BdMAPKKK61**	0.4305	0.1967		10.2148	0.0205		0.0022	0.0004	down	2.6898	0.1630		7.1934	0.0226		7.1411	0.0249	
**BdMAPKKK62**	0.4461	0.1582		0.2958	0.0381		0.2687	0.0530		0.6825	0.4430		0.3918	0.0532		0.2089	0.0106	
**BdMAPKKK63**	1.9791	0.0022		0.8397	0.4445		0.8379	0.0321		2.2033	0.0002	up	1.2912	0.1866		1.3410	0.1563	
**BdMAPKKK64**	0.7359	0.0014		0.5335	0.0000		0.4061	0.0000	down	0.6766	0.0004		0.6181	0.0000		0.5865	0.0000	
**BdMAPKKK65**	0.5684	0.1090		0.4687	0.0546		0.3717	0.0183		0.8481	0.5028		0.5295	0.0473		0.4270	0.0262	
**BdMAPKKK66**	0.2572	0.0353		0.4534	0.0978		0.1344	0.0017	down	0.7346	0.5211		0.6131	0.1596		0.6665	0.2900	
**BdMAPKKK67**	0.5632	0.1844		0.2795	0.0131		0.1973	0.0039	down	0.9418	0.8471		0.5408	0.0513		0.3922	0.0296	
**BdMAPKKK68**	6.9316	0.0126		3.4126	0.0390		2.8640	0.0721		2.1548	0.2078		2.6647	0.1074		5.4533	0.0184	
**BdMAPKKK69**	29.6064	0.0003	up	16.1694	0.0001	up	16.1917	0.0001	up	26.9639	0.0002	up	5.9153	0.0009	up	3.4385	0.0045	up
**BdMAPKKK70**	1.0804	0.7305		0.6472	0.1076		0.4608	0.0221		1.7515	0.0797		0.7348	0.2438		0.4829	0.0390	
**BdMAPKKK71**	0.2448	0.0000	down	0.3512	0.0010	down	0.1428	0.0000	down	0.4836	0.0001	down	0.4855	0.0033	down	0.2515	0.0000	down
**BdMAPKKK72**	0.1778	0.0075	down	0.1438	0.0009	down	0.0789	0.0006	down	0.5046	0.0553		0.3031	0.0053	down	0.1406	0.0027	down
**BdMAPKKK73**	3.8417	0.0004	up	1.7212	0.1846		0.7187	0.0082		6.0975	0.0003	up	0.5200	0.0034		3.0967	0.0195	
**BdMAPKKK74**	0.6965	0.4692		0.2820	0.0734		0.4555	0.1499		0.9185	0.8493		0.4985	0.1935		0.5094	0.1324	
**BdMAPKKK75**	1.2167	0.1590		0.5304	0.0190		0.6257	0.0571		1.5676	0.0187		0.9606	0.7361		0.8798	0.3251	
