# Supplementary material for: Genome-wide exploration of the molecular evolution and regulatory network of mitogen-activated protein kinase cascades upon multiple stresses in Brachypodium distachyon
Source: BMC Genomics. 2015 Mar 24;16(1):228. doi: 10.1186/s12864-015-1452-1 (PMC4404688; doi:10.1186/s12864-015-1452-1)
Supplement: Additional file 9: — The co-expression regulatory MAPK cascade kinase genes. [file 12864_2015_1452_MOESM9_ESM.pdf]

Additional file 9 The co-expression regulatory MAPK cascades genes.

treatments		Co-expression regulatory MAPK cascades genes*												
Biotic	MAPKKKs	MAPKKK5<br>MAPKKK21<br>MAPKKK53<br>MAPKKK55<br>MAPKKK58<br>MAPKKK68<br>MAPKKK69	MAPKKK27<br>MAPKKK39<br>MAPKKK43	MAPKKK35<br>MAPKKK37<br>MAPKKK49<br>MAPKKK70<br>MAPKKK75	MAPKKK2<br>MAPKKK3<br>MAPKKK12<br>MAPKKK15<br>MAPKKK16<br>MAPKKK19<br>MAPKKK20<br>MAPKKK26<br>MAPKKK29<br>MAPKKK46<br>MAPKKK67<br>MAPKKK74	MAPKKK23<br>MAPKKK25<br>MAPKKK28<br>MAPKKK31<br>MAPKKK34<br>MAPKKK41<br>MAPKKK56<br>MAPKKK62<br>MAPKKK65	MAPKKK13<br>MAPKKK14<br>MAPKKK30<br>MAPKKK32<br>MAPKKK64	MAPKKK48<br>MAPKKK66<br>MAPKKK71<br>MAPKKK72	MAPKKK60					
	MAPKKs	MKK10-5		MKK10-2	MKK3-1<br>MKK3-2<br>MKK6	MKK5	MKK1<br>MKK3-3	MKK10-3						
	MAPKs		MPK14<br>MPK17	MPK20-1	MPK20-3<br>MPK20-4	MPK7-1<br>MPK20-2<br>MPK20-5	MPK4<br>MPK11<br>MPK21-1		MPK3<br>MPK6<br>MPK7-2<br>MPK16					
Abiotic	MAPKKKs	MAPKKK3<br>MAPKKK5<br>MAPKKK6<br>MAPKKK12<br>MAPKKK13<br>MAPKKK15<br>MAPKKK17<br>MAPKKK23	MAPKKK52	MAPKKK53<br>MAPKKK72	MAPKKK8<br>MAPKKK10<br>MAPKKK11	MAPKKK66<br>MAPKKK71	MAPKKK25<br>MAPKKK48	MAPKKK9<br>MAPKKK24<br>MAPKKK26<br>MAPKKK33<br>MAPKKK65	MAPKKK2<br>MAPKKK7<br>MAPKKK54<br>MAPKKK56<br>MAPKKK73	MAPKKK50<br>MAPKKK68				

		MAPKKK28<br>MAPKKK38<br>MAPKKK43<br>MAPKKK46												
	MAPKKs			MKK10-5		MKK5		MKK1	MKK6					
	MAPKs	MPK16	MPK3<br>MPK17		MPK4		MPK7-2	MPK11<br>MPK20-1	MPK20-4	MPK7-1<br>MPK20-2<br>MPK20-5				
Hormone	MAPKKKs	MAPKKK1<br>MAPKKK2<br>MAPKKK7<br>MAPKKK8<br>MAPKKK9<br>MAPKKK10<br>MAPKKK13<br>MAPKKK16<br>MAPKKK20<br>MAPKKK22<br>MAPKKK29<br>MAPKKK31<br>MAPKKK32<br>MAPKKK38<br>MAPKKK39<br>MAPKKK47<br>MAPKKK68<br>MAPKKK75	MAPKKK12<br>MAPKKK15	MAPKKK14<br>MAPKKK23<br>MAPKKK28<br>MAPKKK45<br>MAPKKK48<br>MAPKKK56<br>MAPKKK57<br>MAPKKK74	MAPKKK6<br>MAPKKK17<br>MAPKKK65	MAPKKK11<br>MAPKKK24<br>MAPKKK25<br>MAPKKK33	MAPKKK18<br>MAPKKK43	MAPKKK26<br>MAPKKK50	MAPKKK30<br>MAPKKK66<br>MAPKKK71	MAPKKK5<br>MAPKKK44<br>MAPKKK49	MAPKKK34	MAPKKK27	MAPKKK19<br>MAPKKK62<br>MAPKKK64<br>MAPKKK70	MAPKKK21<br>MAPKKK51<br>MAPKKK53<br>MAPKKK63<br>MAPKKK72
	MAPKKs		MKK10-3	MKK10-1		MKK1		MKK6	MKK4<br>MKK5		MKK10-5	MKK3-1<br>MKK3-2		MKK10-2
	MAPKs	MPK4			MPK7-1	MPK11	MPK7-2	MPK16	MPK6	MPK3		MPK21-1	MPK17	

					MPK20-5	MPK14<br>MPK20-4		MPK20-1<br>MPK20-2<br>MPK20-3						
Heavy Metal	MAPKKKs		MAPKKK3<br>MAPKKK8<br>MAPKKK11<br>MAPKKK13<br>MAPKKK18<br>MAPKKK26<br>MAPKKK36<br>MAPKKK48<br>MAPKKK50<br>MAPKKK61<br>MAPKKK66	MAPKKK10<br>MAPKKK19<br>MAPKKK20<br>MAPKKK32<br>MAPKKK35<br>MAPKKK37<br>MAPKKK45<br>MAPKKK51<br>MAPKKK52<br>MAPKKK58<br>MAPKKK67<br>MAPKKK69<br>MAPKKK71<br>MAPKKK72	MAPKKK25<br>MAPKKK57<br>MAPKKK63	MAPKKK28<br>MAPKKK42	MAPKKK1<br>MAPKKK22<br>MAPKKK29<br>MAPKKK30<br>MAPKKK34<br>MAPKKK39<br>MAPKKK40<br>MAPKKK41<br>MAPKKK49<br>MAPKKK53<br>MAPKKK64<br>MAPKKK74	MAPKKK16<br>MAPKKK27<br>MAPKKK31<br>MAPKKK4<br>MAPKKK73	MAPKKK14<br>MAPKKK44	MAPKKK60				
	MAPKKs	MKK1			MKK6	MKK5	MKK3-3<br>MKK10-2<br>MKK10-5	MKK4	MKK3-1<br>MKK3-2	MKK10-1<br>MKK10-3				
	MAPKs	MPK4<br>MPK11<br>MPK16<br>MPK20-1<br>MPK20-2<br>MPK20-4<br>MPK20-5	MPK17	MPK3<br>MPK6	MPK7-1<br>MPK20-3		MPK7-2<br>MPK14		MPK21-1					
Tissue	MAPKKKs	MAPKKK8<br>MAPKKK10	MAPKKK1<br>MAPKKK22	MAPKKK3<br>MAPKKK12	MAPKKK4<br>MAPKKK6	MAPKKK2<br>MAPKKK11	MAPKKK9<br>MAPKKK23							

		MAPKKK16<br>MAPKKK37<br>MAPKKK44<br>MAPKKK47<br>MAPKKK49<br>MAPKKK75	MAPKKK31<br>MAPKKK32<br>MAPKKK38<br>MAPKKK40<br>MAPKKK64	MAPKKK14<br>MAPKKK19<br>MAPKKK27<br>MAPKKK34<br>MAPKKK35<br>MAPKKK39<br>MAPKKK51<br>MAPKKK66	MAPKKK7<br>MAPKKK18<br>MAPKKK24<br>MAPKKK26<br>MAPKKK30<br>MAPKKK33<br>MAPKKK41<br>MAPKKK43<br>MAPKKK45<br>MAPKKK52<br>MAPKKK54<br>MAPKKK55<br>MAPKKK57<br>MAPKKK58<br>MAPKKK61<br>MAPKKK71<br>MAPKKK72<br>MAPKKK73	MAPKKK15<br>MAPKKK17<br>MAPKKK20<br>MAPKKK28<br>MAPKKK29<br>MAPKKK36<br>MAPKKK42<br>MAPKKK46<br>MAPKKK50<br>MAPKKK63<br>MAPKKK67<br>MAPKKK74	MAPKKK25<br>MAPKKK48<br>MAPKKK53<br>MAPKKK56<br>MAPKKK59<br>MAPKKK60<br>MAPKKK62<br>MAPKKK65<br>MAPKKK68<br>MAPKKK69<br>MAPKKK70							
	MAPKKs		MKK3-1<br>MKK3-3<br>MKK6<br>MKK10-2	MKK1<br>MKK3-2	MKK4<br>MKK5<br>MKK10-5	MKK10-3	MKK10-1							
	MAPKs	MPK17	MPK3<br>MPK6<br>MPK7-2<br>MPK14<br>MPK20-4	MPK4<br>MPK16<br>MPK20-1<br>MPK20-3	MPK7-1	MPK11<br>MPK20-2<br>MPK20-5<br>MPK21-1								
All	MAPKKKs	MAPKKK23<br>MAPKKK25	MAPKKK57<br>MAPKKK66		MAPKKK35<br>MAPKKK37		MAPKKK24<br>MAPKKK33	MAPKKK26	MAPKKK21<br>MAPKKK55	MAPKKK63	MAPKKK6			

		MAPKKK28<br>MAPKKK48<br>MAPKKK56	MAPKKK71		MAPKKK39<br>MAPKKK49		MAPKKK50							
	MAPKKs		MKK5	MKK6		MKK3-1<br>MKK3-2<br>MKK3-3		MKK1<br>MKK10-3	MKK10-5	MKK10-2				
	MAPKs	MPK4		MPK6<br>MPK16<br>MPK20-3<br>MPK20-4	MPK3<br>MPK17	MPK7-2<br>MPK21-1	MPK7-1<br>MPK20-2<br>MPK20-5	MPK11			MPK20-1			
All+Tissue	MAPKKKs	MAPKKK2<br>MAPKKK13<br>MAPKKK15	MAPKKK24<br>MAPKKK33		MAPKKK43<br>MAPKKK55<br>MAPKKK57<br>MAPKKK59<br>MAPKKK60<br>MAPKKK61<br>MAPKKK68<br>MAPKKK73	MAPKKK14<br>MAPKKK29<br>MAPKKK34	MAPKKK20<br>MAPKKK30<br>MAPKKK36<br>MAPKKK41<br>MAPKKK45<br>MAPKKK46<br>MAPKKK71<br>MAPKKK72<br>MAPKKK74	MAPKKK26<br>MAPKKK50<br>MAPKKK66	MAPKKK53<br>MAPKKK58<br>MAPKKK69					
	MAPKKs		MKK1	MKK6	MKK10-1		MKK5	MKK4<br>MKK10-3	MKK10-5					
	MAPKs	MPK4	MPK7-1<br>MPK20-2<br>MPK20-5	MPK20-1<br>MPK20-4		MPK16<br>MPK20-3<br>MPK21-1	MPK11							

* The same color background indicated the same group of co-expression genes in corresponding treatment, respectively. “All” means the expression levels of all kind of treatment, and “Tissue” means the tissue specific expression. MAPKKK genes were marked in blue; MAPKK genes were marked in red; and MAPK genes were marked in cyan.
