# Supplementary material for: Genome-wide exploration of the molecular evolution and regulatory network of mitogen-activated protein kinase cascades upon multiple stresses in Brachypodium distachyon
Source: BMC Genomics. 2015 Mar 24;16(1):228. doi: 10.1186/s12864-015-1452-1 (PMC4404688; doi:10.1186/s12864-015-1452-1)
Supplement: Additional file 10: — The list of qRT-PCR primers of MAPK cascade kinase genes. [file 12864_2015_1452_MOESM10_ESM.pdf]

Additional file 10 The list of qRT-PCR primers of MAPK cascades genes.

Genename	Genemodel	ForwardPrimer	RewardPrimer
BdMAPKKK1	Bradi5g24870	CCTGTCGAACCTGTTCCCTC	CCACATAAAGACGAAAGTGGGC
BdMAPKKK2	Bradi1g28950	GTCTTGCAGCGCAACAAAAAT	TCCATGGAAGGGTGCCTCTC
BdMAPKKK3	Bradi3g60210	GCTGTTCCTGCATTACCCCT	ATGCTGGCTTCATGCTCTGT
BdMAPKKK4	Bradi1g47570	CGGCTCAGGTTGCTGGTTAT	GCTGTTGCGGCACATAAGAG
BdMAPKKK5	Bradi3g59510	AAGCGGTTGCGTCATCCTAA	TGCCATTCTTAAGCGCCTCT
BdMAPKKK6	Bradi1g74480	GCTGCAGGATCAACCACCAT	TGTCCATGAATCTGTCGAGTGG
BdMAPKKK7	Bradi1g45040	GCATCTTCAATGGTCGTCGC	CCGGACAGATTCACTTGCCT
BdMAPKKK8	Bradi5g18180	AAGCTGACATTCTGAGCCCC	ATGCCTGAGTCCAAGGTGTG
BdMAPKKK9	Bradi1g30720	AGGGGCAAGCTCATACCAAC	TTGCTCAGATGGCTTCCCAG
BdMAPKKK10	Bradi2g46340	TCGGTCTTTCTCGAGCGATG	AGCCGTGACCCTTCATTAGC
BdMAPKKK11	Bradi3g51380	GTGGTCTCTGAAGCTGGCAT	GGTTGCGTATGAGGTGTGGA
BdMAPKKK12	Bradi3g27120	TGGCCAGAGGCATGAATTGT	ATTGCTCGTTCCGCAAAACC
BdMAPKKK13	Bradi3g09170	CCTTGGCGCATGTATGAAGC	ACCCCTACAGATGTCACGGA
BdMAPKKK14	Bradi3g08260	TCTGGATTTCGAACAGTGGAGG	CGCCAGAAACTTTTGATTAGCA
BdMAPKKK15	Bradi4g04470	GCTTTGGATGTTGCAAGGGG	CATCCATTGAGGCGTTCCCT
BdMAPKKK16	Bradi1g23970	AGAGGTCCTTCTGATCGCCT	CCTACTTGCCCCGTTTGAGT
BdMAPKKK17	Bradi4g38400	CCTGTGAAAGGGCAAGCAAC	ATGCCAGTCAGCACGATGAA
BdMAPKKK18	Bradi3g44710	TGTTGCTGTCAGCTTCTCGT	AGGCTGGCTGCTAAGAGAGA
BdMAPKKK19	Bradi1g07650	CTGGGTCATTGGCTCATCGT	ATGAGCTGCAGAACCAGGAC
BdMAPKKK20	Bradi5g21330	ATGGCACCAGAGTTTCTCCG	TTTTGGAATGCAACCGCTCC
BdMAPKKK21	Bradi4g36880	GTCTTCAAGCTCCCTGCCTT	TGTTCAATGACCTCTGGGGC
BdMAPKKK22	Bradi2g39350	CATACCGGCACCAAGGAAGT	AGTGCTCTGCCAGTGAATCC
BdMAPKKK23	Bradi4g29500	GCTGCAGCAAGCAATTCTCA	ACTTTCTTTGCCTCTCCGCA
BdMAPKKK24	Bradi1g60340	AGGGCACAAGGGAATGAAGG	TGCTCCTGTAGCTTGATGGC
BdMAPKKK25	Bradi3g36080	GTGCACAGCTTCGCCATTAG	GGCAGCCTTCATTCCGTAGT
BdMAPKKK26	Bradi1g58810	GCCGTCCTTGCAAATACCCA	GGGCTGCATACAGGACTTGA
BdMAPKKK27	Bradi4g22760	GTGCTAGAGGAAGGGCGTTT	TTTCTGTCGATTTCCGGCCA
BdMAPKKK28	Bradi3g51460	TTCTGTGCAGCTTGTTGGGA	CCAGTTCTGAACCAGGCGAT
BdMAPKKK29	Bradi1g10970	GGATGGCACCTGAGGTTGTT	AGCTGGGAATAGGAGGTCGT
BdMAPKKK30	Bradi3g01850	TGATGATTCCTTCGACCCCG	GGTGAGCCCTGAAAGAACCA
BdMAPKKK31	Bradi2g06260	CCCGACAAGAGGCCAGATTT	CCAACCGGATAAAACAGCGG
BdMAPKKK32	Bradi2g19590	ATGACTCCAGTCCAAGCAGC	ACTCGGGCCTCTTATCTGGT
BdMAPKKK33	Bradi3g48360	GGAAGTACGCCGGCATTAGA	TTCAAGCGGGTTCAGCTCAT
BdMAPKKK34	Bradi1g67400	ACTTATGCCAAGGCAGGACC	GCAGAACGGTCGATACATGC
BdMAPKKK35	Bradi2g49700	GGATGGCTCCGGAGATGATG	AGCTAACAGGGATGGTTGGC
BdMAPKKK36	Bradi2g57470	TGAGCTCCACAGGAACGAAC	TAGCCATGGTGTGTGTGTCC
BdMAPKKK37	Bradi3g05520	TGGGATTGTCTTGTGGGAGC	CGCATTCTCAAGCATGCACA
BdMAPKKK38	Bradi3g18150	AGGAAGCCAATGGTCTGGTG	TGCCACGTGCAACATCTAGT
BdMAPKKK39	Bradi1g28110	GGGAGCTACTGGCAGGAAAG	TTGGTCTTAGGGCGGGATCT
BdMAPKKK40	Bradi2g49790	AAAATCCTGCTGACAGGCCA	CCCTCGAAGCTCAGGAAACA
BdMAPKKK41	Bradi1g14000	TGAGGTCACAAAGGCCTCAC	AGAAAATGACATCTGCTGAACACC
BdMAPKKK42	Bradi1g35350	ACAGCATGGAATCCTGGTCG	GTAGATGCCCCGGTAGATGC
BdMAPKKK43	Bradi1g04080	AGATTGGGGAGGGAGCTCAT	GTTCCTTGCAAGCCCCAATG
BdMAPKKK44	Bradi5g10670	TACCTCCAGCAGCCCAATTC	TCCTGCCAAGGGTCTGAAAC

BdMAPKKK45	Bradi3g47600	TGTCTAAATTGCCCGCCCAT	CACCAACCCCACTTCTAGCC
BdMAPKKK46	Bradi2g44910	TCAGCATTCGCACAAGAGGT	CTCAACCACAACGCAGCAAA
BdMAPKKK47	Bradi2g15560	GGGGCCCTTGATGGATGAAT	GGGCTTGTCGAGAGATCACA
BdMAPKKK48	Bradi2g00670	GATATCCCAATGCTGGGCGA	GAGATGCAGATCGTCGGAGG
BdMAPKKK49	Bradi1g20390	GAGCTATGTTGGGATGCGGA	AATGCCAGTGGTGATCGTGT
BdMAPKKK50	Bradi4g44430	CGCATGTTCTTCAACACCGT	TAATTGTTGAGCTTGCCGCC
BdMAPKKK51	Bradi2g47510	TTCGGTCCTTGGAGTGAAGC	CTAGCCACAAGGACACCCAG
BdMAPKKK52	Bradi2g47480	TAGTGTCGCTGGCGACTGA	GACAGTATCCCTTGCTCCCG
BdMAPKKK53	Bradi2g47490	GCGGGTGTGCTGTAGGATTA	TTCGGCGGAACTTGTCAGAT
BdMAPKKK54	Bradi1g23320	GTGCAGATCTACCACAGCGT	GGAGAAAAAGGGGTCGAGCA
BdMAPKKK55	Bradi2g47500	TGGATTCCTTGCTCCCGATG	GGCGAAGAGATCGCTCATGT
BdMAPKKK56	Bradi4g41940	AGCTCCAGTGGCTGAAAGAC	TTCAGCAGGAAATCGCCCTT
BdMAPKKK57	Bradi3g10890	GGCCACACAAAGAGATGGGA	CATGCCAAATCCGTCCGTTC
BdMAPKKK58	Bradi2g17820	GAAGCAGATAATAATTCTTTG	ATGGTACAGCTTCGTCGGCT
BdMAPKKK59	Bradi2g17830	TGCAAGTTCGGATTCCGCTA	TCACGAGTTGAGGGCTTTGC
BdMAPKKK60	Bradi1g65500	ATCGACTCGACGAAACTCGC	GACCAGACGTCAGAAGCCG
BdMAPKKK61	Bradi1g41850	AATTAGCAACCCTCAGGCCC	ACTGGCGTGTGCTCTACTTC
BdMAPKKK62	Bradi4g09990	GAGCACAGATAGCCAGGGTC	CTAACGCCTCGACTGTCCTC
BdMAPKKK63	Bradi3g45660	GGAAATTCCAAGCGACGCAG	ACGCTCATCATCTTTTCGGC
BdMAPKKK64	Bradi4g02900	CTTGACTGGCGAGGAACCAT	AGCATTGCTCCATCAGGCTT
BdMAPKKK65	Bradi1g00580	TGCACCTATGGGGGACAGTA	CACGGTTCCAAAAGTGCCAG
BdMAPKKK66	Bradi1g14010	AAACTGCTGCTGCCTTTGTG	TGCCATCATGCTAACCACACA
BdMAPKKK67	Bradi3g45790	TCCCCAGTAGAGGCAAGACA	TTGAGGCGAAGGGTAAGCTG
BdMAPKKK68	Bradi2g17800	ATAGACAGGGCAATTGGGGC	CACGCATGCGAAAGTCAAC
BdMAPKKK69	Bradi2g17840	GACAGCAGCGATGTAGGACA	ACCTGAAACTTTGGGCGAGAG
BdMAPKKK70	Bradi3g57740	AGGCTCTTTGGCGGCTTTAT	TGACCGACCCATTTGCATCA
BdMAPKKK71	Bradi4g24830	GAGAGCGCTACATCTGGTCG	TACTCTGCTTTCCCGGCATC
BdMAPKKK72	Bradi4g24840	GATGCCGGGAAAGCAGAGTA	ACATGACGATATGCGGGTGT
BdMAPKKK73	Bradi3g13050	CGCAGGATATACGAGCACCT	AGGATACTCCGGTGGAGGAG
BdMAPKKK74	Bradi3g13060	TAGCTGCTGCTTTTCCCCAA	AGAAGGAACTACACGCTGCC
BdMAPKKK75	Bradi4g41870	GCTGGGAAACCATCCCATCA	TTTGCCGAGGTGGAGACATC
BdMKK1	Bradi1g51000	CAAATTCCTGACGCAGAGCG	CGAACCAGTTGCACGATTCC
BdMKK3-1	Bradi4g39490	ACATTCCAAGCAAGCGGGTA	CCAATAAGGTTCCCACCATG
BdMKK3-2	Bradi1g41860	GAGATCCATGGGGTGCTACT	AAGGTTTCCACCATGACAGC
BdMKK3-3	Bradi3g11260	ATAGCCATCGAGGAATCGC	ATGCAATGCACTTCCTCTC
BdMKK4	Bradi3g53650	AGGTTCGTCCCTATCGTCCA	GGTTCCCGTAGAGCACCTTG
BdMKK5	Bradi1g46880	TGAAGCAGCTCTACGGGAAC	TTGGATTGATCCTGGGACCG
BdMKK6	Bradi1g75150	TGGCCGCTTCCCCTATACTC	CGCGGGATCCTTTTGTATG
BdMKK10-1	Bradi1g11525	GTACGCCCTCAAGGTGCAG	GGAACTGGGAGAACGCCTC
BdMKK10-2	Bradi1g69400	GGGACTTGTAGGCGAAACGA	CGCCACATGGGACAGGTTAT
BdMKK10-3	Bradi1g10800	GTGCTGTCTGACGGTGAGG	CAACAAACGGGTGAGCCAG
BdMKK10-5	Bradi1g10790	GGAGCTCAGGGGATTCATCG	CCATATTCCTCCCGGCGAC
BdMPK3	Bradi1g65810	TCCCCTCCAGAGGATCACAG	TGGTCTTCCGTCAAAGGGTG
BdMPK4	Bradi3g32000	TCAGCCATTGACCGATGACC	CAAGCCCAAAGTCCGCAATC
BdMPK6	Bradi1g49100	TGGAGCTCAGTTTCGGCATT	TACGCAGAAGTTCGACCACC
BdMPK7-1	Bradi1g34030	GCCTCTTCAGATCTGCTGCT	TCTTCCGATGGGCTTGATCG

BdMPK7-2	Bradi4g24912	TATGACCCAAGTGCGAACCC	GCCGAACATGTCAAGACCCT
BdMPK11	Bradi3g16560	TGTGCACTCAGCGAATGTCT	CCGATACCACCGGGTAACAA
BdMPK14	Bradi3g03780	GCCCATCGACCTCGACTTAG	ATCGCAGTAGGGCCTTTGAG
BdMPK16	Bradi2g36470	ACTTGAGCAGTATGAGGCGG	GCGGAAGGCTCTCTATCCAC
BdMPK17	Bradi1g34700	AGAAAGAGGTTCTCCGCTGC	TTGCGCTCTTCTGGTAGCTC
BdMPK20-1	Bradi2g44350	CGTATTGGCCCATCAAGGGA	TACGGATGACTGGTGCCAAC
BdMPK20-2	Bradi2g15317	GCTCTGTGGATCCTTTTTCACG	TACGGACCCGCGAAATTGTA
BdMPK20-3	Bradi1g41780	CTGCCAAGGCAAGCCTTCAAC	CAGGCACATGTTGTGGTGGG
BdMPK20-4	Bradi2g45870	AACTGGGAAGCCTTTGTTCC	AGGGTCTGCTTTGGGGAATC
BdMPK20-5	Bradi2g16337	CGGTTGCATTTTTGCGGAGA	GCGCTGAAGGATCTGCATTG
BdMPK21-1	Bradi2g15620	AAGCATGGAGATGGTGACTACA	CTCCGAAAGGGAATCCTCATC
BdActin	Bradi2g24070	CCCGATGGACAGGTTATCACTA	ATAGAGCCACCAATCCAAACAC
